# All-Carbon [3+3] Oxidative Annulations of 1,3-Enynes by Rhodium(III)-Catalyzed C–H Functionalization and 1,4-Migration**

**DOI:** 10.1002/anie.201503978

**Published:** 2015-07-14

**Authors:** David J Burns, Daniel Best, Martin D Wieczysty, Hon Wai Lam

**Affiliations:** School of Chemistry, University of Nottingham, University Park Nottingham, NG7 2RD (UK) E-mail: hon.lam@nottingham.ac.uk; EaStCHEM, School of Chemistry, University of Edinburgh, Joseph Black Building, The King's Buildings David Brewster Road, Edinburgh EH9 3FJ (UK)

**Keywords:** allylation, C–H activation, enynes, homogeneous catalysis, rhodium

## Abstract

1,3-Enynes containing allylic hydrogens cis to the alkyne function as three-carbon components in rhodium(III)-catalyzed, all-carbon [3+3] oxidative annulations to produce spirodialins. The proposed mechanism of these reactions involves the alkenyl-to-allyl 1,4-rhodium(III) migration.

Transition metal-catalyzed oxidative annulations of alkynes[1] that proceed by directing group-promoted C(sp^2^)–H functionalization[1, 2] are versatile methods for heterocycle[3] and carbocycle[4] synthesis. Alkynes, including 1,3-enynes,[5] serve as two-carbon components in these reactions (Scheme 1 a). However, analogous reactions that result in three-carbon annulation are currently underdeveloped,[6] and addressing this shortcoming would expand the range of products accessible using C–H functionalization/oxidative annulation chemistry.

**Scheme 1:**
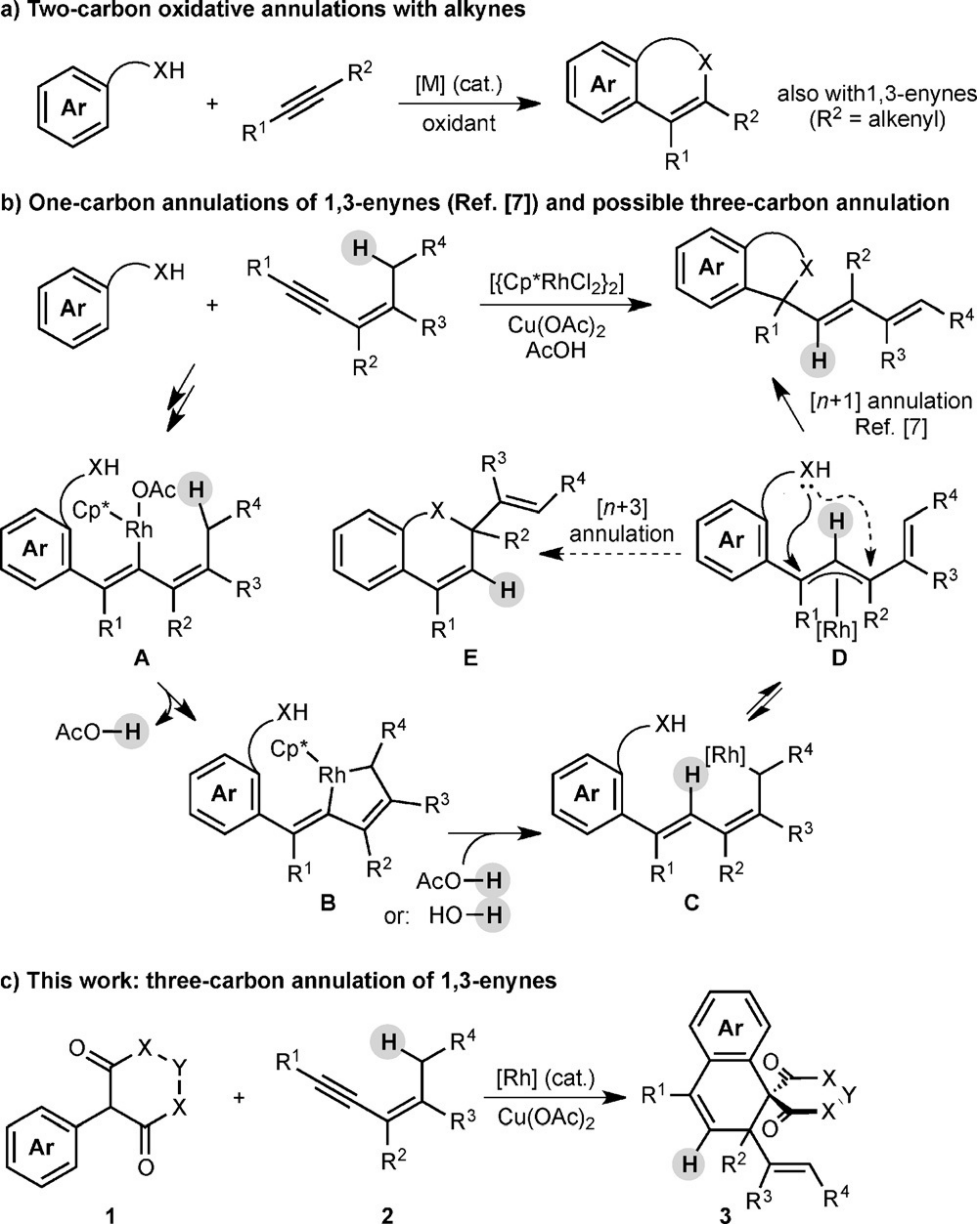
Catalytic oxidative annulations of alkynes and 1,3-enynes.

Using rhodium(III) catalysis, we recently discovered a new mode of oxidative annulation of 1,3-enynes that contain allylic hydrogens *cis* to the alkyne, in which they act as one-carbon components (Scheme 1 b).[7] The proposed mechanism[7] involves the 1,4-rhodium(III) migration[8, 9] of alkenylrhodium species **A** to give σ-allylrhodium(III) species **C** via rhodacycle **B**. Following isomerization of **C** into the electrophilic π-allylrhodium(III) species **D**, nucleophilic trapping by the directing group gives the product of [*n*+1] annulation. Given the isomerization of **C** into **D**, there exists the possibility for cyclization to occur at a different position of the extended π-system to give **E**, a product of [*n*+3] annulation (Scheme 1 b).[6]

Herein, we describe the realization of this possibility in rhodium(III)-catalyzed reactions of 2-aryl cyclic 1,3-dicarbonyls **1** with 1,3-enynes **2** to give spirodialins **3** (Scheme 1 c). The majority of the products obtained are spirocyclic barbiturates, which are of interest given the well-established medicinal importance of the barbiturate motif, and the biological activity of structurally related spirocycles (Figure 1).[10]

**Figure 1:**
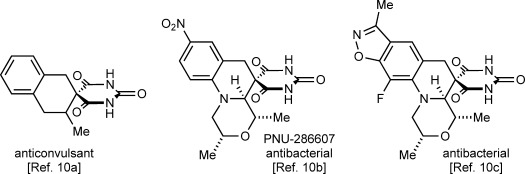
Biologically active spirocyclic barbiturates.

During our studies of metal-catalyzed oxidative annulations of alkynes,[4a,b,e, 7] the reaction of 5-arylbarbituric acid **1 a** with 1,3-enyne **2 a** was performed using [{Cp*RhCl_2_}_2_] (2.5 mol %) and Cu(OAc)_2_⋅H_2_O (2.1 equiv) in dioxane/H_2_O (5:1) at 60 °C [Eq. (1)]. As well as providing the spiroindene **4 a** through a standard two-carbon annulation,[4a,b,e] a [3+3] annulation occurred to give spirodialin **3 a** as the major product. No one-carbon annulation product **5**[7] was detected. Chromatographic purification gave a 72:28 mixture of **3 a** and **4 a** in 92 % yield. Without H_2_O, more side products were formed and the ratio of **3 a**:**4 a** decreased to ca. 50:50. No reaction occurred without Cu(OAc)_2_⋅H_2_O.[11]


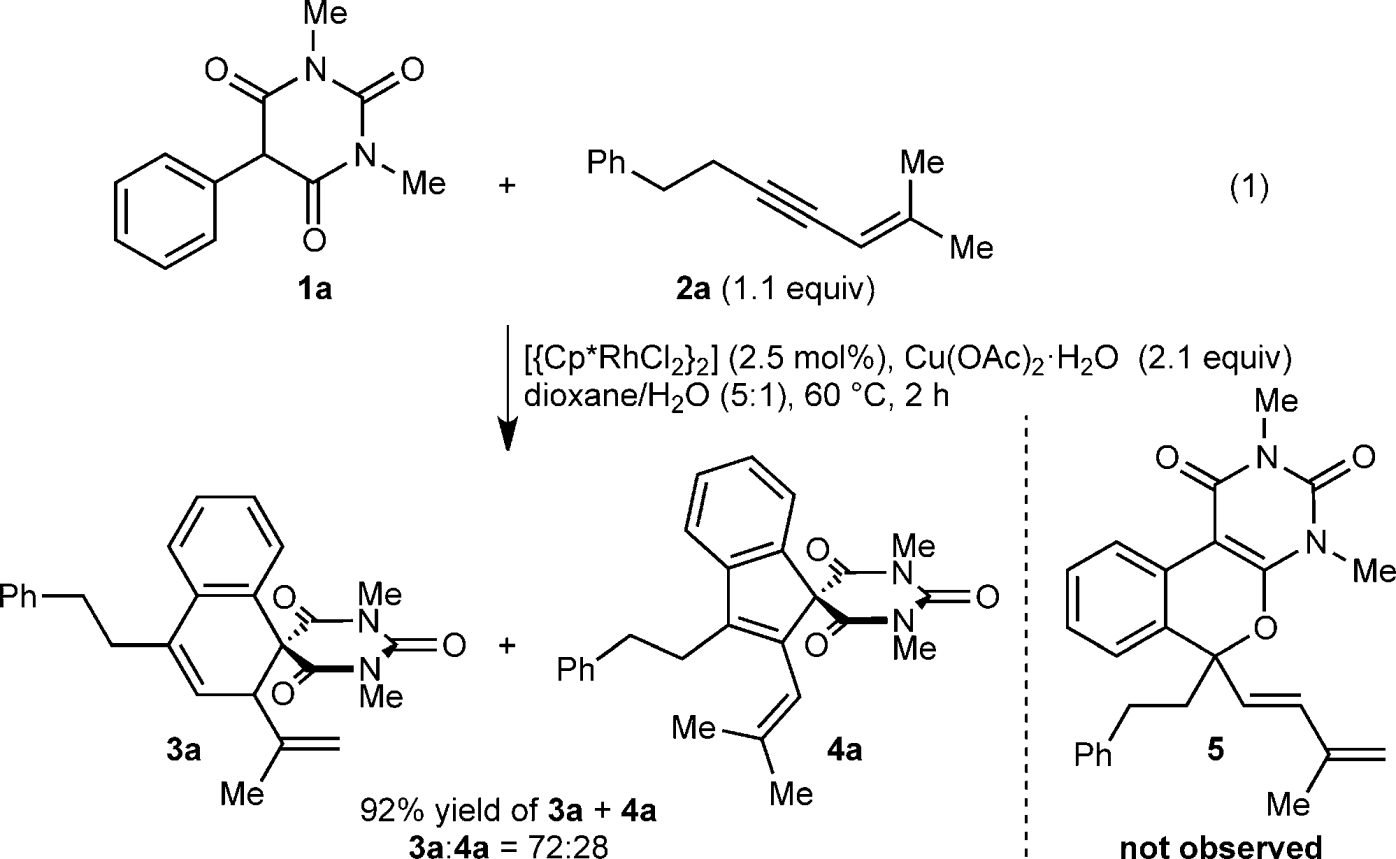
(1)

Further studies revealed the benzyloxy-containing 1,3-enyne **2 b** to be superior to **2 a**; the reaction of **2 b** with **1 a** gave spirodialin **3 b** only, in 88 % yield as the *E*-isomer (Scheme 2). Reaction of **2 b** with various 5-arylbarbituric acids[12] demonstrated compatibility with nitro (**3 c**), acetoxy (**3 d**), and halogen substituents (**3 f**–**3 h**) on the aryl group.[13] Spirodialin **3 e** was not formed under the standard conditions,[14] but replacing dioxane/H_2_O with undried DMF enabled productive [3+3] annulation and isolation of **3 e** in 37 % yield, along with several side products.[14] Free N–H groups on the barbituric acids were also tolerated (**3 i** and **3 j**). In the latter case, **3 j** was formed as a 1:1 inseparable mixture of diastereomers. The reaction of 2-phenyl Meldrum’s acid also gave [3+3] annulation, but the yield of **6** was only 32 % due to decomposition of the starting material and product under the acidic conditions.[15] Decreasing the loading of [{Cp*RhCl_2_}_2_] to 0.5 mol % in the reaction of **1 a** with **2 b** was well-tolerated and provided **3 b** in 77 % yield.

**Scheme 2:**
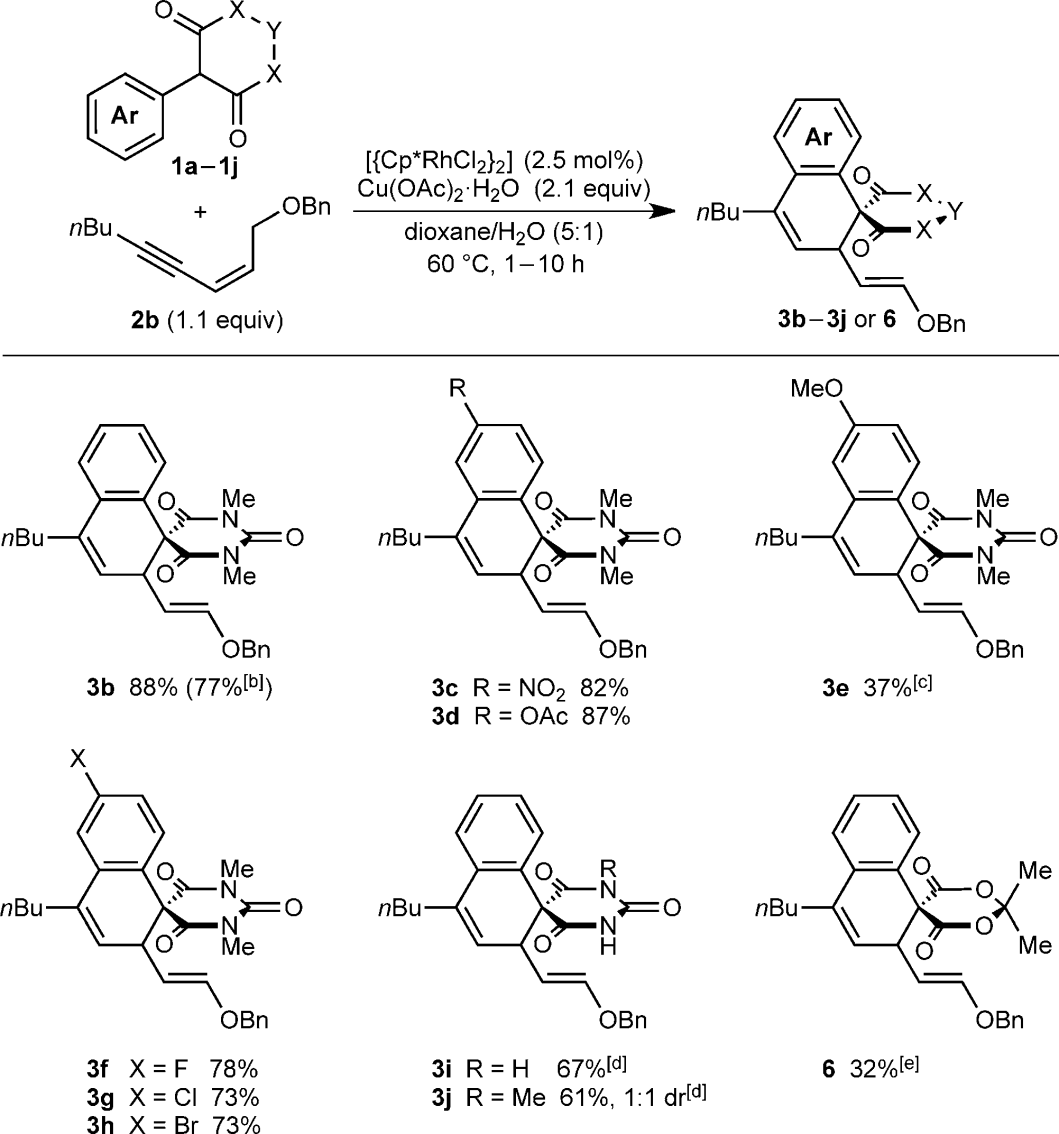
[a] Conducted with 0.50 mmol of 1 a–1 j. [b] Yield of isolated products. [b] Conducted with 0.5 mol % of [{Cp*RhCl_2_}_2_]. [c] Conducted in undried DMF. Side products were also obtained; see Ref. [14]. [d] Conducted at 120 °C. [e] Conducted with 5 mol % of [{Cp*RhCl_2_}_2_].

Interestingly, Cu(OAc)_2_⋅H_2_O rapidly decomposed cyclic hydrazide **7**, precluding its use as the oxidant in the reaction with 1,3-enyne **2 b** [Eq. (2)]. However, reaction of **7** (2.0 equiv) with **2 b** without Cu(OAc)_2_⋅H_2_O but with inclusion of NaOAc⋅3H_2_O (3.0 equiv) gave spirodialin **8** in 47 % yield, along with **2 b** (30 % recovery). We speculate that the N–N bond of **7** could be serving as an oxidant to regenerate the catalyst,[16] but we were unable to isolate the reduced form of **7** to confirm this hypothesis.


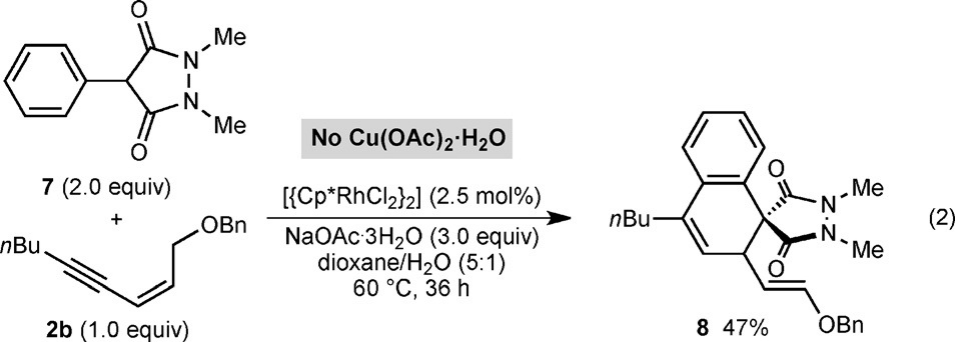
(2)

Table 1 presents the results of oxidative annulations of 5-arylbarbituric acids with various 1,3-enynes. No spiroindenes or benzopyrans from two- or one-carbon annulations, respectively, were detected. 1,3-Enynes **2 c** and **2 d**, containing protected or unprotected 2-hydroxyethyl groups on the alkyne were tolerated (entries 1 and 2). Use of a methoxy group in the 1,3-enyne in place of a benzyloxy group was also possible (entry 3). With a 5-(4-nitrophenyl)-substituted barbituric acid, oxidative annulations with 1,3-enynes **2 a**, **2 f**, and **2 g** containing various groups *trans* to the alkyne proceeded efficiently to give spirodialins **3 n**–**3 p** in 72–95 % yield (entries 4–6). As with the corresponding one-carbon annulations,[7] the 4-nitrophenyl group favors 1,4-rhodium(III) migration over the formation of spiroindenes [compare with Eq. (1)]. 1,3-Enynes **2 h** and **2 i** containing cyclic groups were also competent substrates (entries 7 and 8), and spirodialin **3 r** was isolated in 57 % yield, despite containing a potentially acid-sensitive enol acetal.

**Table 1:** [3+3] Oxidative annulations of various 1,3-enynes^[a]^ 
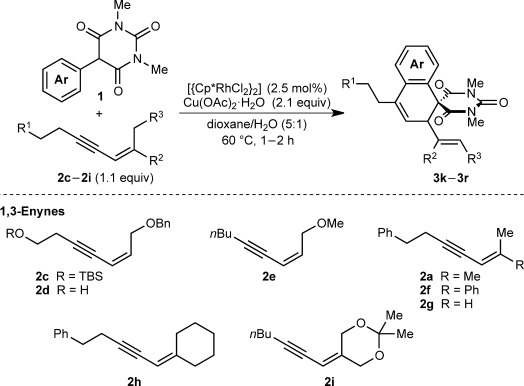

Entry	1,3-Enyne	Product		Yield [%]^[b]^
1 2	**2 c 2 d**	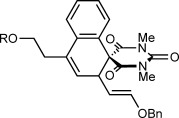	**3 k** R=TBS **3 l** R=H	60 64
				
3	**2 e**	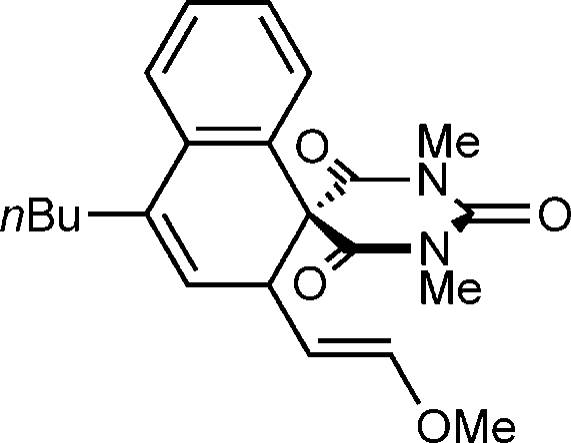	**3 m**	80
				
4 5 6	**2 a 2 f 2 g**	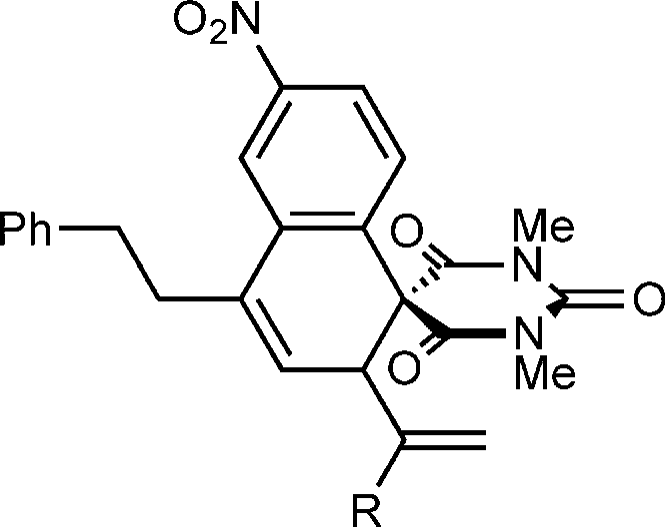	**3 n** R=Me **3 o** R=Ph **3 p** R=H	95 78 72
				
7	**2 h**	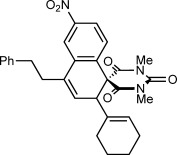	**3 q**	86
				
8	**2 i**	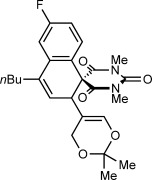	**3 r**	57

[a] Conducted with 0.50 mmol of **1**. [b] Yield of isolated products.

Notably, the formation of a highly sterically hindered spirodialin **3 s** containing contiguous all-carbon sp^3^ quaternary centers from 1,3-enyne **2 j** occurred efficiently [Eq. (3)]. The reaction of **1 b** with 1,3-enyne **9**, which does not contain any *cis*-allylic hydrogens, led only to the formation of spiroindene **4 b** in 82 % yield, thus highlighting the importance of this structural feature for [3+3] annulation [Eq. (4)].


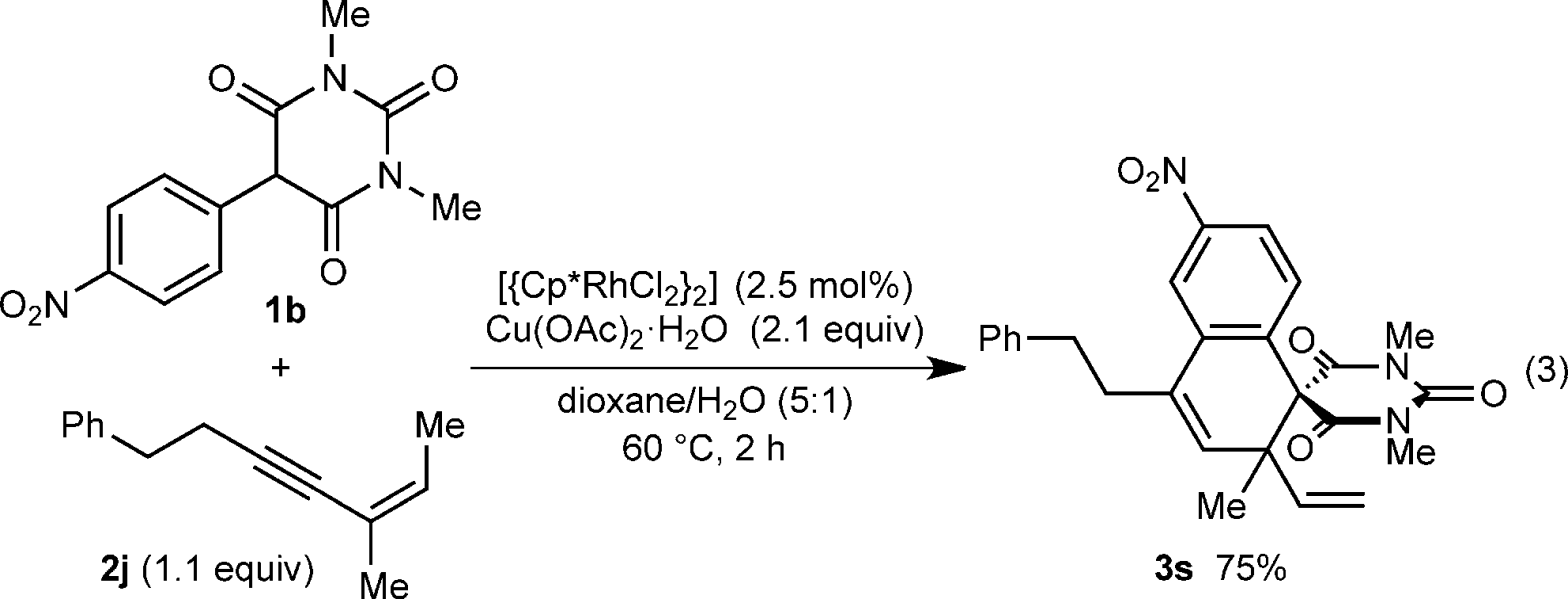
(3)


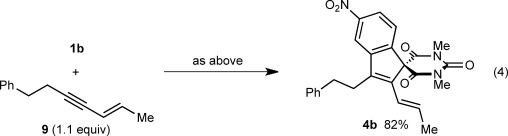
(4)

Scheme 3 depicts a possible catalytic cycle for these reactions, using representative substrates **1 a** and **2 a**. This cycle is similar to that proposed for the one-carbon annulations we described previously.[7] Cyclorhodation of **1 a** with rhodium diacetate **10** would give rhodacycle **11**. Migratory insertion of 1,3-enyne **2 a** then provides rhodacycle **12**, which upon reductive elimination would give spiroindene **4 a**. However, reversible protonolysis of **12** forms alkenyrhodium species **13**, which can then undergo 1,4-rhodium(III) migration to form σ-allylrhodium(III) species **14**. This intermediate can lead to π-allylrhodium(III) species **15** by a series of σ–π–σ interconversions and *E*/*Z* isomerization. Outer sphere nucleophilic attack of the π-allylrhodium(III) moiety[17, 18] of **15** by C5 of the barbituric acid then gives spirodialin **3 a** and rhodium(I) species **16**, which undergoes Cu(OAc)_2_-promoted oxidation to regenerate **10**. The preference of 5-monosubstituted barbituric acids for *C*-allylation over *O*-allylation has been observed previously in Pd-catalyzed asymmetric allylic alkylations.[19] However, an alternative pathway involving an inner-sphere reductive elimination cannot be excluded.

**Scheme 3:**
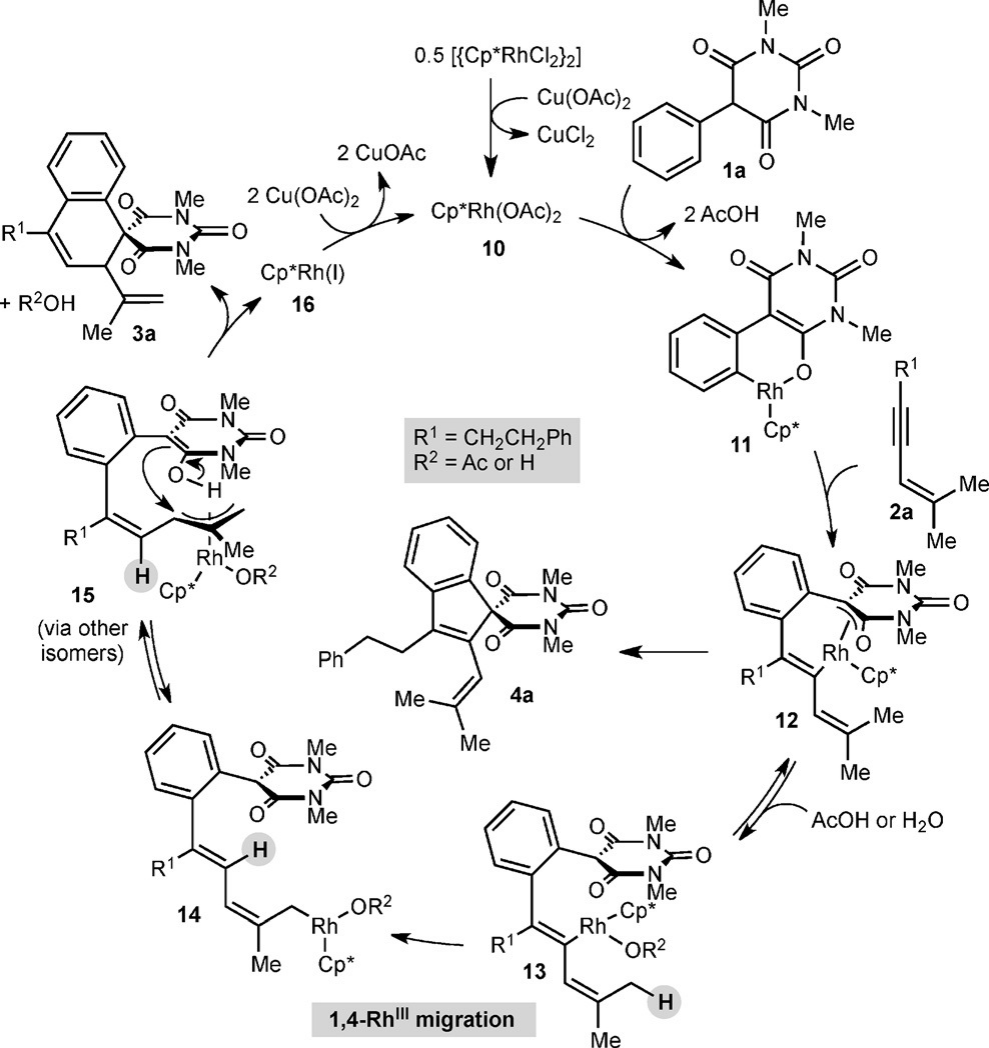
Possible catalytic cycle.

The reaction of **1 a** with 1,3-enyne **2 k** gave spiroindene **4 c** only (Scheme 4), a result that differs from the formation of spirodialins **3 b** (Scheme 2) and **3 m** (Table 1, entry 3) from 1,3-enynes **2 b** and **2 e**, respectively. A possible explanation for this contrasting behavior might be coordination of the acetoxy group to rhodium, resulting in stabilization of 18-electron intermediates such as rhodacycles **17** and **18** (analogous to **12** in Scheme 3, but the σ-haptomers) or alkenylrhodium species **19**. This stabilization likely disfavors 1,4-rhodium(III) migration and leads instead to reductive elimination from **18** to give **4 c**.

**Scheme 4:**
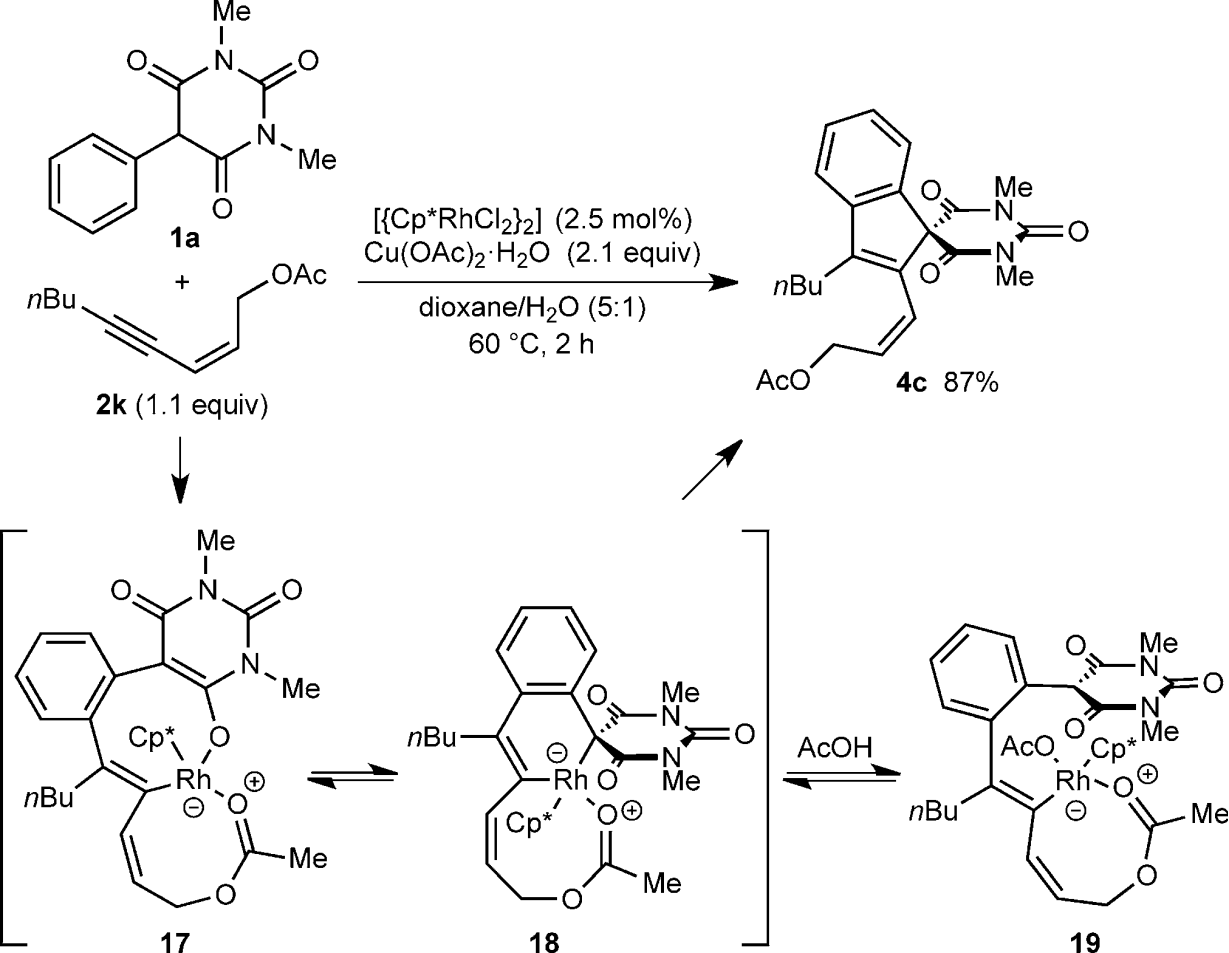
Formation of spiroindene 4 c from 1,3-enyne 2 k.

The reaction of **1 b** with the hexadeuterated 1,3-enyne [D]_6_-**2 a** gave traces of a spiroindene [D]_6_-**4 d** (<5 %), and spirodialin [D]_6_-**3 n** in 88 % yield (Scheme 5 a), in which incomplete deuterium transfer (91 % D) from the *cis*-allylic position of [D]_6_-**2 a** to the alkenyl position of the dialin ring of [D]_6_-**3 n** was observed. Furthermore, the reaction of **1 b** with **2 a** in 5:1 dioxane/D_2_O led to 10 % deuteration at the same position of [D]_*n*_-**3 n**, with no spiroindene detected (Scheme 5 b). These results are similar to the corresponding experiments with [D]_6_-**2 a** in the one-carbon annulations reported previously,[7] and are consistent with 1,4-rhodium(III) migration occurring by a concerted metalation-deprotonation/reprotonation sequence (similar to **A** to **C** in Scheme 1 b).[7]

**Scheme 5:**
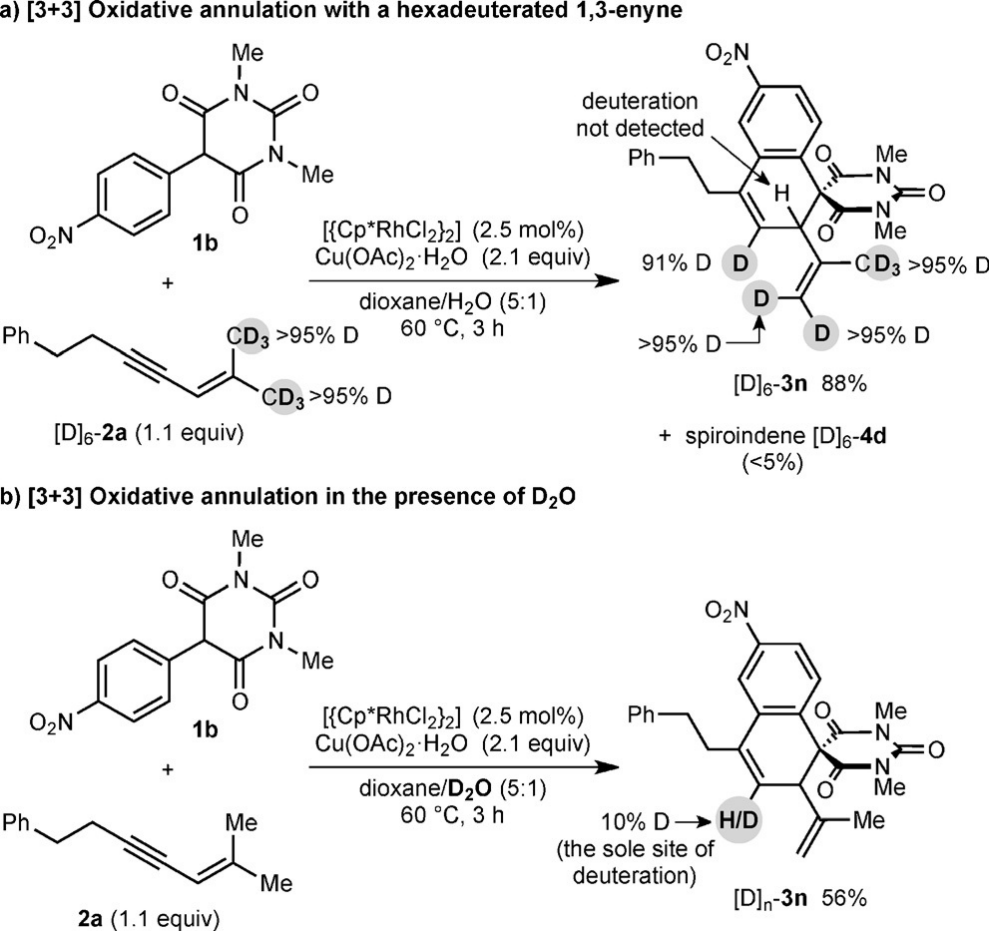
Oxidative annulation with a hexadeuterated 1,3-enyne.

Although the spirocyclic barbiturates prepared in this study are themselves of interest, they can be transformed into other compounds. For example, treatment of **3 o** with aqueous NaOH in THF gave the highly functionalized naphthalene **20** in 90 % yield [Eq. (5)].


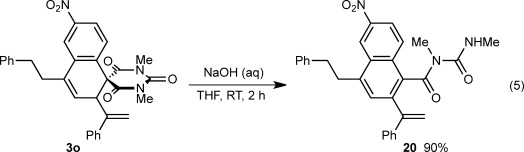
(5)

In conclusion, we have reported rhodium(III)-catalyzed, all-carbon [3+3] oxidative annulations of 5-arylbarbituric acids and related compounds with 1,3-enynes containing allylic hydrogens *cis* to the alkyne. This new mode of oxidative annulation further demonstrates the power of alkenyl-to-allyl 1,4-rhodium(III) migration in generating electrophilic allylrhodium species for the construction of polycyclic systems. Other applications of this method of allylmetal generation will be reported in due course.

## Supporting Information

As a service to our authors and readers, this journal provides supporting information supplied by the authors. Such materials are peer reviewed and may be re-organized for online delivery, but are not copy-edited or typeset. Technical support issues arising from supporting information (other than missing files) should be addressed to the authors.

miscellaneous_information
