# Supplementary material for: All-Carbon [3+3] Oxidative Annulations of 1,3-Enynes by Rhodium(III)-Catalyzed C–H Functionalization and 1,4-Migration
Source: Angew Chem Int Ed Engl. 2015 Jul 14;54(34):9958–62. doi: 10.1002/anie.201503978 (PMC4557058; doi:10.1002/anie.201503978)
Supplement: Supplementary file 1 — miscellaneous_information [file anie0054-9958-sd1.pdf]

Angewandte Chemie
Eine Zeitschrift der Gesellschaft Deutscher Chemiker

Supporting Information

All-Carbon [3+3] Oxidative Annulations of 1,3-Enynes by Rhodium(III)-Catalyzed C–H Functionalization and 1,4-Migration**

*David J. Burns, Daniel Best, Martin D. Wieczysty, and Hon Wai Lam**

anie_201503978_sm_miscellaneous_information.pdf

Supporting Information

Contents **Page**

General Information….. 2
Synthesis of Substrates... 3
[3+3] Oxidative Annulations of 1,3-Enynes... 15
Deuterium Labeling Experiments.. 30
Conversion of Spirodialin **3o** into Naphthalene **20**.. 33
NMR Spectra.. 34

1. General Information

Unless specified otherwise, all reactions were carried out under an atmosphere of nitrogen using oven-dried glassware. Unless specified otherwise, all commercially available reagents and solvents were used as received. Dioxane used for oxidative annulation reactions was non-anhydrous. THF was dried and purified by passage through activated alumina columns using a solvent purification system. All petroleum ether used was 40–60 °C petroleum ether. IMS stands for industrial methylated spirits. Thin layer chromatography (TLC) was performed on Merck DF-Alufoilien $60F_{254}$ 0.2 mm precoated plates. Compounds were visualized by exposure to UV light or by dipping the plates into solutions of potassium permanganate or vanillin followed by heating. Flash column chromatography was carried out using silica gel (Fisher Scientific 60 Å particle size 35–70 micron). Melting points were recorded on a Gallenkamp melting point apparatus and are uncorrected. The solvent of recrystallization is reported in parentheses. Infra-red spectra were recorded on a Nicolet Avatar 360 FT instrument on the neat compound using the attenuated total refraction technique. NMR spectra were acquired on Bruker DPX300, AV400, AV(III)400, or DPX400 spectrometers at room temperature unless otherwise stated. ^{1}H and ^{13}C NMR spectra were referenced to external tetramethylsilane *via* the residual protonated solvent (^{1}H) or the solvent itself (^{13}C). All chemical shifts are reported in parts per million (ppm). For $CDCl_3$, the shifts are referenced to 7.27 ppm for ^{1}H NMR spectroscopy and 77.0 ppm for ^{13}C NMR spectroscopy. For $(CD_3)_2CO$, the shifts are referenced to 2.05 ppm for ^{1}H NMR spectroscopy and 29.84 ppm for ^{13}C NMR spectroscopy. For $(CD_3)_2SO$, the shifts are referenced to 2.50 ppm for ^{1}H NMR spectroscopy and 39.52 ppm for ^{13}C NMR spectroscopy. All ^{19}F NMR spectra were not proton-decoupled. High-resolution mass spectra were recorded using electrospray ionization (ESI) techniques at the School of Chemistry, University of Nottingham.

2. Synthesis of Substrates

Preparation of α-Aryl Cyclic-1,3-Dicarbonyl Compounds

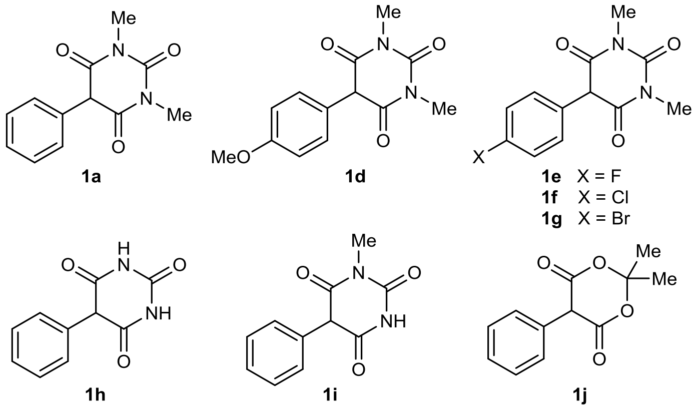

Substrates **1a**,[1] **1d-g**[2] **1h**,[3] **1i**,[4] and **1j**[1] were prepared according to literature procedures.

5-(4-Hydroxyphenyl)-1,3-dimethylbarbituric acid (S1)

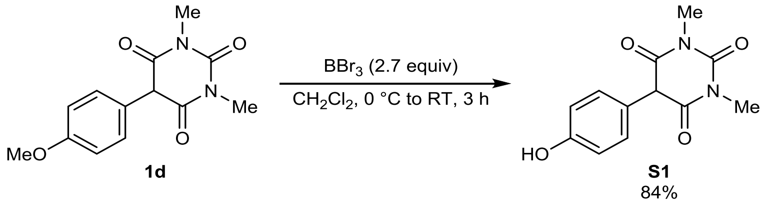

To a solution of 5-arylbarbituric acid **1d** (800 mg, 3.05 mmol) in CH_2Cl_2 (80 mL) at 0 °C was added BBr_3 (0.80 mL, 8.30 mmol) dropwise and the mixture was then stirred at room temperature for 3 h. Then, the mixture was cooled to 0 °C and quenched by the slow addition of H_2O (40 mL). The resulting precipitate was collected by vacuum filtration, washed with H_2O (50 mL) and CH_2Cl_2 (50 mL) and dried *in vacuo* to provide the *5-arylbarbituric acid* **S1** (639 mg, 84%) as an off-white crystalline solid. R_f 0.25 (40:40:20:1 PhMe/petroleum ether/acetone/AcOH on AcOH-doped SiO_2); m.p. 240−242 °C (CH_2Cl_2); IR 3315 (OH), 1685 (C=O), 1608 (C=O), 1586, 1519, 1191, 1121, 852, 753 cm^{-1}; ^{1}H NMR (300 MHz, $(CD_3)_2SO$) δ 9.50 (1H, s, O**H**), 7.09−7.02 (2H, m, 2 × Ar**H**), 6.74–6.68 (2H, m, 2 × Ar**H**), 4.79 (1H, s, C**H**), 3.15 (6H, s, 2 × NC**H**$_3$); ^{13}C NMR (75.5 MHz, $(CD_3)_2SO$) δ 168.1 (2 × C), 157.1 (C), 151.8 (C), 130.0 (2 × CH), 125.0 (C), 115.5 (2 × CH), 54.5 (CH), 28.3 (2 × CH_3); HRMS (ESI −ve) Exact mass calculated for $C_{12}H_{13}N_2O_4$ $[M{-}H]^-$: 276.0626, found

1. Prepared according to: Reddy Chidipudi, S.; Khan, I.; Lam, H. W. *Angew. Chem., Int. Ed.* **2012**, *51*, 12115−12119.
2. Prepared according to: Best, D.; Burns, D. J.; Lam, H. W. *Angew. Chem., Int. Ed.* **2015**, *54*, In Press, DOI: 10.1002anie.201502324.
3. Prepared according to: Dziewiszek, K.;; Schinazi, R. F.; Chou, T.; Su, T.; Dzik, J. M.; Rode, W.; Watanabe, K. A. *Nucleosides and Nucleotides* **1994** , *13*, 77−94.
4. Prepared according to: Bolz, I; Schaarschmidt, D.; Rüffer, T; Lang, H.; Spange, S. *Angew. Chem. Int. Ed.* **2009**, *48*, 7440–7443.

276.0619.

1,3-Dimethyl-5-(4-nitrophenyl)barbituric acid (1b)

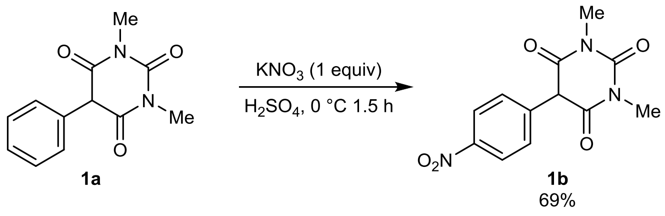

To a flask of vigorously stirring H_2SO_4 (10 mL) at room temperature was slowly added KNO_3 (252 mg, 2.50 mmol), taking care to avoid clumping. The mixture was stirred vigorously at room temperature for 15 min, after which it was almost homogeneous. The mixture was cooled to 0 °C and 1,3-dimethyl-5-phenylbarbituric acid (**1a**) (580 mg, 2.50 mmol) was added portionwise over 10 min. The mixture was stirred at 0 °C for 1.5 h and poured onto crushed ice (~50 g) and stirred vigorously until all the ice had melted. The precipitate was collected by vacuum filtration of the cold mixture and the filter cake was washed with cold H_2O (50 mL) and Et_2O (3 × 50 mL) to give *5-arylbarbituric acid* **1b** (476 mg, 69%) as a pale yellow crystalline solid. R_f 0−0.18 streak (40:40:20:1 PhMe/petroleum ether/acetone/AcOH on AcOH-doped SiO_2); m.p. 174−178 °C (IMS); IR 1689 (NO_2), 1674 (C=O), 1606 (C=O), 1515, 1446, 1421, 1377, 1289, 1275, 1110, 751, 734 cm^{-1}; ^1H NMR (300 MHz, $(CD_3)_2CO$) δ 8.27–8.20 (2H, m, 2 × Ar**H**), 7.70–7.64 (2H, m, 2 × Ar**H**), 5.28 (1H, s, C**H**), 3.26 (6H, s, 2 × NC**H**$_3$); ^{13}C NMR (75.5 MHz, $(CD_3)_2CO$) δ 167.5 (2 × C), 152.6 (C), 148.6 (C), 142.5 (C), 132.1 (2 × CH), 124.3 (2 × CH), 56.0 (CH), 29.0 (2 × CH_3); HRMS (ESI -ve) Exact mass calculated for $C_{12}H_{10}N_3O_5$ $[M−H]^-$: 276.0620, found 276.0635.

4-(1,3-Dimethyl-2,4,6-trioxo-1,3-diazinan-5-yl)phenyl acetate (1c)

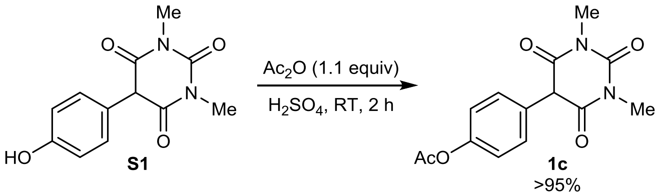

To a solution of phenol **S1** (200 mg, 0.81 mmol) in conc. H_2SO_4 (1 mL) was added acetic anhydride (83.7 µL, 80.0 mmol) and the mixture was stirred at room temperature for 2 h. The reaction was diluted with CH_2Cl_2 (10 mL), washed with brine (10 mL), and the aqueous layer was further extracted with CH_2Cl_2 (2 × 10 mL). The organic layers were combined, dried ($MgSO_4$), and concentrated *in vacuo* to give *acetate* **1c** (235 mg, >95%) as an off-white solid. R_f 0.25 (40:40:20:1 PhMe/petroleum ether/acetone/AcOH on AcOH-doped SiO_2); m.p. 98−100 °C (IMS/petroleum

ether) IR 1767 (C=O), 1661 (C=O), 1508, 1375, 1195, 1171, 908 cm^{-1}; ^{1}H NMR (300 MHz, $CDCl_3$) δ 7.29–7.18 (2H, m, 2 × Ar**H**), 7.17–7.06 (2H, m, 2 × Ar**H**), 4.68 (1H, s, C**H**), 3.36 (6H, s, 2 × NC**H**$_3$), 2.30 (3H, s, COC**H**$_3$); ^{13}C NMR (75.5 MHz, $CDCl_3$) δ 169.0 (C), 166.9 (2 × C), 151.4 (C), 150.8 (C), 130.8 (C), 129.0 (2 × CH), 122.4 (2 × CH), 54.8 (CH), 28.8 (2 × CH_3), 21.0 (CH_3); HRMS (ESI +ve) Exact mass calculated for $C_{14}H_{15}O_5$ [M+H]$^+$: 291.0975, found: 291.0992.

1,2-Dimethyl-4-phenylpyrazolidine-3,5-dione (7)

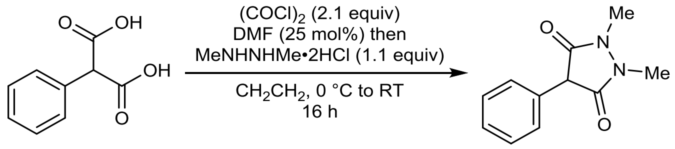

7
40%

To a solution of phenylmalonic acid (1.50 g, 8.33 mmol) and DMF (0.16 mL, 2.10 mmol) in CH_2Cl_2 (20 mL) at 0 °C was added $(COCl)_2$ (1.50 mL, 17.7 mmol) and the mixture was then stirred at room temperature for 1.5 h to give a crude malonyl dichloride solution. Meanwhile, in a separate vessel, Et_3N (9.20 mL, 66.0 mmol) was added to a suspension of MeNHNHMe·2HCl (1.22 g, 9.17 mmol) in CH_2Cl_2 (40 mL) at 0 °C with vigorous stirring and the mixture was then stirred at room temperature for 30 min. Then, the mixture was cooled to 0 °C before addition of the previously prepared crude malonyl dichloride solution *via* syringe and the combined mixture was stirred at room temperature for 16 h. Then, 3.0 M $HCl_{(aq)}$ (100 mL) was added and the layers were separated. The combined organic layers were washed with brine (100 mL), dried ($MgSO_4$) and concentrated *in vacuo*. Purification of the residue by flash column chromatography (20:1 to 2:1 acetone/PhMe with 1% AcOH on AcOH-doped SiO_2) gave *hydrazide* **7** (676 mg, 40%) as a white solid. R_f 0.10 (40:40:20:1 PhMe/petroleum ether/acetone/AcOH on AcOH-doped SiO_2); m.p. 141−143 °C (IMS/petroleum ether); IR 1767, 1669 (C=O), 1599, 1568, 1545, 1509, 1419, 1374, 1341, 1197, 1172, 995, 772 cm^{-1}; ^{1}H NMR (400 MHz, $CDCl_3$) δ 7.41–7.28 (5H, m, 5 × Ar**H**), 4.21 (1H, s, C**H**), 3.28 (6H, s, 2 × NC**H**$_3$); ^{13}C NMR (100.6 MHz, $CDCl_3$) δ 168.5 (2 × C), 131.4 (C), 129.0 (2 × CH), 128.4 (2 × CH), 128.2 (CH), 51.3 (CH), 30.2 (2 × CH_3); HRMS (ESI −ve) Exact mass calculated for $C_{11}H_{11}N_2O_2$ [M−H]$^-$: 203.0826, found 203.0824.

Preparation of 1,3-Enynes

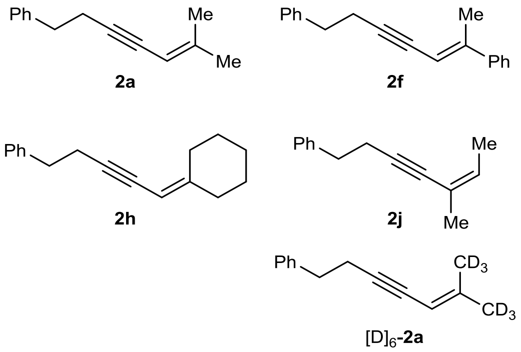

Substrates **2a**, **2f–g**, **2j**, **9**, and $[D]_6$-**2a** were prepared according to literature procedures.[5]

Methyl (*Z*)-3-iodoprop-2-enoate (S2)[6]

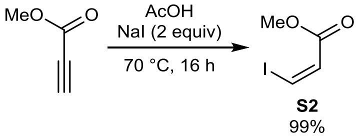

To a solution of methyl propiolate (3.56 mL, 40.0 mmol) in AcOH (20 mL) was added NaI (12.0 g, 80.0 mmol) and the mixture was stirred at 70 °C for 16 h. Then, the reaction was cooled to 0 °C, Et_2O (50 mL) and 1.0 M $NaOH_{(aq)}$ (100 mL) were added and the layers were separated. The aqueous layer was extracted with Et_2O (2 × 50 mL). The combined organic extracts were washed with saturated $Na_2S_2O_3$ (100 mL), dried ($MgSO_4$), and concentrated *in vacuo* to give vinyl iodide **S2** (8.40 g, 99%) as a yellow oil. Physical and spectral properties were in accordance with the literature.[6]

Methyl (*Z*)-non-2-en-4-ynoate (S3)[7]

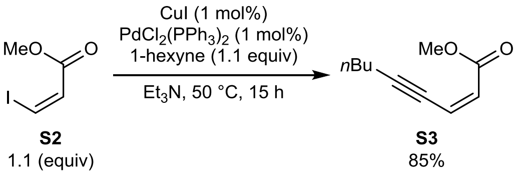

To a solution of vinyl iodide **S2** (8.69 g, 41.0 mmol), $PdCl_2(PPh_3)_2$ (288 mg, 0.41 mmol), and CuI (78.1 mg, 0.41 mmol) in Et_3N (160 mL) was added 1-hexyne (5.18 mL, 45.1 mmol) and the mixture was stirred at 50 °C for 15 h. The reaction was diluted with Et_2O (100 mL), washed with saturated $NH_4Cl_{(aq)}$ (100 mL), and the aqueous layer was further extracted with Et_2O (2 × 100 mL). The organic layers were combined, dried ($MgSO_4$), and concentrated *in vacuo*. Purification of the residue by flash column chromatography (50:1 Et_2O:petroleum ether) gave 1,3-enyne **S3** (6.35 g,

5. Burns, D. J.; Lam, H. W. *Angew. Chem., Int. Ed.* **2014**, *53*, 9931–9935.
6. Rezaei, H.; Dupont-Gaudet, K.; Bélanger, F.; Spino, C. *J. Am. Chem. Soc.* **2004**, *126*, 9926–9927.

85%) as a yellow oil. Physical and spectral properties were in accordance with the literature.[7]

(*Z*)-Non-2-en-4-yn-1-ol (S4)[8]

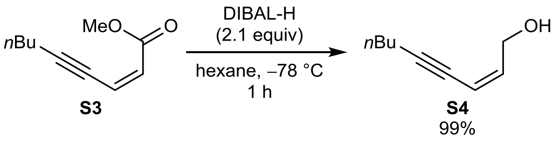

To DIBAL-H (21.2 mL of a 1.0 M solution in hexane, 21.2 mmol) at −78 °C was added dropwise 1,3-enyne **S3** (1.68 g, 10.1 mmol) and the mixture was stirred at −78 °C for 1 h. The reaction was warmed to 0 °C and 1.0 M $HCl_{(aq)}$ was added dropwise until effervescence ceased. Then, Et_2O (50 mL) and 1.0 M $HCl_{(aq)}$ (50 mL) were added, the layers were separated, and the aqueous layer was further extracted with Et_2O (2 × 50 mL). The organic layers were combined, dried ($MgSO_4$), and concentrated *in vacuo*. Purification of the residue by flash column chromatography (3:2 petroleum ether:Et_2O) gave alcohol **S4** (1.39 g, 99%) as a colorless oil. Physical and spectral properties were in accordance with the literature.[8]

[{(*Z*)-Non-2-en-4-yn-1-yloxy}methyl]benzene (2b)

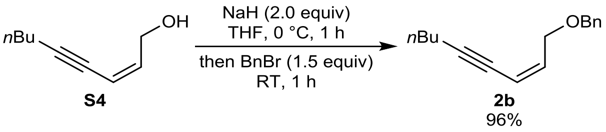

To a stirred solution of alcohol **S4** (5.00 g, 36.2 mmol) in THF (200 mL) at 0 °C was added NaH (2.78 g of 60% wt in mineral oil, 72.4 mmol) and the mixture was stirred at 0 °C for 1 h. Then, benzyl bromide (6.46 mL, 54.3 mmol) was added, the reaction was warmed to room temperature and stirred for a further 1 h. After this time 1.0 M $HCl_{(aq)}$ (200 mL) was added, the layers were separated, and the aqueous layer was further extracted with Et_2O (2 × 100 mL). The organic layers were combined, dried ($MgSO_4$), and concentrated *in vacuo*. Purification of the residue by flash column chromatography (30:1 petroleum ether:Et_2O) gave *1,3-enyne* **2b** (7.97 g, 96%) as a colorless oil. R_f 0.39 (10:1 petroleum ether:Et_2O); IR 1096, 732, 696, 626 cm^{-1}; ^{1}H NMR (400 MHz, $CDCl_3$) δ 7.41–7.27 (5H, m, 5 × Ar**H**), 6.01 (1H, dt, *J* = 10.8, 6.5 Hz, CH=C**H**CH_2OBn), 5.70–5.62 (1H, m, C**H**=CHCH_2OBn), 4.55 (2H, s, OC**H**$_2$Ph), 4.31 (2H, dd, *J* = 6.5, 1.3 Hz, C**H**$_2$OBn), 2.34 (2H, td, *J* = 6.9, 2.0 Hz, C**H**$_2CH_2CH_2CH_3$), 1.57–1.47 (2H, m, C**H**$_2CH_2CH_3$), 1.47–1.37 (2H, m, C**H**$_2CH_3$), 0.93 (3H, t, *J* = 7.2 Hz, C**H**$_3$); ^{13}C NMR (100.6 MHz, $CDCl_3$) δ 138.3 (C), 137.7 (CH), 128.3 (2 × CH), 127.8 (2 × CH), 127.6 (CH), 112.3 (CH), 96.6 (C), 76.4 (C), 72.2

7. Tuanli Yao, T.; Larock, R. C. *J. Org. Chem.* **2003**, *68*, 5936–5942.
8. Lauria, E.; Salerno, G.; Gabriele, B. *J. Org. Chem.* **1999**, *64*, 7687–7692.

(CH_2), 67.8 (CH_2), 30.7 (CH_2), 21.9 (CH_2), 19.2 (CH_2), 13.6 (CH_3); HRMS (ESI +ve) Exact mass calculated for $C_{16}H_{20}NaO$ $[M+Na]^+$: 251.1406, found: 251.1419.

(*Z*)-8-[(*tert*-Butyldimethylsilyl)oxy]oct-3-en-5-yn-2-one (S5)

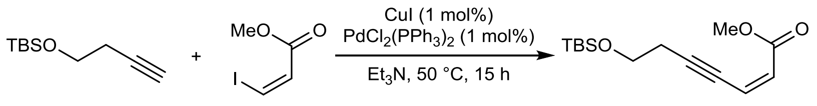

S2
1.1 (equiv)

S5
74%

To a solution of vinyl iodide **S2** (3.73 g, 17.6 mmol), $PdCl_2(PPh_3)_2$ (124 mg, 0.18 mmol), and CuI (33.5 mg, 0.18 mmol) in Et_3N (50 mL) was added 4-(*tert*-butyldimethylsilyloxy)-1-butyne (4.00 mL, 19.4 mmol) and the mixture was stirred at 50 °C for 15 h. The reaction was diluted with Et_2O (100 mL), washed with saturated $NH_4Cl_{(aq)}$ (100 mL), and the aqueous layer was further extracted with Et_2O (2 × 100 mL). The organic layers were combined, dried ($MgSO_4$), and concentrated *in vacuo*. Purification of the residue by flash column chromatography (10:1 Et_2O:petroleum ether) gave *1,3-enyne* **S5** (3.51 g, 74%) as a yellow oil. R_f 0.19 (10:1 petroleum ether:Et_2O); IR 1732 (C=O), 1193, 1174, 1101, 836, 777, 652 cm^{-1}; ^1H NMR (400 MHz, $CDCl_3$) δ 6.14 (1H, dt, J = 11.4, 2.4 Hz, C**H**=CHCO), 6.05 (1H, d, J = 11.4 Hz, CH=C**H**CO), 3.80 (2H, t, J = 7.2 Hz, TBSOC**H**$_2$CH$_2$), 3.75 (3H, s, OC**H**$_3$), 2.67 (2H, td, J = 7.2, 2.4 Hz, TBSOCH$_2$C**H**$_2$), 0.89 (9H, s, C(C**H**$_3$)$_3$), 0.07 (6H, s, 2 × SiC**H**$_3$); ^{13}C NMR (100.6 MHz, $CDCl_3$) δ 165.2 (C), 127.3 (CH), 123.9 (CH), 100.8 (C), 78.5 (C), 61.5 (CH_2), 51.3 (CH_3), 25.8 (3 × CH_3), 24.4 (CH_2), 18.3 (C), −5.3 (2 × CH_3); HRMS (ESI +ve) Exact mass calculated for $C_{14}H_{25}O_3Si$ $[M+H]^+$: 269.1567, found: 268.1564.

(*Z*)-7-[(*tert*-Butyldimethylsilyl)oxy]hept-2-en-4-yn-1-ol (S6)

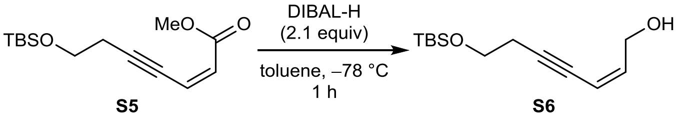

S5

S6
96%

To DIBAL-H (17.2 mL of a 1.5 M solution in toluene, 26.5 mmol) at −78 °C was added dropwise 1,3-enyne **S5** (3.39 g, 12.6 mmol) and the mixture was stirred at −78 °C for 1 h. The reaction was warmed to 0 °C and 1.0 M $HCl_{(aq)}$ was added dropwise until effervescence ceased. Then, Et_2O (50 mL) and 1.0 M $HCl_{(aq)}$ (50 mL) were added, the layers were separated, and the aqueous layer was further extracted with Et_2O (2 × 50 mL). The organic layers were combined, dried ($MgSO_4$), and concentrated *in vacuo*. Purification of the residue by flash column chromatography (3:2 petroleum ether:Et_2O) gave *alcohol* **S6** (2.91 g, 96%) as a colorless oil. R_f 0.27 (2:1 petroleum ether:Et_2O); IR 3339 (OH), 1256, 1102, 835, 776, 648 cm^{-1}; ^1H NMR (400 MHz, $CDCl_3$) δ 6.03 (1H, dt, J = 10.8, 6.4 Hz, CH=C**H**CH$_2$OH), 5.62–5.55 (1H, m, C**H**=CHCH$_2$OH), 4.39 (2H, dd, J = 6.4, 1.2 Hz,

C**H**$_2$OH), 3.76 (2H, t, *J* = 7.0 Hz, TBSOC**H**$_2$CH$_2$), 2.56 (2H, td, *J* = 7.0, 2.1 Hz, TBSOCH$_2$C**H**$_2$), 0.91 (9H, s, C(C**H**$_3$)$_3$), 0.09 (6H, s, 2 × SiC**H**$_3$); ^{13}C NMR (100.6 MHz, CDCl$_3$) δ 140.3 (CH), 111.1 (CH), 93.5 (C), 77.2 (C), 61.8 (CH$_2$), 61.0 (CH$_2$), 25.9 (3 × CH$_3$), 23.9 (CH$_2$), 18.3 (C), −5.3 (2 × CH$_3$); HRMS (ESI +ve) Exact mass calculated for $C_{13}H_{24}NaO_2Si$ [M+Na]$^+$: 263.1438, found: 263.1447.

(*Z*)-[((7-Benzyloxy)hept-5-en-3-yn-1-yl)oxy](*tert*-butyl)dimethylsilane (2c)

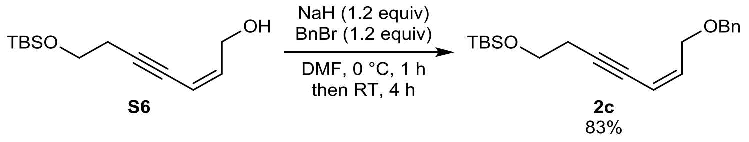

To a stirred solution of alcohol **S6** (2.91 g, 12.1 mmol) and benzyl bromide (1.72 mL, 14.5 mmol) in DMF (30 mL) at 0 °C was added NaH (568 mg of 60% wt in mineral oil, 14.5 mmol) and the mixture was stirred at 0 °C for 1 h. Then, the reaction was warmed to room temperature and stirred for a further 4 h. After this time, 1.0 M HCl$_{(aq)}$ (100 mL) was added, the layers were separated, and the aqueous layer was further extracted with Et$_2$O (2 × 50 mL). The organic layers were combined, dried (MgSO$_4$), and concentrated *in vacuo*. Purification of the residue by flash column chromatography (30:1 petroleum ether:Et$_2$O) gave *1,3-enyne* **2c** (3.33 g, 83%) as a colorless oil. R$_f$ 0.40 (5% Et$_2$O/petroleum ether); IR 2929, 2855, 1472, 1255, 1100, 1006, 912, 835, 776 cm^{-1}; ^{1}H NMR (300 MHz, CDCl$_3$) δ 7.42–7.26 (5H, m, 5 × Ar**H**), 6.04 (1H, dt, *J* = 10.9, 6.4 Hz, HC=C**H**CH$_2$OBn), 5.66 (1H, dtt, *J* = 10.9, 2.2, 1.4 Hz, C**H**=CHCH$_2$OBn), 4.55 (2H, s, OC**H**$_2$Ph), 4.32 (2H, dd, *J* = 6.4, 1.4 Hz, HC=CHC**H**$_2$OBn), 3.75 (2H, t, *J* = 7.1 Hz, TBSOC**H**$_2$), 2.56 (2H, td, *J* = 7.1, 2.2 Hz, TBSOCH$_2$C**H**$_2$), 0.94 (9H, s, C(C**H**$_3$)$_3$), 0.11 (6H, s, 2 × SiC**H**$_3$); ^{13}C NMR (75.5 MHz, CDCl$_3$) δ 138.3 (CH), 138.2 (C), 128.3 (2 × CH), 127.8 (2 × CH), 127.6 (CH), 112.0 (CH), 93.2 (C), 77.4 (C), 72.2 (CH$_2$), 67.8 (CH$_2$), 61.8 (CH$_2$), 25.8 (3 × CH$_3$), 23.9 (CH$_2$), 18.3 (C), −5.3 (2 × CH$_3$); HRMS (ESI +ve) Exact mass calculated for $C_{20}H_{30}NaO_2Si$ [M+Na]$^+$: 353.1907, found 353.1915.

(*Z*)-7-(Benzyloxy)hept-5-en-3-yn-1-ol (2d)

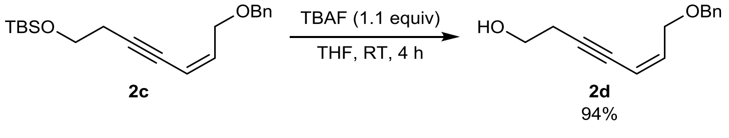

To a stirred solution of 1,3-enyne **2c** (1.50 g, 4.54 mmol) in THF (10 mL) at room temperature was added TBAF (5.00 mL of a 1.0 M solution in THF, 5.00 mmol) and the mixture was stirred for 4 h. Then, Et$_2$O (50 mL) and 1.0 M HCl$_{(aq)}$ (50 mL) were added, the layers were separated, and the

aqueous layer was further extracted with Et_2O (2 × 50 mL). The organic layers were combined, dried ($MgSO_4$), and concentrated *in vacuo*. Purification of the residue by flash column chromatography (95:5 to 13:7 petroleum ether:acetone) gave *alcohol* **2d** (925 mg, 94%) as a colorless oil. R_f 0.23 (1:1 petroleum ether:Et_2O); IR 3391 (OH), 1050, 736, 697, 646 cm^{-1}; ^1H NMR (300 MHz, $CDCl_3$) δ 7.42–7.26 (5H, m, 5 × Ar**H**), 6.05 (1H, dt, *J* = 10.9, 6.5 Hz, CH=C**H**CH_2OBn), 5.71–5.61 (1H, m, C**H**=CHCH_2OBn), 4.54 (2H, s, OC**H**$_2$Ph), 4.29 (2H, dd, *J* = 6.5, 1.3 Hz, C**H**$_2$OBn), 3.69 (2H, t, *J* = 6.4 Hz, HOC**H**$_2$$CH_2$), 2.63 (1H, br s, O**H**), 2.57 (2H, td, *J* = 6.4, 2.1 Hz, HOCH_2C**H**$_2$); ^{13}C NMR (75.5 MHz, $CDCl_3$) δ 138.4 (CH), 138.0 (C), 128.3 (2 × CH), 127.8 (2 × CH), 127.6 (CH), 111.9 (CH), 92.7 (C), 77.9 (C), 72.1 (CH_2), 67.6 (CH_2), 60.9 (CH_2), 23.7 (CH_2); HRMS (ESI +ve) Exact mass calculated for $[M+H]^+$: $C_{14}H_{17}O_2$, 217.1223, found: 217.1245.

(*Z*)-1-Methoxynon-2-en-4-yne (2e)

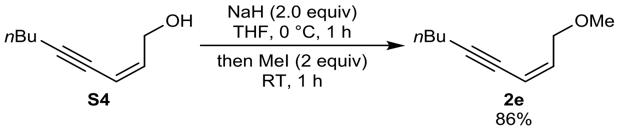

To a stirred solution of alcohol **S4** (1.69 g, 12.2 mmol) in THF (100 mL) at 0 °C was added NaH (978 mg of 60% wt in mineral oil, 24.5 mmol) and the mixture was stirred at 0 °C for 1 h. Then, methyl iodide (1.52 mL, 24.5 mmol) was added, the reaction was warmed to room temperature and stirred for a further 1 h. After this time, 1.0 M $HCl_{(aq)}$ (100 mL) was added, the layers were separated, and the aqueous layer was further extracted with Et_2O (2 × 50 mL). The organic layers were combined, dried ($MgSO_4$), and concentrated *in vacuo*. Purification of the residue by flash column chromatography (2:1 petroleum ether:Et_2O) gave *1,3-enyne* **2e** (1.60 g, 86%) as a colorless oil. R_f 0.33 (10:1 petroleum ether:Et_2O); IR 1101, 729, 641 cm^{-1}; ^1H NMR (400 MHz, $CDCl_3$) δ 5.92 (1H, dt, *J* = 10.9, 6.4 Hz, CH=C**H**CH_2OMe), 5.66–5.60 (1H, m, C**H**=CHCH_2OMe), 4.18 (2H, dd, *J* = 6.4, 1.4 Hz, C**H**$_2$OMe), 3.35 (3H, s, OC**H**$_3$), 2.35 (2H, td, *J* = 6.9, 2.1 Hz, C**H**$_2$$CH_2$$CH_2$$CH_3$), 1.58–1.49 (2H, m, C**H**$_2$$CH_2$$CH_3$), 1.49–1.37 (2H, m, C**H**$_2$$CH_3$), 0.93 (3H, t, *J* = 7.3 Hz, CH_2C**H**$_3$); ^{13}C NMR (100.6 MHz, $CDCl_3$) δ 137.6 (CH), 112.3 (CH), 96.5 (C), 76.3 (C), 70.1 (CH_2), 58.0 (CH_3), 30.8 (CH_2), 21.9 (CH_2), 19.2 (CH_2), 13.6 (CH_3).

6-(Bromomethylene)-2,2,3,3,9,9,10,10-octamethyl-4,8-dioxa-3,9-disilaundecane (S7)[9]

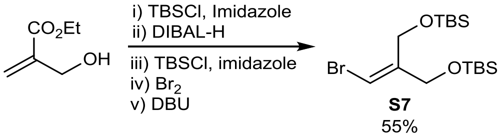

The title vinyl bromide was prepared by a slightly modified literature procedure, spectroscopic data was in accordance with the literature.[9] To a solution of ethyl 2-(hydroxymethyl)acrylate[10] (8.60 g, 66.2 mmol) and imidazole (5.83 g, 85.8 mmol) in CH_2Cl_2 (100 mL) at 0 °C was added TBSCl (12.0 g, 79.6 mmol) in 3 × 4.0 g portions at 5 min intervals. Then, the mixture was warmed to room temperature and stirred for a further 1 h. After this, time Et_2O (200 mL) and 1.0 M $HCl_{(aq)}$ (100 mL) were added, the layers were separated, and the organic layer was further extracted with 1.0 M $HCl_{(aq)}$ (100 mL) and brine (3 × 50 mL). The organic layer was dried ($MgSO_4$) and concentrated *in vacuo* to provide the crude silyl ether (16.8 g, >95%). This material was dissolved in CH_2Cl_2 (100 mL) and cooled to −78 °C before slow addition of DIBAL-H (92.0 mL of a 1.5 M solution in toluene, 138 mmol). The mixture was stirred at −78 °C for a further 30 min, diluted with EtOAc (100 mL) and stirred vigorously with saturated potassium sodium tartrate$_{(aq)}$ (200 mL) at room temperature for 15 h. After this time, the layers were separated, and the organic layer was further extracted with brine (2 × 100 mL), dried ($MgSO_4$), and concentrated *in vacuo* to provide the crude alcohol (14.1 g, >95%). This material was dissolved in CH_2Cl_2 (100 mL) with imidazole (5.83 g, 85.8 mmol) and cooled to 0 °C. TBSCl (12.0 g, 79.6 mmol) was added in 3 × 4.0 g portions at 5 min intervals. The mixture was stirred at room temperature for 1 h. Then Et_2O (200 mL) and 1.0 M $HCl_{(aq)}$ (100 mL) were added, the layers were separated, and the organic layer was further extracted with 1.0 M $HCl_{(aq)}$ (2 × 100 mL), dried ($MgSO_4$), and concentrated *in vacuo* to provide the crude bis(silyl ether) (20.1 g, >95%). This material was dissolved in CH_2Cl_2 (100 mL) and cooled to −78 °C before slow addition of bromine (3.28 mL, 64.0 mmol). The mixture was stirred at −78 °C for a further 10 min, quenched with 2% $Na_2SO_{3(aq)}$ (100 mL) and warmed to room temperature. The layers were separated and the aqueous layer was further extracted with CH_2Cl_2 (2 × 50 mL). The organic layers were combined, dried ($MgSO_4$), and concentrated *in vacuo*. The crude residue was filtered through a short plug of silica gel, eluting with Et_2O, to provide the pure dibromide (22.4 g, 71% over 4 steps) as a colorless oil. This material was heated at reflux in toluene (100 mL) with DBU (28.4 mL, 190 mmol) for 3 h. Then, the mixture was cooled to room temperature and 2.0 M $HCl_{(aq)}$ (75 mL) was added, the layers were separated, and the aqueous layer was further extracted

9. Prepared according to: Danishefsky, S. J.; Mantlo, N. *J. Am. Chem. Soc.* **1988**, *110*, 8129−8133.
10. Prepared according to: Gravel, D.; Ratemiel, A. S.; Hatam, M.; Boileau, G.; Crine, P.; Lemire, I. World Patent 2004 WO2004050620 A2.

with brine (2 × 100 mL), the organic layers were combined, washed with saturated $NaHCO_{3(aq)}$ (100 mL), dried ($MgSO_4$), and concentrated *in vacuo* to provide the vinyl bromide **S7** (14.5 g, 78%, or 55% over 5 steps), which was used without further purification.

2-(Bromomethylene)propane-1,3-diol (S8)

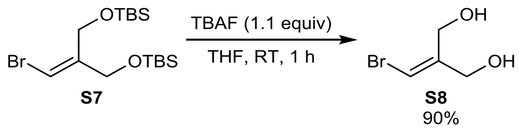

To a solution of the bis(silyl ether) **S7** (7.91 g, 20.0 mmol) in THF (60 mL) at 0 °C was added TBAF (40 mL of a 1.0 M solution in THF, 40.0 mmol) and the reaction mixture was stirred at room temperature for 1 h. Then, the mixture was preadsorbed onto silica gel and purified by flash column chromatography (10:1 to 1:1 acetone/petroleum ether) to give *diol* **S8** (2.99 g, 90%) as a colorless oil. R_f 0.60 (1:1 petroleum ether:acetone); IR 3320 (OH), 1630, 1457, 1362, 1299, 1228, 1136, 1010, 842, 852 cm^{-1}; ^1H NMR (300 MHz, $CDCl_3$) δ 6.35 (1H, p, *J* = 1.0 Hz, C=C**H**Br), 4.47 (2H, d, *J* = 1.0 Hz, C**H**$_2$OH), 4.30 (2H, d, *J* = 1.0 Hz, C**H**$_2$OH), 2.15 (2H, br s, 2 × O**H**); ^{13}C NMR (75.5 MHz, $CDCl_3$) δ 142.8 (C), 106.4 (CH), 64.6 (CH_2), 61.9 (CH_2); HRMS (ESI +ve) Exact mass calculated for $C_4H_7BrNaO_2$ $[M+Na]^+$: 188.9522, found 188.9525.

5-(Bromomethylene)-2,2-dimethyl-1,3-dioxane (S9)[11]

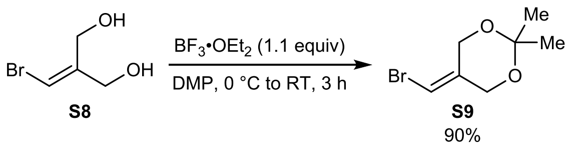

To a solution of diol **S8** (1.25 g, 7.49 mmol) in 2,2-dimethoxypropane (10 mL) at 0 °C was added $BF_3 \cdot OEt_2$ (1.0 mL, 8.1 mmol) and the reaction mixture was stirred at 0 °C for 2 h and room temperature for 1 h. Then the mixture was preadsorbed on silica gel and purified by flash column chromatography (10:1 to 0:1 petroleum ether:CH_2Cl_2) to give vinyl bromide **S9** (1.50 g, >95%) as a pale brown oil. Spectroscopic data were in accordance with the literature.[11]

11. Prepared according to: Riehs, G.; Urban, E.; Völlenkle, H.; Tetrahedron, **1996**, *52*, 8725−8732.

5-(Hept-2-yn-1-ylidene)-2,2-dimethyl-1,3-dioxane (2i)

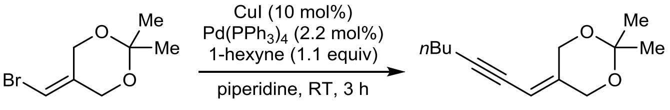

To a solution of vinyl bromide **S9** (1.04 g, 5.00 mmol), $Pd(PPh_3)_4$ (127 mg, 0.110 mmol), and CuI (95 mg, 0.50 mmol) in piperidine (10 mL) was added 1-hexyne (0.63 mL, 5.5 mmol) and the mixture was stirred at room temperature for 3 h. The reaction was diluted with Et_2O (100 mL), washed with saturated NH_4Cl (100 mL), and the aqueous layer was further extracted with Et_2O (2 × 100 mL). The organic layers were combined, dried ($MgSO_4$), and concentrated *in vacuo*. Purification of the residue by flash column chromatography (50:1 petroleum ether:Et_2O) gave *1,3-enyne* **2i** (935 mg, 90%) as a pale yellow oil. R_f 0.35 (90:1 petroleum ether:Et_2O); IR 2958, 1381, 1370, 1241, 1221, 1203, 1152, 1084, 835 cm^{-1}; ^{1}H NMR (300 MHz, $CDCl_3$) δ 5.27 (1H, sept, *J* = 1.8 Hz, C**H**=), 4.50 (2H, s, OC$\mathbf{H_2}$), 4.27 (2H, s, OC$\mathbf{H_2}$), 2.30 (2H, td, *J* = 6.7, 1.8 Hz, C$\mathbf{H_2}$$CH_2CH_2CH_3$), 1.55–1.32 (4H, m, C$\mathbf{H_2}C\mathbf{H_2}$$CH_3$), 1.40 (6H, s, C(C$\mathbf{H_3}$)$_2$), 0.89 (3H, t, *J* = 7.2 Hz, CH_2C$\mathbf{H_3}$); ^{13}C NMR (75.5 MHz, $CDCl_3$) δ 144.6 (C), 103.0 (CH), 99.0 (C), 96.1 (C), 75.4 (C), 63.2 (CH_2), 61.3 (CH_2), 30.7 (CH_2), 23.8 (2 × CH_3), 21.8 (CH_2), 19.1 (CH_2), 13.5 (CH_3); HRMS (ESI +ve) Exact mass calculated for $C_{13}H_{20}NaO_2$ $[M+Na]^+$: 231.1356, found 231.1355.

(*Z*)-Non-2-en-4-yn-1-yl acetate (2k)

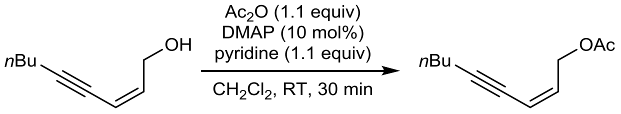

To a stirred solution of alcohol **S4** (1.39 g, 10.1 mmol) in CH_2Cl_2 (10 mL) at room temperature was added DMAP (123 mg, 1.00 mmol), pyridine (896 µL, 11.1 mmol), and acetic anhydride (1.05 mL, 11.1 mmol) and the mixture was stirred for 30 min. Then, Et_2O (50 mL) and 1.0 M $HCl_{(aq)}$ (50 mL) were added, the layers were separated, and the aqueous layer was further extracted with Et_2O (2 × 50 mL). The organic layers were combined, dried ($MgSO_4$), and concentrated *in vacuo*. Purification of the residue by flash column chromatography (2:1 petroleum ether:Et_2O) gave *1,3-enyne* **2k** (1.22 g, 64%) as a colorless oil. R_f 0.31 (10:1 petroleum ether:Et_2O); IR 1741 (C=O), 1370, 1231, 1028, 645 cm^{-1}; ^{1}H NMR (400 MHz, $CDCl_3$) δ 5.91 (1H, dt, *J* = 10.8, 6.6 Hz, CH=C**H**CH_2OAc), 5.71–5.63 (1H, m, C**H**=CHCH_2OAc), 4.82 (2H, dd, *J* = 6.6, 1.1 Hz, C$\mathbf{H_2}$OAc), 2.35 (2H, td, *J* = 7.0, 2.1 Hz, C$\mathbf{H_2}$$CH_2CH_2CH_3$), 2.08 (3H, s, COC$\mathbf{H_3}$), 1.57–1.49 (2H, m, C$\mathbf{H_2}$$CH_2CH_3$), 1.48–1.37 (2H, m, C$\mathbf{H_2}$$CH_3$), 0.93 (3H, t, *J* = 7.3 Hz, CH_2C$\mathbf{H_3}$); ^{13}C NMR (100.6 MHz, $CDCl_3$) δ 170.9 (C), 134.6

(CH), 113.6 (CH), 97.6 (C), 75.8 (C), 62.4 (CH_2), 30.7 (CH_2), 22.0 (CH_3), 20.9 (CH_2), 19.2 (CH_2), 13.6 (CH_3); HRMS (ESI +ve) Exact mass calculated for $C_{11}H_{16}NaO_2$ $[M+Na]^+$: 203.1043, found: 203.1059.

3. [3+3] Oxidative Annulations of 1,3-Enynes

General Procedure A

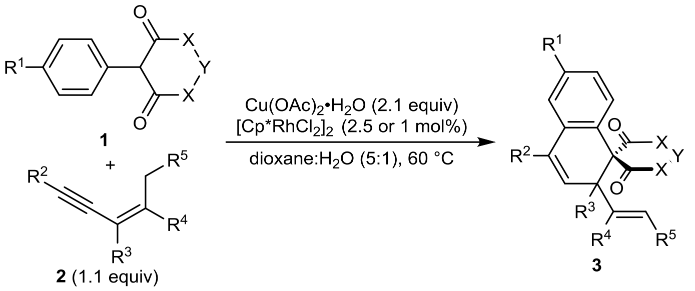

To a microwave vial, was added the appropriate α-aryl cyclic-1,3-dicarbonyl compound (0.50 mmol), $[Cp^*RhCl_2]_2$ (7.7 mg, 13 μmol), $Cu(OAc)_2{\cdot}H_2O$ (210 mg, 1.05 mmol), and the appropriate 1,3-enyne (0.55 mmol). The vessel was then sealed, flushed with N_2, and 1,4-dioxane:H_2O mixture (5:1, 5 mL) was added. The reaction was then heated at the indicated temperature for the specified time. The reaction was cooled to room temperature, filtered through a short pad of silica using Et_2O (25 mL) as eluent, and concentrated *in vacuo*. Purification of the residue by flash column chromatography or trituration with hot EtOH gave the title compound(s).

1,5-Dimethyl-4'-(2-phenylethyl)-2'-(prop-1-en-2-yl)-2'*H*-spiro[1,5-diazinane-3,1'-naphthalene]-2,4,6-trione (3a) and 1,5-Dimethyl-2'-(2-methylprop-1-en-1-yl)-3'-(2-phenylethyl)spiro[1,5-diazinane-3,1'-indene]-2,4,6-trione (4a)

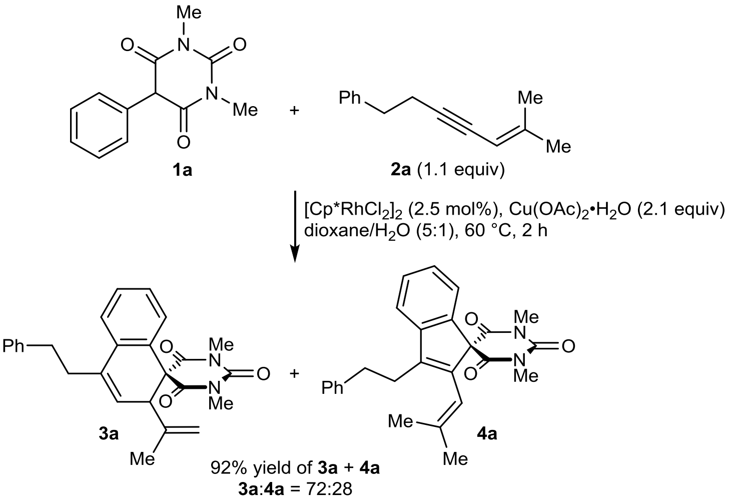

General Procedure A was followed using 5-arylbarbituric acid **1a** (108 mg, 0.50 mmol) and 1,3-enyne **2a** (101 mg, 0.55 mmol) at 60 °C for a reaction time of 2 h. Purification by flash column chromatography (2:1 to 1:1 petroleum ether:Et_2O) gave a 72:28 mixture (by ^{1}H NMR spectroscopy) of *spirodialin* **3a** and *spiroindene* **4a** respectively as a yellow oil (190 mg, 92%). Subsequent flash column chromatography (1:0 to 10:1 petroleum ether:EtOAc) gave sufficiently pure fractions of

spirodialin **3a** and *spiroindene* **4a** to allow individual characterization.

Data for spirodialin 3a:

R_f 0.37 (5:1 petroleum ether:EtOAc); m.p. 98–100 °C (EtOAc/hexane); IR 2960, 1673 (C=O), 1419, 1370, 1274, 1093, 1048, 921, 745, 699 cm^{-1}; ^{1}H NMR (400 MHz, $CDCl_3$) δ 7.48 (1H, dd, *J* = 7.8, 0.8 Hz, Ar**H**), 7.37–7.28 (5H, m, 5 × Ar**H**), 7.25–7.17 (2H, m, 2 × Ar**H**), 6.87 (1H, dd, *J* = 7.7, 1.1 Hz, Ar**H**), 5.56 (1H, d, *J* = 3.0 Hz, =C**H**CH), 4.98–4.95 (1H, m, C$\mathbf{H_A}$$H_B$=), 4.86 (1H, s, C$H_A$$\mathbf{H_B}$=), 4.17 (1H, d, *J* = 3.0 Hz, =CHC**H**), 3.43 (3H, s, NC$\mathbf{H}_3$), 3.25 (3H, s, NC$\mathbf{H}_3$), 3.01–2.75 (4H, m, C$\mathbf{H}_2$C$\mathbf{H}_2$), 1.64 (3H, s, =CC$\mathbf{H}_3$); ^{13}C NMR (100.6 MHz, $CDCl_3$) δ 172.6 (C), 167.7 (C), 151.1 (C), 143.2 (C), 142.0 (C), 135.9 (C), 134.8 (C), 132.9 (C), 129.0 (CH), 128.4 (2 × CH), 128.36 (2 × CH), 127.8 (CH), 127.2 (CH), 125.9 (CH), 124.1 (CH), 122.4 (CH), 116.7 (CH_2), 60.7 (C), 53.1 (CH), 34.6 (CH_2), 34.5 (CH_2), 29.0 (CH_3), 28.7 (CH_3), 21.1 (CH_3); HRMS (ESI +ve) Exact mass calculated for $C_{26}H_{27}N_2O_3$ $[M+H]^+$: 415.2016, found: 415.2008.

Data for spiroindene 4a:

R_f 0.33 (5:1 petroleum ether:EtOAc); m.p. 150–152 °C (EtOAc/hexane); IR 2972, 1680 (C=O), 1438, 1366, 1258, 1118, 1066, 822, 752, 736 cm^{-1}; ^{1}H NMR (400 MHz, $CDCl_3$) δ 7.42–7.37 (2H, m, 2 × Ar**H**), 7.30 (2H, dd, *J* = 9.6, 5.5 Hz, 2 × Ar**H**), 7.25–7.19 (4H, m, 4 × Ar**H**), 7.17 (1H, d, *J* = 7.5 Hz, Ar**H**), 5.42 (1H, s, =C**H**), 3.37 (6H, s, 2 × NC$\mathbf{H}_3$), 2.93 (2H, dd, *J* = 9.7, 6.4 Hz, C$\mathbf{H}_2$), 2.80 (2H, dd, *J* = 9.7, 6.4 Hz, C$\mathbf{H}_2$), 1.81 (3H, d, *J* = 1.1 Hz, =CC$\mathbf{H}_3$), 1.69 (3H, s, =CC$\mathbf{H}_3$); ^{13}C NMR (100.6 MHz, $CDCl_3$) δ 166.8 (2 × C), 151.7 (C), 145.6 (C), 143.9 (C), 142.2 (C), 141.5 (C), 141.1 (C), 139.0 (C), 129.2 (CH), 128.5 (2 × CH), 128.3 (2 × CH), 126.2 (CH), 126.0 (CH), 121.4 (CH), 120.8 (CH), 115.5 (CH), 69.6 (C), 34.0 (CH_2), 29.2 (2 × CH_3), 28.4 (CH_2), 25.9 (CH_3), 20.1 (CH_3); HRMS (ESI +ve) Exact mass calculated for $C_{26}H_{27}N_2O_3$ $[M+H]^+$: 415.2016, found: 415.2006.

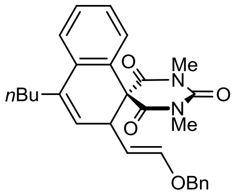

2'-[(*E*)-2-(Benzyloxy)ethenyl]-4'-butyl-1,5-dimethyl-2'*H*-spiro[1,5-diazinane-3,1'-naphthalene]-2,4,6-trione (3b). The title compound was prepared according to General Procedure A from 5-arylbarbituric acid **1a** (116 mg, 0.50 mmol) and 1,3-enyne **2b** (125 mg, 0.55 mmol) at 60 °C for a reaction time of 10 h and was purified by flash column chromatography (3:1 petroleum ether:Et_2O) to give a pale yellow oil which solidified on standing (201 mg, 88%). R_f 0.55 (1:1 petroleum ether:Et_2O); IR 1679 (C=O), 1445, 1374, 1152, 752, 640 cm^{-1}; ^{1}H NMR (400 MHz, $CDCl_3$) δ 7.44–7.25 (7H, m, 7 × Ar**H**), 7.14 (1H, td, *J* = 7.7, 1.3 Hz, Ar**H**), 6.81 (1H, dd, *J* = 7.7, 1.1 Hz, Ar**H**), 6.48 (1H, d, *J* = 12.5 Hz, OC**H**=), 5.55–5.49 (1H, m, CH_2C=C**H**), 4.77 (1H, d, *J* =

12.3 Hz, OC**H$_A$**H$_B$Ph), 4.77 (1H, dd, *J* = 12.5, 10.4 Hz, OCH=C**H**), 4.71 (1H, d, *J* = 12.3 Hz, OCH$_A$**H$_B$**Ph), 4.10 (1H, ddd, *J* = 10.4, 3.6, 1.8 Hz, C=CHC**H**), 3.38 (3H, s, NC**H**$_3$), 3.19 (3H, s, NC**H**$_3$), 2.61–2.49 (1H, m, C**H$_A$**H$_B$CH$_2$CH$_2$CH$_3$), 2.48–2.35 (1H, m, CH$_A$**H$_B$**CH$_2$CH$_2$CH$_3$), 1.68–1.54 (2H, m, C**H**$_2$CH$_2$CH$_3$), 1.55–1.42 (2H, m, C**H**$_2$CH$_3$), 0.98 (3H, t, *J* = 7.3 Hz, CH$_2$C**H**$_3$); ^{13}C NMR (100.6 MHz, CDCl$_3$) δ 172.5 (C), 167.7 (C), 150.9 (C), 149.7 (CH), 136.4 (C), 135.4 (C), 134.7 (C), 132.8 (C), 128.9 (CH), 128.5 (2 × CH), 128.0 (CH), 127.5 (CH), 127.1 (2 × CH), 126.4 (CH), 124.5 (CH), 124.4 (CH), 101.2 (CH), 71.3 (CH$_2$), 61.3 (C), 44.3 (CH), 32.2 (CH$_2$), 30.3 (CH$_2$), 28.9 (CH$_3$), 28.4 (CH$_3$), 22.6 (CH$_2$), 14.0 (CH$_3$); HRMS (ESI +ve) Exact mass calculated for $C_{28}H_{30}N_2NaO_4$ [M+Na]$^+$: 481.2098, found: 481.2124.

(Using 1.0 mol% Rhodium catalyst)

The title compound was prepared according to a modification of General Procedure A (in that 0.5 mol% of [Cp*RhCl$_2$]$_2$ (1.5 mg, 2.5 µmol) was used) from 5-arylbarbituric acid **1a** (116 mg, 0.50 mmol) and 1,3-enyne **2b** (125 mg, 0.55 mmol) at 60 °C for a reaction time of 10 h and was purified by flash column chromatography (3:1 petroleum ether:Et$_2$O) to give a pale yellow oil which solidified on standing (182 mg, 77%).

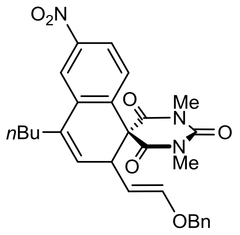

2'-[(*E*)-2-(Benzyloxy)ethenyl]-4'-butyl-1,5-dimethyl-6'-nitro-2'*H*-spiro[1,5-diazinane-3,1'-naphthalene]-2,4,6-trione (3c). The title compound was prepared according to General Procedure A from 5-arylbarbituric acid **1b** (139 mg, 0.50 mmol) and 1,3-enyne **2b** (125 mg, 0.55 mmol) at 60 °C for a reaction time of 2 h and was purified by flash column chromatography (3:1 petroleum ether:Et$_2$O) to give a yellow oil (206 mg, 82%). R$_f$ 0.20 (2:1 petroleum ether:Et$_2$O); IR 1679 (C=O), 1523 (NO$_2$), 1374 (NO$_2$), 1346, 1161, 737, 641 cm^{-1}; ^{1}H NMR (400 MHz, CDCl$_3$) δ 8.21 (1H, d, *J* = 2.4 Hz, Ar**H**), 7.98 (1H, dd, *J* = 8.5, 2.4 Hz, Ar**H**), 7.41–7.32 (3H, m, 3 × Ar**H**), 7.31–7.26 (2H, m, 2 × Ar**H**), 7.02 (1H, d, *J* = 8.5 Hz, Ar**H**), 6.46 (1H, d, *J* = 12.5 Hz, OC**H**=), 5.63–5.58 (1H, m, CH$_2$C=C**H**), 4.75 (1H, d, *J* = 12.1 Hz, OC**H$_A$**H$_B$Ph), 4.70 (1H, dd, *J* = 12.5, 10.0 Hz, OCH=C**H**), 4.70 (1H, d, *J* = 12.1 Hz, OCH$_A$**H$_B$**Ph), 3.98 (1H, ddd, *J* = 10.0, 4.1, 2.0 Hz, C=CHC**H**), 3.35 (3H, s, NC**H**$_3$), 3.20 (3H, s, NC**H**$_3$), 2.64–2.43 (2H, m, C**H**$_2$CH$_2$CH$_2$CH$_3$), 1.69–1.55 (2H, m, C**H**$_2$CH$_2$CH$_3$), 1.55–1.40 (2H, m, C**H**$_2$CH$_3$), 0.99 (3H, t, *J* = 7.3 Hz, CH$_2$C**H**$_3$); ^{13}C NMR (100.6 MHz, CDCl$_3$) δ 171.5 (C), 166.5 (C), 150.5 (C), 150.2 (CH), 148.3 (C), 139.0 (C), 137.1 (C), 136.1 (C), 134.8 (C), 128.6 (2 × CH), 128.2 (2 × CH), 127.2 (2 × CH), 125.9 (CH), 122.1 (CH), 118.8 (CH), 99.9 (CH), 71.5 (CH$_2$), 61.4 (C), 45.4 (CH), 31.9 (CH$_2$), 29.8 (CH$_2$), 29.0 (CH$_3$), 28.6 (CH$_3$), 22.5 (CH$_2$), 13.9 (CH$_3$); HRMS (ESI +ve) Exact mass calculated for $C_{28}H_{29}N_3NaO_6$ [M+Na]$^+$:

526.1949, found: 526.1958.

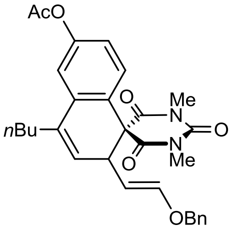

2'-[(*E*)-2-(Benzyloxy)ethenyl]-4'-butyl-1,5-dimethyl-2,4,6-trioxo-2'*H*-spiro[1,5-diazinane-3,1'-naphthalene]-6'-yl acetate (3d). The title compound was prepared according to General Procedure A from 5-arylbarbituric acid **1c** (145 mg, 0.50 mmol) and 1,3-enyne **2b** (125 mg, 0.55 mmol) at 60 °C for a reaction time of 2 h and was purified by flash column chromatography (2:1 to 1:1 petroleum ether:Et_2O) to give a yellow oil which solidified on standing (225 mg, 87%). R_f 0.12 (2:1 petroleum ether:Et_2O); IR 1762 (C=O), 1679 (C=O), 1442, 1202, 1174, 634 cm^{-1}; ^1H NMR (400 MHz, $CDCl_3$) δ 7.40–7.30 (3H, m, 3 × Ar**H**), 7.30–7.25 (2H, m, 2 × Ar**H**), 7.09 (1H, d, *J* = 2.4 Hz, Ar**H**), 6.88 (1H, dd, *J* = 8.4, 2.4 Hz, Ar**H**), 6.80 (1H, d, *J* = 8.4 Hz, Ar**H**), 6.46 (1H, d, *J* = 12.5 Hz, OC**H**=), 5.57–5.52 (1H, m, CH_2C=C**H**), 4.76 (1H, d, *J* = 12.8 Hz, OC$\mathbf{H_A}H_B$Ph), 4.75 (1H, dd, *J* = 12.5, 10.2 Hz, OCH=C**H**), 4.70 (1H, d, *J* = 12.8 Hz, OC$H_A\mathbf{H_B}$Ph), 4.07 (1H, ddd, *J* = 10.2, 4.2, 2.4 Hz, C=CHC**H**), 3.35 (3H, s, NC$\mathbf{H_3}$), 3.17 (3H, s, NC$\mathbf{H_3}$), 2.55–2.33 (2H, m, C$\mathbf{H_2}$$CH_2CH_2CH_3$), 2.29 (3H, s, COC$\mathbf{H_3}$), 1.63–1.52 (2H, m, C$\mathbf{H_2}$$CH_2CH_3$), 1.52–1.39 (2H, m, C$\mathbf{H_2}$$CH_3$), 0.97 (3H, t, *J* = 7.3 Hz, CH_2C$\mathbf{H_3}$); ^{13}C NMR (100.6 MHz, $CDCl_3$) δ 172.1 (C), 169.0 (C), 167.4 (C), 151.0 (C), 150.8 (C), 149.8 (CH), 136.5 (C), 136.3 (C), 134.8 (C), 130.0 (C), 128.5 (2 × CH), 128.0 (CH), 127.3 (CH), 127.1 (2 × CH), 125.5 (CH), 120.2 (CH), 117.6 (CH), 101.0 (CH), 71.3 (CH_2), 60.8 (C), 44.2 (CH), 32.0 (CH_2), 30.0 (CH_2), 28.9 (CH_3), 28.4 (CH_3), 22.5 (CH_2), 21.1 (CH_3), 13.9 (CH_3); HRMS (ESI +ve) Exact mass calculated for $C_{30}H_{33}N_2O_6$ $[M+H]^+$: 517.2333, found: 517.2311.

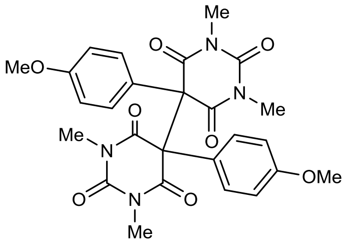

5-(4-Methoxyphenyl)-5-[5-(4-methoxyphenyl)-1,3-dimethyl-2,4,6-trioxo-1,3-diazinan-5-yl]-1,3-dimethyl-1,3-diazinane-2,4,6-trione (S10). The title compound was prepared according to General Procedure A from 5-arylbarbituric acid **1d** (131 mg, 0.50 mmol) and 1,3-enyne **2b** (125 mg, 0.55 mmol) at 60 °C for a reaction time of 2 h and was purified by flash column chromatography (1:1 petroleum ether:Et_2O) to give a yellow oil (125 mg, 96%). R_f 0.16 (1:1 petroleum ether:Et_2O); m.p. 194–195 ºC (CH_2Cl_2/petroleum ether); IR 1684 (C=O), 1522, 1437, 1374, 1189, 911, 640 cm^{-1}; ^1H NMR (400 MHz, $CDCl_3$) δ 7.05–6.64 (8H, m, 8 × Ar**H**), 3.82 (6H, s, 2 × OC$\mathbf{H_3}$), 3.37 (6H, s, 2 × NC$\mathbf{H_3}$), 3.15 (6H, s, 2 × NC$\mathbf{H_3}$); ^{13}C NMR (100.6 MHz, $(CD_3)_2SO$, 333 K) δ 159.3 (4 × C), 149.3 (2 × C), 130.6 (4 × CH), 123.0 (4 × C), 112. 9 (4 × CH), 64.4 (2 × C), 54.9(2 × CH_3), 28.7 (4 × CH_3); HRMS (ESI +ve) Exact mass calculated for $C_{26}H_{27}N_4O_8$ $[M+H]^+$: 523.1823, found: 523.1853.

2'-[(*Z*)-2-(Benzyloxy)ethenyl]-4'-butyl-6'-methoxy-1,5-dimethyl-2'*H*-spiro[1,5-diazinane-3,1'-naphthalene]-2,4,6-trione (3e), 2'-[(*Z*)-3-(Benzyloxy)prop-1-en-1-yl]-3'-butyl-5'-methoxy-1,5-dimethylspiro[1,5-diazinane-3,1'-indene]-2,4,6-trione (S11) and 5-(4-Methoxyphenyl)-5-[5-(4-methoxyphenyl)-1,3-dimethyl-2,4,6-trioxo-1,3-diazinan-5-yl]-1,3-dimethyl-1,3-diazinane-2,4,6-trione (S10)

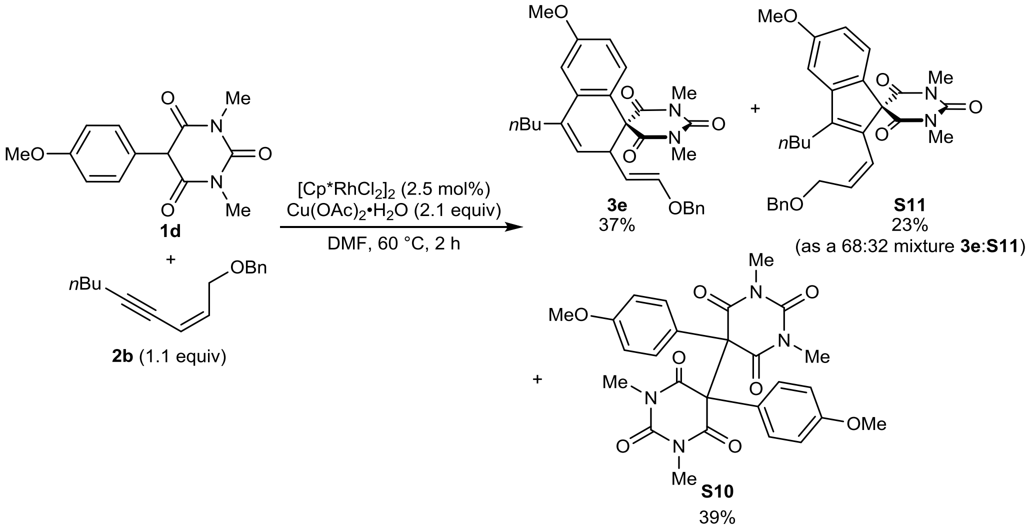

To a microwave vial, was added 5-arylbarbituric acid **1d** (131 mg, 0.50 mmol), $[Cp^*RhCl_2]_2$ (7.7 mg, 13 µmol), $Cu(OAc)_2{\cdot}H_2O$ (210 mg, 1.05 mmol), and 1,3-enyne **2b** (115 mg, 0.55 mmol). The vessel was sealed, flushed with N_2, and DMF (5 mL) was added. The reaction was then heated at 60 °C for 2 h. The reaction was cooled to room temperature, filtered through a short pad of silica using Et_2O (25 mL) as eluent, and concentrated *in vacuo*. Purification of the residue by flash column chromatography (2:1 to 1:1 petroleum ether:Et_2O) gave *spirodialin* **3e** as an off-white solid (90.8 mg, 37%), a 68:32 mixture (by ^{1}H NMR spectroscopy) of *spirodialin* **3e** and *spiroindene* **S11** respectively as a yellow oil (57.3 mg, 23%), and *dimer* **S10** as a yellow oil (50.2 mg, 39%).

Data for spirodialin 3e:

R_f 0.30 (1:1 petroleum ether:Et_2O); m.p. 61–62 ºC (CH_2Cl_2/petroleum ether); IR 1679 (C=O), 1374, 1170, 1041, 736, 640 cm^{-1}; ^{1}H NMR (400 MHz, $CDCl_3$) δ 7.40–7.31 (3H, m, 3 × Ar**H**), 7.31–7.25 (2H, m, 2 × Ar**H**), 6.94 (1H, d, *J* = 2.6 Hz, Ar**H**), 6.75–6.70 (1H, m, Ar**H**), 6.65 (1H, dd, *J* = 8.5, 2.6 Hz, Ar**H**), 6.47 (1H, d, *J* = 12.6 Hz, OC**H**=), 5.56–5.52 (1H, m, CH_2C=C**H**), 4.76 (1H, dd, J = 12.6, 10.4 Hz, OCH=C**H**), 4.76 (1H, d, *J* = 12.3 Hz, OC**H$_A$**H$_B$Ph), 4.70 (1H, d, *J* = 12.3 Hz, OCH$_A$**H$_B$**Ph), 4.06 (1H, ddd, *J* = 10.4, 4.0, 2.1 Hz, C=CHC**H**), 3.80 (3H, s, OC**H$_3$**), 3.37 (3H, s, NC**H$_3$**), 3.17 (3H, s, NC**H$_3$**), 2.56–2.46 (1H, m, C**H$_A$**H$_B$CH$_2$CH$_2$CH$_3$), 2.46–2.35 (1H, m, CH$_A$**H$_B$**CH$_2$CH$_2$CH$_3$), 1.67–1.55 (2H, m, C**H$_2$**CH$_2$CH$_3$), 1.53–1.39 (2H, m, C**H$_2$**CH$_3$), 0.98 (3H, t, *J* = 7.3 Hz, CH$_2$C**H$_3$**); ^{13}C NMR (100.6 MHz, $CDCl_3$) δ 172.6 (C), 168.1 (C), 159.9 (C), 151.0 (C),

149.7 (CH), 136.4 (C), 136.1 (C), 135.1 (C), 128.5 (2 × CH), 128.0 (CH), 127.3 (CH), 127.1 (2 × CH), 125.4 (CH), 125.0 (C), 111.5 (CH), 111.3 (CH), 101.4 (CH), 71.2 (CH_2), 60.7 (C), 55.2 (CH_3), 44.3 (CH), 32.2 (CH_2), 30.3 (CH_2), 28.9 (CH_3), 28.4 (CH_3), 22.6 (CH_2), 14.0 (CH_3); HRMS (ESI +ve) Exact mass calculated for $C_{29}H_{33}N_2O_5$ $[M+H]^+$: 489.2384, found: 489.2404.

Diagnostic signals for spiroindene S11:

^{1}H NMR (400 MHz, $CDCl_3$) δ 7.03 (1H, d, *J* = 8.3 Hz, Ar**H**), 6.91 (1H, d, *J* = 2.4 Hz, Ar**H**), 6.70 (1H, dd, *J* = 8.3, 2.4 Hz, Ar**H**), 6.11 (1H, dt, *J* = 11.8, 1.4 Hz, C**H**=CHCH$_2$OBn), 5.95 (1H, dt, *J* = 11.8, 6.3 Hz, CH=C**H**CH$_2$OBn), 4.47 (2H, s, OC**H**$_2$Ph), 4.09 (2H, dd, *J* = 6.3, 1.4 Hz, CH=CHC**H**$_2$OBn).

Data for dimer S10:

See above.

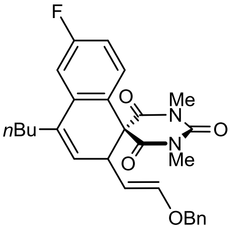

2'-[(*E*)-2-(Benzyloxy)ethenyl]-4'-butyl-6'-fluoro-1,5-dimethyl-2'*H*-spiro[1,5-diazinane-3,1'-naphthalene]-2,4,6-trione (3f). The title compound was prepared according to General Procedure A from 5-arylbarbituric acid **1e** (125 mg, 0.50 mmol) and 1,3-enyne **2b** (125 mg, 0.55 mmol) at 60 °C for a reaction time of 1 h and was purified by flash column chromatography (3:1 petroleum ether:Et_2O) to give an off-white solid (185 mg, 78%). R_f 0.48 (2:1 petroleum ether:Et_2O); m.p. 96–97 °C (CH_2Cl_2/petroleum ether); IR 1677 (C=O), 1376, 1174, 872, 735, 646 cm^{-1}; ^{1}H NMR (400 MHz, $CDCl_3$) δ 7.41–7.31 (3H, m, 3 × Ar**H**), 7.31–7.26 (2H, m, 2 × Ar**H**), 7.09 (1H, dd, *J* = 10.3, 2.5 Hz, Ar**H**), 6.87–6.80 (1H, m, Ar**H**), 6.78 (1H, dd, *J* = 8.5, 5.6 Hz, Ar**H**), 6.47 (1H, d, *J* = 12.5 Hz, OC**H**=), 5.56 (1H, d, *J* = 2.0 Hz, CH$_2$C=C**H**), 4.76 (1H, d, *J* = 12.3 Hz, OC$\mathbf{H_A}$H$_B$Ph), 4.74 (1H, dd, *J* = 12.5, 10.3 Hz, OCH=C**H**), 4.70 (1H, d, *J* = 12.3 Hz, OCH$_A$$\mathbf{H_B}$Ph), 4.04 (1H, ddd, *J* = 10.3, 4.1, 2.0 Hz, C=CHC**H**), 3.36 (3H, s, NC$\mathbf{H_3}$), 3.18 (3H, s, NC$\mathbf{H_3}$), 2.55–2.44 (1H, m, C$\mathbf{H_A}$H$_B$CH$_2$CH$_2$CH$_3$), 2.44–2.34 (1H, m, CH$_A$$\mathbf{H_B}CH_2CH_2CH_3$), 1.65–1.53 (2H, m, C$\mathbf{H_2}CH_2CH_3$), 1.53–1.41 (2H, m, C$\mathbf{H_2}CH_3$), 0.98 (3H, t, *J* = 7.3 Hz, CH$_2$C$\mathbf{H_3}$); ^{13}C NMR (100.6 MHz, $CDCl_3$) δ 172.3 (C), 167.5 (C), 164.9 (d, *J* = 247.0 Hz, C), 150.8 (C), 149.9 (CH), 137.4 (d, *J* = 8.0 Hz, C), 136.3 (C), 134.9 (d, *J* = 1.9 Hz, C), 128.5 (2 × CH), 128.3 (d, *J* = 3.3 Hz, C), 128.1 (d, *J* = 8.4 Hz, CH), 128.0 (CH), 127.1 (2 × CH), 125.6 (CH), 113.8 (d, *J* = 22.0 Hz, CH), 111.6 (d, *J* = 23.0 Hz, CH), 100.8 (CH), 71.3 (CH_2), 60.8 (C), 44.7 (CH), 32.1 (CH_2), 30.1 (CH_2), 28.9 (CH_3), 28.4 (CH_3), 22.5 (CH_2), 13.9 (CH_3); ^{19}F NMR (376 MHz, $CDCl_3$) δ –112.3 (m); HRMS (ESI +ve) Exact mass calculated for $C_{28}H_{29}FN_2NaO_4$ $[M+Na]^+$: 499.2004, found: 499.2023.

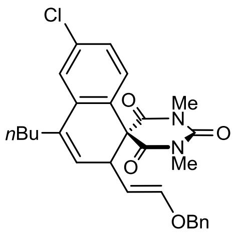

2'-[(*E*)-2-(Benzyloxy)ethenyl]-4'-butyl-6'-chloro-1,5-dimethyl-2'*H*-spiro[1,5-diazinane-3,1'-naphthalene]-2,4,6-trione (3g). The title compound was prepared according to General Procedure A from 5-arylbarbituric acid **1f** (133 mg, 0.50 mmol) and 1,3-enyne **2b** (125 mg, 0.55 mmol) at 60 °C for a reaction time of 1 h and was purified by flash column chromatography (3:1 petroleum ether:Et_2O) to give a pale yellow oil which solidified on standing (180 mg, 73%). R_f 0.47 (2:1 petroleum ether:Et_2O); IR 1675 (C=O), 1374, 1167, 1096, 737, 634 cm^{-1}; ^1H NMR (400 MHz, $CDCl_3$) δ 7.40–7.31 (4H, m, 4 × Ar**H**), 7.30–7.26 (2H, m, 2 × Ar**H**), 7.11 (1H, dd, *J* = 8.3, 2.2 Hz, Ar**H**), 6.75 (1H, d, *J* = 8.3 Hz, Ar**H**), 6.46 (1H, d, *J* = 12.5 Hz, OC**H**=), 5.56–5.52 (1H, m, CH_2C=C**H**), 4.76 (1H, d, *J* = 12.2 Hz, $OCH_A\mathbf{H}_B$Ph), 4.72 (1H, d, *J* = 12.5, 10.4 Hz, OCH=C**H**), 4.70 (1H, d, *J* = 12.2 Hz, $OCH_A\mathbf{H}_B$Ph), 4.02 (1H, ddd, *J* = 10.4, 4.1, 2.2 Hz, C=CHC**H**), 3.35 (3H, s, $NC\mathbf{H}_3$), 3.18 (3H, s, $NC\mathbf{H}_3$), 2.55–2.34 (2H, m, $C\mathbf{H}_2CH_2CH_2CH_3$), 1.65–1.53 (2H, m, $C\mathbf{H}_2CH_2CH_3$), 1.53–1.41 (2H, m, $C\mathbf{H}_2CH_3$), 0.99 (3H, t, *J* = 7.3 Hz, $CH_2C\mathbf{H}_3$); ^{13}C NMR (100.6 MHz, $CDCl_3$) δ 172.1 (C), 167.4 (C), 150.8 (C), 149.9 (CH), 136.8 (C), 136.3 (C), 135.0 (C), 134.8 (C), 131.0 (C), 128.6 (2 × CH), 128.1 (CH), 127.9 (CH), 127.2 (CH), 127.15 (2 × CH), 125.6 (CH), 124.6 (CH), 100.7 (CH), 71.4 (CH_2), 60.9 (C), 44.7 (CH), 32.0 (CH_2), 30.0 (CH_2), 28.9 (CH_3), 28.5 (CH_3), 22.5 (CH_2), 13.9 (CH_3); HRMS (ESI +ve) Exact mass calculated for $C_{28}H_{30}ClN_2O_4$ $[M+H]^+$: 493.1889, found: 493.1897.

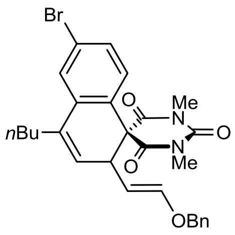

2'-[(*E*)-2-(Benzyloxy)ethenyl]-4'-butyl-6'-bromo-1,5-dimethyl-2'*H*-spiro[1,5-diazinane-3,1'-naphthalene]-2,4,6-trione (3h). The title compound was prepared according to General Procedure A from 5-arylbarbituric acid **1g** (155 mg, 0.50 mmol) and 1,3-enyne **2b** (125 mg, 0.55 mmol) at 60 °C for a reaction time of 1 h and was purified by flash column chromatography (3:1 petroleum ether:Et_2O) to give an off-white solid (194 mg, 73%). R_f 0.45 (2:1 petroleum ether:Et_2O); m.p. 77–78 ºC (CH_2Cl_2/petroleum ether); IR 1674 (C=O), 1643 (C=O), 1373, 1164, 1094, 947, 738, 646 cm^{-1}; ^1H NMR (400 MHz, $CDCl_3$) δ 7.51 (1H, d, *J* = 2.0 Hz, Ar**H**), 7.41–7.31 (3H, m, 3 × Ar**H**), 7.30–7.24 (3H, m, 3 × Ar**H**), 6.69 (1H, d, *J* = 8.2 Hz, Ar**H**), 6.46 (1H, d, *J* = 12.5 Hz, OC**H**=), 5.56–5.51 (1H, m, CH_2C=C**H**), 4.76 (1H, d, *J* = 12.2 Hz, $OC\mathbf{H}_AH_B$Ph), 4.72 (1H, d, *J* = 12.5 Hz, OCH=C**H**), 4.70 (1H, d, *J* = 12.2 Hz, $OCH_A\mathbf{H}_\mathbf{B}$Ph), 4.02 (1H, dd, *J* = 10.4, 2.0 Hz, C=CHC**H**), 3.35 (3H, s, $NC\mathbf{H}_3$), 3.18 (3H, s, $NC\mathbf{H}_3$), 2.56–2.33 (2H, m, $C\mathbf{H}_2CH_2CH_2CH_3$), 1.65–1.53 (2H, m, $C\mathbf{H}_2CH_2CH_3$), 1.53–1.40 (2H, m, $C\mathbf{H}_2CH_3$), 0.99 (3H, t, *J* = 7.3 Hz, $CH_2C\mathbf{H}_3$); ^{13}C NMR (100.6 MHz, $CDCl_3$) δ 172.0 (C), 167.2 (C), 150.7 (C), 149.9 (CH), 137.0 (C), 136.2 (C), 134.7 (C), 131.5 (C), 130.2 (CH), 128.5 (2 × CH), 128.1 (CH), 128.1 (CH), 127.4 (CH), 127.1 (2 ×

CH), 125.5 (CH), 123.2 (C), 100.7 (CH), 71.4 (CH_2), 60.9 (C), 44.6 (CH), 32.0 (CH_2), 30.0 (CH_2), 28.9 (CH_3), 28.5 (CH_3), 22.5 (CH_2), 13.9 (CH_3); HRMS (ESI +ve) Exact mass calculated for $C_{28}H_{29}BrN_2NaO_4$ $[M+Na]^+$: 559.1203, found: 559.1212.

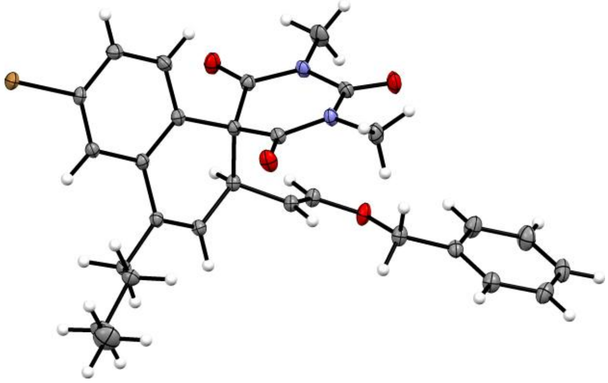

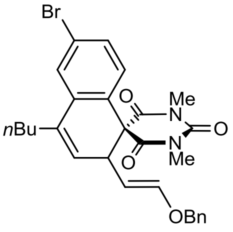

CCDC 1057433
3h

A single crystal suitable for X-ray crystallography was prepared by slow evaporation from a solution of **3h** in CH_2Cl_2 and hexanes.

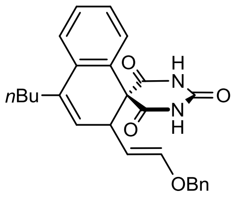

2'-[(*E*)-2-(Benzyloxy)ethenyl]-4'-butyl-2'*H*-spiro[1,5-diazinane-3,1'-naphthalene]-2,4,6-trione (3i). The title compound was prepared according to a slight modification of General Procedure A from 5-arylbarbituric acid **1h** (102 mg, 0.50 mmol) and 1,3-enyne **2b** (125 mg, 0.55 mmol) at 120 °C for a reaction time of 2 h and was purified by flash column chromatography (1:1 petroleum ether:Et_2O) to give a yellow solid (145 mg, 67%). R_f 0.16 (1:1 petroleum ether:Et_2O); IR 3217 (NH), 3098 (NH), 1708 (C=O), 1335, 1157, 940, 641 cm^{-1}; ^{1}H NMR (400 MHz, $CDCl_3$) δ 9.14 (1H, s, N**H**), 8.55 (1H, s, N**H**), 7.42–7.24 (7H, m, 7 × Ar**H**), 7.13 (1H, td, *J* = 7.7, 1.0 Hz, Ar**H**), 6.96 (1H, dd, *J* = 7.7, 1.0 Hz, Ar**H**), 6.53 (1H, d, *J* = 12.5 Hz, OC**H**=), 5.55–5.48 (1H, m, CH_2C=C**H**), 4.89 (1H, dd, *J* = 12.5, 10.4 Hz, OCH=C**H**), 4.77 (1H, d, *J* = 12.1 Hz, OC$\mathbf{H_A}$$H_B$Ph), 4.71 (1H, d, *J* = 12.1 Hz, OC$H_A$$\mathbf{H_B}$Ph), 4.14–4.06 (1H, m, C=CHC**H**), 2.61–2.47 (1H, m, C$\mathbf{H_A}$$H_B$$CH_2CH_2CH_3$), 2.45–2.33 (1H, m, C$H_A$$\mathbf{H_B}$$CH_2CH_2CH_3$), 1.64–1.51 (2H, m, C$\mathbf{H_2}$$CH_2CH_3$), 1.51–1.37 (2H, m, C$\mathbf{H_2}$$CH_3$), 0.96 (3H, t, *J* = 7.3 Hz, CH_2C$\mathbf{H_3}$); ^{13}C NMR (100.6 MHz, $CDCl_3$) δ 173.2 (C), 168.0 (C), 150.2 (CH), 149.0 (C), 136.4 (C), 135.5 (C), 134.7 (C), 131.6 (C), 129.2 (CH), 128.5 (2 × CH), 128.0 (CH), 127.6 (CH), 127.4 (2 × CH), 126.4 (CH), 124.6 (CH), 124.5 (CH), 101.1 (CH), 71.4 (CH_2), 61.1 (C), 43.2 (CH), 32.2 (CH_2), 30.3 (CH_2), 22.5 (CH_2), 13.9 (CH_3); HRMS (ESI +ve) Exact mass calculated for $C_{26}H_{26}N_2NaO_4$ $[M+Na]^+$: 453.1785, found: 453.1804.

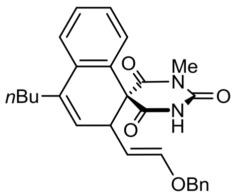

2'-[(*E*)-2-(Benzyloxy)ethenyl]-4'-butyl-1-methyl-2'*H*-spiro[1,5-diazinane-3,1'-naphthalene]-2,4,6-trione (3j). The title compound was prepared according to General Procedure A from 5-arylbarbituric acid **1i** (109 mg, 0.50 mmol) and 1,3-enyne **2b** (125 mg, 0.55 mmol) at 120 °C for a reaction time of 2 h and was purified by flash column chromatography (2:1 petroleum ether:Et_2O) to give a 1:1 mixture of inseparable diastereoisomers (by ^{1}H NMR spectroscopy) as a colorless glass (136 mg, 61%). R_f 0.11 (2:1 petroleum ether:Et_2O); m.p. 124–127 ºC (CH_2Cl_2/hexane); IR 3153 (NH), 1711 (C=O), 1685 (C=O), 1442, 1345, 1160, 749, 641 cm^{-1}; ^{1}H NMR (400 MHz, $CDCl_3$) δ 9.28 (1H, s, N**H**), 8.71 (1H, s, N**H**), 7.44–7.28 (7H, m, 7 × Ar**H**), 7.18–7.11 (1H, m, Ar**H**), 6.96 (0.5H, dd, *J* = 7.7, 1.1 Hz, Ar**H**), 6.84 (0.5H, dd, *J* = 7.7, 1.1 Hz, Ar**H**), 6.55 (0.5H, d, *J* = 12.5 Hz, OC**H**=), 6.50 (0.5H, d, *J* = 12.5 Hz, OC**H**=), 5.56–5.51 (1H, m, CH_2C=C**H**), 4.89 (0.5H, d, *J* = 12.5, 10.4 Hz, OCH=C**H**), 4.79 (0.5H, d, *J* = 12.5, 10.4 Hz, OCH=C**H**), 4.79 (0.5H, d, *J* = 12.2 Hz, OC$\mathbf{H_A}$$H_B$Ph), 4.77 (0.5H, d, *J* = 12.2 Hz, OC$\mathbf{H_A}$$H_B$Ph), 4.73 (0.5H, d, *J* = 12.2 Hz, OC$H_A$$\mathbf{H_B}$Ph), 4.71 (0.5H, d, *J* = 12.2 Hz, OC$H_A$$\mathbf{H_B}$Ph), 4.15–4.08 (1H, m, C=CHC**H**), 3.33 (1.5H, s, NC$\mathbf{H_3}$), 3.14 (1.5H, s, NC$\mathbf{H_3}$), 2.62–2.50 (1H, m, C$\mathbf{H_A}$$H_B$$CH_2CH_2CH_3$), 2.49–2.34 (1H, m, C$H_A$$\mathbf{H_B}$$CH_2CH_2CH_3$), 1.66–1.54 (2H, m, C$\mathbf{H_2}$$CH_2CH_3$), 1.54–1.38 (2H, m, C$\mathbf{H_2}$$CH_3$), 0.99 (1.5H, t, *J* = 7.2 Hz, CH_2C$\mathbf{H_3}$), 0.98 (1.5H, t, *J* = 7.2 Hz, CH_2C$\mathbf{H_3}$); ^{13}C NMR (100.6 MHz, $CDCl_3$) δ 173.2 (C), 172.4 (C), 168.4 (C), 167.2 (C), 150.1 (CH), 150.0 (C), 149.9 (C), 149.8 (CH), 136.4 (C), 136.3 (C), 135.5 (C), 135.3 (C), 134.7 (C), 134.6 (C), 132.3 (C), 132.0 (C), 129.1 (CH), 129.0 (CH), 128.5 (4 × CH), 127.99 (CH), 127.96 (CH), 127.6 (CH), 127.5 (CH), 127.3 (2 × CH), 127.2 (2 × CH), 126.4 (2 × CH), 124.5 (CH), 124.5 (CH), 124.4 (CH), 124.4 (CH), 101.2 (CH), 100.9 (CH), 71.3 (CH_2), 71.3 (CH_2), 61.5 (C), 61.0 (C), 43.8 (CH), 43.6 (CH), 32.2 (2 × CH_2), 30.3 (CH_2), 30.2 (CH_2), 28.1 (CH_3), 27.6 (CH_3), 22.6 (CH_2), 22.5 (CH_2), 13.9 (2 × CH_3); HRMS (ESI +ve) Exact mass calculated for $C_{27}H_{28}N_2NaO_4$ $[M+Na]^+$: 467.1941, found: 467.1935.

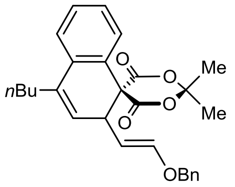

2'-[(*E*)-2-(Benzyloxy)ethenyl]-4'-butyl-6,6-dimethyl-2'*H*-spiro[1,5-dioxane-3,1'-naphthalene]-2,4-dione (6). The title compound was prepared according to a modification of General Procedure A (in that 5.0 mol% of $[Cp^*RhCl_2]_2$ (15.4 mg, 25 µmol) was used) from 2-aryl Meldrum's acid **1j** (110 mg, 0.50 mmol) and 1,3-enyne **2b** (125 mg, 0.55 mmol) at 60 °C for a reaction time of 5 h and was purified by flash column chromatography (4:1 petroleum ether:Et_2O) to give a yellow oil which solidified on standing (71.6 mg, 32%). R_f 0.23 (3:1 petroleum ether:Et_2O); IR 1781 (C=O), 1740 (C=O), 1638, 1265, 1159, 917, 646 cm^{-1}; ^{1}H NMR (400 MHz, $CDCl_3$) δ 7.42–7.31 (7H, m, 7 × Ar**H**), 7.19 (1H, td, *J* = 7.5, 1.4 Hz, Ar**H**), 7.10 (1H, dd, *J* = 7.5, 1.1 Hz, Ar**H**), 6.59 (1H, d, *J* = 12.5 Hz,

OC**H**=), 5.58–5.51 (1H, m, CH_2C=C**H**), 4.96 (1H, dd, *J* = 12.5, 10.4 Hz, OCH=C**H**), 4.80 (1H, d, *J* = 11.9 Hz, OC**H$_A$**H_BPh), 4.74 (1H, d, *J* = 11.9 Hz, OCH_A**H$_B$**Ph), 4.14 (1H, ddd, *J* = 10.4, 3.7, 2.1 Hz, C=CHC**H**), 2.64–2.51 (1H, m, C**H$_A$**$H_B$$CH_2$$CH_2$$CH_3$), 2.47–2.35 (1H, m, C$H_A$**H$_B$**$CH_2$$CH_2$$CH_3$), 1.81 (3H, s, C(C**H**$_3$)$_2$), 1.68 (3H, s, C(C**H**$_3$)$_2$), 1.62–1.53 (2H, m, C**H**$_2$$CH_2$$CH_3$), 1.53–1.40 (2H, m, C**H**$_2$$CH_3$), 0.97 (3H, t, *J* = 7.2 Hz, CH_2C**H**$_3$); ^{13}C NMR (100.6 MHz, $CDCl_3$) δ 170.4 (C), 165.3 (C), 150.5 (CH), 136.4 (C), 135.7 (C), 134.6 (C), 131.7 (C), 129.2 (CH), 128.5 (2 × CH), 128.1 (CH), 127.5 (3 × CH), 126.7 (CH), 124.8 (CH), 124.5 (CH), 105.6 (C), 101.6 (CH), 71.6 (CH_2), 60.7 (C), 43.3 (CH), 32.2 (CH_2), 30.2 (CH_2), 29.7 (CH_3), 29.7 (CH_3), 22.5 (CH_2), 14.0 (CH_3); HRMS (ESI +ve) Exact mass calculated for $C_{28}H_{30}NaO_5$ [M+Na]$^+$: 469.1985, found: 469.2031.

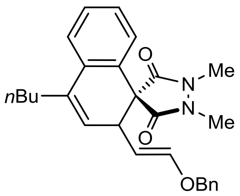

(*E*)-2-(2-(Benzyloxy)vinyl)-4-butyl-1',2'-dimethyl-2*H*-spiro[naphthalene-1,4'-pyrazolidine]-3',5'-dione (8). The title compound was prepared according to a modification of General Procedure A (in that the reaction was carried out on a 0.50 mmol scale with respect to the 1,3-enyne **2b**, NaOAc·3H_2O (3.0 equiv) was added and no Cu(OAc)$_2$ was used) from hydrazide **7** (204 mg, 1.00 mmol), NaOAc·3H_2O (204 mg, 3.00 mmol) and 1,3-enyne **2b** (114 mg, 0.50 mmol) at 60 °C for a reaction time of 36 h and was purified by flash column chromatography (50:1 to 5:1 petroleum ether:acetone) to give a colorless oil (102 mg, 47%). R_f 0.60 (5:1 PhMe/acetone); IR 1741 (C=O), 1695 (C=O), 1489, 1454, 1336, 1247, 1157, 1029, 940, 741 cm^{-1}; ^{1}H NMR (300 MHz, $(CD_3)_2CO$) δ 7.46–7.26 (7H, m, 7 × Ar**H**), 7.16 (1H, td, *J* = 7.5, 1.3 Hz, Ar**H**), 6.89 (1H, dd, *J* = 7.6, 1.1 Hz, Ar**H**), 6.53 (H, d, *J* = 12.4 Hz, OC**H**=CH), 5.49 (1H, dt, *J* = 2.1, 1.0 Hz, CH_2C=C**H**), 4.84 (1H, d, *J* = 12.3 Hz, OC**H$_A$**H_BPh), 4.81 (1H, dd, *J* = 12.7, 10.0 Hz, OCH=C**H**), 4.78 (1H, d, *J* = 12.3 Hz, OCH_A**H$_B$**Ph), 3.61 (1H, ddd, *J* = 10.0, 3.4, 2.3 Hz, C=CHC**H**), 3.17 (3H, s, NC**H**$_3$), 3.06 (3H, s, NC**H**$_3$), 2.61 (1H, dtdd, *J* = 14.6, 7.1, 2.6, 1.4 Hz, C**H$_A$**$H_B$$CH_2$$CH_2$$CH_3$), 2.37 (1H, dt, *J* = 14.6, 7.2 Hz, CH_A**H$_B$**$CH_2$$CH_2$$CH_3$), 1.64–1.35 (4H, m, C**H**$_2$C**H**$_2$$CH_3$), 0.94 (1H, t, *J* = 7.1 Hz, C**H**$_3$); ^{13}C NMR (75.5 MHz, $(CD_3)_2CO$) δ 174.0 (C), 169.8 (C), 150.8 (CH), 138.2 (C), 136.7 (C), 135.5 (C), 132.9 (C), 129.2 (CH), 129.2 (CH), 128.6 (CH), 128.3 (CH), 128.3 (CH), 127.5 (CH), 125.7 (CH), 124.8 (CH), 101.9 (CH), 71.7 (CH_2), 58.4 (C), 41.7 (CH), 32.9 (CH_2), 31.1 (CH_2), 30.6 (2 × CH_3), 23.0 (CH_2), 14.3 (CH_3); HRMS (ESI +ve) Exact mass calculated for $C_{27}H_{31}N_2O_3$ [M+H]$^+$: 431.2329, found 431.2337.

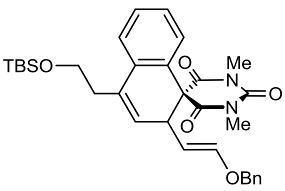

2'-[(*E*)-2-(Benzyloxy)ethenyl]-4'-{2-[(*tert*-butyldimethylsilyl)oxy]ethyl}-1,5-dimethyl-2'*H*-spiro[1,5-diazinane-3,1'-naphthalene]-2,4,6-trione (3k). The title compound was prepared according to General Procedure A from 5-arylbarbituric acid **1a** (116 mg, 0.50 mmol) and 1,3-enyne **2c** (182 mg, 0.55 mmol) at 60 °C for a reaction time of 2 h and was purified by flash column chromatography (4:1 to 1:1 petroleum ether:Et_2O) to give a colorless oil (168 mg, 60%). R_f 0.35 (2:1 petroleum ether:Et_2O); IR 1679 (C=O), 1376, 1170, 1091, 834, 772, 640 cm^{-1}; ^{1}H NMR (400 MHz, $CDCl_3$) δ 7.44 (1H, dd, *J* = 7.9, 0.9 Hz, Ar**H**), 7.40–7.26 (6H, m, 6 × Ar**H**), 7.13 (1H, td, *J* = 7.6, 1.2 Hz, Ar**H**), 6.79 (1H, dd, *J* = 7.7, 1.1 Hz, Ar**H**), 6.47 (1H, d, *J* = 12.5 Hz, OC**H**=), 5.56 (1H, dt, *J* = 2.4, 1.2 Hz, CH_2C=C**H**), 4.77 (1H, d, *J* = 12.2 Hz, OC$\mathbf{H_A}H_B$Ph), 4.75 (1H, t, J = 12.5 Hz, OCH=C**H**), 4.71 (1H, d, *J* = 12.2 Hz, OC$H_A\mathbf{H_B}$Ph), 4.13–4.05 (1H, m, C=CHC**H**), 3.92–3.80 (2H, m, TBSOC$\mathbf{H_2}$), 3.37 (3H, s, NC$\mathbf{H_3}$), 3.17 (3H, s, NC$\mathbf{H_3}$), 2.84–2.65 (2H, m, TBSOCH_2C$\mathbf{H_2}$), 0.92 (9H, s, C(C$\mathbf{H_3}$)$_3$), 0.09 (3H, s, SiC$\mathbf{H_3}$), 0.08 (3H, s, SiC$\mathbf{H_3}$); ^{13}C NMR (100.6 MHz, $CDCl_3$) δ 172.4 (C), 167.6 (C), 150.9 (C), 149.8 (CH), 136.4 (C), 134.6 (C), 132.6 (C), 132.3 (C), 129.0 (CH), 128.6 (2 × CH), 128.0 (CH), 127.7 (CH), 127.1 (2 × CH), 126.5 (CH), 126.4 (CH), 124.5 (CH), 101.1 (CH), 71.3 (CH_2), 62.5 (CH_2), 61.3 (C), 44.3 (CH), 36.0 (CH_2), 28.9 (CH_3), 28.4 (CH_3), 25.9 (3 × CH_3), 18.3 (C), −5.3 (2 × CH_3); HRMS (ESI +ve) Exact mass calculated for $C_{32}H_{41}N_2O_5Si$ $[M+H]^+$: 561.2779, found: 561.2825.

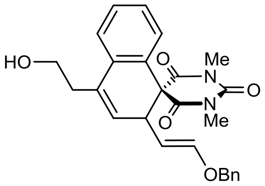

2'-[(*E*)-2-(Benzyloxy)ethenyl]-4'-(2-hydroxyethyl)-1,5-dimethyl-2'*H*-spiro[1,5-diazinane-3,1'-naphthalene]-2,4,6-trione (3l). The title compound was prepared according to General Procedure A from 5-arylbarbituric acid **1a** (108 mg, 0.50 mmol) and 1,3-enyne **2d** (119 mg, 0.55 mmol) at 60 °C for a reaction time of 2 h and was purified by flash column chromatography (1:1 petroleum ether:Et_2O) to give an off-white solid (142 mg, 64%). R_f 0.40 (1:1 petroleum ether:Et_2O); m.p. 98–99 ºC (CH_2Cl_2/petroleum ether); IR 3393 (OH), 1674 (C=O), 1634 (C=O), 1375, 1148, 1045, 739 cm^{-1}; ^{1}H NMR (400 MHz, $CDCl_3$) δ 7.40–7.25 (7H, m, 7 × Ar**H**), 7.18–7.13 (1H, m, Ar**H**), 6.82 (1H, d, *J* = 8.2 Hz, Ar**H**), 6.49 (1H, d, *J* = 12.3 Hz, OC**H**=), 5.66 (1H, d, *J* = 2.1 Hz, CH_2C=C**H**), 4.77 (1H, d, *J* = 12.6 Hz, OC$\mathbf{H_A}H_B$Ph), 4.72 (1H, d, *J* = 12.3 Hz, OCH=C**H**), 4.69 (1H, d, *J* = 12.6 Hz, OC$H_A\mathbf{H_B}$Ph), 4.14 (1H, dt, *J* = 10.4, 2.5 Hz, C=CHC**H**), 3.78–3.64 (2H, m, OC$\mathbf{H_2}$), 3.36 (3H, s, NC$\mathbf{H_3}$), 3.15 (3H, s, NC$\mathbf{H_3}$), 3.03–2.92 (1H, m, C$\mathbf{H_A}H_B$$CH_2$OH), 2.55 (1H, ddd, *J* = 14.0, 10.6, 5.3 Hz, C$H_A\mathbf{H_B}$$CH_2$OH); ^{13}C NMR (100.6 MHz, $CDCl_3$) δ 172.1 (C), 168.4 (C), 150.5 (C), 150.1 (CH), 136.2 (C), 133.7 (C), 133.5 (C), 132.5 (C), 129.0 (CH), 128.5 (2 × CH), 128.3 (CH), 128.0 (CH), 128.0 (CH), 127.0 (2 × CH), 126.7 (CH), 124.6 (CH), 100.2 (CH), 71.3

(CH_2), 61.2 (C), 59.2 (CH_2), 44.9 (CH), 36.0 (CH_2), 28.8 (CH_3), 28.5 (CH_3); HRMS (ESI +ve) Exact mass calculated for $C_{26}H_{26}N_2NaO_5$ $[M+Na]^+$: 469.1734, found: 469.1750.

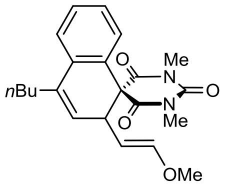

4'-Butyl-2'-[(*E*)-2-methoxyethenyl]-1,5-dimethyl-2'*H*-spiro[1,5-diazinane-3,1'-naphthalene]-2,4,6-trione (3m). The title compound was prepared according to General Procedure A from 5-arylbarbituric acid **1a** (116 mg, 0.50 mmol) and 1,3-enyne **2e** (83.6 mg, 0.55 mmol) at 60 °C for a reaction time of 1 h and was purified by flash column chromatography (3:1 petroleum ether:Et_2O) to give a yellow oil (152 mg, 80%). R_f 0.32 (2:1 petroleum ether:Et_2O); IR 1677 (C=O), 1375, 1174, 947, 751, 634 cm^{-1}; ^{1}H NMR (300 MHz, $CDCl_3$) δ 7.40 (1H, dd, *J* = 7.7, 1.2 Hz, Ar**H**), 7.31 (1H, td, *J* = 7.7, 1.2 Hz, Ar**H**), 7.14 (1H, td, *J* = 7.7, 1.2 Hz, Ar**H**), 6.83 (1H, dd, *J* = 7.7, 1.2 Hz, Ar**H**), 6.45 (1H, d, *J* = 12.6 Hz, OC**H**=), 5.48 (1H, dt, *J* = 2.5, 1.3 Hz, CH_2C=C**H**), 4.62 (1H, dd, *J* = 12.6, 10.4 Hz, OCH=C**H**), 4.06 (1H, ddd, *J* = 10.4, 4.2, 2.2 Hz, C**H**), 3.48 (3H, s, OC$\mathbf{H}_3$), 3.44 (3H, s, NC$\mathbf{H}_3$), 3.25 (3H, s, NC$\mathbf{H}_3$), 2.62–2.48 (1H, m, C$\mathbf{H_A}$$H_B$$CH_2CH_2CH_3$), 2.48–2.36 (1H, m, C$H_A$$\mathbf{H_B}$$CH_2CH_2CH_3$), 1.66–1.53 (2H, m, C$\mathbf{H}_2$$CH_2CH_3$), 1.52–1.40 (2H, m, C$\mathbf{H}_2$$CH_3$), 0.97 (3H, t, *J* = 7.2 Hz, C$\mathbf{H}_3$); ^{13}C NMR (100.6 MHz, $CDCl_3$) δ 172.8 (C), 167.7 (C), 151.2 (C), 151.1 (CH), 135.8 (C), 134.8 (C), 132.7 (C), 129.0 (CH), 127.6 (CH), 126.7 (CH), 124.4 (CH), 124.2 (CH), 99.1 (CH), 61.6 (C), 56.2 (CH_3), 44.7 (CH), 32.3 (CH_2), 30.4 (CH_2), 29.0 (CH_3), 28.4 (CH_3), 22.7 (CH_2), 14.0 (CH_3); HRMS (ESI +ve) Exact mass calculated for $C_{22}H_{27}N_2O_4$ $[M+H]^+$: 383.1965, found: 383.1978.

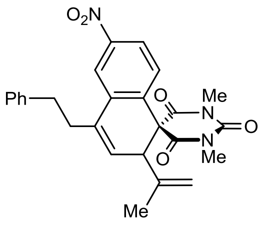

1,5-Dimethyl-6'-nitro-4'-(2-phenylethyl)-2'-(prop-1-en-2-yl)-2'*H*-spiro[1,5-diazinane-3,1'-naphthalene]-2,4,6-trione (3n). The title compound was prepared according to General Procedure A from 5-arylbarbituric acid **1b** (139 mg, 0.50 mmol) and 1,3-enyne **2a** (101 mg, 0.55 mmol) at 60 °C for a reaction time of 2 h and was purified by flash column chromatography (2:1 to 1:1 petroleum ether:Et_2O) to give an off-white solid (210 mg, 95%). R_f 0.17 (2:1 petroleum ether:Et_2O); m.p. 176–177 °C (CH_2Cl_2/petroleum ether); IR 1684 (C=O), 1675 (C=O), 1522 (NO_2), 1372 (NO_2), 1351, 749, 640 cm^{-1}; ^{1}H NMR (400 MHz, $CDCl_3$) δ 8.29 (1H, d, *J* = 2.3 Hz, Ar**H**), 8.04 (1H, dd, *J* = 8.5, 2.3 Hz, Ar**H**), 7.36–7.29 (3H, m, 3 × Ar**H**), 7.32 (1H, s, Ar**H**), 7.26–7.20 (1H, m, Ar**H**), 7.12 (1H, d, *J* = 8.5 Hz, Ar**H**), 5.66 (1H, d, *J* = 3.5 Hz, CH_2C=C**H**), 4.99 (1H, s, C$\mathbf{H_A}$$H_B$=), 4.86 (1H, s, C$H_A$$\mathbf{H_B}$=), 4.08–4.03 (1H, m, C=CHC**H**), 3.41 (3H, s, NC$\mathbf{H}_3$), 3.26 (3H, s, NC$\mathbf{H}_3$), 3.02–2.83 (4H, m, C$\mathbf{H}_2$C$\mathbf{H}_2$), 1.59 (3H, s, =CC$\mathbf{H}_3$); ^{13}C NMR (100.6 MHz, $CDCl_3$) δ 171.0 (C), 166.7 (C), 150.6 (C), 148.2 (C), 142.3 (C), 141.1 (C), 138.8 (C), 137.0 (C), 135.0 (C), 128.7 (CH), 128.4 [2 × (2 × CH)], 126.1 (CH), 124.0 (CH), 122.3 (CH), 118.5 (CH),

117.3 (CH_2), 60.6 (C), 53.4 (CH), 34.2 (CH_2), 34.1 (CH_2), 29.1 (CH_3), 28.8 (CH_3), 20.3 (CH_3); HRMS (ESI +ve) Exact mass calculated for $C_{26}H_{26}N_3O_5$ $[M+H]^+$: 460.1867, found: 460.1881.

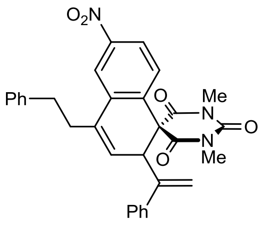

1,5-Dimethyl-6'-nitro-2'-(1-phenylethenyl)-4'-(2-phenylethyl)-2'*H*-spiro[1,5-diazinane-3,1'-naphthalene]-2,4,6-trione (3o). The title compound was prepared according to General Procedure A from 5-arylbarbituric acid **1b** (139 mg, 0.50 mmol) and 1,3-enyne **2f** (135 mg, 0.55 mmol) at 60 °C for a reaction time of 2 h and was purified by trituration with hot EtOH, dissolving in $CHCl_3$ and evaporation *in vacuo* to give an off-white solid (204 mg, 78%). R_f 0.39 (1:1 petroleum ether:Et_2O); m.p. 149–150 ºC (CH_2Cl_2/petroleum ether); IR 1679 (C=O), 1374, 1170, 1049, 736, 640 cm^{-1}; ^{1}H NMR (400 MHz, $(CD_3)_2SO$) δ 8.18 (1H, d, J = 2.4 Hz, Ar**H**), 8.01 (1H, dd, J = 8.5, 2.4 Hz, Ar**H**), 7.39–7.30 (7H, m, 7 × Ar**H**), 7.28–7.23 (1H, m, Ar**H**), 7.23–7.18 (3H, m, 3 × Ar**H**), 5.87 (1H, d, J = 2.1 Hz, CH_2C=C**H**), 5.50 (1H, s, C$\mathbf{H}_A$$H_B$=), 5.19 (1H, s, $CH_A$$\mathbf{H}_\mathbf{B}$=), 4.94 (1H, s, C=CHC**H**), 3.18 (3H, s, NC$\mathbf{H}_3$), 3.09–2.84 (4H, m, C$\mathbf{H}_2$C$\mathbf{H}_2$), 2.63 (3H, s, NC$\mathbf{H}_3$); ^{13}C NMR (100.6 MHz, $(CD_3)_2SO$) δ 171.2 (C), 166.0 (C), 150.0 (C), 147.8 (C), 146.4 (C), 141.4 (C), 139.6 (C), 139.4 (C), 136.1 (C), 133.7 (C), 130.3 (CH), 128.5 (2 × CH), 128.5 (2 × CH), 128.3 (CH), 128.3 (2 × CH), 126.8 (CH), 126.1 (2 × CH), 125.9 (CH), 122.1 (CH), 119.2 (CH_2), 117.8 (CH), 59.9 (C), 48.5 (CH), 33.8 (CH_2), 33.4 (CH_2), 27.9 (CH_3), 27.8 (CH_3); HRMS (ESI +ve) Exact mass calculated for $C_{31}H_{21}N_3NaO_5$ $[M+Na]^+$: 544.1843, found: 544.1870.

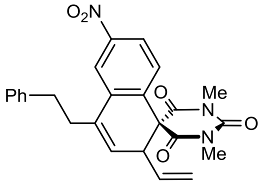

2'-Ethenyl-1,5-dimethyl-6'-nitro-4'-(2-phenylethyl)-2'*H*-spiro[1,5-diazinane-3,1'-naphthalene]-2,4,6-trione (3p). The title compound was prepared according to General Procedure A from 5-arylbarbituric acid **1b** (139 mg, 0.50 mmol) and 1,3-enyne **2g** (93.5 mg, 0.55 mmol) at 60 °C for a reaction time of 2 h and was purified by trituration with hot EtOH to give an off-white solid (160 mg, 72%). R_f 0.17 (2:1 petroleum ether:Et_2O); m.p. 199–200 ºC (CH_2Cl_2/petroleum ether); IR 1685, 1675, 1522 (NO_2), 1425, 1373 (NO_2), 1086, 1048, 745, 641 cm^{-1}; ^{1}H NMR (400 MHz, $CDCl_3$) δ 8.26 (1H, d, J = 2.3 Hz, Ar**H**), 8.04 (1H, dd, J = 8.5, 2.3 Hz, Ar**H**), 7.32 (4H, m, 4 × Ar**H**), 7.26–7.20 (1H, m, Ar**H**), 7.09 (1H, d, J = 8.5 Hz, Ar**H**), 5.75–5.63 (2H, m, C**H**=CH_2 and CH_2C=C**H**), 5.29–5.26 (1H, d, J = 10.2 Hz, CH=C$\mathbf{H}_\mathbf{A}$$H_B$), 5.25 (1H, d, J = 17.0 Hz, CH=$CH_A$$\mathbf{H}_\mathbf{B}$), 4.11–4.03 (1H, m, C=CHC**H**), 3.43 (3H, s, NC$\mathbf{H}_3$), 3.28 (3H, s, NC$\mathbf{H}_3$), 3.01–2.91 (2H, m, C$\mathbf{H}_2$), 2.91–2.79 (2H, m, C$\mathbf{H}_2$); ^{13}C NMR (100.6 MHz, $CDCl_3$) δ 171.2 (C), 166.3 (C), 150.6 (C), 148.3 (C), 141.2 (C), 138.7 (C), 136.9 (C), 134.7 (C), 133.6 (CH), 128.5 (2 × CH), 128.4 (2 × CH and CH), 126.1 (CH), 124.2 (CH), 122.4 (CH), 121.0 (CH_2), 118.8 (CH), 60.5 (C), 49.9 (CH), 34.3 (2 × CH_2), 29.1

(CH_3), 28.7 (CH_3); HRMS (ESI +ve) Exact mass calculated for $C_{25}H_{23}N_3NaO_5$ $[M+Na]^+$: 468.1530, found: 468.1543.

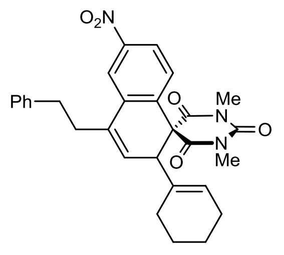

2'-(Cyclohex-1-en-1-yl)-1,5-dimethyl-6'-nitro-4'-(2-phenylethyl)-2'*H*-spiro[1,5-diazinane-3,1'-naphthalene]-2,4,6-trione (3q). The title compound was prepared according to General Procedure A from 5-arylbarbituric acid **1b** (139 mg, 0.50 mmol) and 1,3-enyne **2h** (123 mg, 0.55 mmol) at 60 °C for a reaction time of 2 h and was purified by flash column chromatography (3:1 to 3:2 petroleum ether:Et_2O) to give a yellow solid (215 mg, 86%). R_f 0.19 (2:1 petroleum ether:Et_2O); m.p. 104–105 ºC (CH_2Cl_2/petroleum ether); IR 1684 (C=O), 1523 (NO_2), 1347 (NO_2), 753, 641 cm^{-1}; ^1H NMR (400 MHz, $CDCl_3$) δ 8.27 (1H, d, J = 2.3 Hz, Ar**H**), 8.03 (1H, dd, J = 8.5, 2.4 Hz, Ar**H**), 7.36–7.28 (3H, m, 3 × Ar**H**), 7.31 (1H, s, Ar**H**), 7.22 (1H, ddd, J = 10.4, 4.9, 2.0 Hz, Ar**H**), 7.12 (1H, d, J = 8.5 Hz, Ar**H**), 5.64 (1H, d, J = 3.4 Hz, $PhCH_2CH_2C$=C**H**), 5.58–5.52 (1H, m, =C**H**CH_2), 3.92–3.88 (1H, m, C=CHC**H**), 3.40 (3H, s, NC**H**$_3$), 3.26 (3H, s, NC**H**$_3$), 3.03–2.77 (4H, m, 2 × C**H**$_2$), 2.10–1.92 (2H, m, C**H**$_2$), 1.88–1.41 (6H, m, 3 × C**H**$_2$); ^{13}C NMR (100.6 MHz, $CDCl_3$) δ 171.2 (C), 166.7 (C), 150.7 (C), 148.1 (C), 141.2 (C), 138.8 (C), 137.2 (C), 135.1 (C), 134.6 (C), 128.9 (CH), 128.8 (CH), 128.4 (2 × CH), 128.4 (2 × CH), 126.0 (CH), 124.1 (CH), 122.1 (CH), 118.4 (CH), 61.2 (C), 54.5 (CH), 34.2 (CH_2), 34.1 (CH_2), 29.0 (CH_3), 28.8 (CH_3), 27.0 (CH_2), 25.5 (CH_2), 22.6 (CH_2), 21.7 (CH_2); HRMS (ESI +ve) Exact mass calculated for $C_{29}H_{30}N_3O_5$ $[M+H]^+$: 500.2180, found: 500.2180.

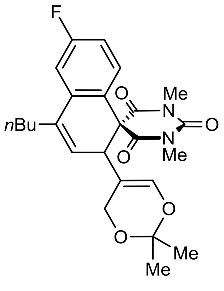

4'-Butyl-2'-(2,2-dimethyl-2,4-dihydro-1,3-dioxin-5-yl)-6'-fluoro-1,5-dimethyl-2'*H*-spiro[1,5-diazinane-3,1'-naphthalene]-2,4,6-trione (3r). The title compound was prepared according to General Procedure A from 5-arylbarbituric acid **1e** (125 mg, 0.50 mmol) and 1,3-enyne **2i** (114 mg, 0.55 mmol) at 60 °C for a reaction time of 2 h and was purified by flash column chromatography (3:1 to 2:1 petroleum ether:Et_2O) to give a colorless oil (130 mg, 57%). R_f 0.23 (2:1 petroleum ether:Et_2O); IR 1680 (C=O), 1370, 1239, 754, 611 cm^{-1}; ^1H NMR (400 MHz, $CDCl_3$) δ 7.11 (1H, dd, J = 10.5, 2.5 Hz, Ar**H**), 6.93 (1H, dd, J = 8.5, 5.5 Hz, Ar**H**), 6.88 (1H, td, J = 8.5, 2.5 Hz, Ar**H**), 6.31 (1H, t, J = 1.0 Hz, OC**H**=), 5.53 (1H, d, J = 4.5 Hz, CH_2C=C**H**), 3.84 (2H, d, J = 1.0 Hz, OC**H**$_2$), 3.70 (1H, d, J = 4.5 Hz, C=CHC**H**), 3.36 (3H, s, NC**H**$_3$), 3.30 (3H, s, NC**H**$_3$), 2.51–2.39 (2H, m, C**H**$_2CH_2CH_2CH_3$), 1.63–1.50 (2H, m, C**H**$_2CH_2CH_3$), 1.50–1.39 (2H, m, C**H**$_2CH_3$), 1.37 (3H, s, C(C**H**$_3$)$_2$), 1.36 (3H, s, C(C**H**$_3$)$_2$), 0.96 (3H, t, J = 7.3 Hz, CH_2C**H**$_3$); ^{13}C NMR (100.6 MHz, $CDCl_3$) δ 170.5 (C), 168.6 (C), 162.9 (d, J =

247.0 Hz, C), 150.8 (C), 140.2 (2 × CH), 137.6 (d, J = 8.0 Hz, C), 136.7 (d, J = 1.8 Hz, C), 129.1 (d, J = 8.5 Hz, CH), 126.7 (d, J = 3.3 Hz, C), 121.3 (2 × CH), 114.1 (d, J = 21.8 Hz, CH), 111.4 (d, J = 23.0 Hz, CH), 108.0 (C), 99.1 (C), 60.6 (C), 58.8 (CH_2), 48.4 (CH), 32.2 (CH_2), 30.2 (CH_2), 29.2 (CH_3), 28.9 (CH_3), 25.4 (CH_3), 22.9 (CH_3), 22.5 (CH_2), 13.9 (CH_3); ^{19}F NMR (376 MHz, $CDCl_3$) δ –112.4 (m); HRMS (ESI +ve) Exact mass calculated for $C_{25}H_{29}FN_2NaO_5$ $[M+Na]^+$: 479.1953, found: 479.1977.

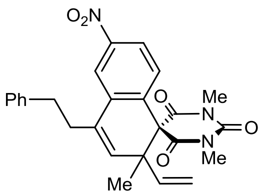

2'Ethenyl-1,2',5-trimethyl-6'-nitro-4'-(2-phenylethyl)-2'*H*-spiro[1,5-diazinane-3,1'-naphthalene]-2,4,6-trione (3s). The title compound was prepared according to General Procedure A from 5-arylbarbituric acid **1b** (139 mg, 0.50 mmol) and 1,3-enyne **2j** (101 mg, 0.55 mmol) at 60 °C for a reaction time of 2 h and was purified by flash column chromatography (2:1 to 1:1 petroleum ether:Et_2O) to give an off-white solid (171 mg, 75%). R_f 0.31 (1:1 petroleum ether:Et_2O); m.p. 145–146 ºC (CH_2Cl_2/petroleum ether); IR 1678 (C=O), 1523, 1451, 1367, 1118, 908, 738, 641 cm^{-1}; ^{1}H NMR (400 MHz, $CDCl_3$) δ 8.27 (1H, d, J = 2.4 Hz, Ar**H**), 8.05 (1H, dd, J = 8.6, 2.4 Hz, Ar**H**), 7.35–7.27 (4H, m, 4 × Ar**H**), 7.25–7.20 (1H, m, Ar**H**), 7.18 (1H, d, J = 8.6 Hz, Ar**H**), 5.76 (1H, dd, J = 17.3, 10.7 Hz, C**H**=CH_2), 5.44 (1H, s, CH_2C=C**H**), 5.20 (1H, d, J = 10.7 Hz, =C$\mathbf{H_A}$$H_B$), 5.11 (1H, d, J = 17.3 Hz, =C$H_A$$\mathbf{H_B}$), 3.39 (3H, s, NC$\mathbf{H_3}$), 3.26 (3H, s, NC$\mathbf{H_3}$), 3.00–2.92 (2H, m, C$\mathbf{H_2}$), 2.92–2.83 (2H, m, C$\mathbf{H_2}$), 1.17 (3H, s, CC$\mathbf{H_3}$); ^{13}C NMR (100.6 MHz, $CDCl_3$) δ 167.4 (C), 166.5 (C), 150.7 (C), 148.1 (C), 141.1 (C), 138.9 (CH), 137.8 (C), 136.8 (C), 133.6 (C), 130.2 (CH), 128.8 (CH), 128.4 (2 × CH), 128.4 (2 × CH), 126.0 (CH), 122.1 (CH), 118.3 (CH), 116.8 (CH_2), 64.7 (C), 47.4 (C), 34.2 (CH_2), 34.1 (CH_2), 28.8 (CH_3), 28.6 (CH_3), 22.2 (CH_3); HRMS (ESI +ve) Exact mass calculated for $C_{26}H_{26}N_3O_5$ $[M+H]^+$: 460.1867, found: 460.1876.

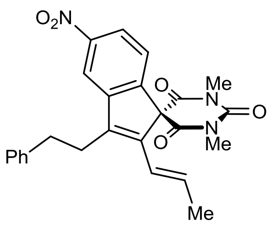

3'-Butyl-1,5-dimethyl-5'-nitro-2'-[(*E*)-prop-1-en-1-yl]spiro[1,5-diazinane-3,1'-indene]-2,4,6-trione (4b). The title compound was prepared according to General Procedure A from 5-arylbarbituric acid **1b** (139 mg, 0.50 mmol) and 1,3-enyne **9** (93.5 mg, 0.55 mmol) at 60 °C for a reaction time of 2 h and was purified by flash column chromatography (2:1 to 1:1 petroleum ether:Et_2O) to give an off-white solid (182 mg, 82%). R_f 0.26 (1:1 petroleum ether:Et_2O); m.p. 217–218 ºC (CH_2Cl_2/petroleum ether); IR 1681 (C=O), 1525, 1442, 1345, 1050, 899, 641 cm^{-1}; ^{1}H NMR (400 MHz, $CDCl_3$) δ 7.98 (1H, dd, J = 8.2, 2.0 Hz, Ar**H**), 7.81 (1H, d, J = 2.0 Hz, Ar**H**), 7.31–7.23 (4H, m, 4 × Ar**H**), 7.22–7.14 (2H, m, 2 × Ar**H**), 6.35 (1H, dq, J = 15.7, 1.5 Hz, C**H**=CHCH_3), 5.29 (1H, dq, J = 15.7, 6.7 Hz, CH=C**H**CH_3), 3.45 (6H, s, 2 × NC$\mathbf{H_3}$), 3.07–2.93

(4H, m, 2 × C**H**$_2$), 1.84 (3H, dd, *J* = 6.7, 1.5 Hz, CH=CHC**H**$_3$); ^{13}C NMR (100.6 MHz, $CDCl_3$) δ 165.2 (2 × C), 150.9 (C), 149.2 (C), 147.8 (C), 147.7 (C), 142.7 (C), 142.0 (C), 140.4 (C), 129.5 (CH), 128.7 (2 × CH), 128.4 (2 × CH), 126.2 (CH), 122.3 (CH), 121.4 (CH), 120.3 (CH), 115.3 (CH), 66.3 (C), 34.9 (CH_2), 29.5 (2 × CH_3), 27.5 (CH_2), 19.2 (CH_3); HRMS (ESI +ve) Exact mass calculated for $C_{25}H_{24}N_3O_5$ $[M+H]^+$: 446.1710, found: 446.1731.

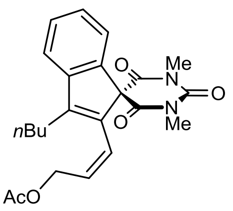

(*Z*)-3-{3'-Butyl-1,5-dimethyl-2,4,6-trioxospiro[1,5-diazinane-3,1'-indene]-2'-yl}prop-2-en-1-yl acetate (4c). The title compound was prepared according to General Procedure A from 5-arylbarbituric acid **1a** (116 mg, 0.50 mmol) and 1,3-enyne **2k** (99.1 mg, 0.55 mmol) at 60 °C for a reaction time of 2 h and was purified by flash column chromatography (2:1 to 1:1 petroleum ether:Et_2O) to give an off-white solid (178 mg, 87%). R_f 0.14 (2:1 petroleum ether:Et_2O); m.p. 101–102 ºC (CH_2Cl_2/petroleum ether); IR 1733 (C=O), 1672 (C=O), 1437, 1364, 1238, 1027, 758, 641 cm^{-1}; ^{1}H NMR (400 MHz, $CDCl_3$) δ 7.41–7.36 (2H, m, 2 × Ar**H**), 7.22–7.15 (1H, m, Ar**H**), 7.15–7.11 (1H, m, Ar**H**), 6.14 (1H, dt, *J* = 11.6, 1.4 Hz, C**H**=CHCH$_2$OAc), 5.88 (1H, dt, *J* = 11.6, 6.8 Hz, CH=C**H**CH$_2$OAc), 4.65 (1H, dd, *J* = 6.8, 1.4 Hz, CH=CHC**H**$_2$OAc), 3.36 (6H, s, 2 × NC**H**$_3$), 2.58 (2H, t, *J* = 7.6 Hz, C**H**$_2$CH$_2$CH$_2$CH$_3$), 2.05 (3H, s, COC**H**$_3$), 1.69–1.59 (2H, m, C**H**$_2$CH$_2$CH$_3$), 1.46–1.36 (2H, m, C**H**$_2$CH$_3$), 0.93 (3H, t, *J* = 7.3 Hz, C**H**$_3$); ^{13}C NMR (100.6 MHz, $CDCl_3$) δ 170.7 (C), 166.2 (2 × C), 151.5 (C), 147.7 (C), 144.7 (C), 142.1 (C), 135.5 (C), 129.7 (CH), 129.4 (CH), 126.5 (CH), 123.9 (CH), 121.5 (CH), 120.6 (CH), 69.1 (C), 61.7 (CH_2), 29.9 (CH_2), 29.2 (2 × CH_3), 25.7 (CH_2), 22.4 (CH_2), 20.7 (CH_3), 13.8 (CH_3); HRMS (ESI +ve) Exact mass calculated for $C_{23}H_{27}N_2O_5$ $[M+H]^+$: 411.1914, found: 411.1910.

4. Deuterium Labeling Experiments

1,5-Dimethyl-6'-nitro-4'-(2-phenylethyl)-2'-[(^{2}H$_5$)prop-1-en-2-yl](3'-^{2}H)-2'*H*-spiro[1,5-diazinane-3,1'-naphthalene]-2,4,6-trione ([D$_6$]-3n)

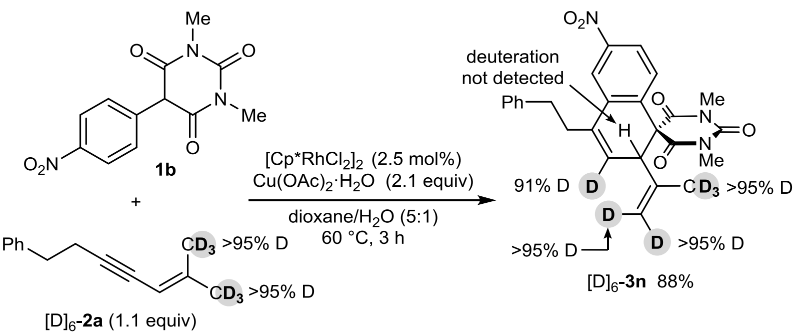

The title compound was prepared according to General Procedure A from 5-arylbarbituric acid **1b** (139 mg, 0.50 mmol) and 1,3-enyne [D$_6$]-**2a** (105 mg, 0.55 mmol) at 60 °C for a reaction time of 3 h and and was purified by flash column chromatography (2:1 to 1:1 petroleum ether:Et$_2$O) to give a yellow solid (205 mg, 88%). R$_f$ 0.31 (1:1 petroleum ether:Et$_2$O); m.p. 177–178 °C (CH$_2$Cl$_2$/petroleum ether); IR 1684 (C=O), 1521, 1374, 1346, 1295, 911, 640 cm^{-1}; ^{1}H NMR (400 MHz, CDCl$_3$) δ 8.28 (1H, d, *J* = 2.4 Hz, Ar**H**), 8.05 (1H, dd, *J* = 8.5, 2.4 Hz, Ar**H**), 7.36–7.29 (4H, m, 4 × Ar**H**), 7.25–7.21 (1H, m, Ar**H**), 7.12 (1H, d, *J* = 8.5 Hz, Ar**H**), 5.65 (0.09H, d, *J* = 3.5 Hz, CH$_2$C=C**H**), 4.05 (1H, t, *J* = 1.7 Hz, C=CHC**H**), 3.41 (3H, s, NC**H**$_3$), 3.26 (3H, s, NC**H**$_3$), 3.01–2.93 (2H, m, PhC**H**$_2$CH$_2$), 2.93–2.85 (2H, m, PhCH$_2$C**H**$_2$); ^{13}C NMR (100.6 MHz, CDCl$_3$) δ 171.0 (C), 166.7 (C), 150.6 (C), 148.2 (C), 142.0 (C), 141.1 (C), 138.8 (C), 137.1 (C), 134.9 (C), 128.7 (CH), 128.4 [2 × (2 × CH)], 126.1 (CH), 124.0 (CH – smaller peak as carbon is 91% deuterated), 122.3 (CH), 118.5 (CH), 60.7 (C), 53.2 (CH), 34.2 (CH$_2$), 34.0 (CH$_2$), 29.1 (CH$_3$), 28.9 (CH$_3$); HRMS (ESI +ve) Exact mass calculated for $C_{26}H_{30}D_6N_3O_5$ [M+H]$^+$: 466.2244, found: 466.2259.

(Expansion of aromatic and alkenyl regions)

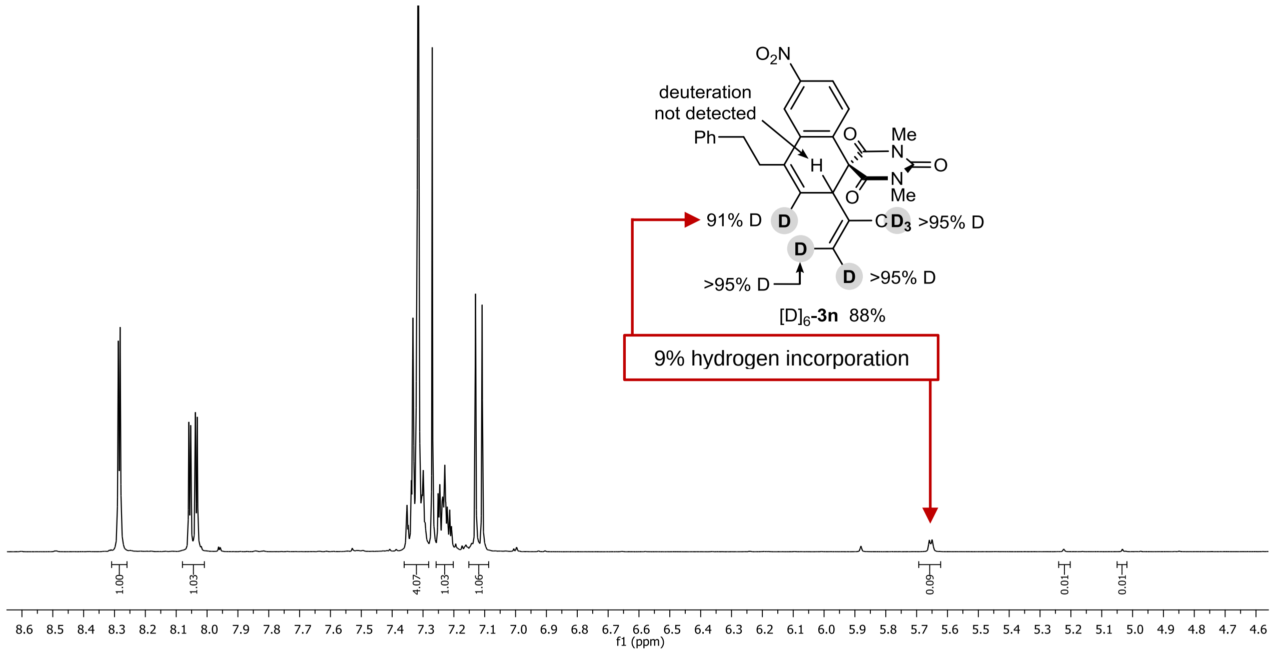

1,5-Dimethyl-4'-(2-phenylethyl)-2'-(prop-1-en-2-yl)(3'-^{2}H)-2'*H*-spiro[1,5-diazinane-3,1'-naphthalene]-2,4,6-trione ([D$_n$]-3n)

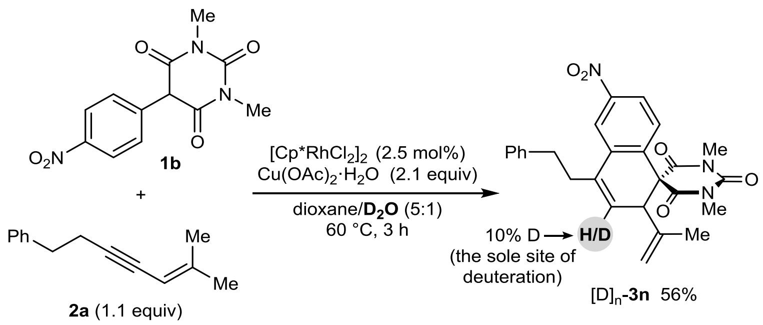

The title compound was prepared according to a modification of General Procedure A (in that 0.25 mmol of **1b** and D_2O were used) from 5-arylbarbituric acid **1b** (69.3 mg, 0.25 mmol) and 1,3-enyne **2a** (50.6 mg, 0.28 mmol) at 60 °C for a reaction time of 3 h and and was purified by flash column chromatography (2:1 to 1:1 petroleum ether:Et_2O) to give a yellow solid (64 mg, 56%).

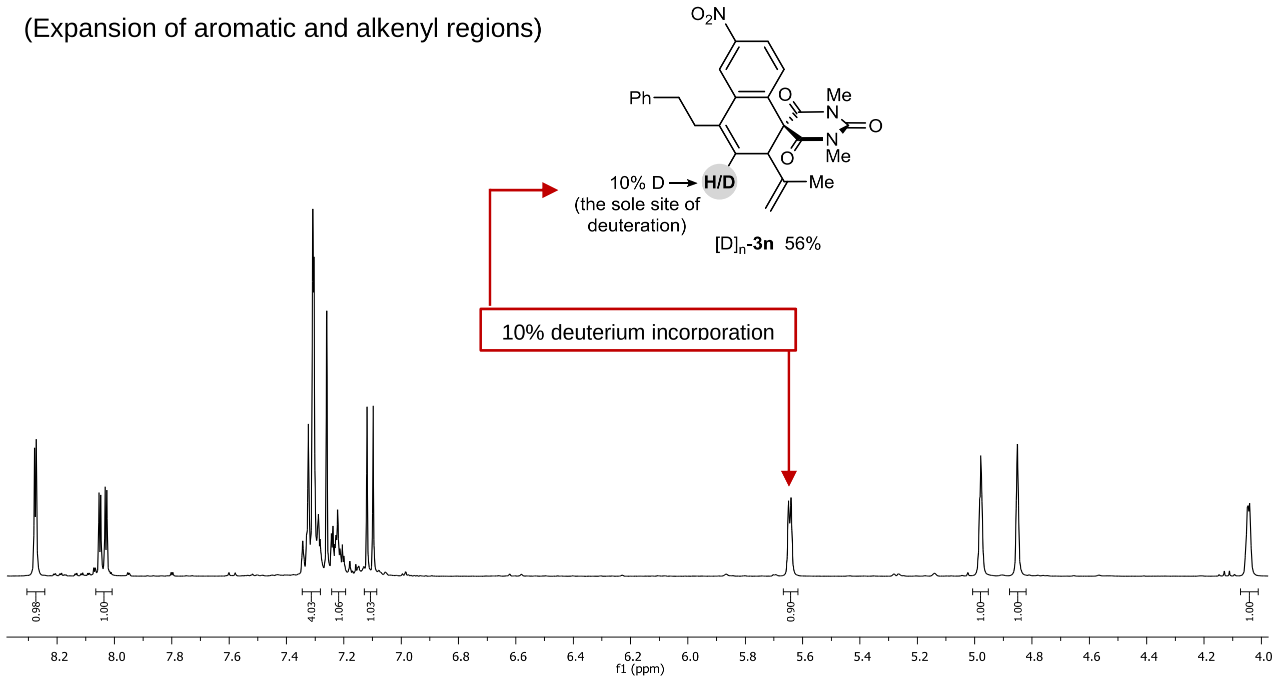

Conversion of Spirodialin 3o into Naphthalene 20

1,3-Dimethyl-1-[2-(1-phenylethenyl)-4-(2-phenylethyl)naphthalene-1-carbonyl]urea (20)

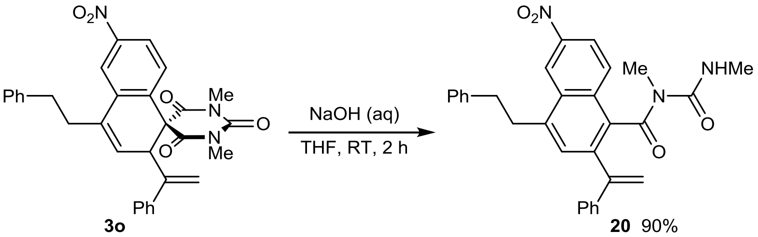

To a solution of spirodialin **3o** (52.1 mg, 0.10 mmol) in THF (2 mL) was added 2.0 M $NaOH_{(aq)}$ (4 mL) and the mixture was stirred at room temperature for 2 h. The reaction was quenched with 3.0 M $HCl_{(aq)}$ (10 mL) and EtOAc (10 mL) was then added. The layers were separated and the aqueous layer was further extracted with EtOAc (2 × 10 mL). The organic layers were combined, dried ($MgSO_4$), and concentrated *in vacuo*. Purification of the residue by flash column chromatography (Et_2O) gave *naphthalene* **20** (44.3 mg, 90%) as a pale yellow oil. R_f 0.33 (1:1 petroleum ether:EtOAc); IR 3312 (NH), 1708 (C=O), 1650 (NH), 1533 (NO_2), 1497, 1341 (NO_2), 1231, 988, 906, 729 cm^{-1}; ^{1}H NMR (400 MHz, $CDCl_3$) δ 9.07 (1H, d, *J* = 2.2 Hz, Ar**H**), 8.85 (1H, q, *J* = 4.7 Hz, N**H**), 8.31 (1H, dd, *J* = 9.2, 2.2 Hz, Ar**H**), 7.85 (1H, d, *J* = 9.2 Hz, Ar**H**), 7.35–7.29 (4H, m, 4 × Ar**H**), 7.29–7.25 (2H, m, 2 × Ar**H**), 7.25–7.18 (3H, m, 3 × Ar**H**), 7.17–7.11 (2H, m, 2 × Ar**H**), 5.79 (1H, s, C$\mathbf{H}_A$$H_B$=), 5.36 (1H, s, C$H_A$$\mathbf{H}_B$=), 3.57–3.42 (2H, m, C$\mathbf{H}_2$), 3.10 (2H, t, *J* = 7.4 Hz, C$\mathbf{H}_2$), 2.92 (3H, s, NC$\mathbf{H}_3$), 2.89 (3H, d, *J* = 4.7 Hz, NHC$\mathbf{H}_3$); ^{13}C NMR (100.6 MHz, $CDCl_3$) δ 172.5 (C), 154.9 (C), 147.4 (C), 145.7 (C), 141.7 (C), 140.3 (C), 139.2 (2 × C), 132.0 (C), 131.5 (C), 130.3 (C), 130.1 (CH), 128.5 (2 × CH), 128.44 (2 × CH), 128.41 (CH), 128.3 (2 × CH), 127.5 (2 × CH), 127.1 (CH), 126.4 (CH), 120.9 (CH), 120.6 (CH), 118.3 (CH_2), 36.9 (CH_2), 34.8 (CH_2), 33.7 (CH_3), 27.0 (CH_3); HRMS (ESI +ve) Exact mass calculated for $C_{30}H_{27}N_3NaO_4$ $[M+Na]^+$: 516.1894, found 516.1909.

5. NMR Spectra

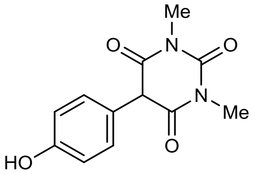

S1

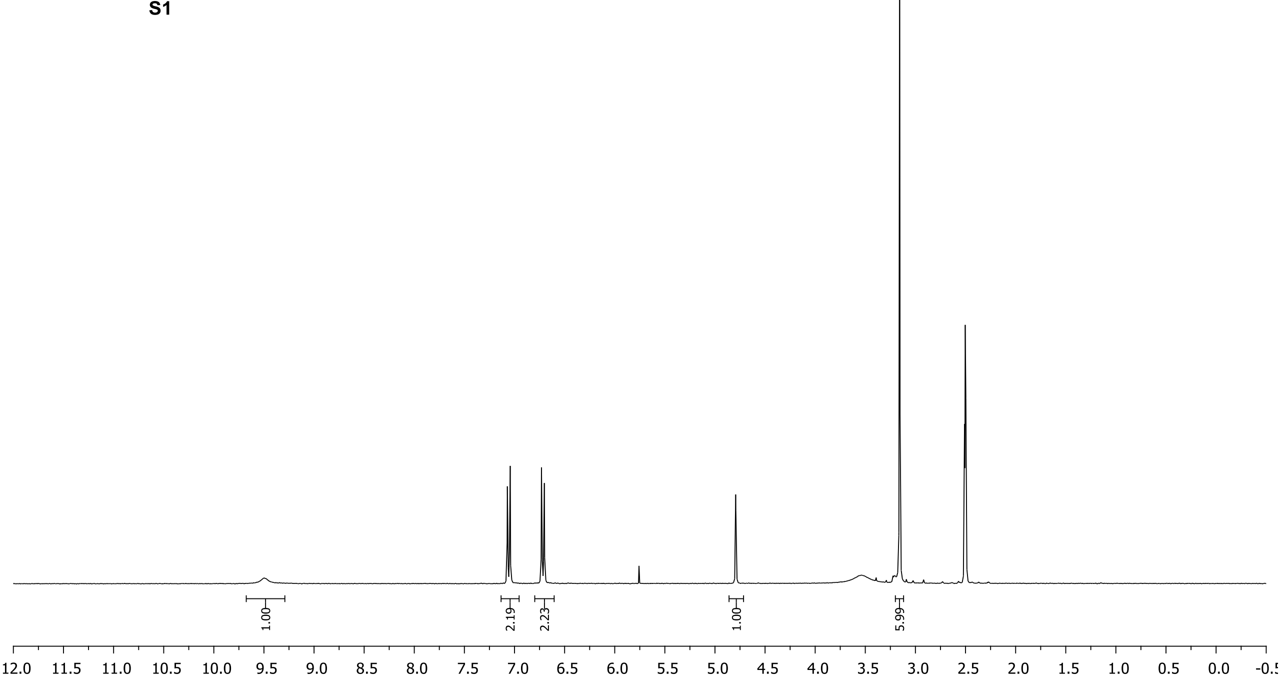

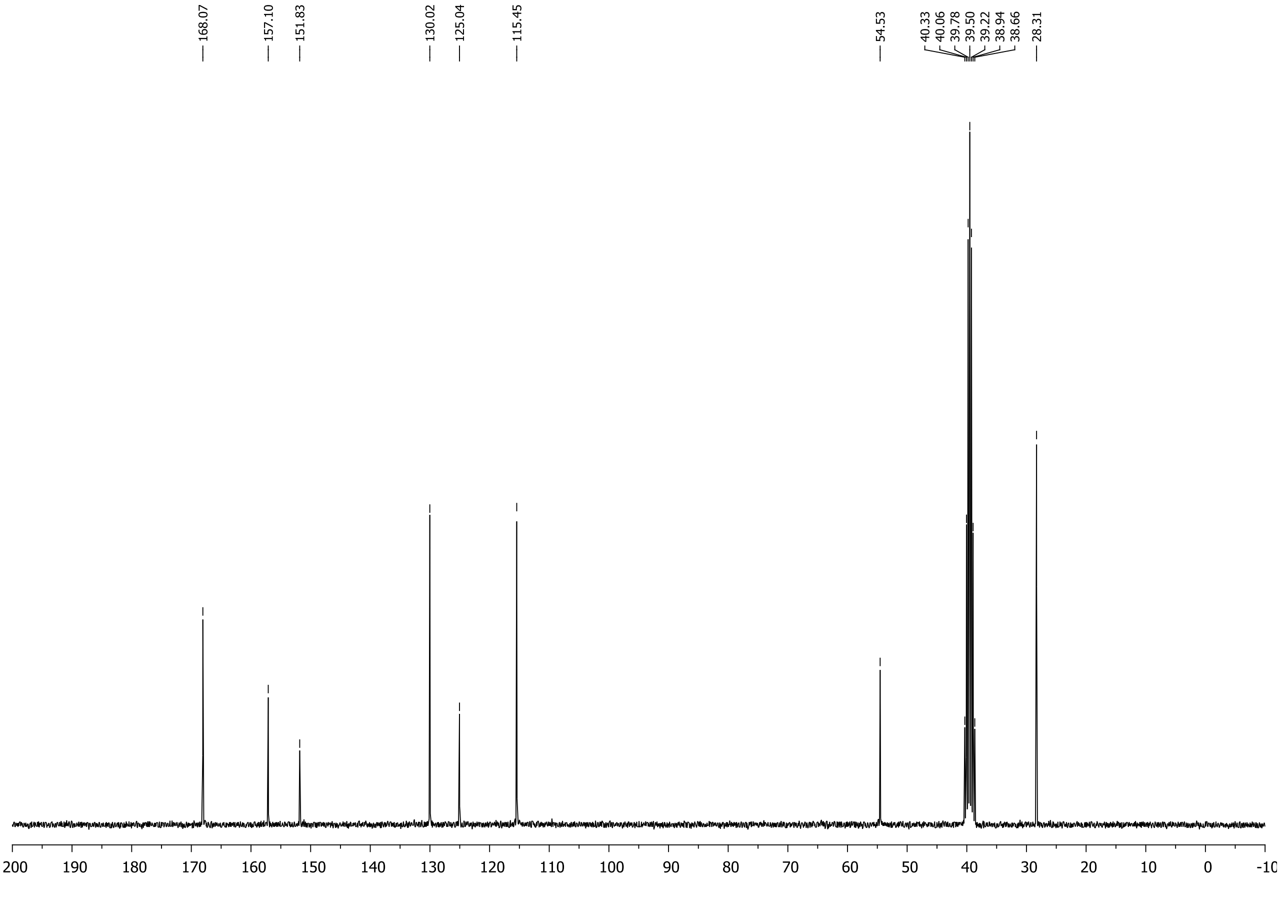

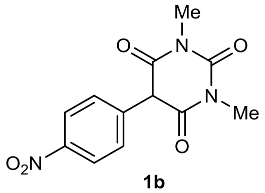
Me
O
N
O
N
Me
O
O2N

1b

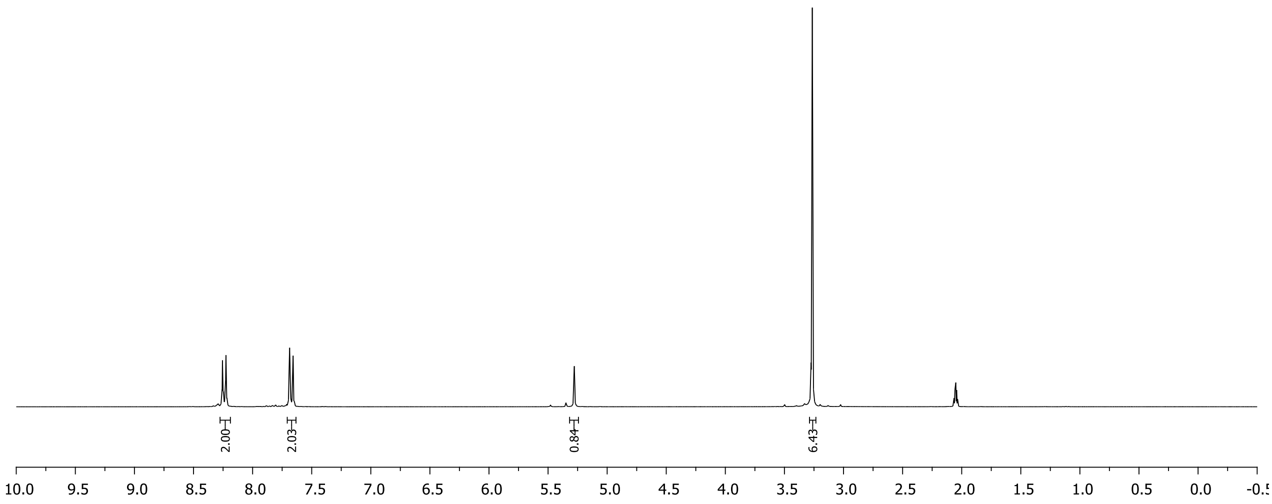
2.00
2.03
0.84
6.43
10.0 9.5 9.0 8.5 8.0 7.5 7.0 6.5 6.0 5.5 5.0 4.5 4.0 3.5 3.0 2.5 2.0 1.5 1.0 0.5 0.0 -0.5

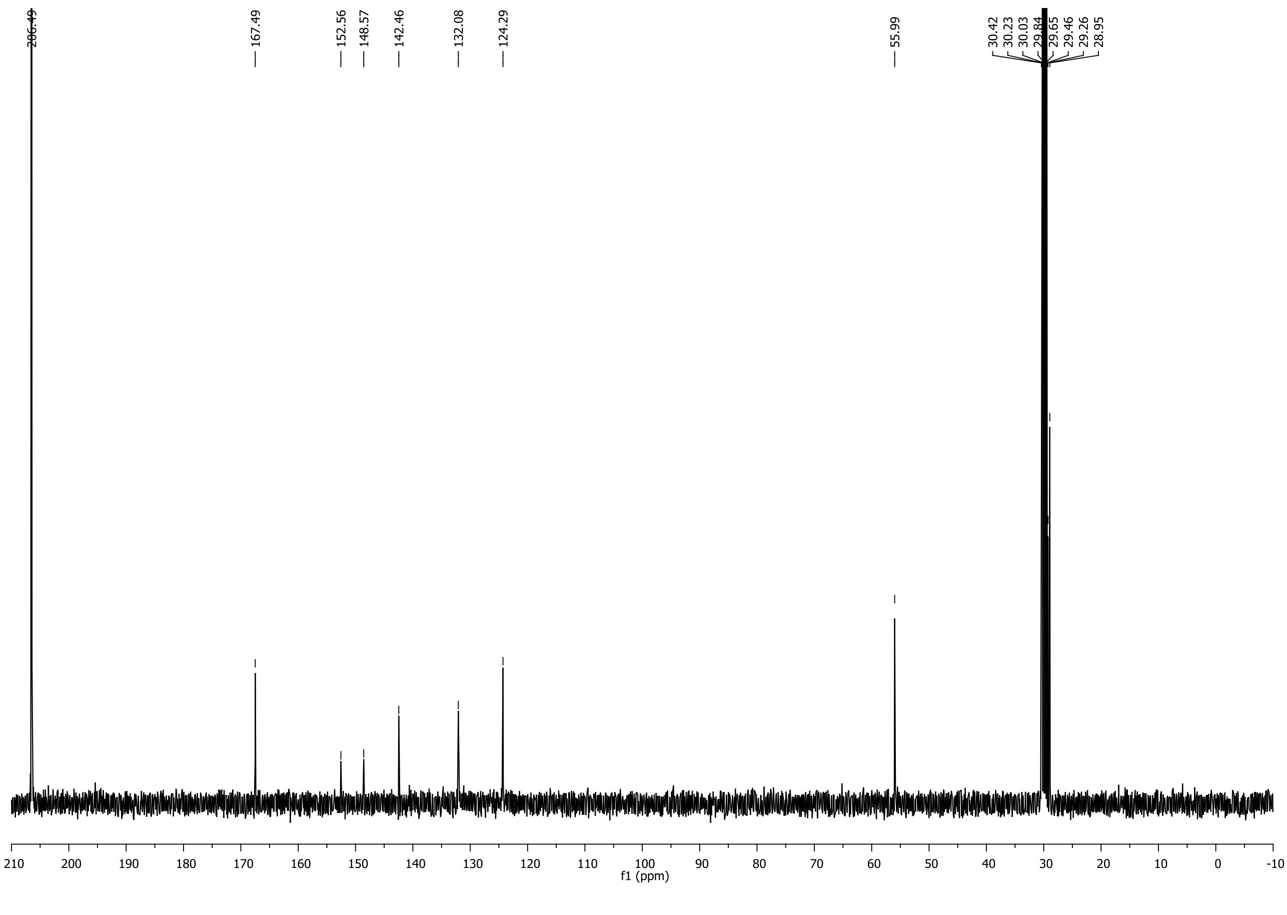
206.49
167.49
152.56
148.57
142.46
132.08
124.29
55.99
30.42
30.23
30.03
29.84
29.65
29.46
29.26
28.95
210 200 190 180 170 160 150 140 130 120 110 100 90 80 70 60 50 40 30 20 10 0 -10
f1 (ppm)

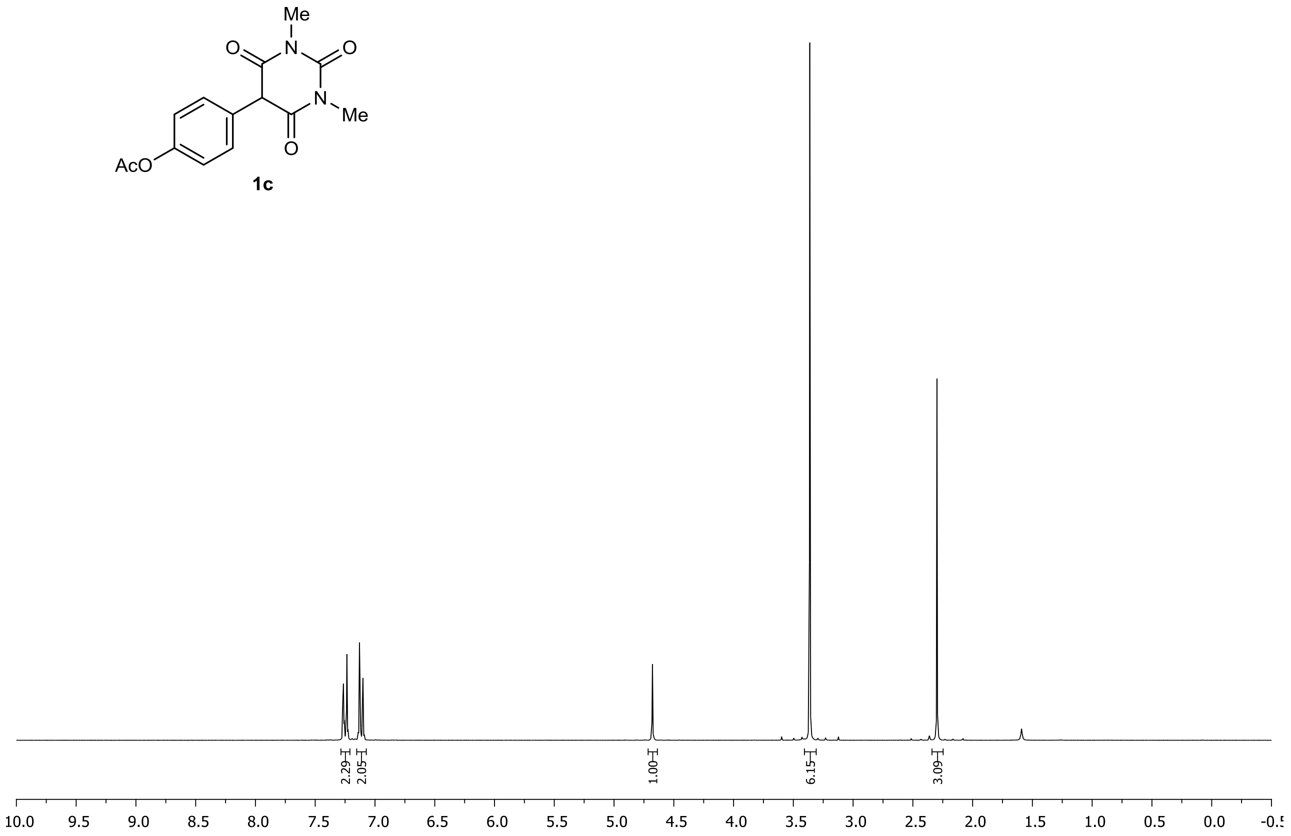
Me
O
N
O
N
Me
O
AcO
1c
2.29
2.05
1.00
6.15
3.09
10.0 9.5 9.0 8.5 8.0 7.5 7.0 6.5 6.0 5.5 5.0 4.5 4.0 3.5 3.0 2.5 2.0 1.5 1.0 0.5 0.0

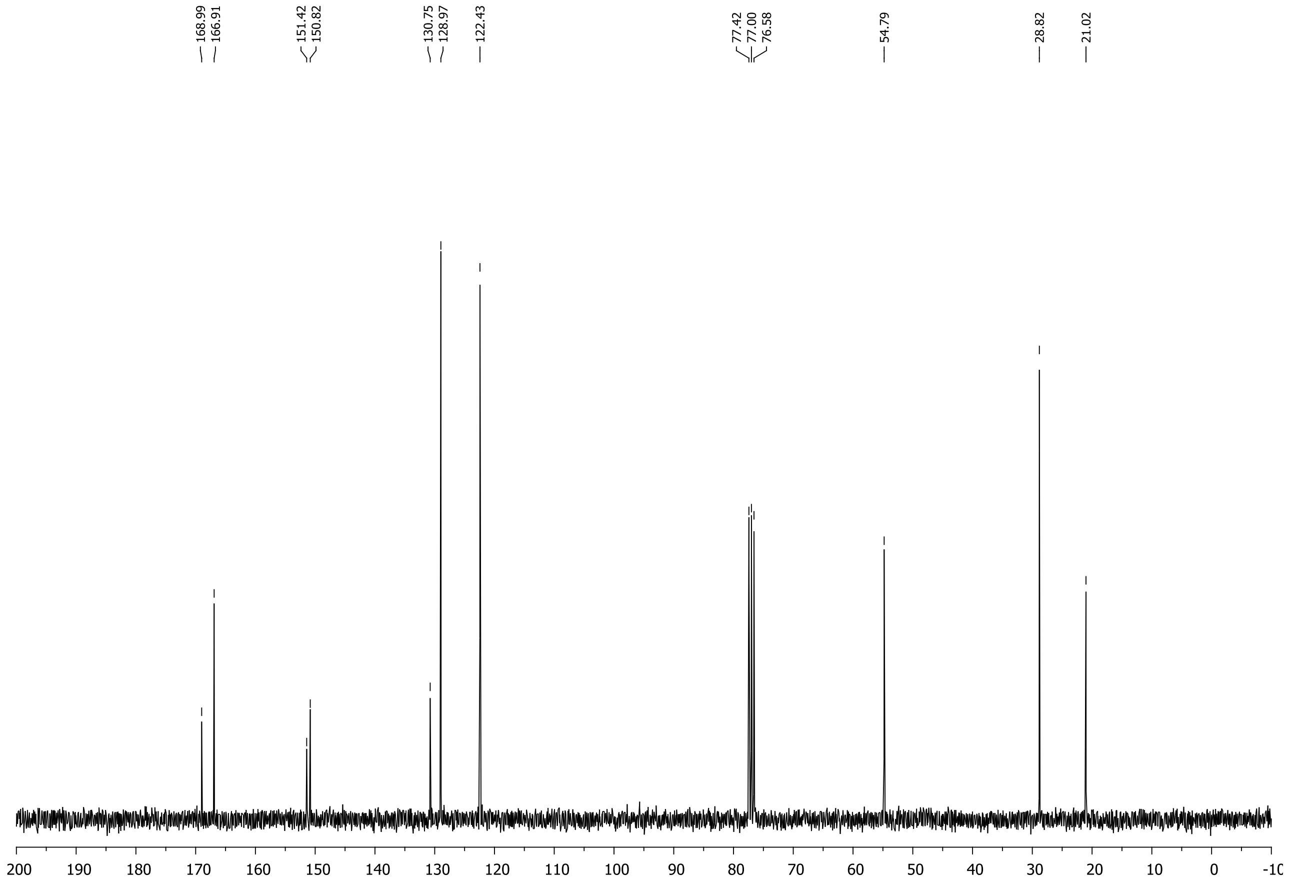
168.99
166.91
151.42
150.82
130.75
128.97
122.43
77.42
77.00
76.58
54.79
28.82
21.02
200 190 180 170 160 150 140 130 120 110 100 90 80 70 60 50 40 30 20 10 0

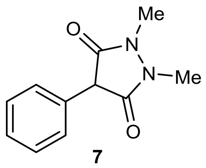
O
Me
N
N
Me
O

7

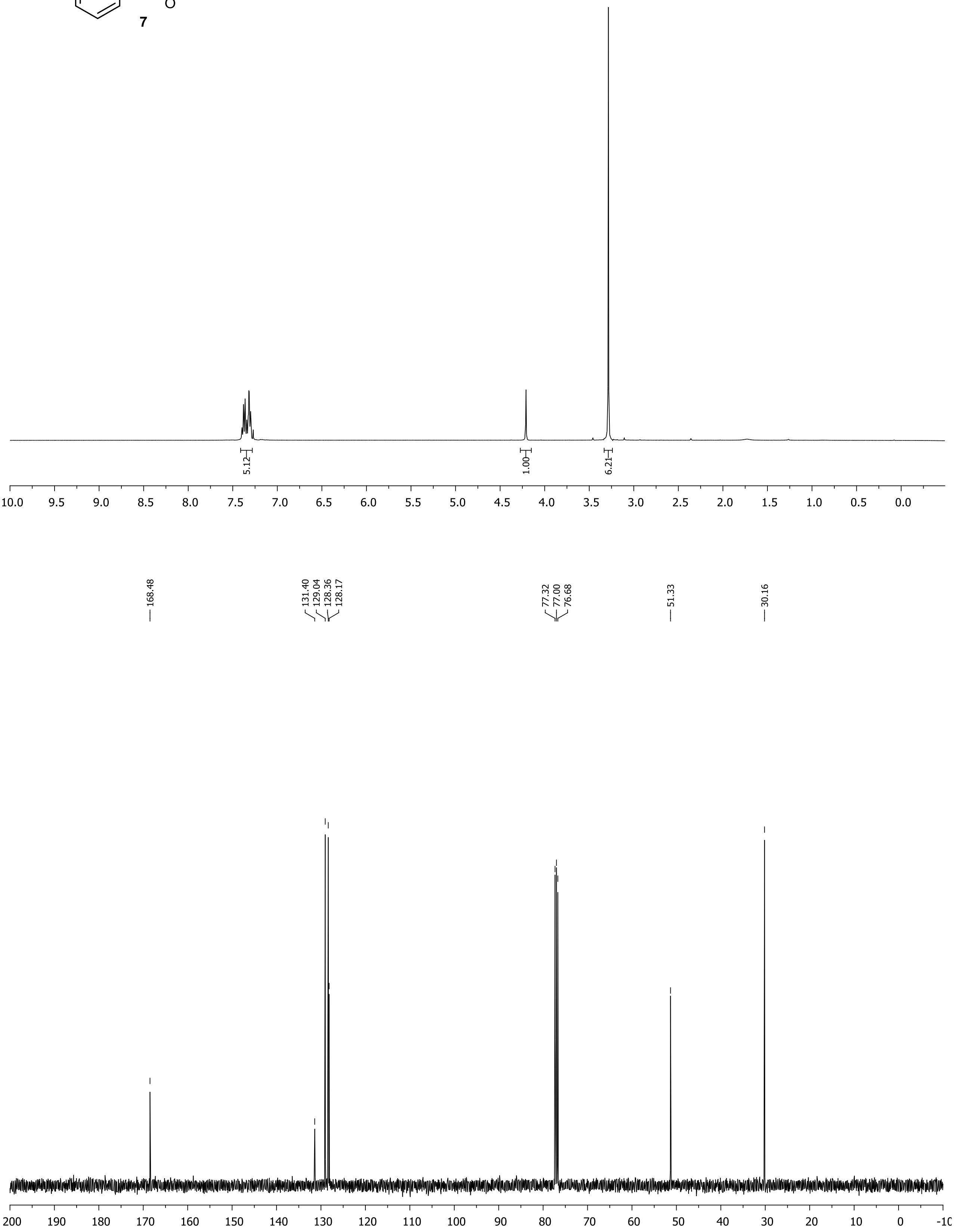
5.12
1.00
6.21
10.0 9.5 9.0 8.5 8.0 7.5 7.0 6.5 6.0 5.5 5.0 4.5 4.0 3.5 3.0 2.5 2.0 1.5 1.0 0.5 0.0
168.48
131.40
129.04
128.36
128.17
77.32
77.00
76.68
51.33
30.16
200 190 180 170 160 150 140 130 120 110 100 90 80 70 60 50 40 30 20 10 0 -10

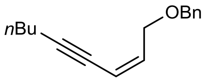
nBu
OBn

2b

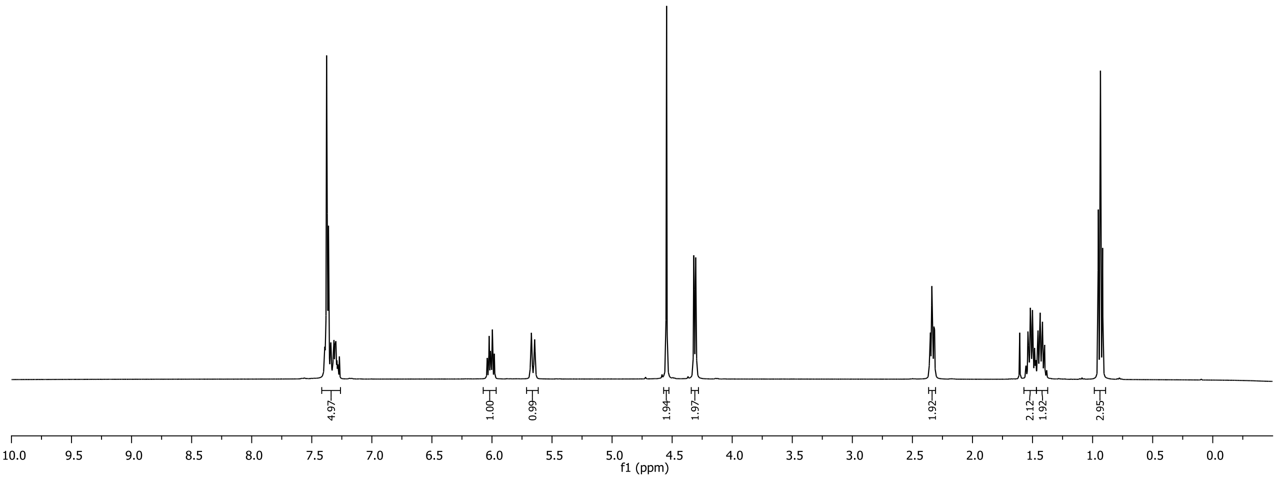
4.97
1.00
0.99
1.94
1.97
1.92
2.12
1.92
2.95
f1 (ppm)

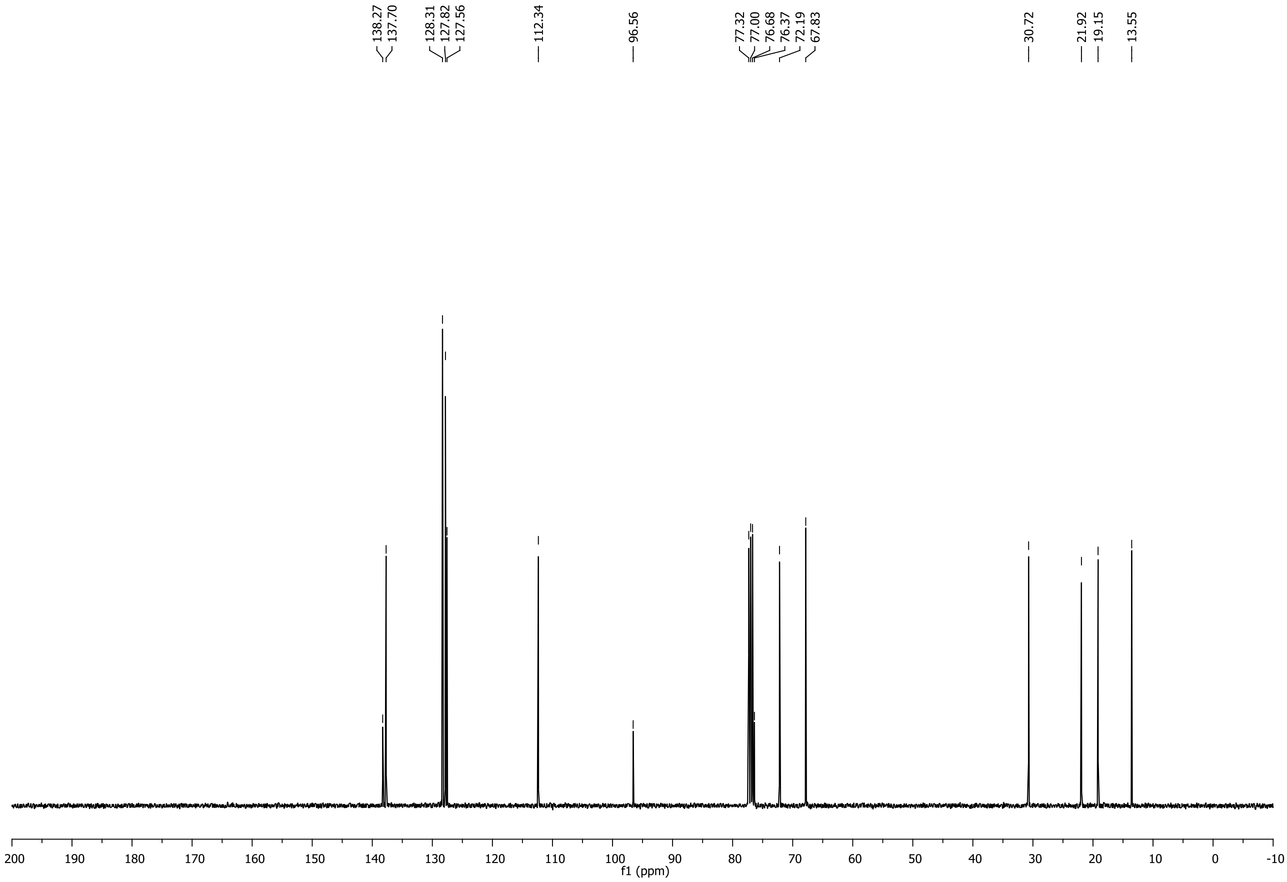
138.27
137.70
128.31
127.82
127.56
112.34
96.56
77.32
77.00
76.68
76.37
72.19
67.83
30.72
21.92
19.15
13.55
f1 (ppm)

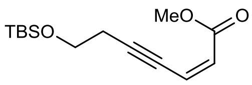
TBSO
MeO
O

S5

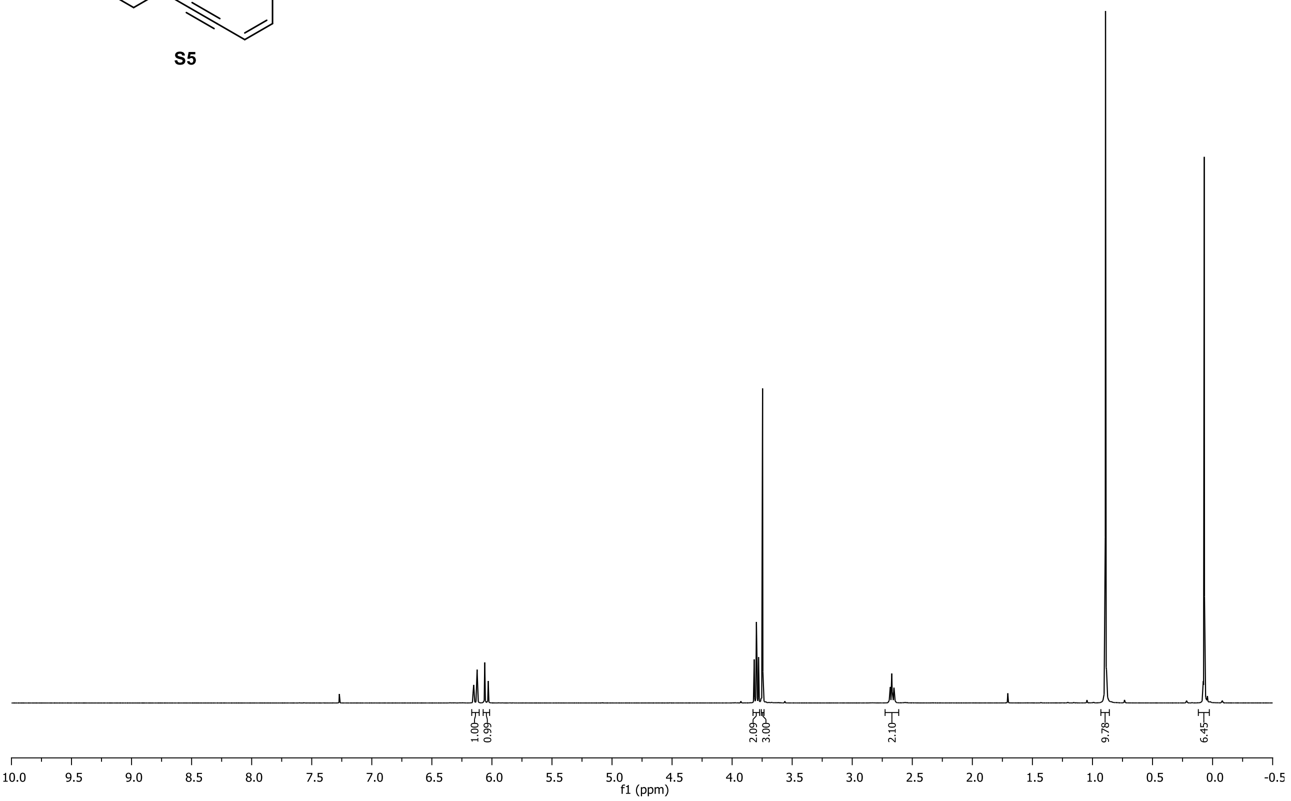
1.00
0.99
2.09
3.00
2.10
9.78
6.45
10.0 9.5 9.0 8.5 8.0 7.5 7.0 6.5 6.0 5.5 5.0 4.5 4.0 3.5 3.0 2.5 2.0 1.5 1.0 0.5 0.0 -0.5
f1 (ppm)

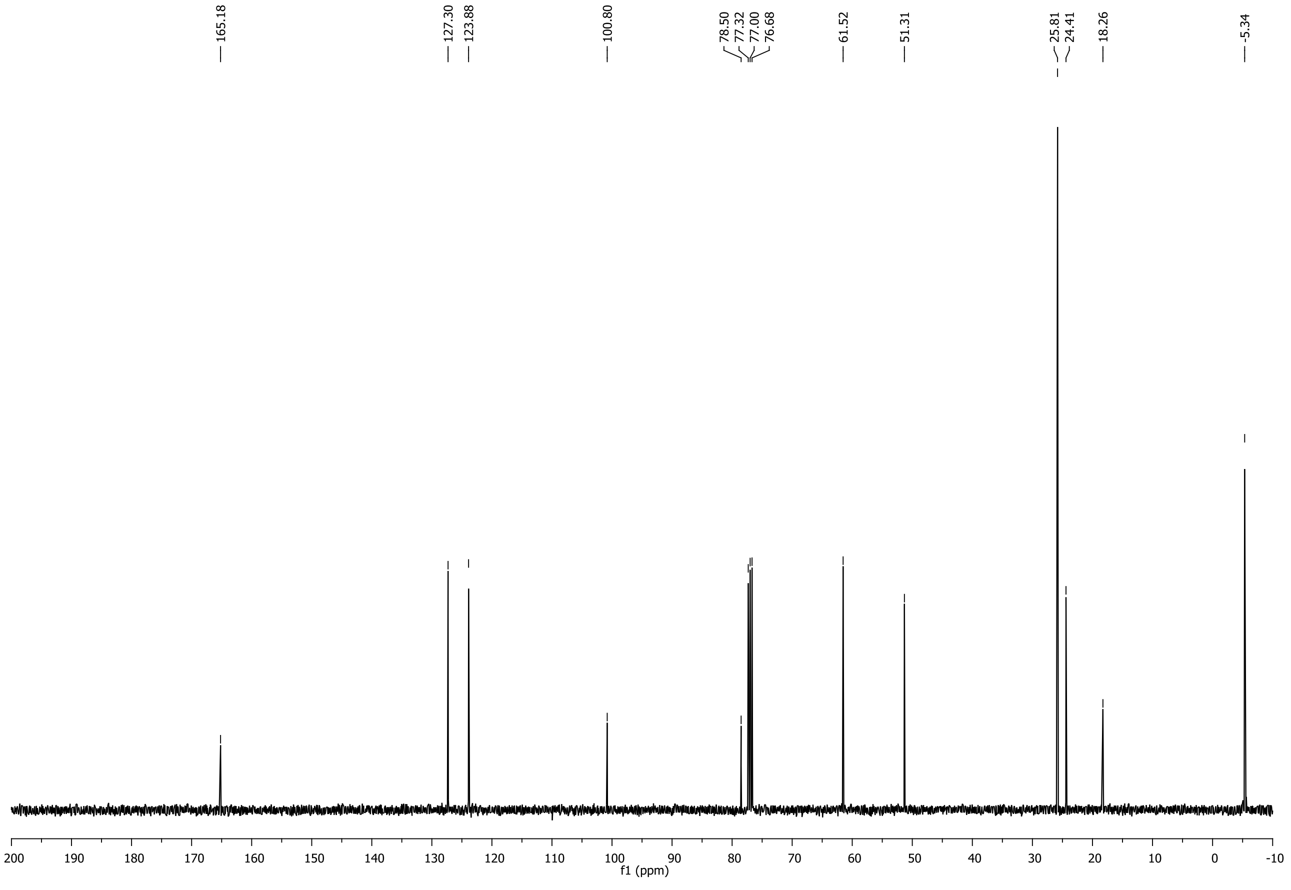
165.18
127.30
123.88
100.80
78.50
77.32
77.00
76.68
61.52
51.31
25.81
24.41
18.26
-5.34
200 190 180 170 160 150 140 130 120 110 100 90 80 70 60 50 40 30 20 10 0 -10
f1 (ppm)

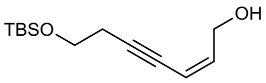
TBSO
OH

S6

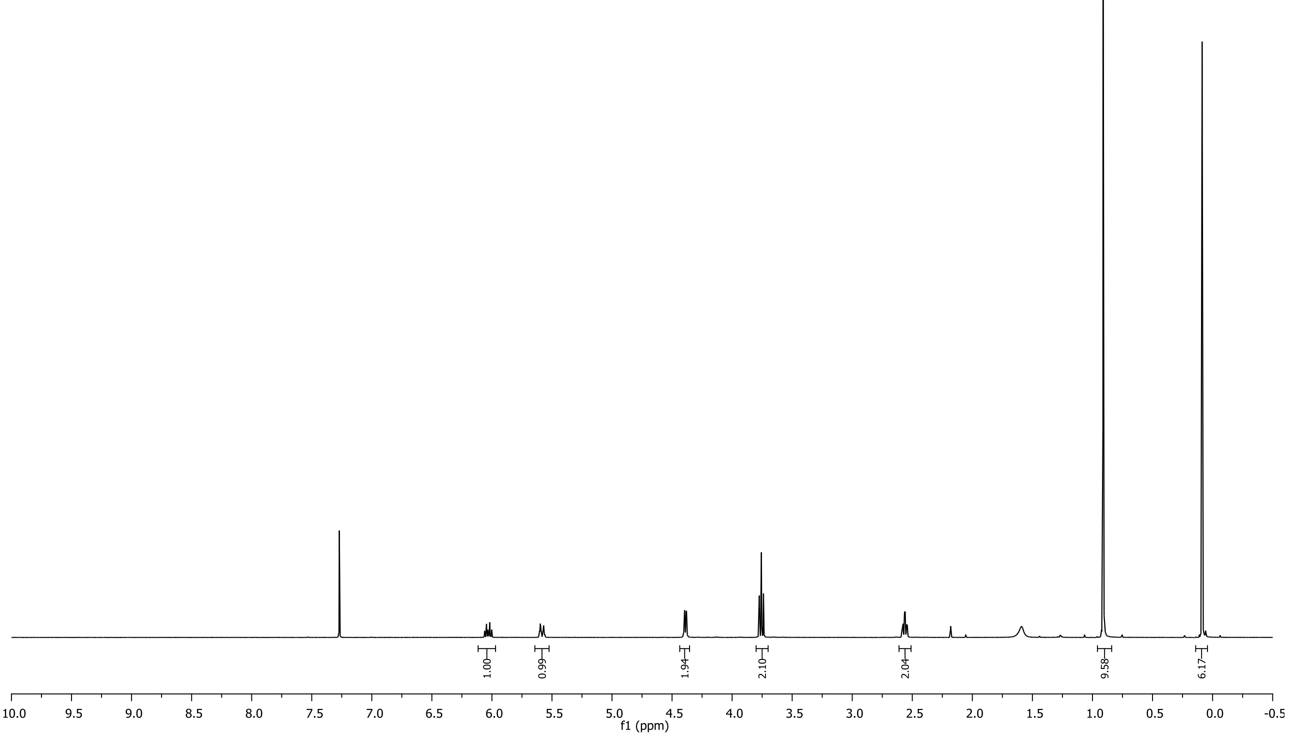
1.00
0.99
1.94
2.10
2.04
9.58
6.17
10.0 9.5 9.0 8.5 8.0 7.5 7.0 6.5 6.0 5.5 5.0 4.5 4.0 3.5 3.0 2.5 2.0 1.5 1.0 0.5 0.0 -0.5
f1 (ppm)

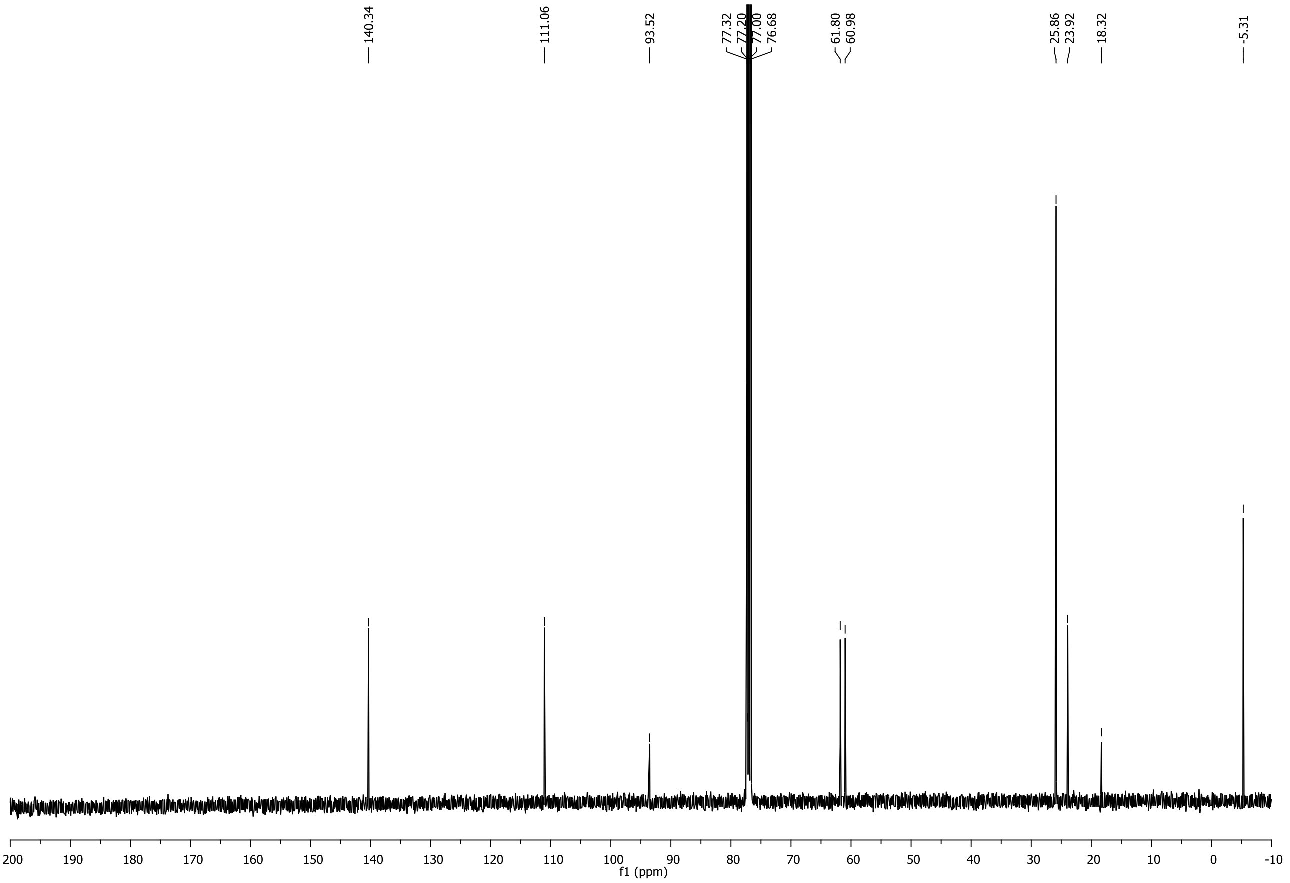
140.34
111.06
93.52
77.32
77.20
77.00
76.68
61.80
60.98
25.86
23.92
18.32
-5.31
200 190 180 170 160 150 140 130 120 110 100 90 80 70 60 50 40 30 20 10 0 -10
f1 (ppm)

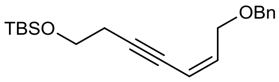
TBSO
OBn

2c

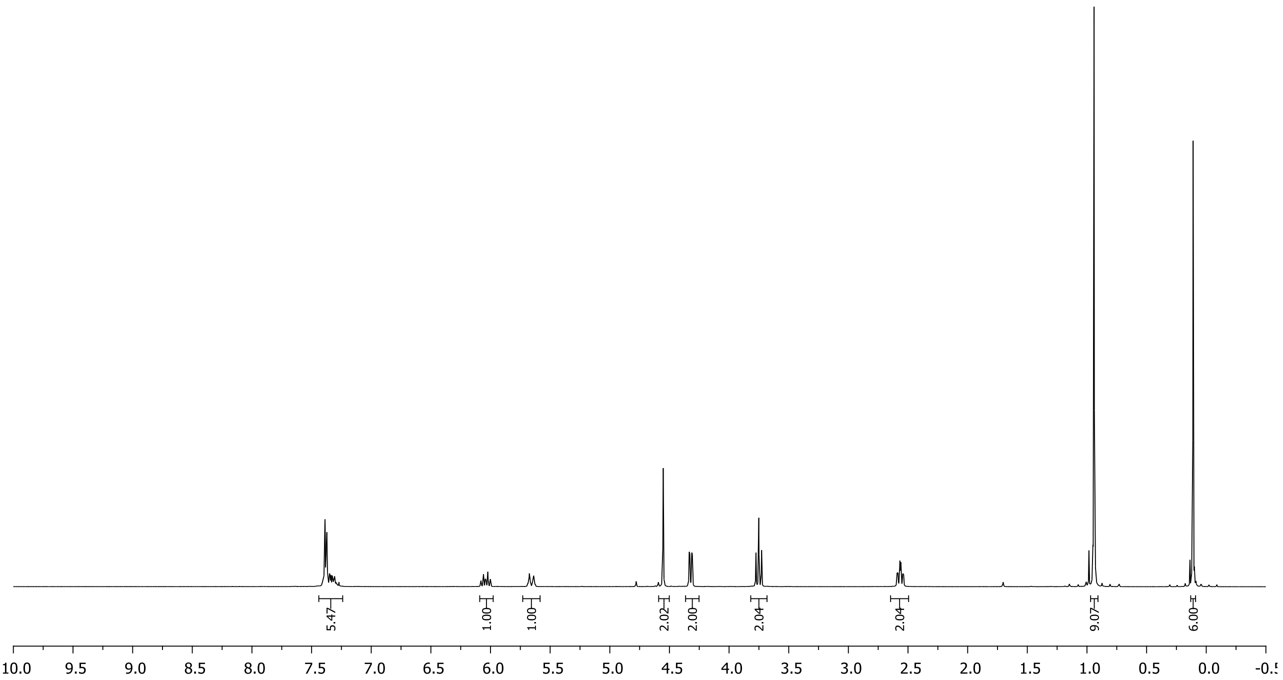
5.47
1.00
1.00
2.02
2.00
2.04
2.04
9.07
6.00
10.0 9.5 9.0 8.5 8.0 7.5 7.0 6.5 6.0 5.5 5.0 4.5 4.0 3.5 3.0 2.5 2.0 1.5 1.0 0.5 0.0

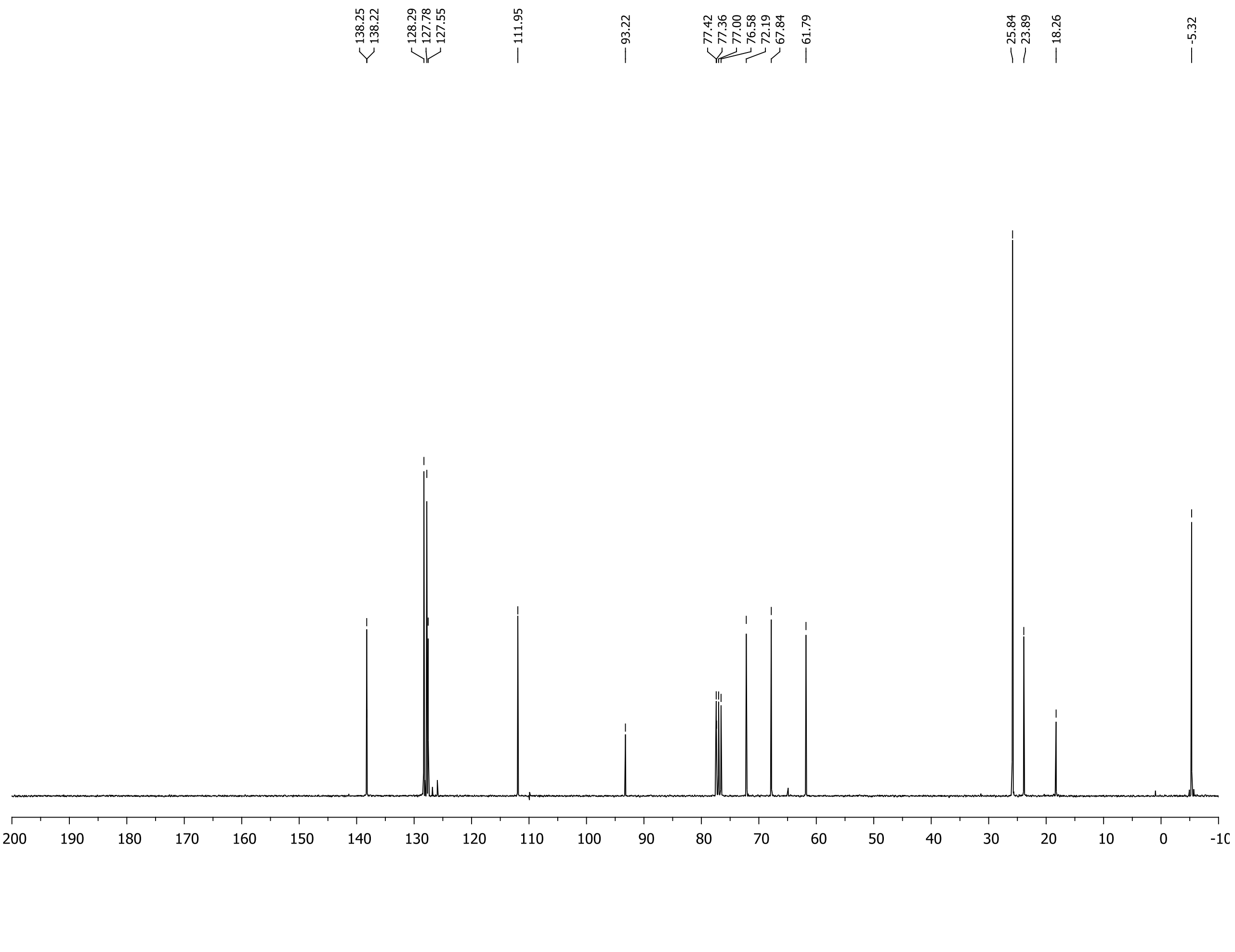
138.25
138.22
128.29
127.78
127.55
111.95
93.22
77.42
77.36
77.00
76.58
72.19
67.84
61.79
25.84
23.89
18.26
-5.32
200 190 180 170 160 150 140 130 120 110 100 90 80 70 60 50 40 30 20 10 0

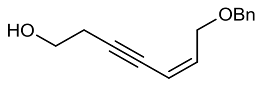
HO
OBn

2d

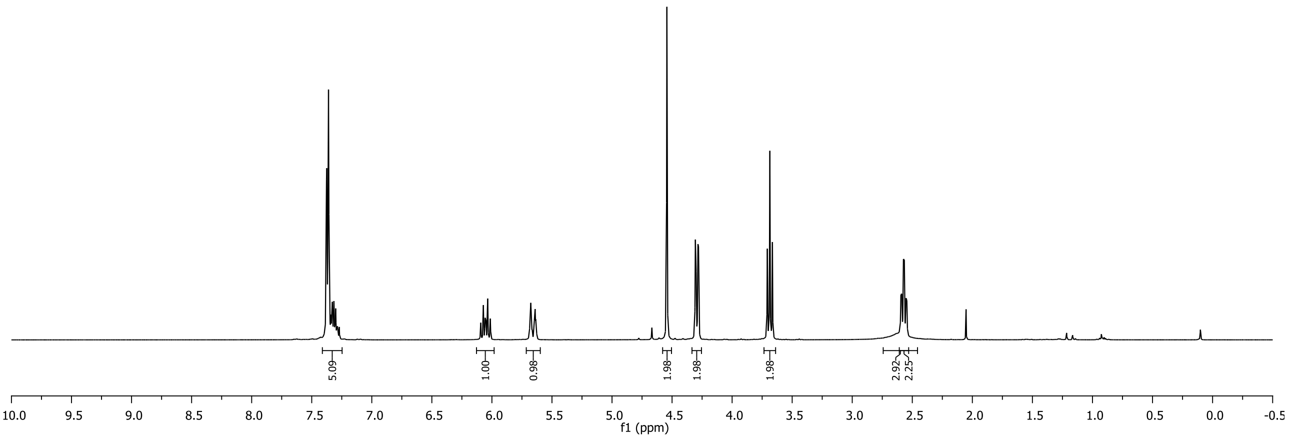
5.09
1.00
0.98
1.98
1.98
1.98
2.92
2.25
10.0 9.5 9.0 8.5 8.0 7.5 7.0 6.5 6.0 5.5 5.0 4.5 4.0 3.5 3.0 2.5 2.0 1.5 1.0 0.5 0.0 -0.5
f1 (ppm)

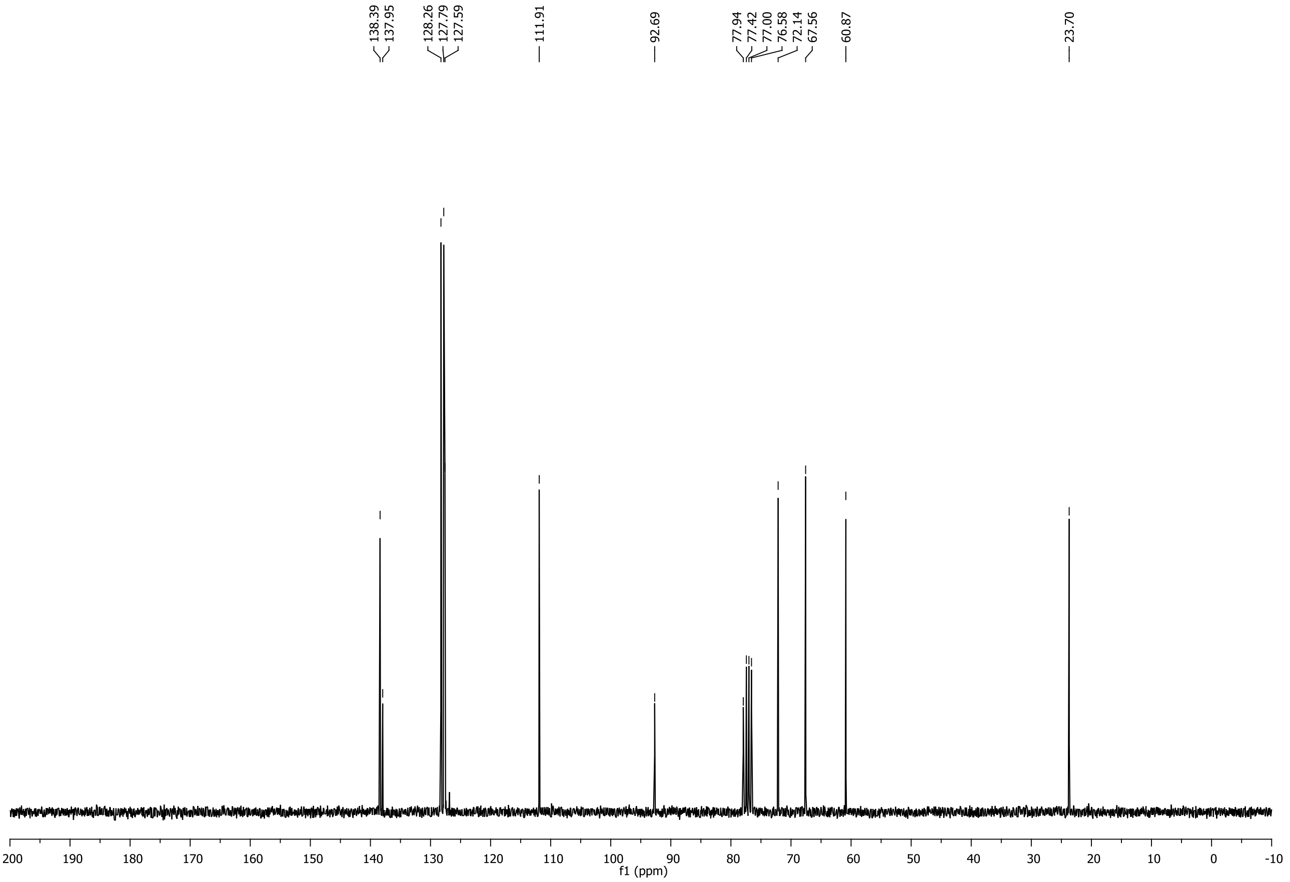
138.39
137.95
128.26
127.79
127.59
111.91
92.69
77.94
77.42
77.00
76.58
72.14
67.56
60.87
23.70
200 190 180 170 160 150 140 130 120 110 100 90 80 70 60 50 40 30 20 10 0 -10
f1 (ppm)

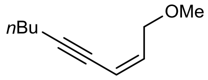
nBu
OMe

2e

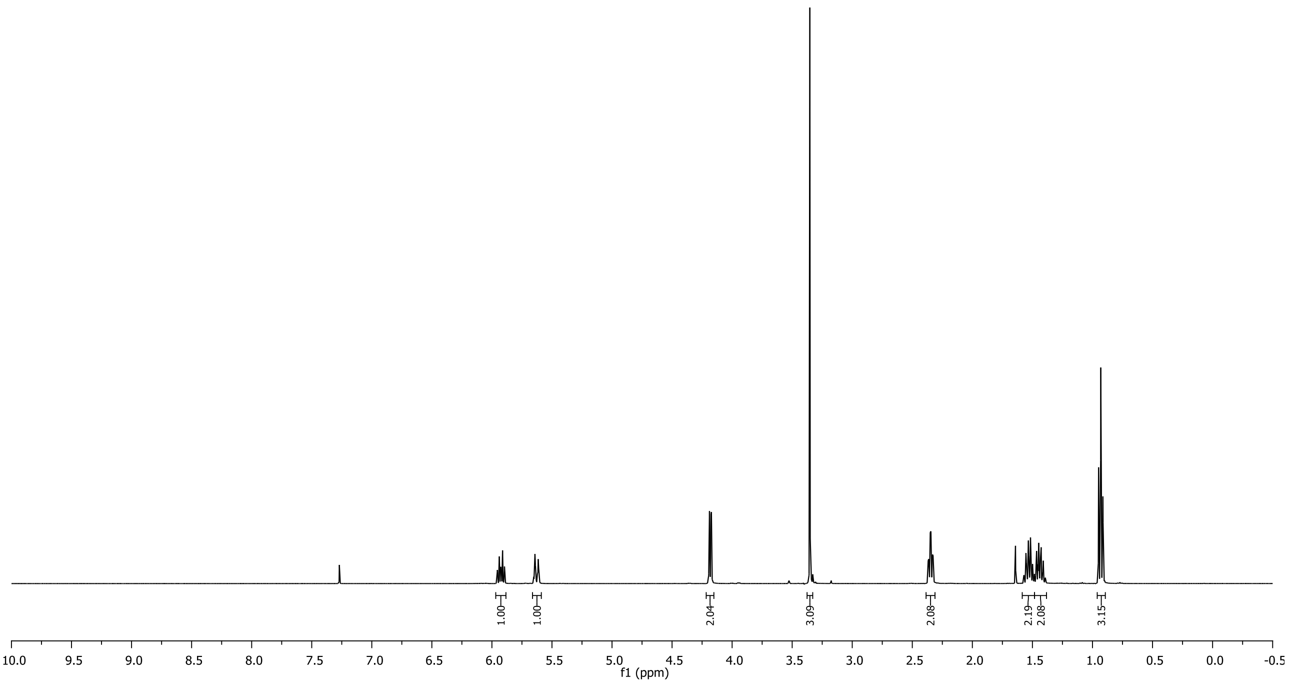
1.00
1.00
2.04
3.09
2.08
2.19
2.08
3.15
10.0
9.5
9.0
8.5
8.0
7.5
7.0
6.5
6.0
5.5
5.0
4.5
4.0
3.5
3.0
2.5
2.0
1.5
1.0
0.5
0.0
-0.5
f1 (ppm)

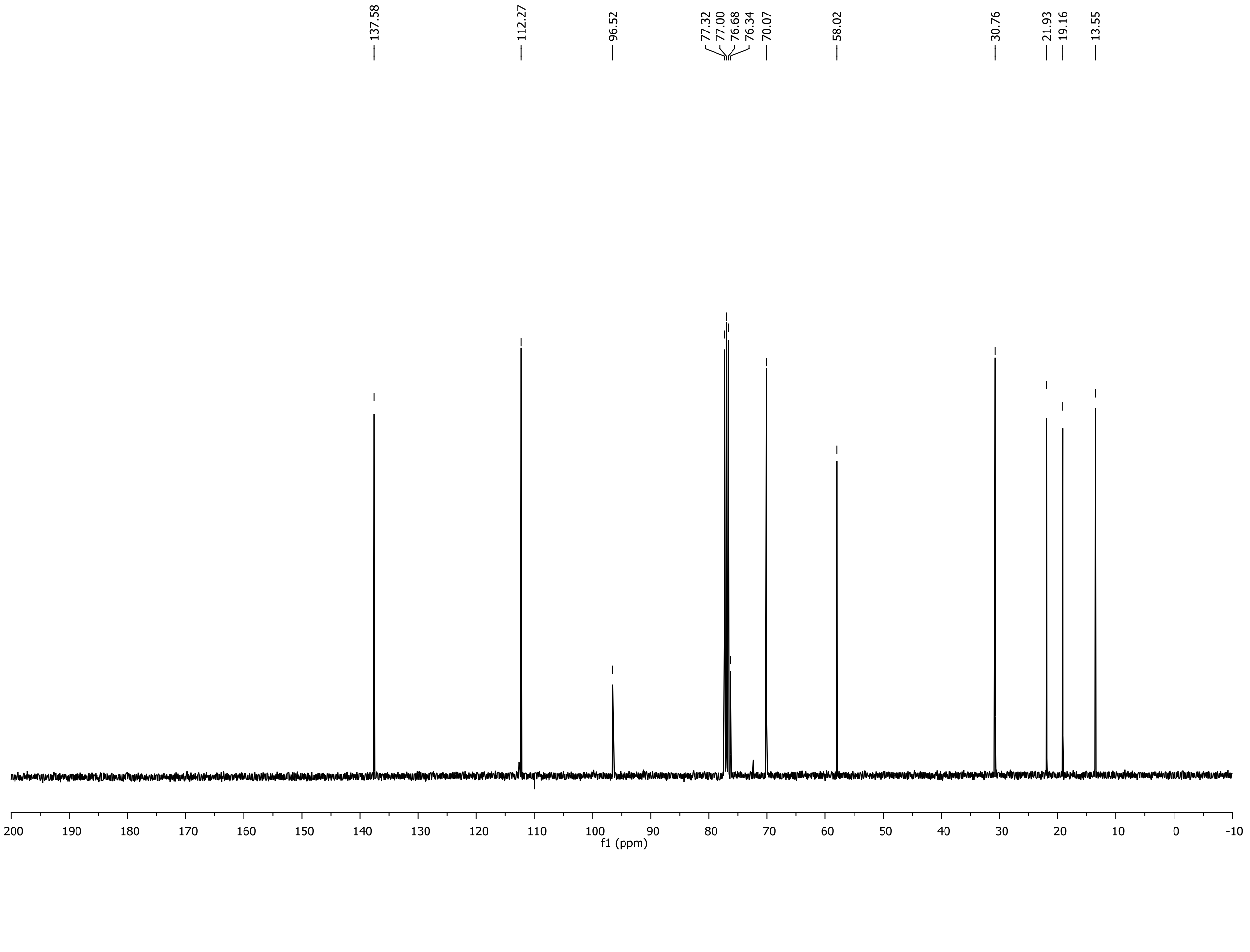
137.58
112.27
96.52
77.32
77.00
76.68
76.34
70.07
58.02
30.76
21.93
19.16
13.55
200
190
180
170
160
150
140
130
120
110
100
90
80
70
60
50
40
30
20
10
0
-10
f1 (ppm)

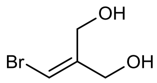

S8

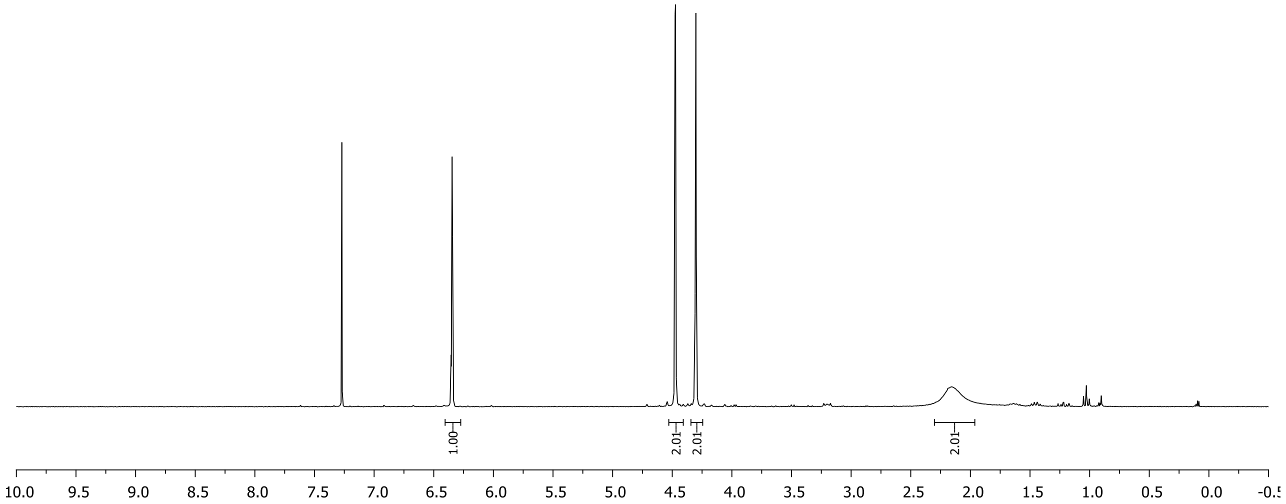
1.00
2.01
2.01
2.01
10.0
9.5
9.0
8.5
8.0
7.5
7.0
6.5
6.0
5.5
5.0
4.5
4.0
3.5
3.0
2.5
2.0
1.5
1.0
0.5
0.0

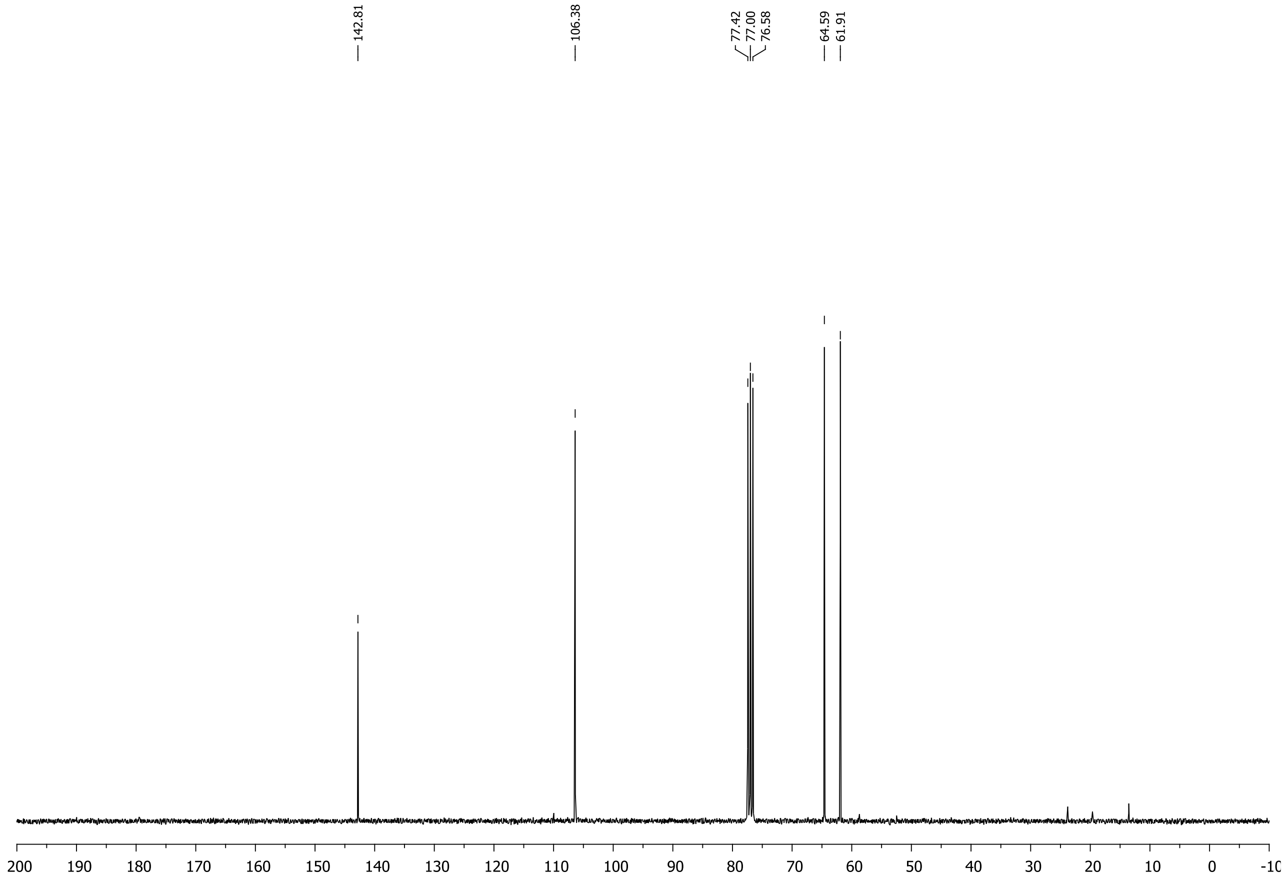
142.81
106.38
77.42
77.00
76.58
64.59
61.91
200
190
180
170
160
150
140
130
120
110
100
90
80
70
60
50
40
30
20
10
0

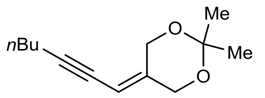
nBu
O
O
Me
Me

2i

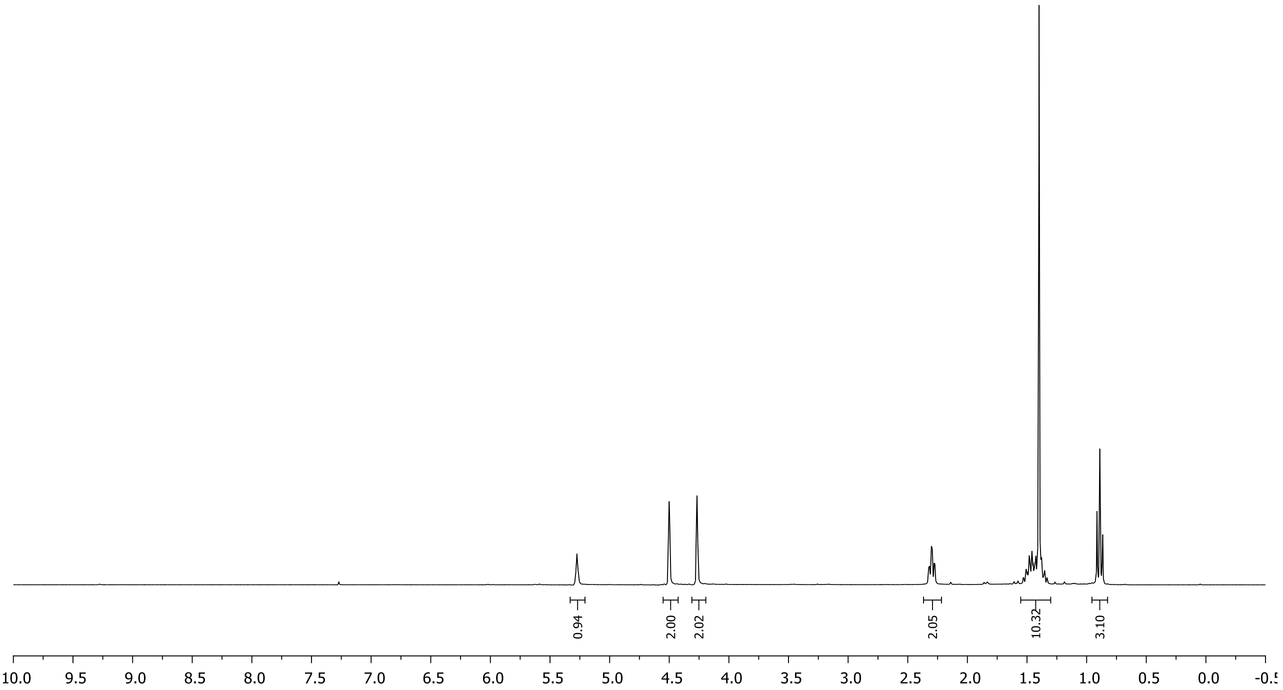
0.94
2.00
2.02
2.05
10.32
3.10
10.0 9.5 9.0 8.5 8.0 7.5 7.0 6.5 6.0 5.5 5.0 4.5 4.0 3.5 3.0 2.5 2.0 1.5 1.0 0.5 0.0

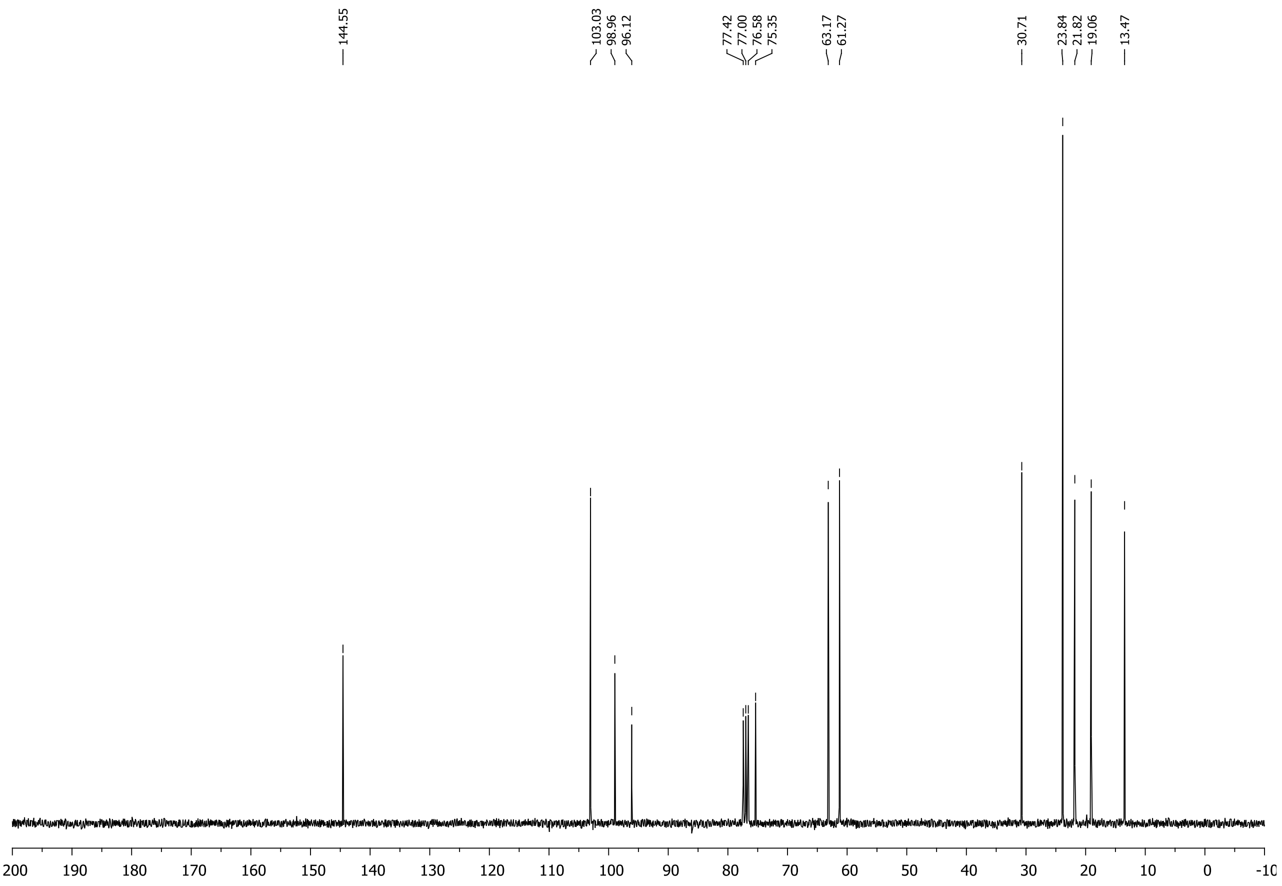
144.55
103.03
98.96
96.12
77.42
77.00
76.58
75.35
63.17
61.27
30.71
23.84
21.82
19.06
13.47
200 190 180 170 160 150 140 130 120 110 100 90 80 70 60 50 40 30 20 10 0

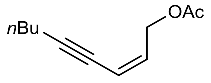
nBu
OAc

2k

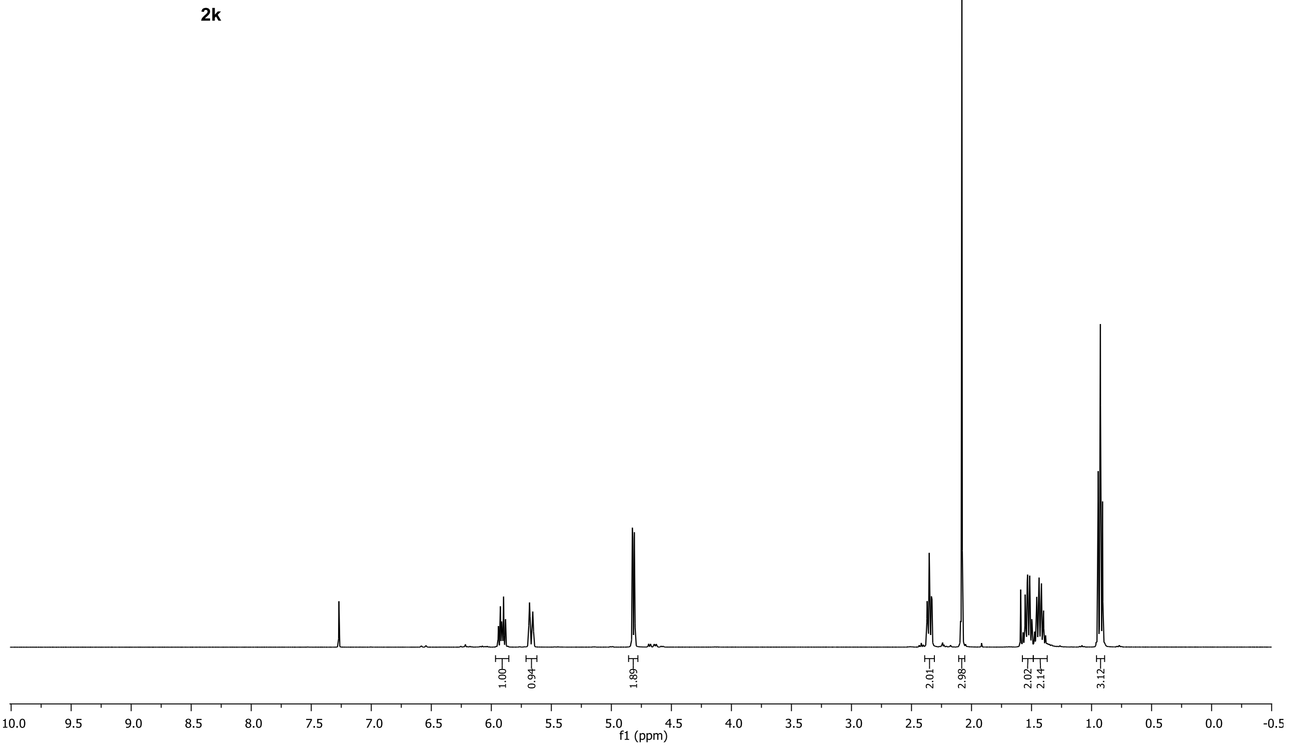
1.00
0.94
1.89
2.01
2.98
2.02
2.14
3.12
10.0 9.5 9.0 8.5 8.0 7.5 7.0 6.5 6.0 5.5 5.0 4.5 4.0 3.5 3.0 2.5 2.0 1.5 1.0 0.5 0.0 -0.5
f1 (ppm)

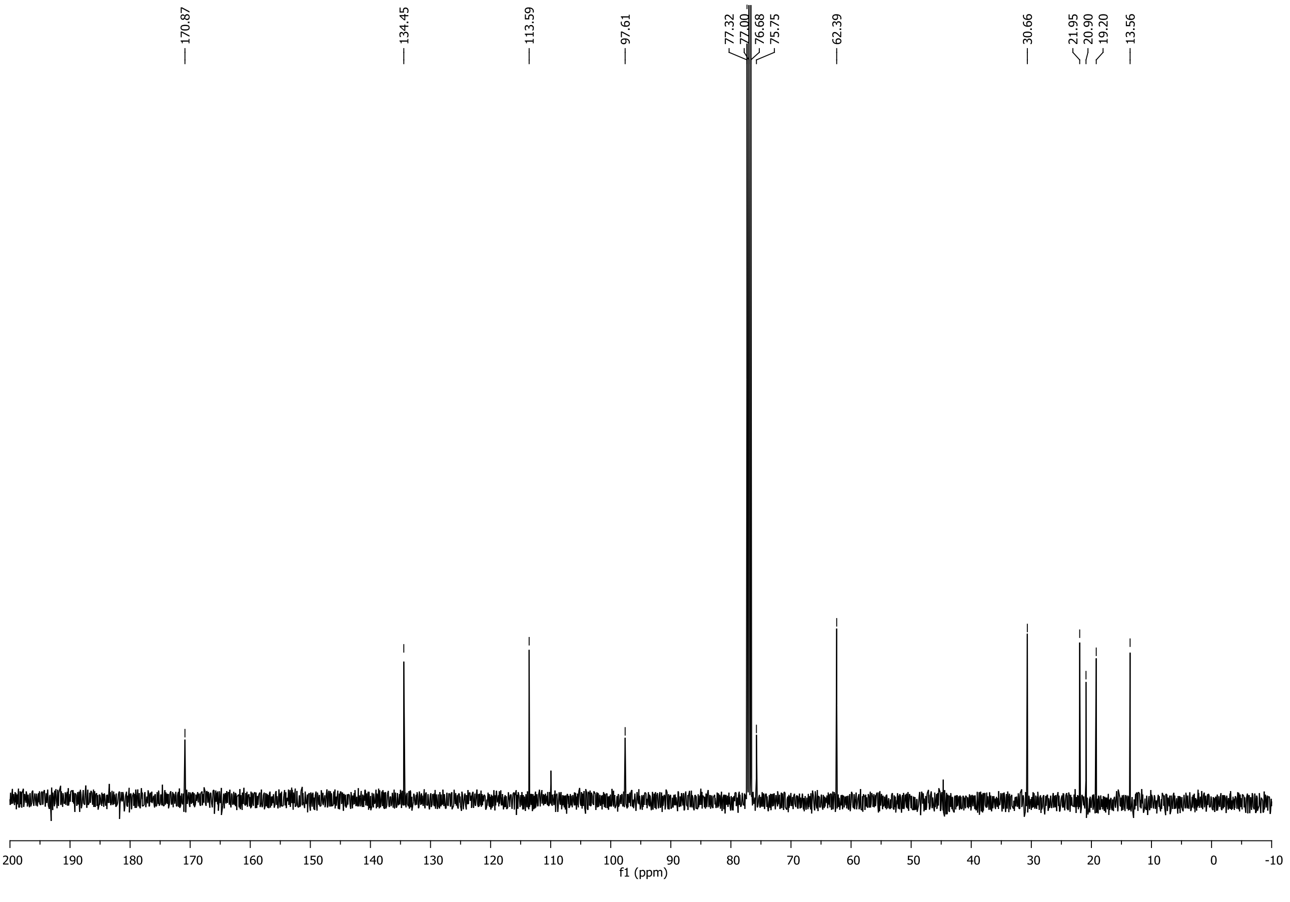
170.87
134.45
113.59
97.61
77.32
77.00
76.68
75.75
62.39
30.66
21.95
20.90
19.20
13.56
200 190 180 170 160 150 140 130 120 110 100 90 80 70 60 50 40 30 20 10 0 -10
f1 (ppm)

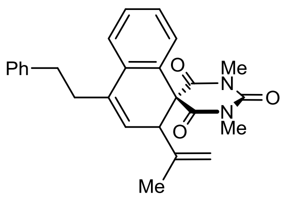

3a

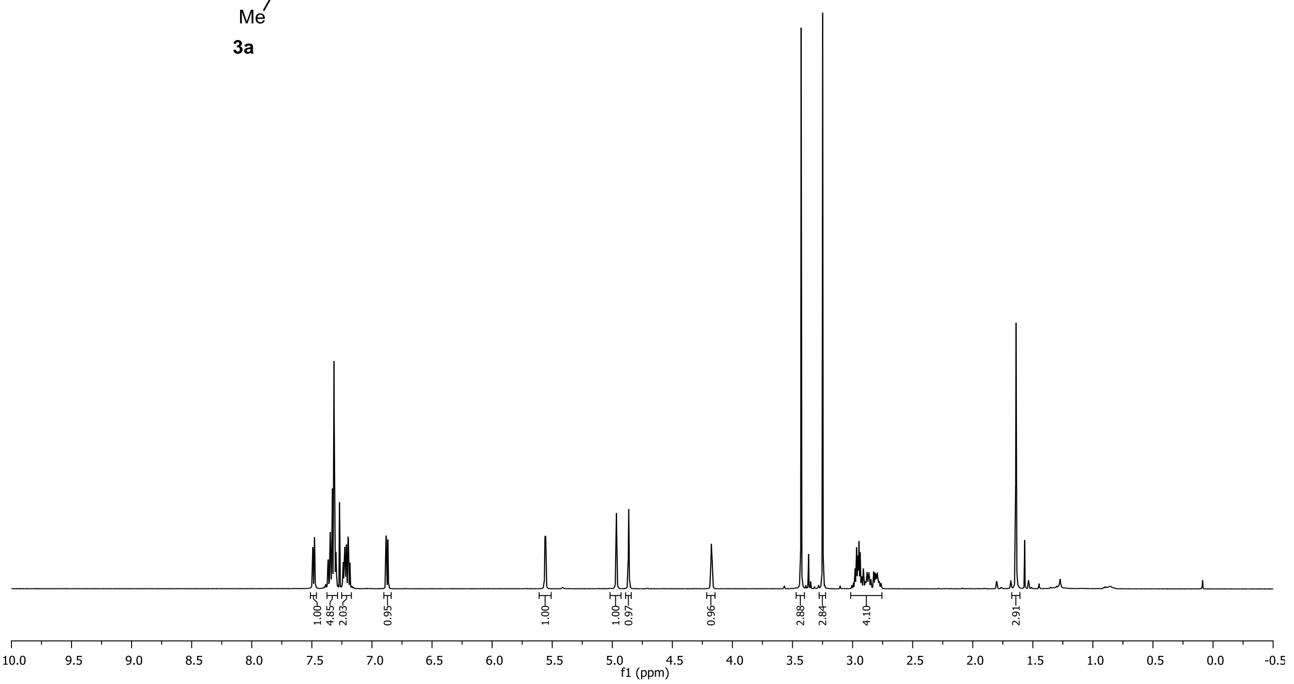

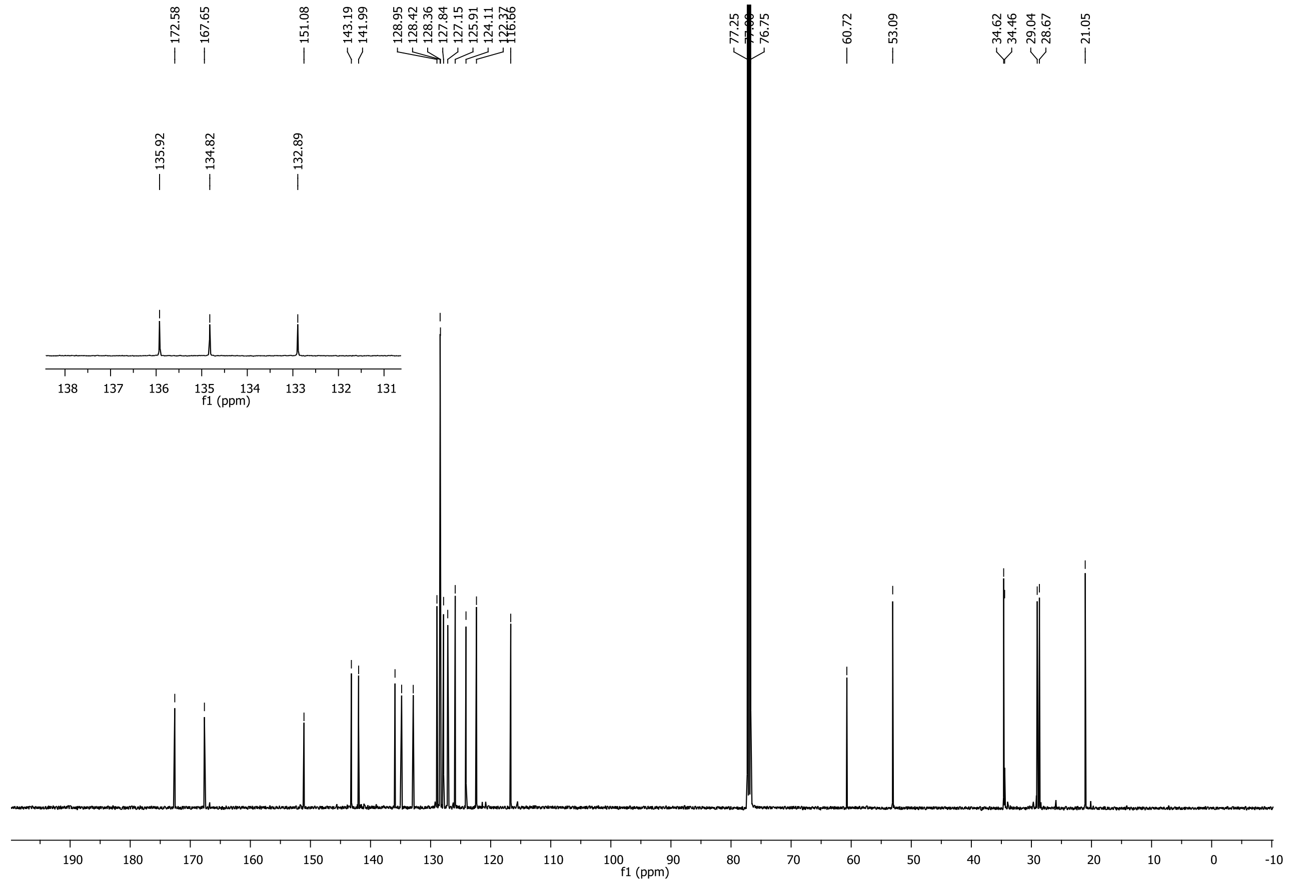

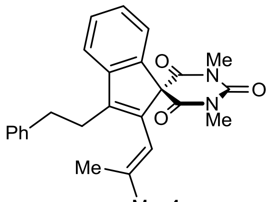
Me
N
O
O
N
Me
O
Ph
Me
Me

4a

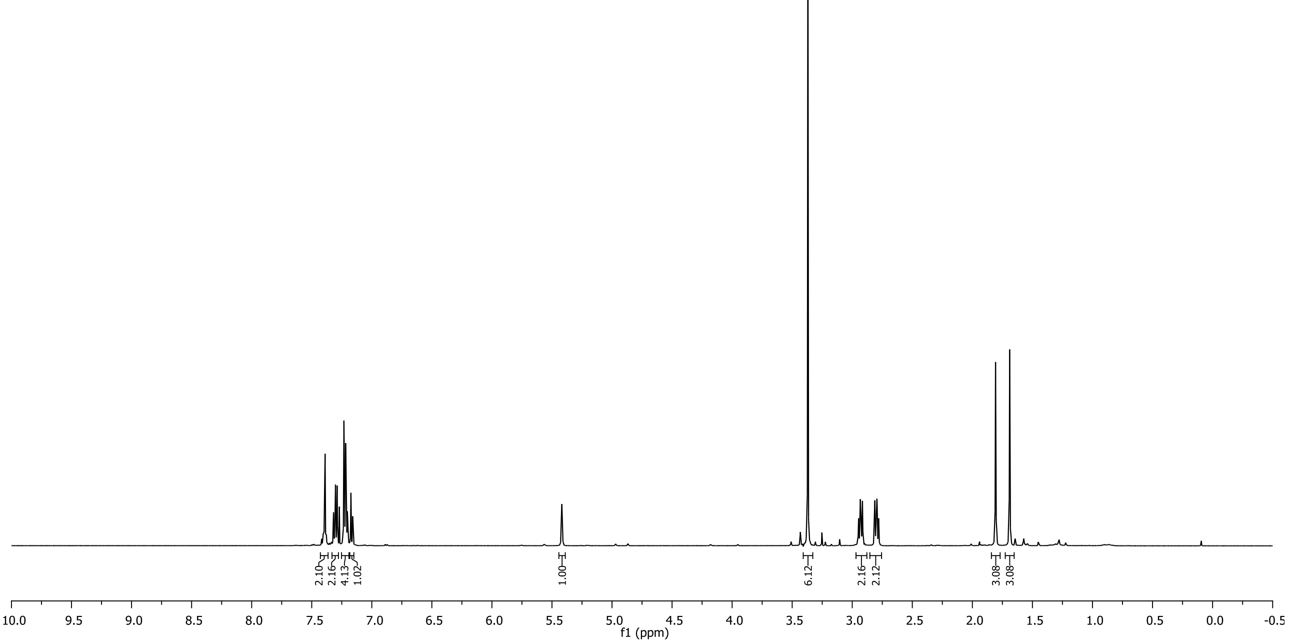
2.10
2.16
4.13
1.02
1.00
6.12
2.16
2.12
3.08
3.08
10.0 9.5 9.0 8.5 8.0 7.5 7.0 6.5 6.0 5.5 5.0 4.5 4.0 3.5 3.0 2.5 2.0 1.5 1.0 0.5 0.0 -0.5
f1 (ppm)

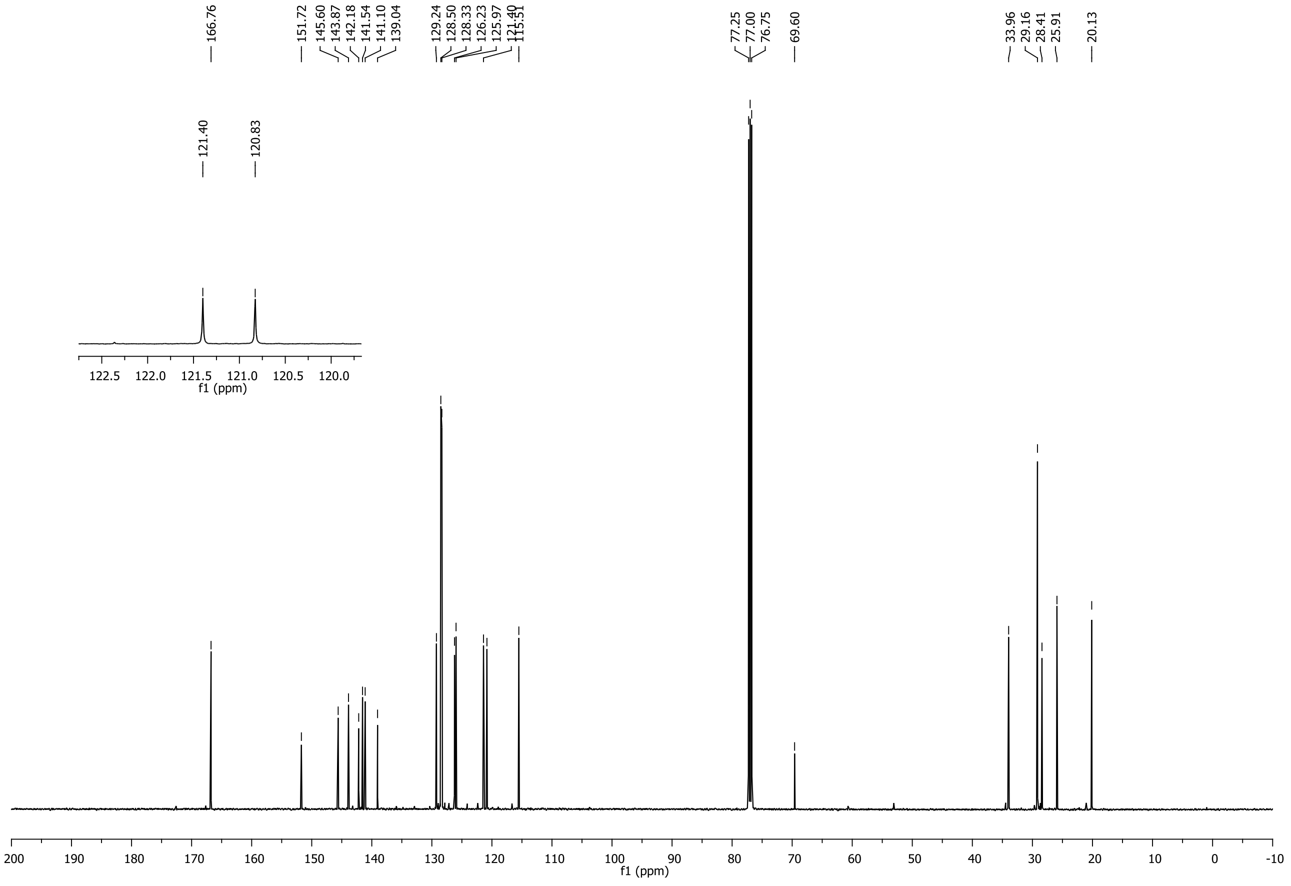
166.76
151.72
145.60
143.87
142.18
141.54
141.10
139.04
129.24
128.50
128.33
126.23
125.97
121.40
115.51
77.25
77.00
76.75
69.60
33.96
29.16
28.41
25.91
20.13
121.40
120.83
122.5 122.0 121.5 121.0 120.5 120.0
f1 (ppm)
200 190 180 170 160 150 140 130 120 110 100 90 80 70 60 50 40 30 20 10 0 -10
f1 (ppm)

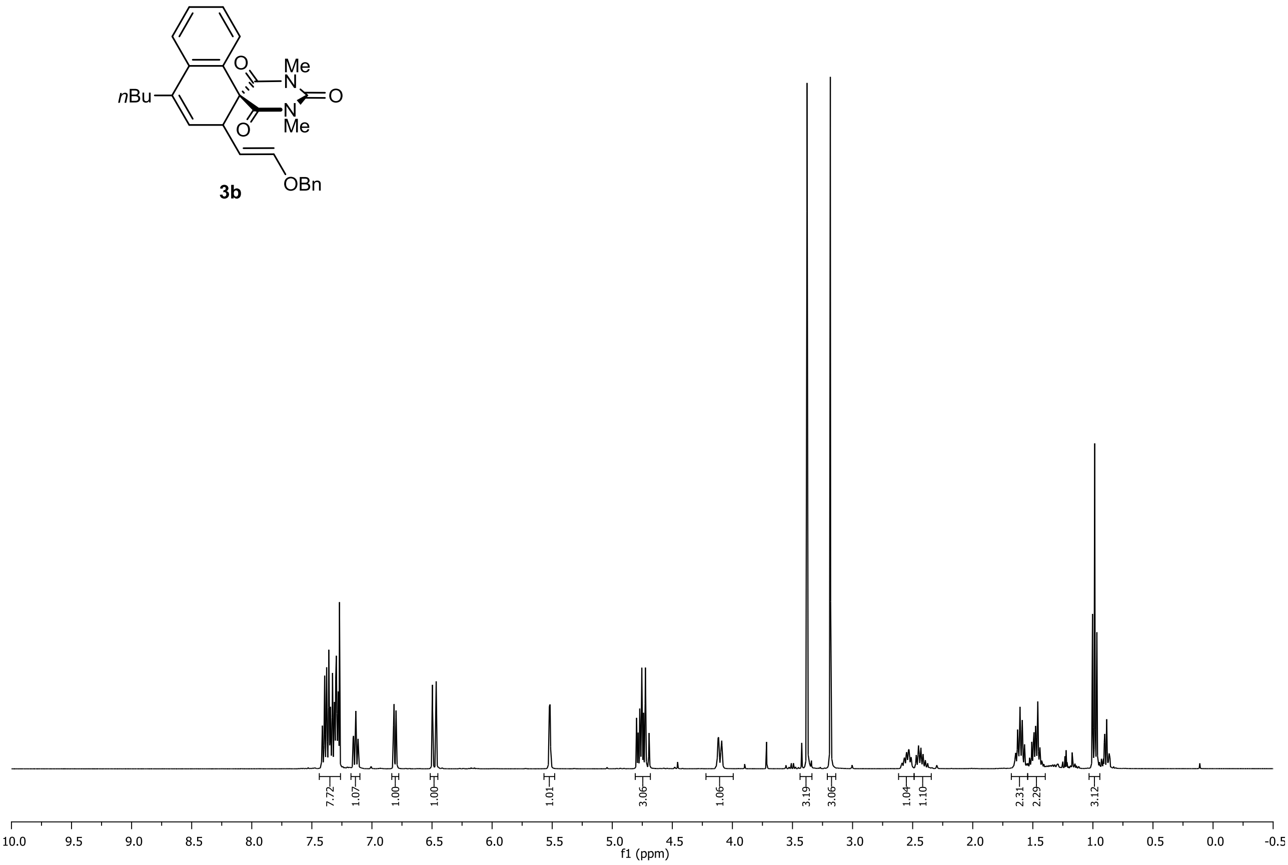
nBu
O
Me
N
O
N
O
Me
OBn
3b
7.72
1.07
1.00
1.00
1.01
3.06
1.06
3.19
3.06
1.04
1.10
2.31
2.29
3.12
10.0 9.5 9.0 8.5 8.0 7.5 7.0 6.5 6.0 5.5 5.0 4.5 4.0 3.5 3.0 2.5 2.0 1.5 1.0 0.5 0.0 -0.5
f1 (ppm)

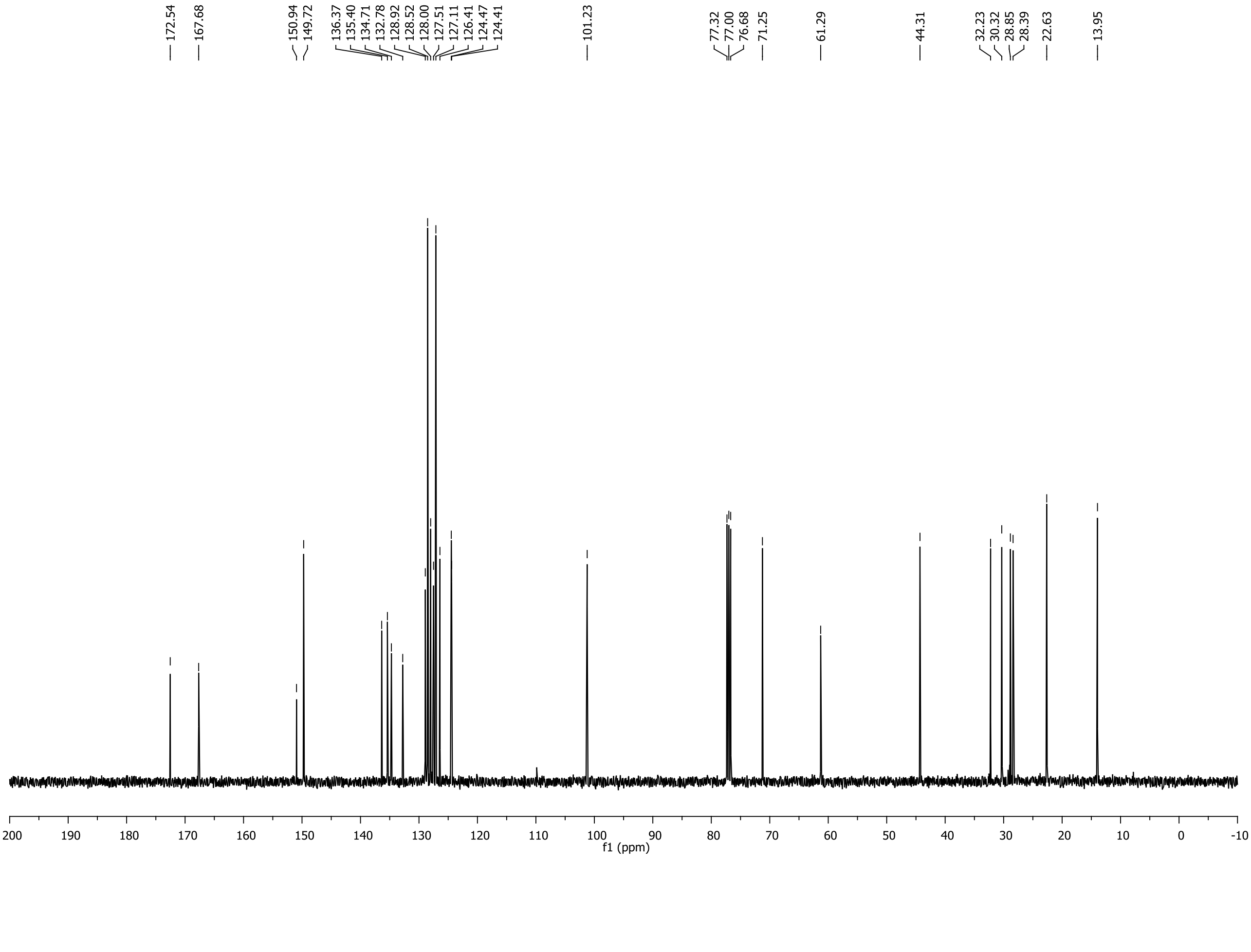
172.54
167.68
150.94
149.72
136.37
135.40
134.71
132.78
128.92
128.52
128.00
127.51
127.11
126.41
124.47
124.41
101.23
77.32
77.00
76.68
71.25
61.29
44.31
32.23
30.32
28.85
28.39
22.63
13.95
200 190 180 170 160 150 140 130 120 110 100 90 80 70 60 50 40 30 20 10 0 -10
f1 (ppm)

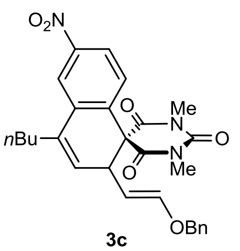
O2N
nBu
O
Me
N
O
N
O
Me
OBn

3c

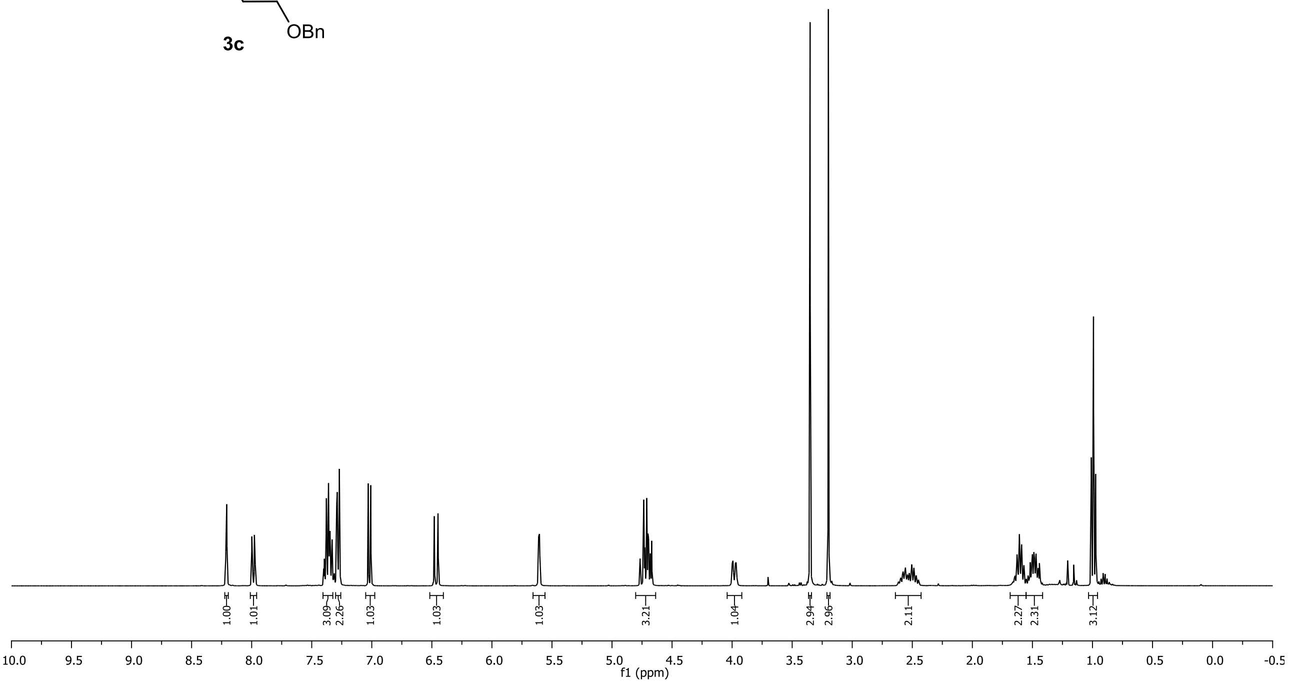
1.00
1.01
3.09
2.26
1.03
1.03
1.03
3.21
1.04
2.94
2.96
2.11
2.27
2.31
3.12
f1 (ppm)

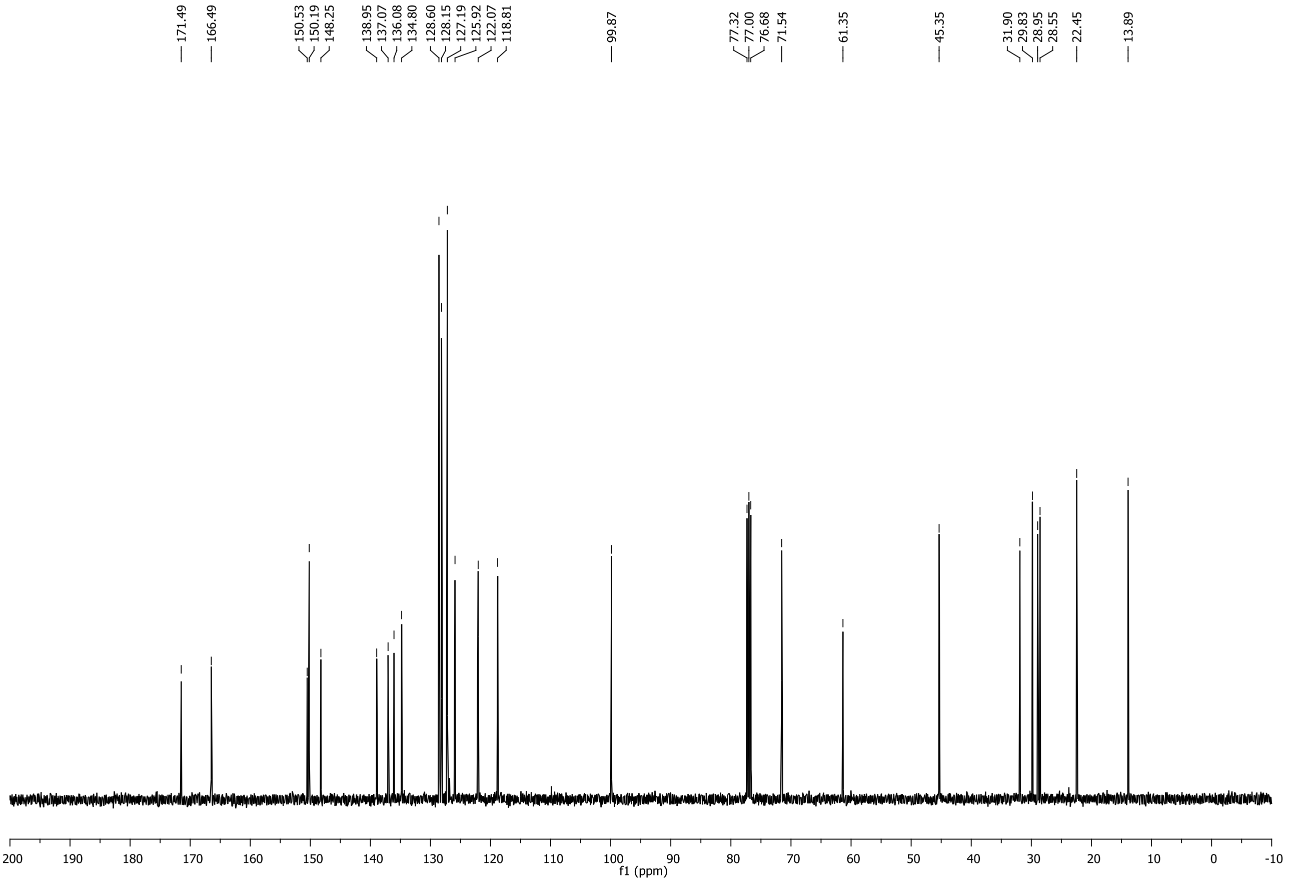
171.49
166.49
150.53
150.19
148.25
138.95
137.07
136.08
134.80
128.60
128.15
127.19
125.92
122.07
118.81
99.87
77.32
77.00
76.68
71.54
61.35
45.35
31.90
29.83
28.95
28.55
22.45
13.89
f1 (ppm)

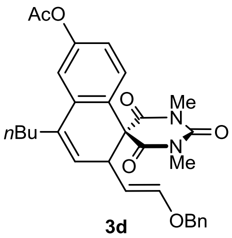
AcO
nBu
O
Me
N
O
N
O
Me
OBn

3d

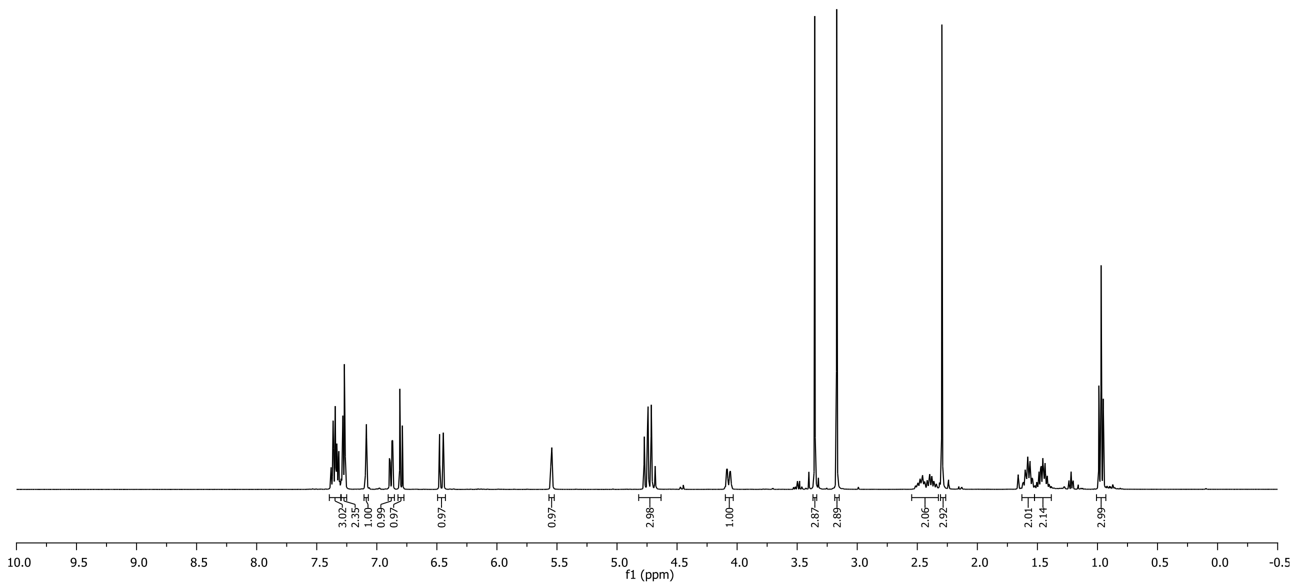
3.02
2.35
1.00
0.99
0.97
0.97
0.97
2.98
1.00
2.87
2.89
2.06
2.92
2.01
2.14
2.99
10.0 9.5 9.0 8.5 8.0 7.5 7.0 6.5 6.0 5.5 5.0 4.5 4.0 3.5 3.0 2.5 2.0 1.5 1.0 0.5 0.0 -0.5
f1 (ppm)

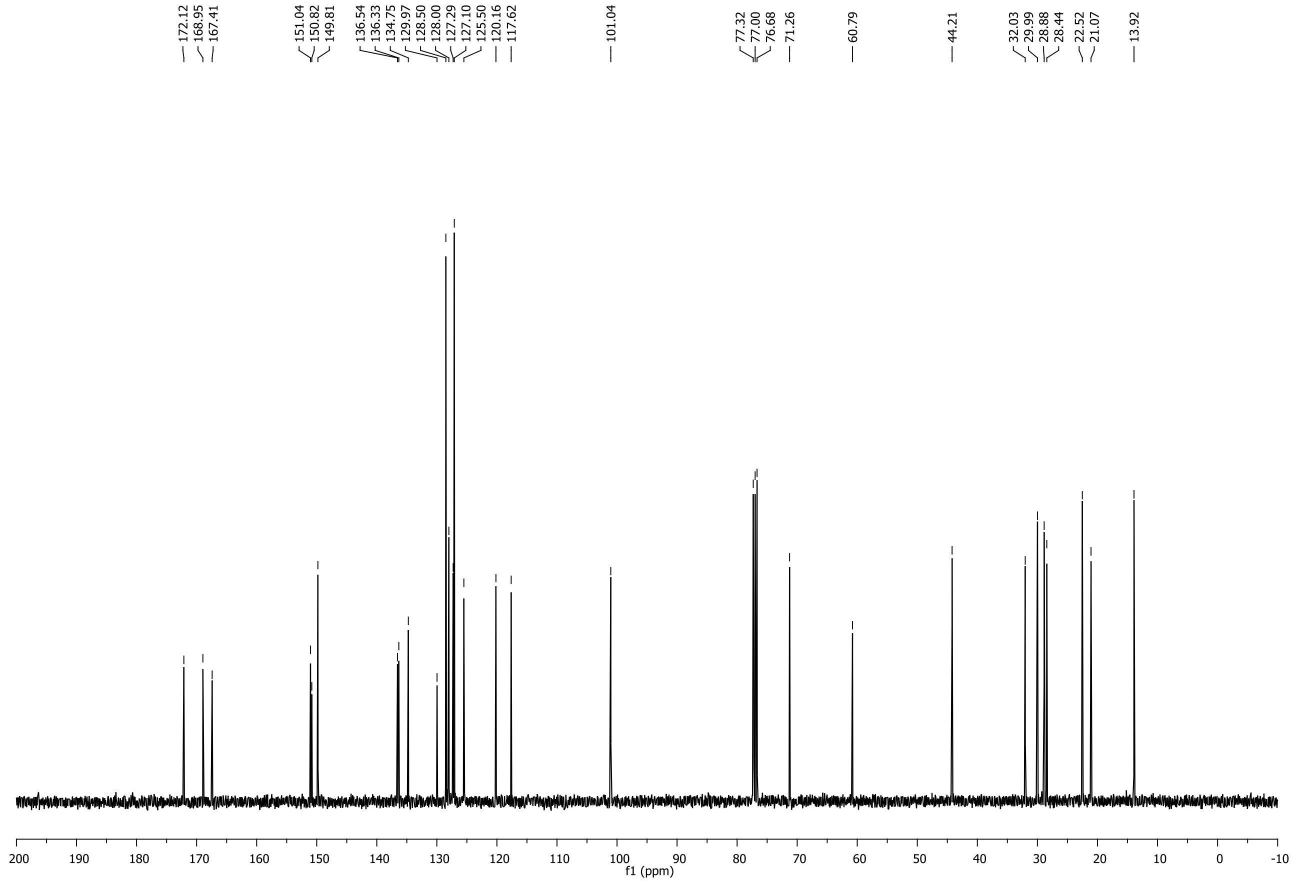
172.12
168.95
167.41
151.04
150.82
149.81
136.54
136.33
134.75
129.97
128.50
128.00
127.29
127.10
125.50
120.16
117.62
101.04
77.32
77.00
76.68
71.26
60.79
44.21
32.03
29.99
28.88
28.44
22.52
21.07
13.92
200 190 180 170 160 150 140 130 120 110 100 90 80 70 60 50 40 30 20 10 0 -10
f1 (ppm)

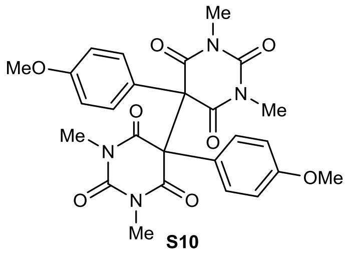
MeO
OMe
Me
N
O
Me

S10

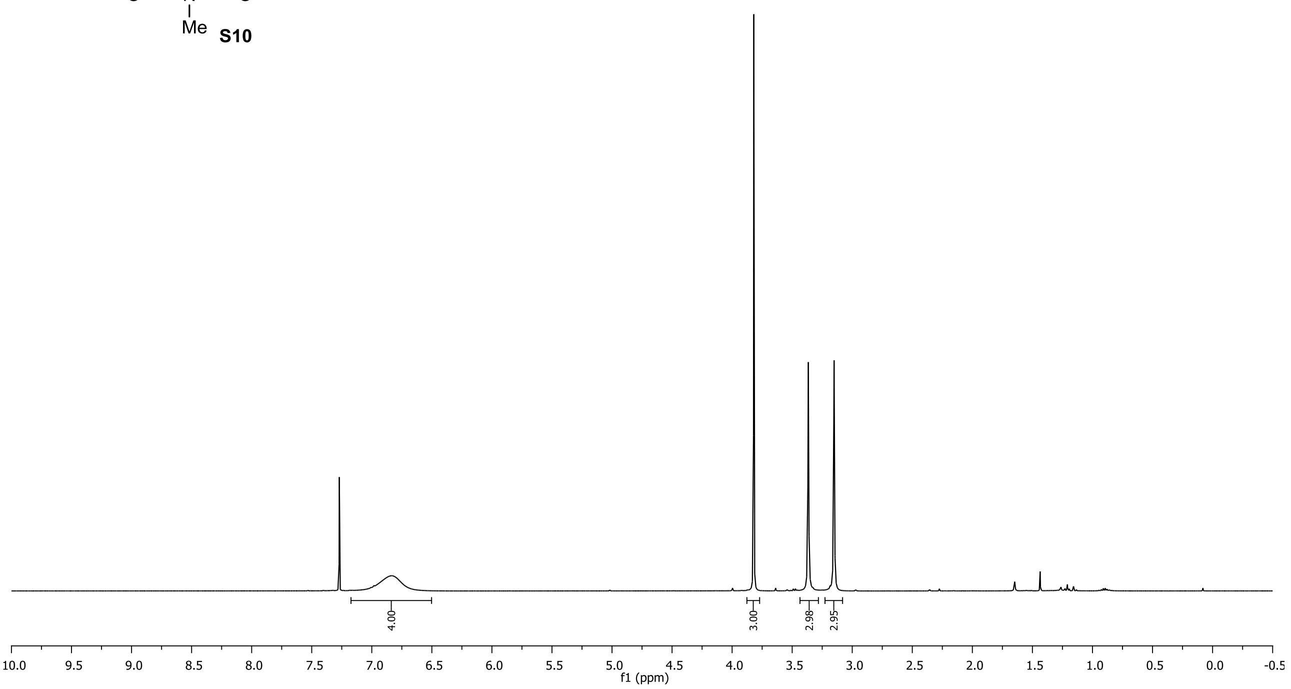
4.00
3.00
2.98
2.95
f1 (ppm)

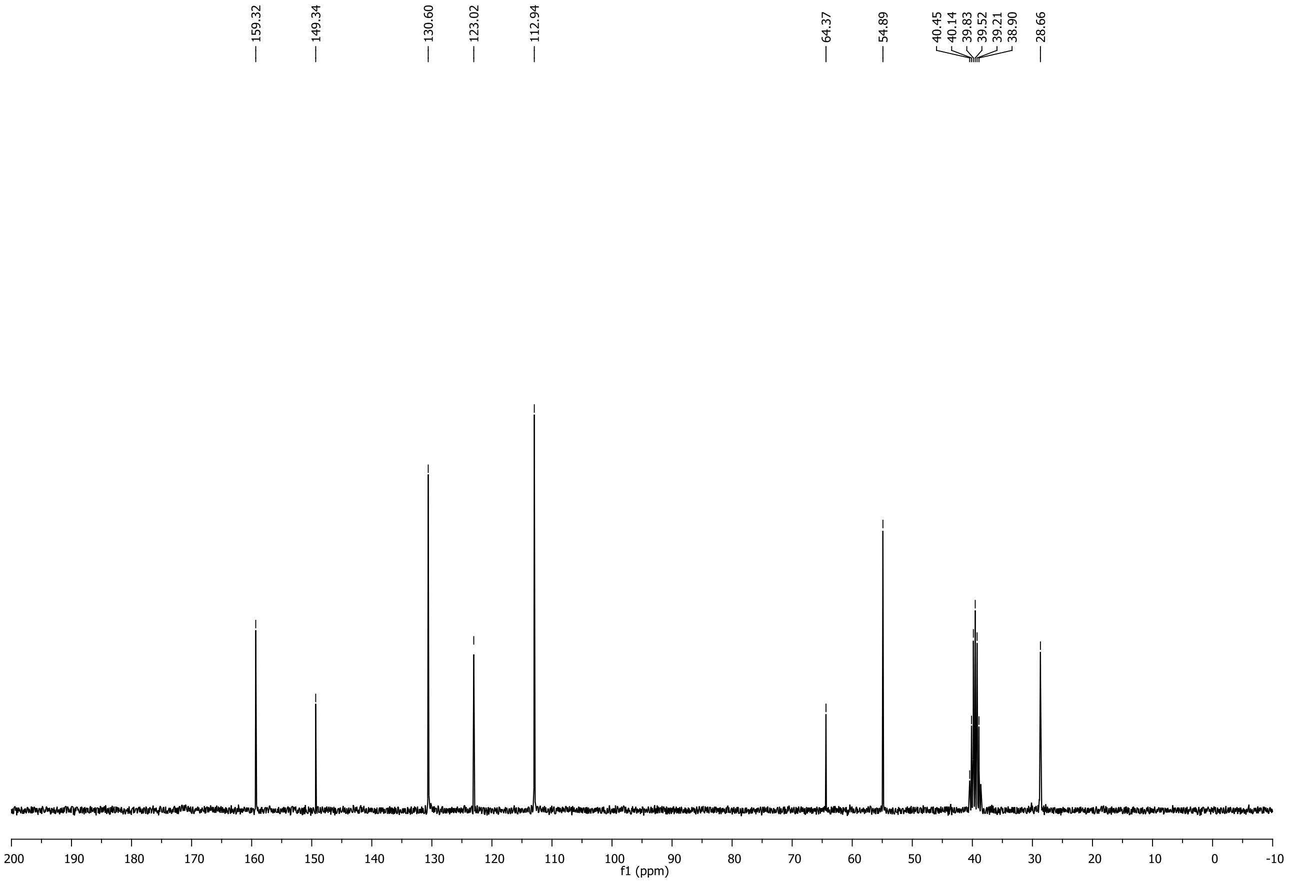
159.32
149.34
130.60
123.02
112.94
64.37
54.89
40.45
40.14
39.83
39.52
39.21
38.90
28.66
f1 (ppm)

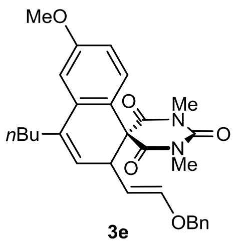
MeO
nBu
O
N
Me
O
O
N
Me
OBn

3e

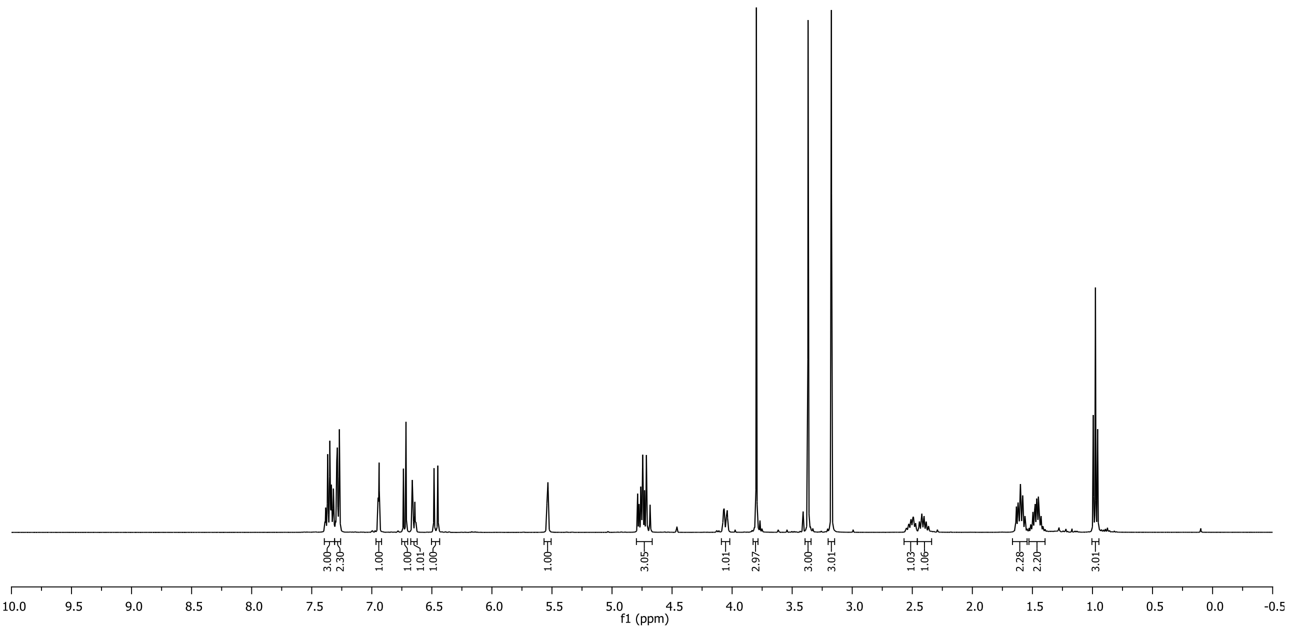
3.00
2.30
1.00
1.00
1.01
1.00
1.00
3.05
1.01
2.97
3.00
3.01
1.03
1.06
2.28
2.20
3.01
10.0 9.5 9.0 8.5 8.0 7.5 7.0 6.5 6.0 5.5 5.0 4.5 4.0 3.5 3.0 2.5 2.0 1.5 1.0 0.5 0.0 -0.5
f1 (ppm)

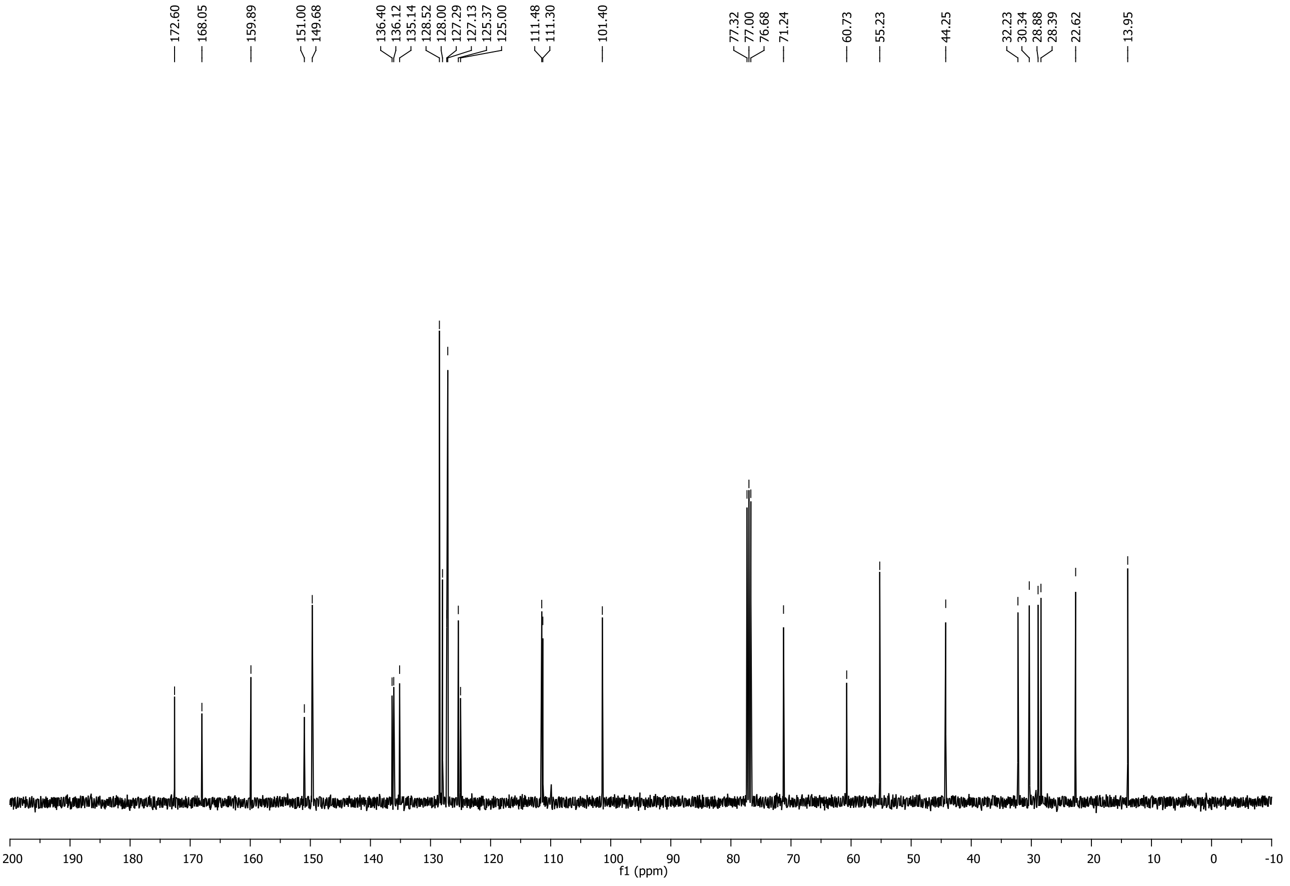
172.60
168.05
159.89
151.00
149.68
136.40
136.12
135.14
128.52
128.00
127.29
127.13
125.37
125.00
111.48
111.30
101.40
77.32
77.00
76.68
71.24
60.73
55.23
44.25
32.23
30.34
28.88
28.39
22.62
13.95
200 190 180 170 160 150 140 130 120 110 100 90 80 70 60 50 40 30 20 10 0 -10
f1 (ppm)

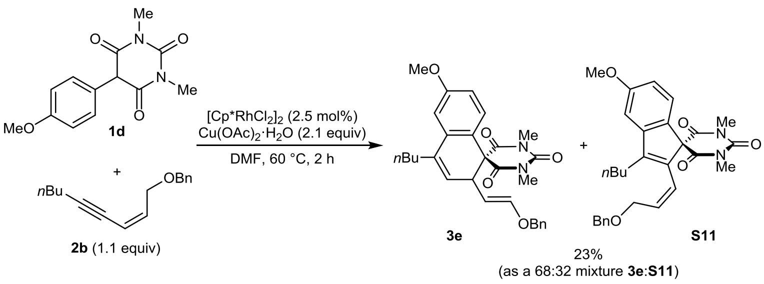

1d
2b (1.1 equiv)
[Cp*RhCl2]2 (2.5 mol%)
Cu(OAc)2·H2O (2.1 equiv)
DMF, 60 °C, 2 h
3e
S11
23%
(as a 68:32 mixture 3e:S11)

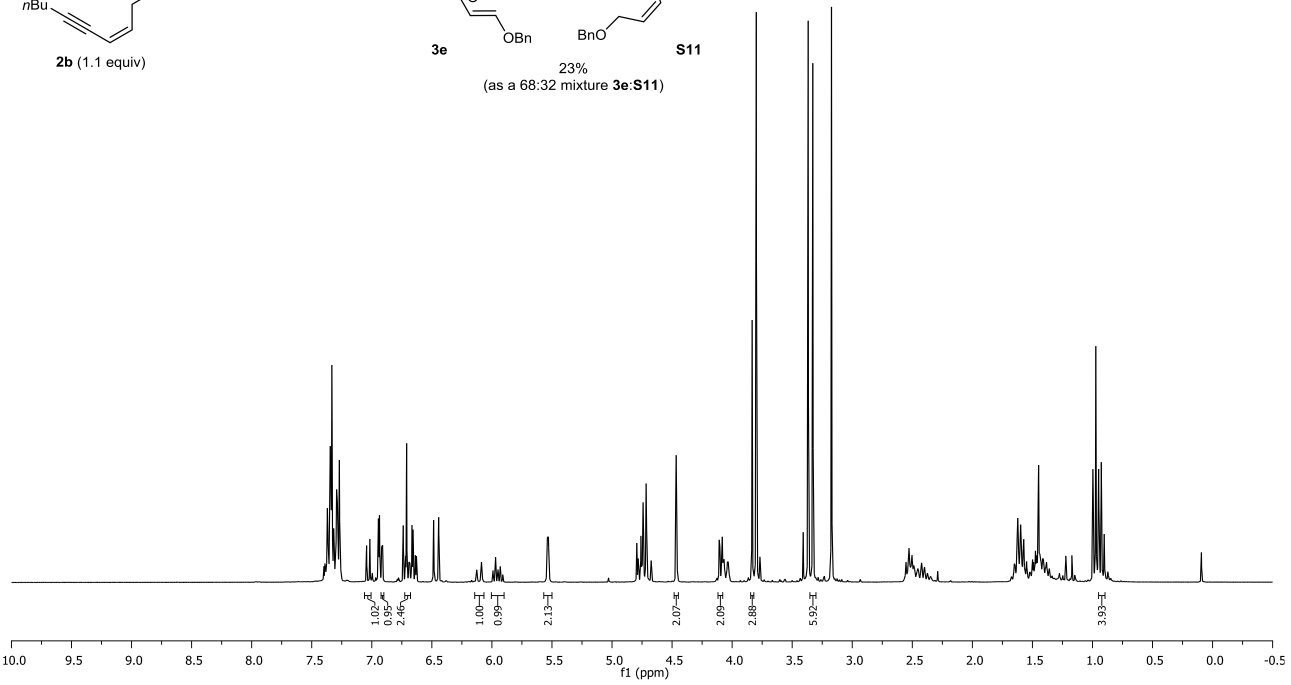

f1 (ppm)

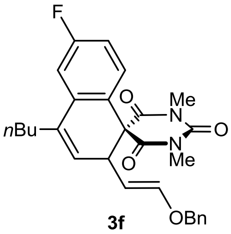
F
nBu
O
Me
N
O
N
O
Me
OBn

3f

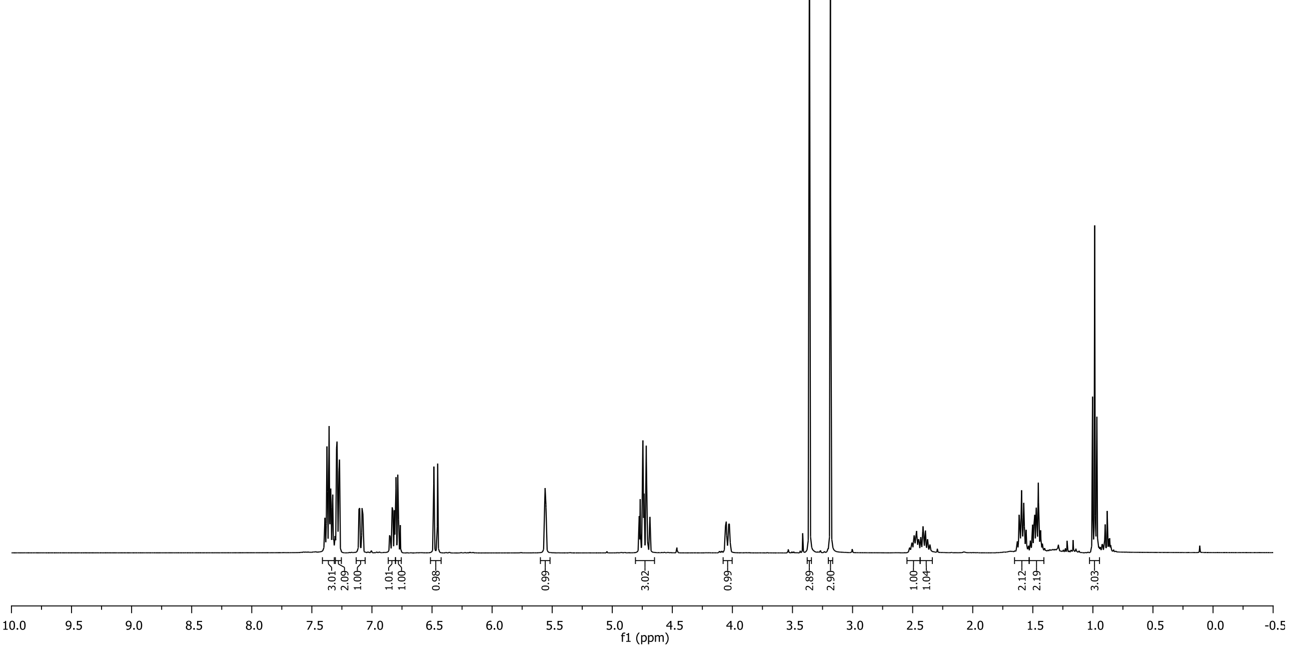
3.01
2.09
1.00
1.01
1.00
0.98
0.99
3.02
0.99
2.89
2.90
1.00
1.04
2.12
2.19
3.03
f1 (ppm)

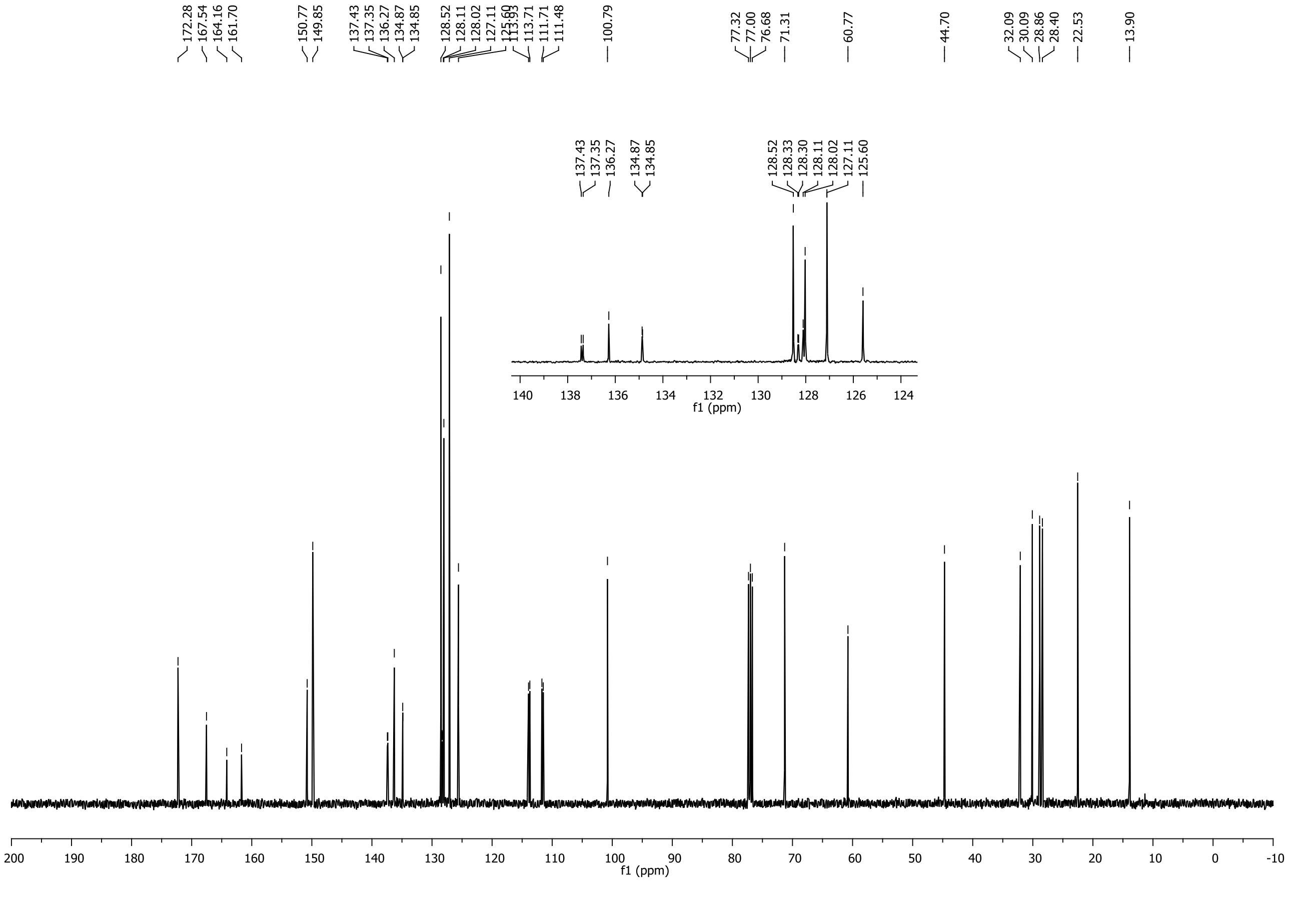
172.28
167.54
164.16
161.70
150.77
149.85
137.43
137.35
136.27
134.87
134.85
128.52
128.11
128.02
127.11
113.71
111.71
111.48
100.79
77.32
77.00
76.68
71.31
60.77
44.70
32.09
30.09
28.86
28.40
22.53
13.90
137.43
137.35
136.27
134.87
134.85
128.52
128.33
128.30
128.11
128.02
127.11
125.60
f1 (ppm)

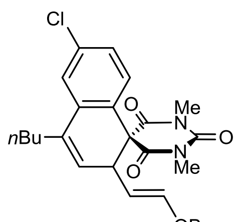
Cl
Me
N
O
nBu
O
N
Me
O
OBn

3g

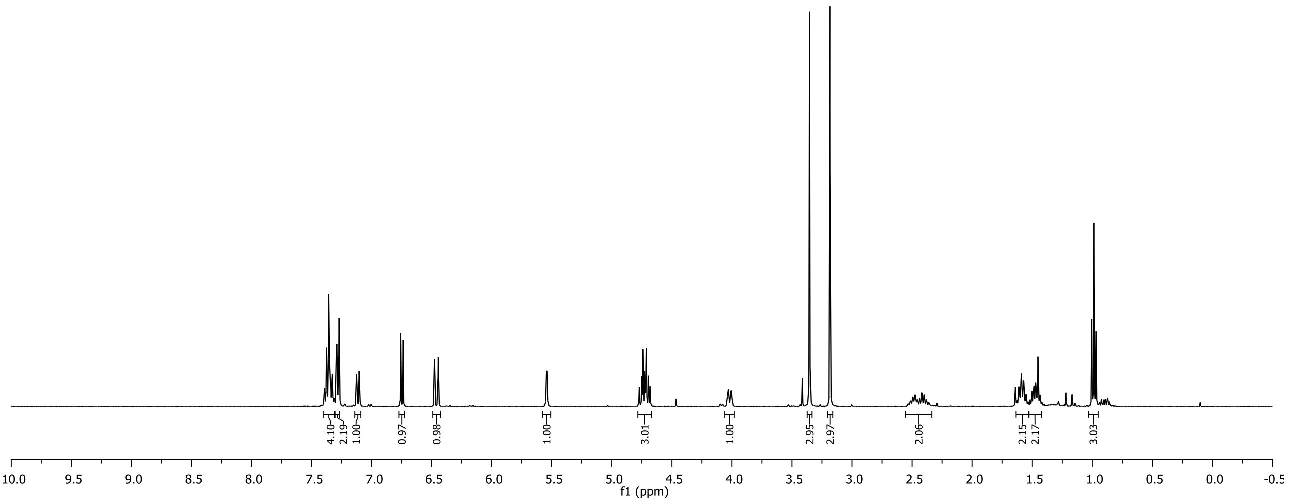
4.10
2.19
1.00
0.97
0.98
1.00
3.01
1.00
2.95
2.97
2.06
2.15
2.17
3.03
10.0
9.5
9.0
8.5
8.0
7.5
7.0
6.5
6.0
5.5
5.0
4.5
4.0
3.5
3.0
2.5
2.0
1.5
1.0
0.5
0.0
-0.5
f1 (ppm)

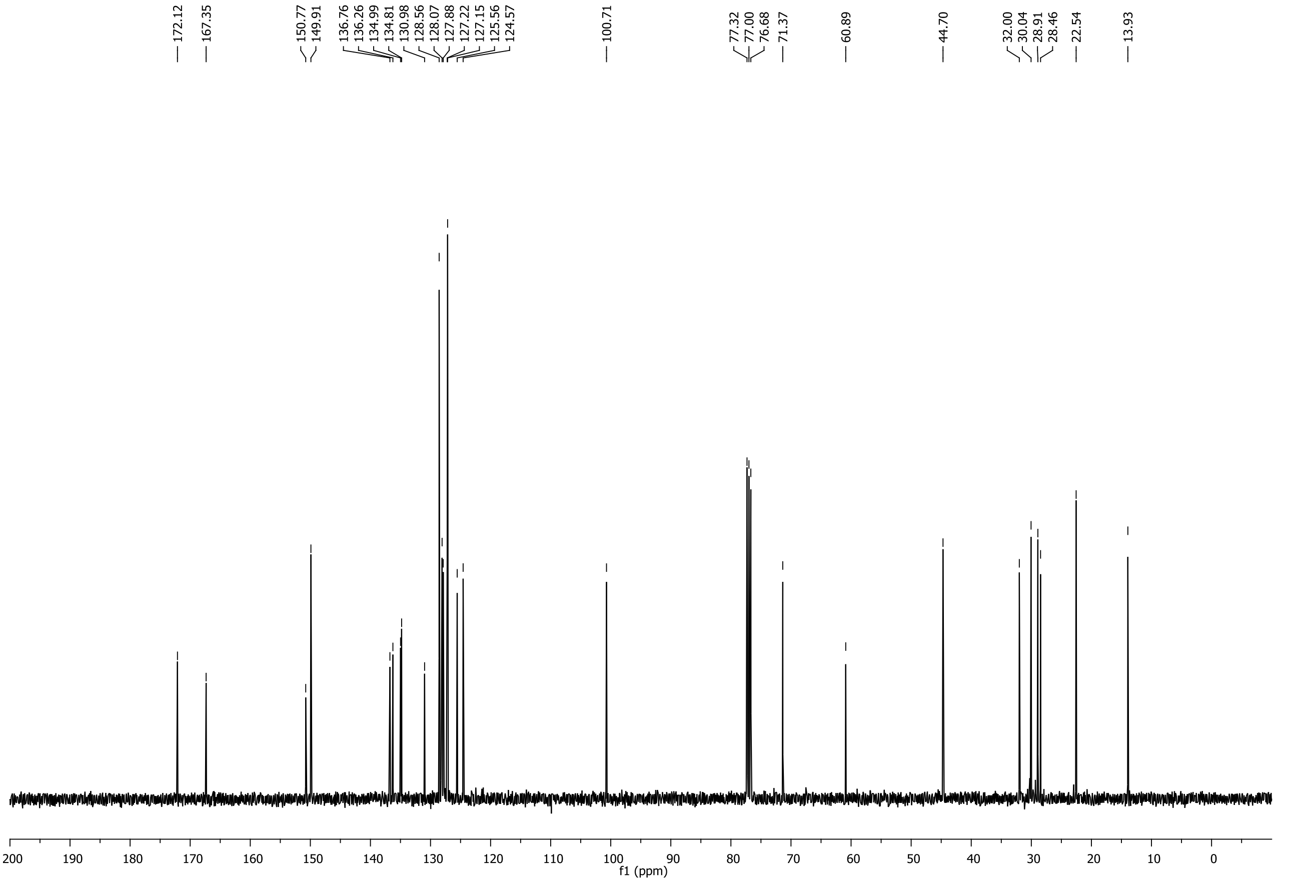
172.12
167.35
150.77
149.91
136.76
136.26
134.99
134.81
130.98
128.56
128.07
127.88
127.22
127.15
125.56
124.57
100.71
77.32
77.00
76.68
71.37
60.89
44.70
32.00
30.04
28.91
28.46
22.54
13.93
200
190
180
170
160
150
140
130
120
110
100
90
80
70
60
50
40
30
20
10
0
f1 (ppm)

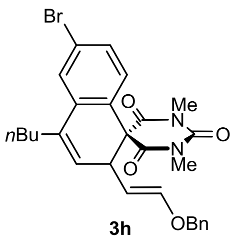
Br
nBu
O
Me
N
O
N
O
Me
OBn

3h

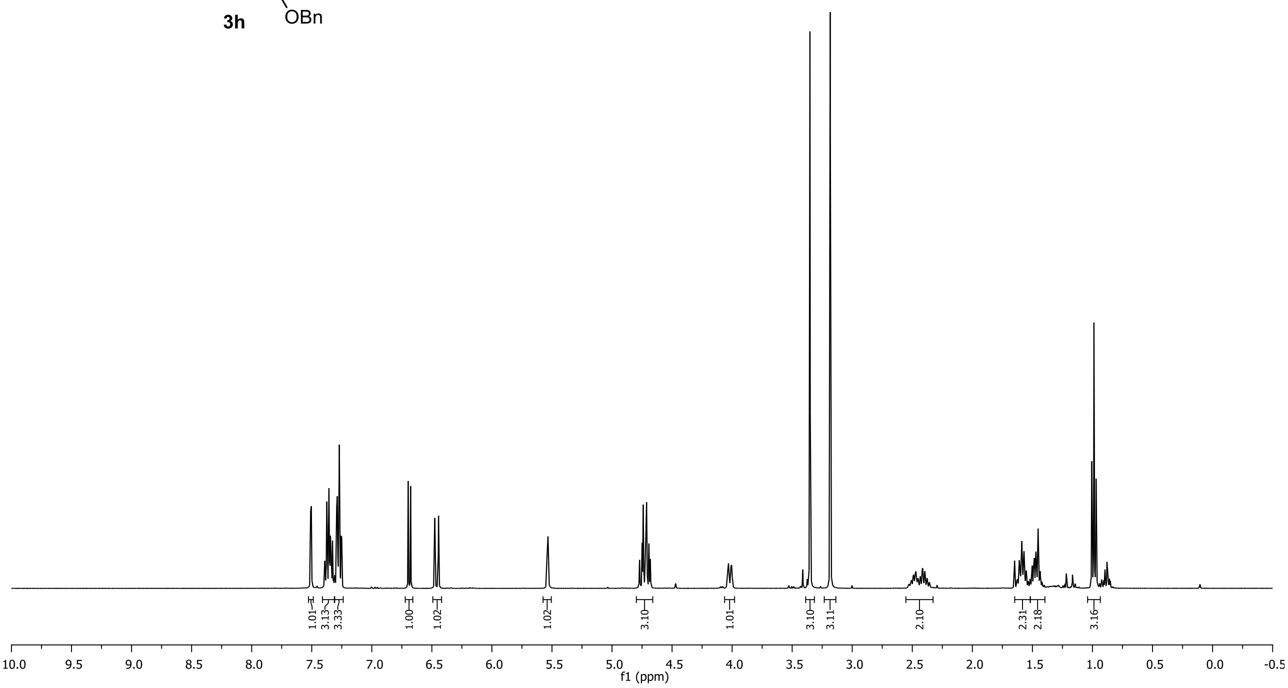
1.01
3.13
3.33
1.00
1.02
1.02
3.10
1.01
3.10
3.11
2.10
2.31
2.18
3.16
f1 (ppm)

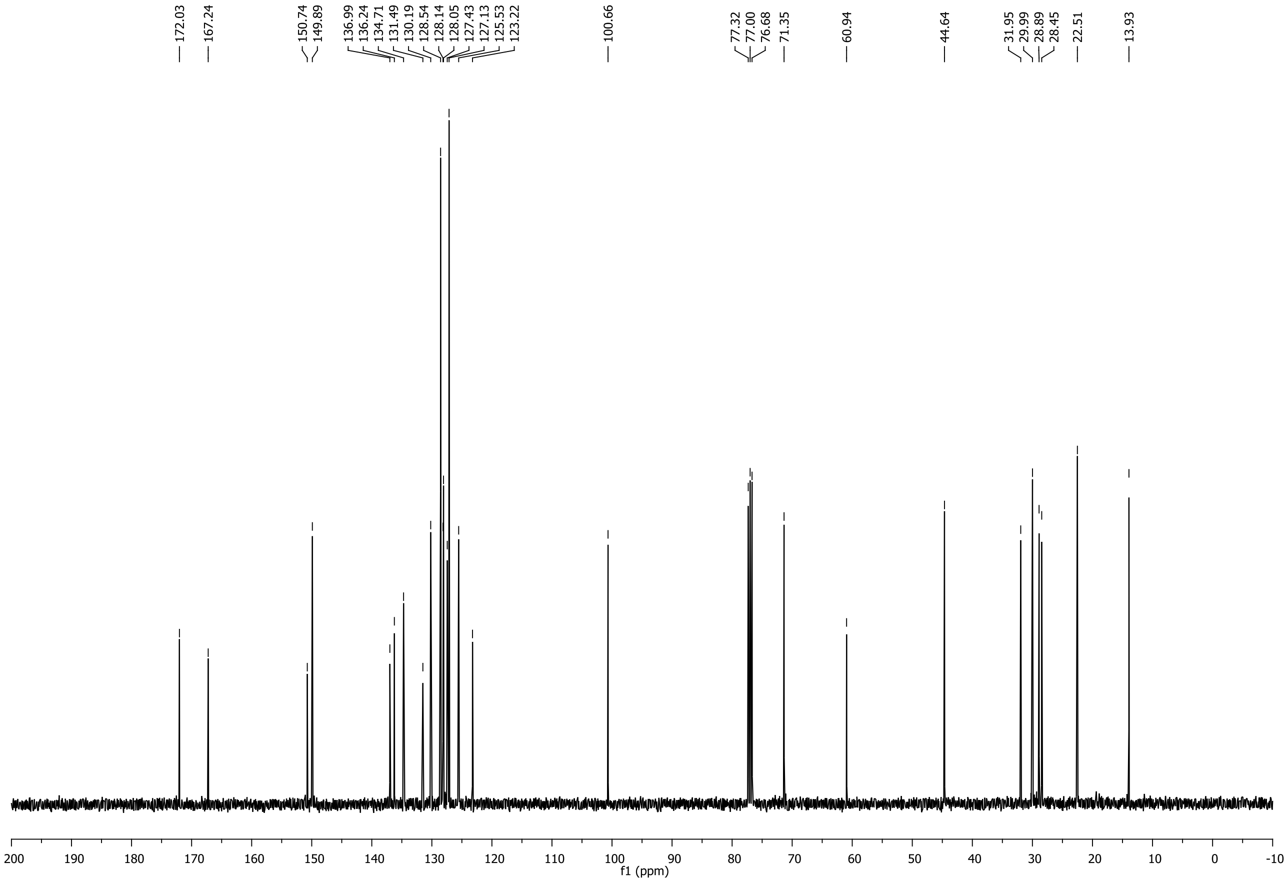
172.03
167.24
150.74
149.89
136.99
136.24
134.71
131.49
130.19
128.54
128.14
128.05
127.43
127.13
125.53
123.22
100.66
77.32
77.00
76.68
71.35
60.94
44.64
31.95
29.99
28.89
28.45
22.51
13.93
f1 (ppm)

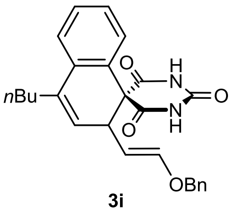
nBu
O
H
N
O
N
H
O
OBn

3i

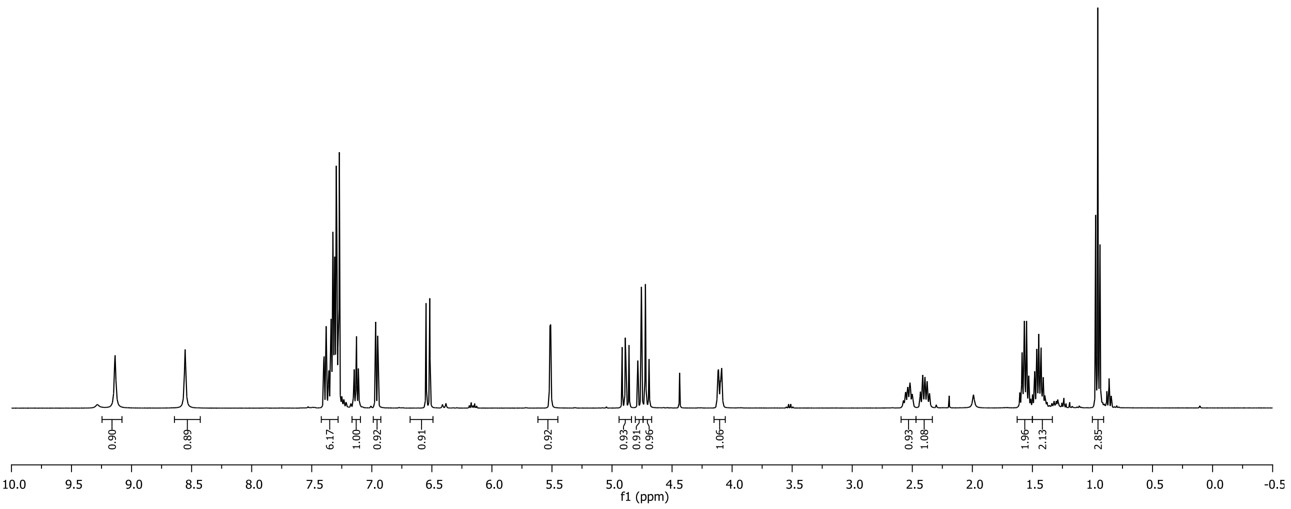
0.90
0.89
6.17
1.00
0.92
0.91
0.92
0.93
0.91
0.96
1.06
0.93
1.08
1.96
2.13
2.85
10.0 9.5 9.0 8.5 8.0 7.5 7.0 6.5 6.0 5.5 5.0 4.5 4.0 3.5 3.0 2.5 2.0 1.5 1.0 0.5 0.0 -0.5
f1 (ppm)

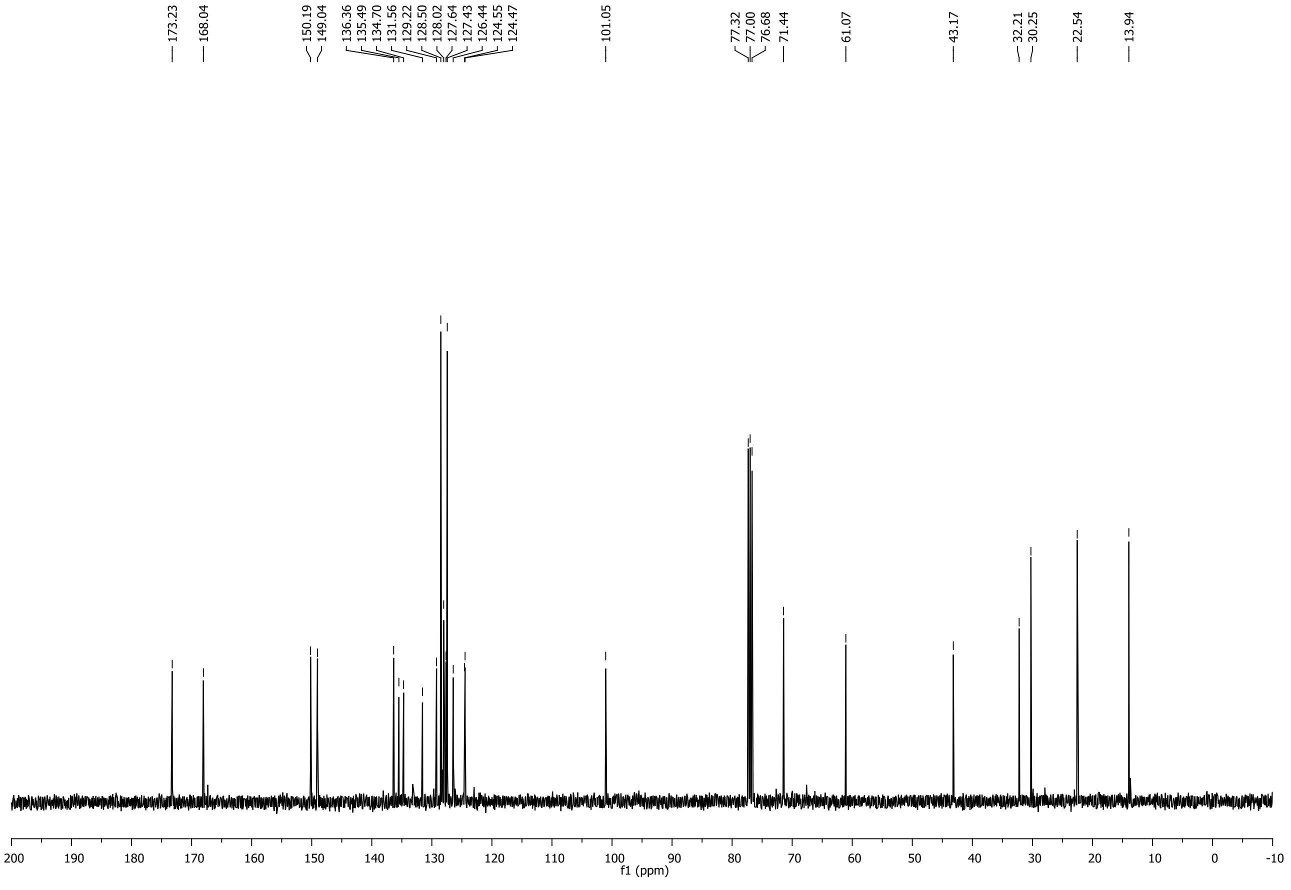
173.23
168.04
150.19
149.04
136.36
135.49
134.70
131.56
129.22
128.50
128.02
127.64
127.43
126.44
124.55
124.47
101.05
77.32
77.00
76.68
71.44
61.07
43.17
32.21
30.25
22.54
13.94
200 190 180 170 160 150 140 130 120 110 100 90 80 70 60 50 40 30 20 10 0 -10
f1 (ppm)

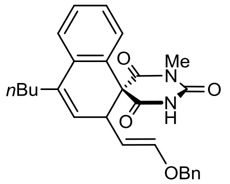
nBu
O
Me
N
O
N
H
O
OBn

3j

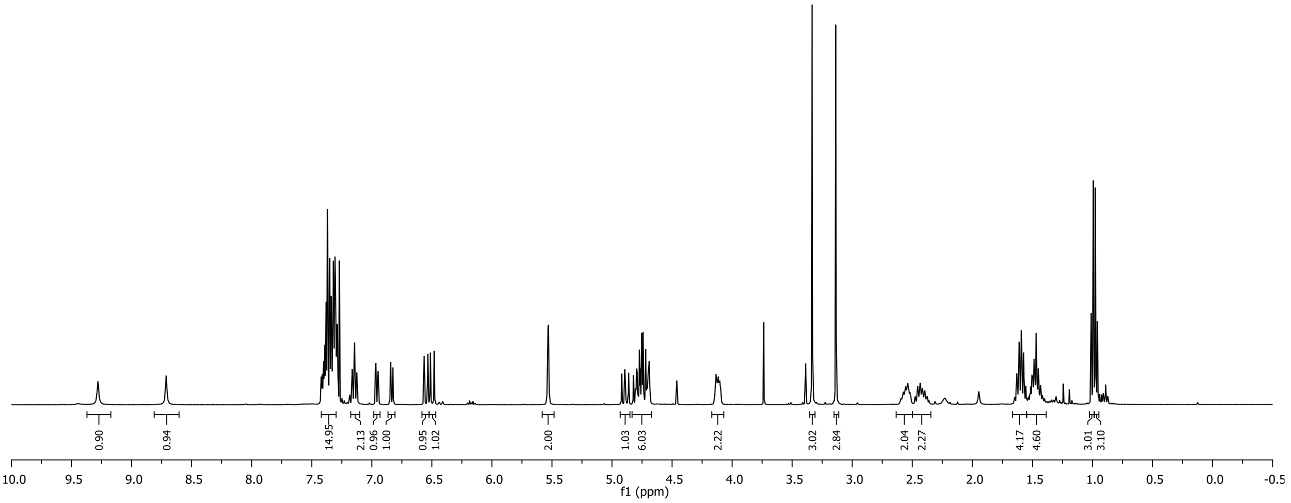
0.90
0.94
14.95
2.13
0.96
1.00
0.95
1.02
2.00
1.03
6.03
2.22
3.02
2.84
2.04
2.27
4.17
4.60
3.01
3.10
f1 (ppm)

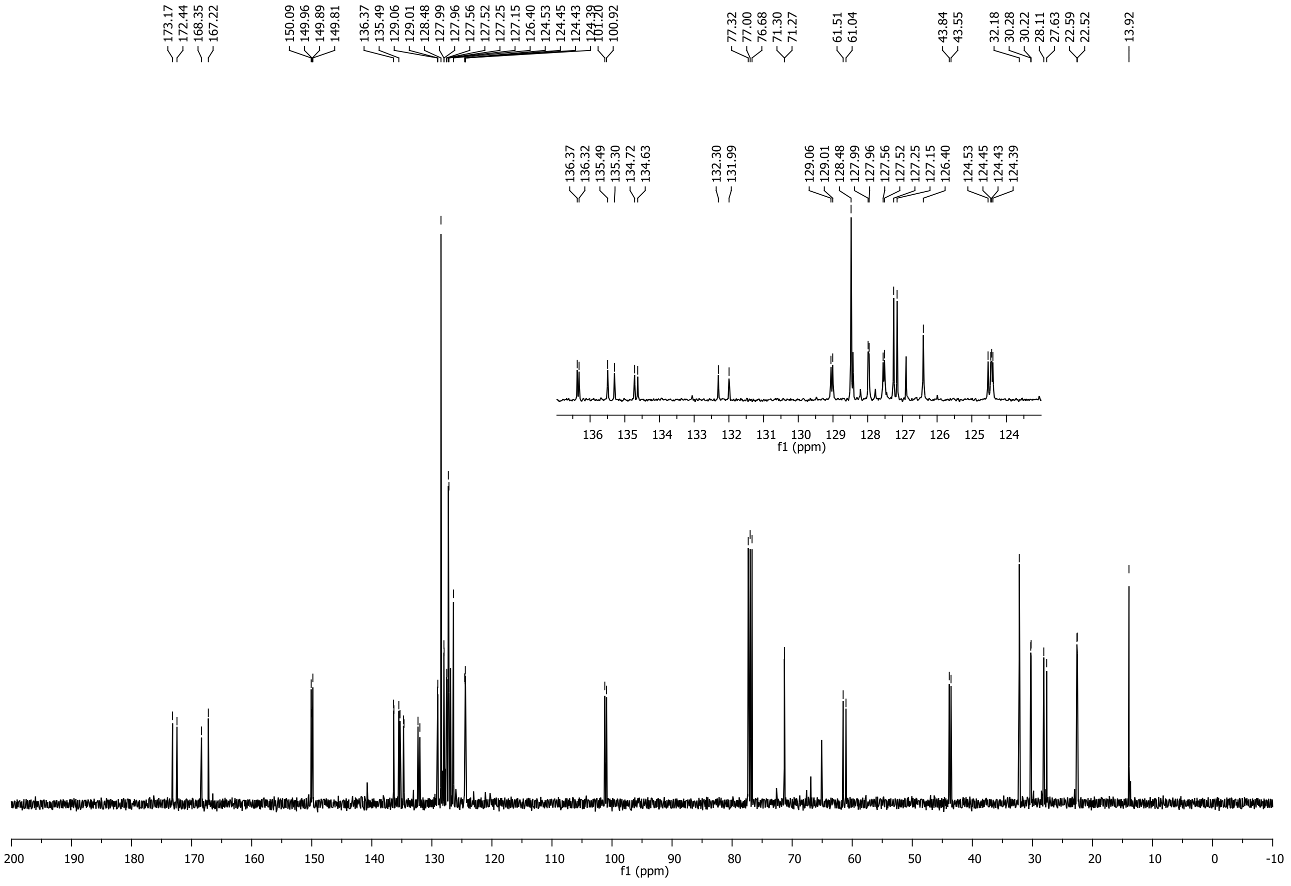
173.17
172.44
168.35
167.22
150.09
149.96
149.89
149.81
136.37
135.49
129.06
129.01
128.48
127.99
127.96
127.56
127.52
127.25
127.15
126.40
124.53
124.45
124.43
101.30
100.92
77.32
77.00
76.68
71.30
71.27
61.51
61.04
43.84
43.55
32.18
30.28
30.22
28.11
27.63
22.59
22.52
13.92
136.37
136.32
135.49
135.30
134.72
134.63
132.30
131.99
129.06
129.01
128.48
127.99
127.96
127.56
127.52
127.25
127.15
126.40
124.53
124.45
124.43
124.39
f1 (ppm)
f1 (ppm)

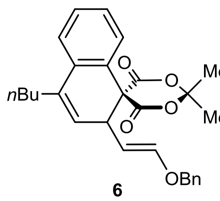
nBu
O
O
Me
O
O
Me
OBn

6

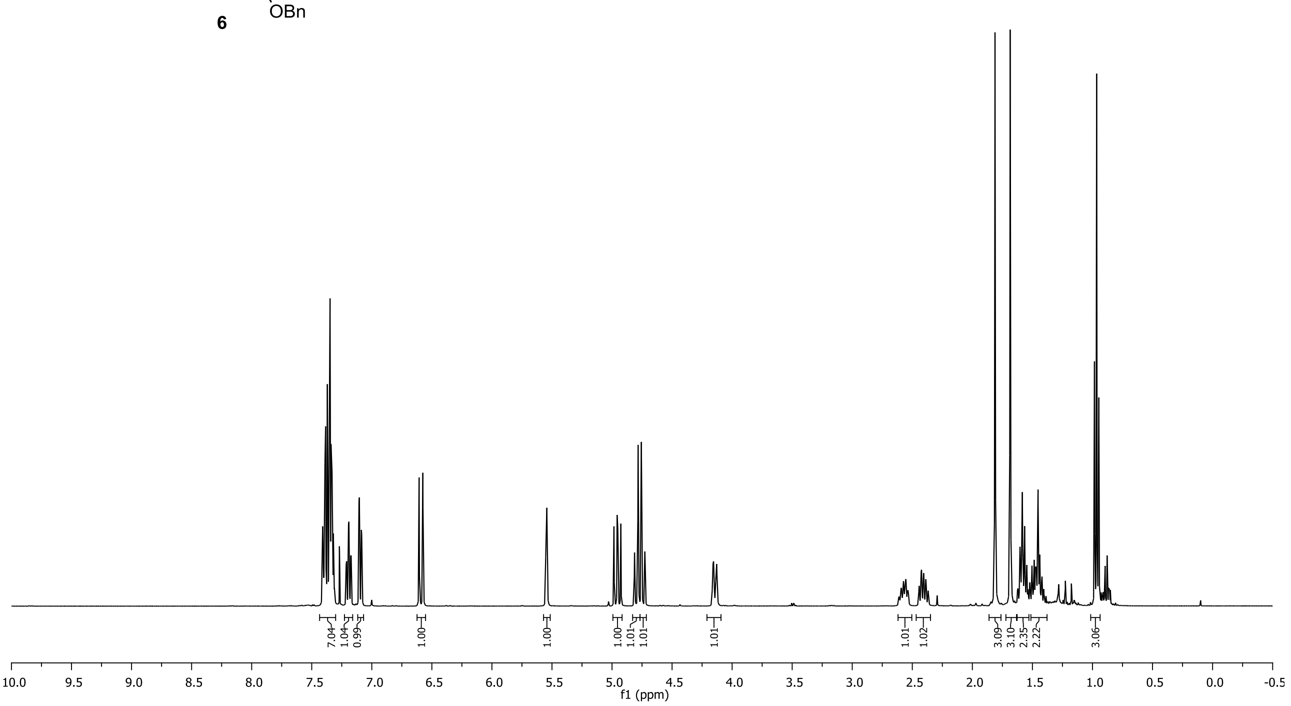
7.04
1.04
0.99
1.00
1.00
1.00
1.01
1.01
1.01
1.01
1.02
3.09
3.10
2.35
2.22
3.06
10.0 9.5 9.0 8.5 8.0 7.5 7.0 6.5 6.0 5.5 5.0 4.5 4.0 3.5 3.0 2.5 2.0 1.5 1.0 0.5 0.0 -0.5
f1 (ppm)

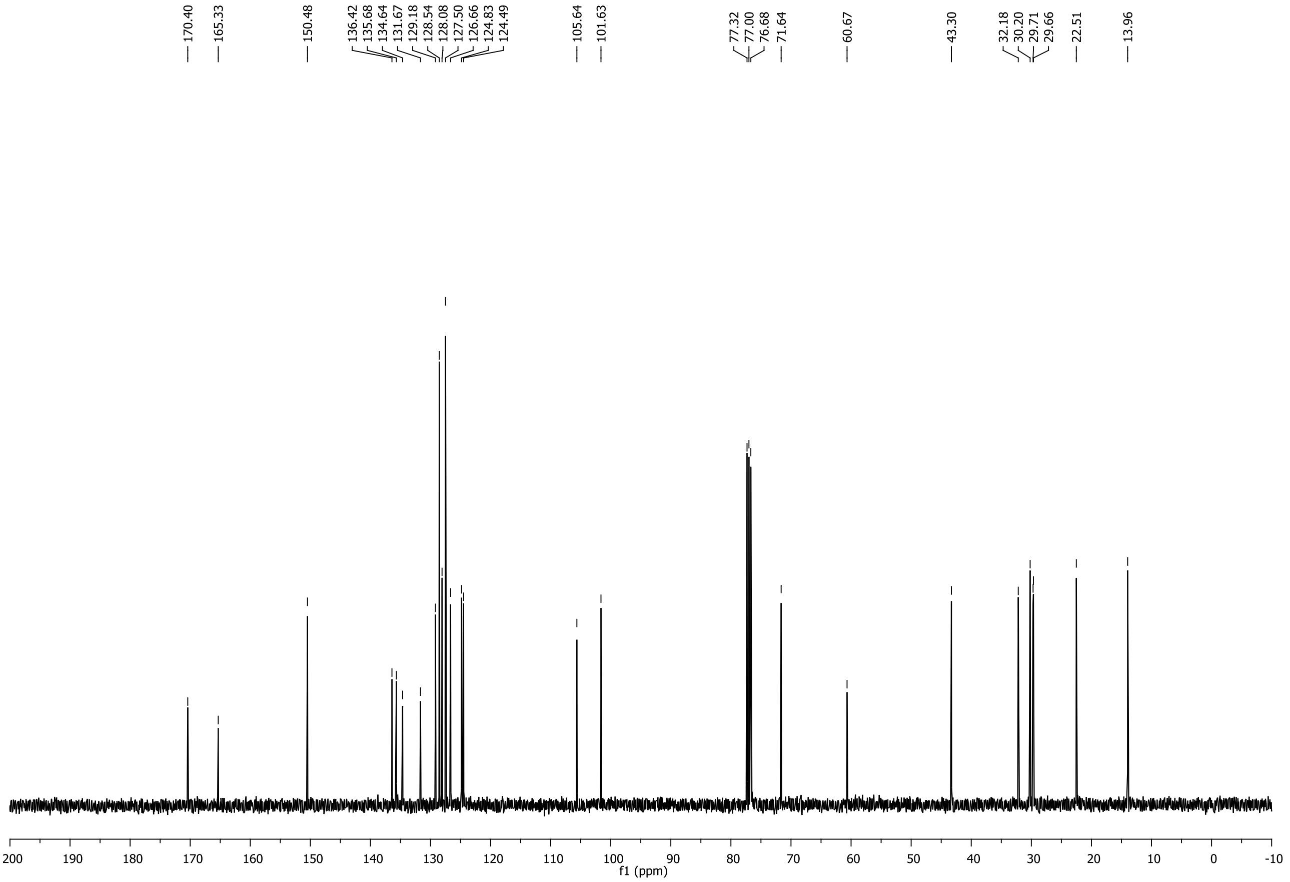
170.40
165.33
150.48
136.42
135.68
134.64
131.67
129.18
128.54
128.08
127.50
126.66
124.83
124.49
105.64
101.63
77.32
77.00
76.68
71.64
60.67
43.30
32.18
30.20
29.71
29.66
22.51
13.96
200 190 180 170 160 150 140 130 120 110 100 90 80 70 60 50 40 30 20 10 0 -10
f1 (ppm)

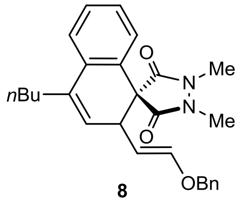

O
N
Me
*n*Bu
N
Me
O
OBn

8

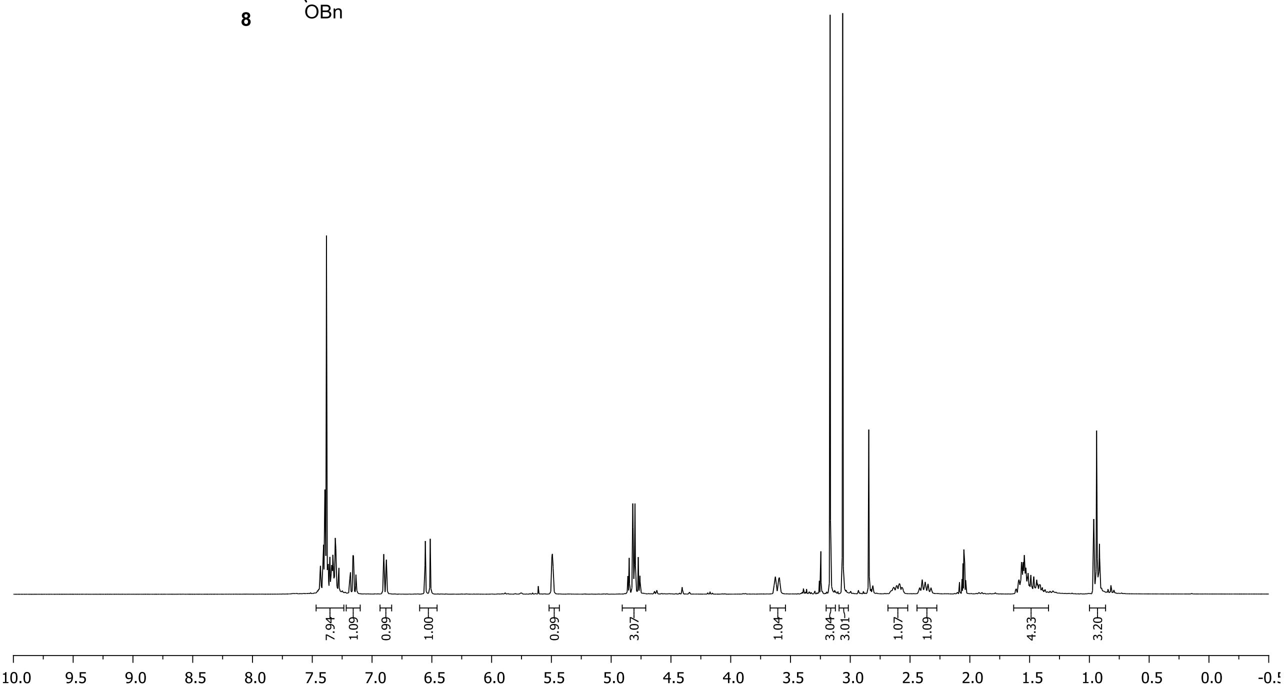

7.94
1.09
0.99
1.00
0.99
3.07
1.04
3.04
3.01
1.07
1.09
4.33
3.20
10.0 9.5 9.0 8.5 8.0 7.5 7.0 6.5 6.0 5.5 5.0 4.5 4.0 3.5 3.0 2.5 2.0 1.5 1.0 0.5 0.0 -0.5

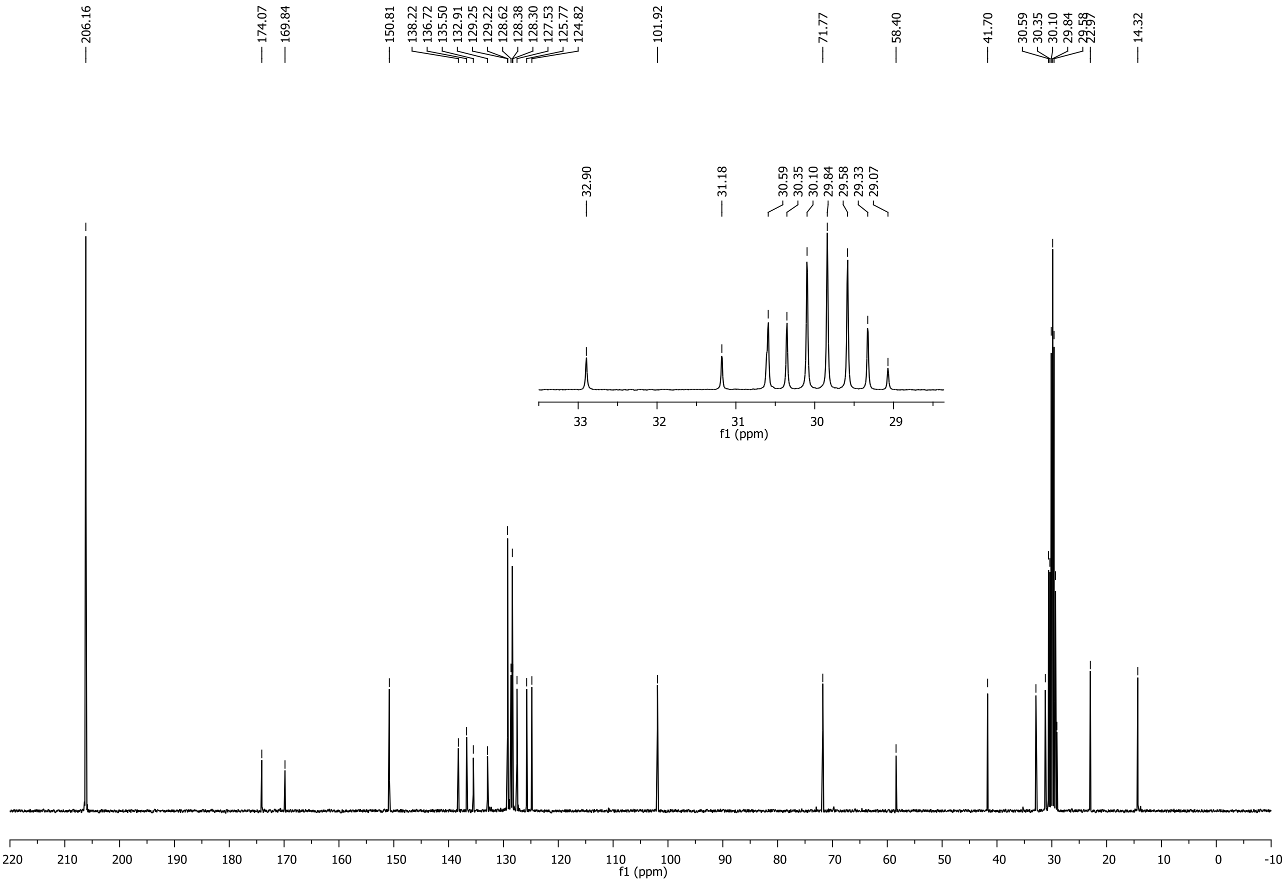

206.16
174.07
169.84
150.81
138.22
136.72
135.50
132.91
129.25
129.22
128.62
128.38
128.30
127.53
125.77
124.82
101.92
71.77
58.40
41.70
30.59
30.35
30.10
29.84
29.58
22.97
14.32
32.90
31.18
30.59
30.35
30.10
29.84
29.58
29.33
29.07
33 32 31 30 29
f1 (ppm)
220 210 200 190 180 170 160 150 140 130 120 110 100 90 80 70 60 50 40 30 20 10 0 -10
f1 (ppm)

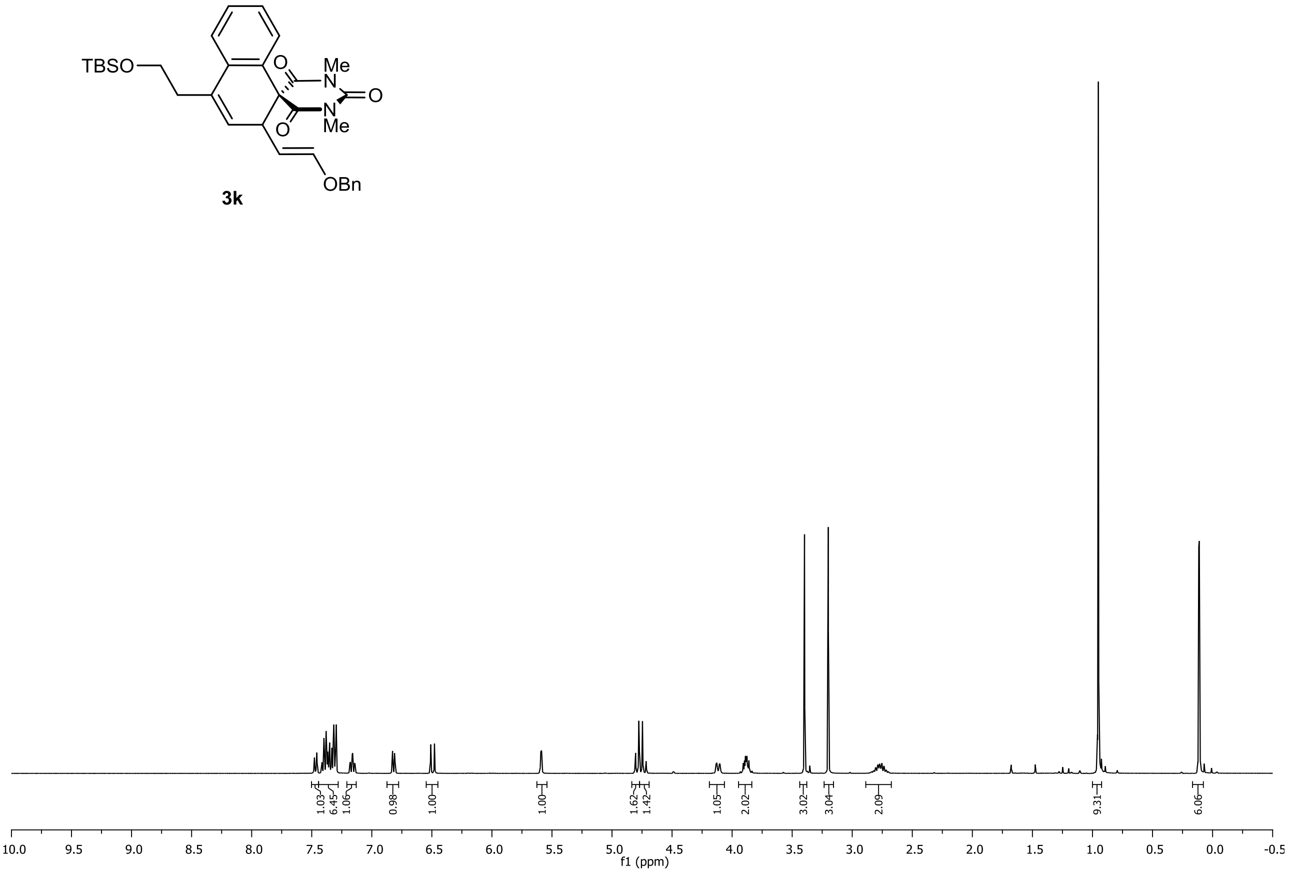
TBSO
O
Me
N
O
N
Me
O
OBn
3k
1.03
6.45
1.06
0.98
1.00
1.00
1.62
1.42
1.05
2.02
3.02
3.04
2.09
9.31
6.06
10.0 9.5 9.0 8.5 8.0 7.5 7.0 6.5 6.0 5.5 5.0 4.5 4.0 3.5 3.0 2.5 2.0 1.5 1.0 0.5 0.0 -0.5
f1 (ppm)

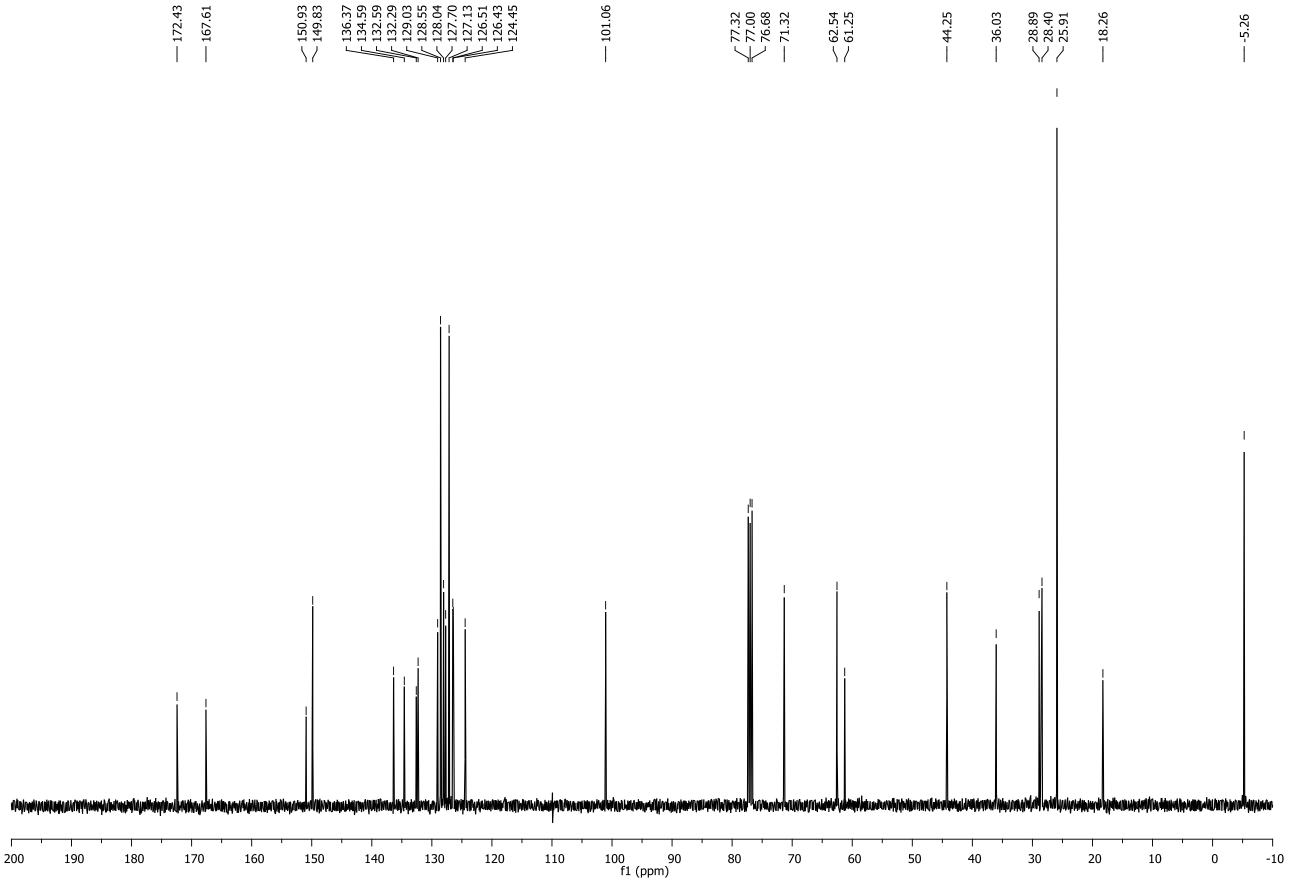
172.43
167.61
150.93
149.83
136.37
134.59
132.59
132.29
129.03
128.55
128.04
127.70
127.13
126.51
126.43
124.45
101.06
77.32
77.00
76.68
71.32
62.54
61.25
44.25
36.03
28.89
28.40
25.91
18.26
-5.26
200 190 180 170 160 150 140 130 120 110 100 90 80 70 60 50 40 30 20 10 0 -10
f1 (ppm)

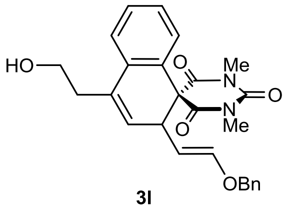
HO
O
Me
N
O
N
O
Me
OBn

3l

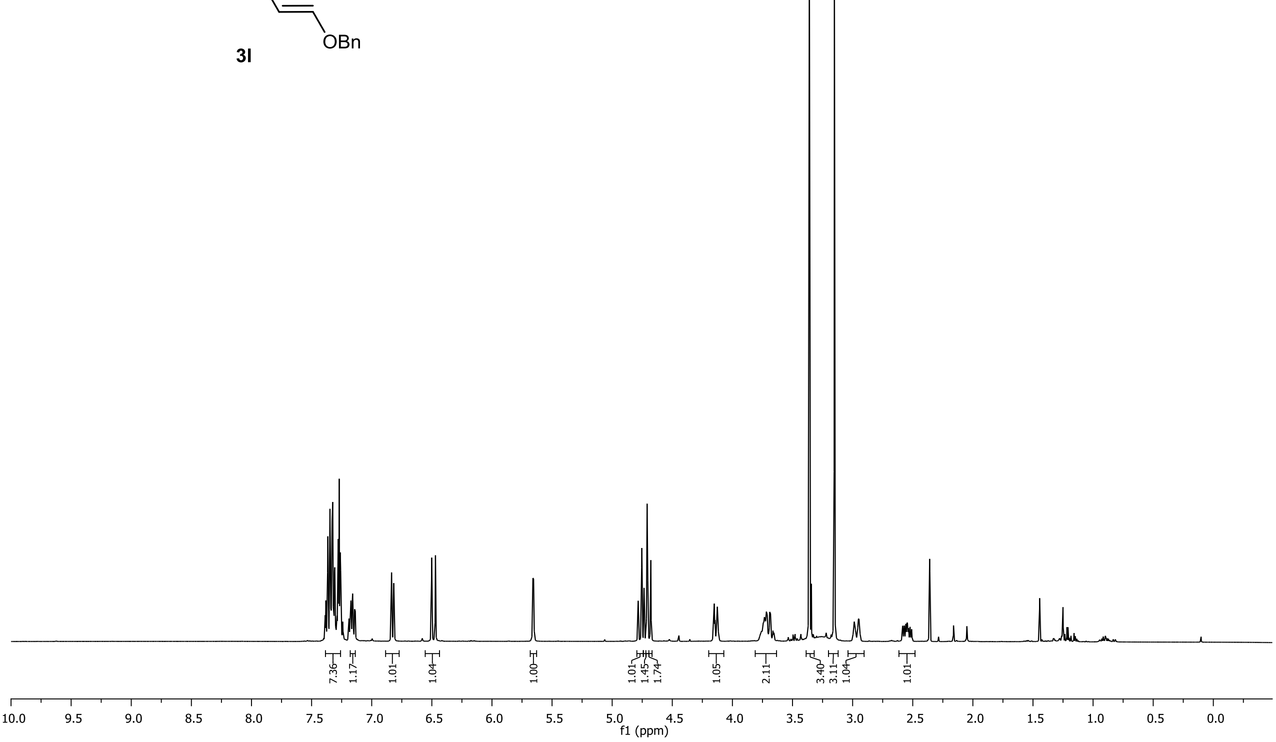
7.36
1.17
1.01
1.04
1.00
1.01
1.45
1.74
1.05
2.11
3.40
3.11
1.04
1.01
10.0 9.5 9.0 8.5 8.0 7.5 7.0 6.5 6.0 5.5 5.0 4.5 4.0 3.5 3.0 2.5 2.0 1.5 1.0 0.5 0.0
f1 (ppm)

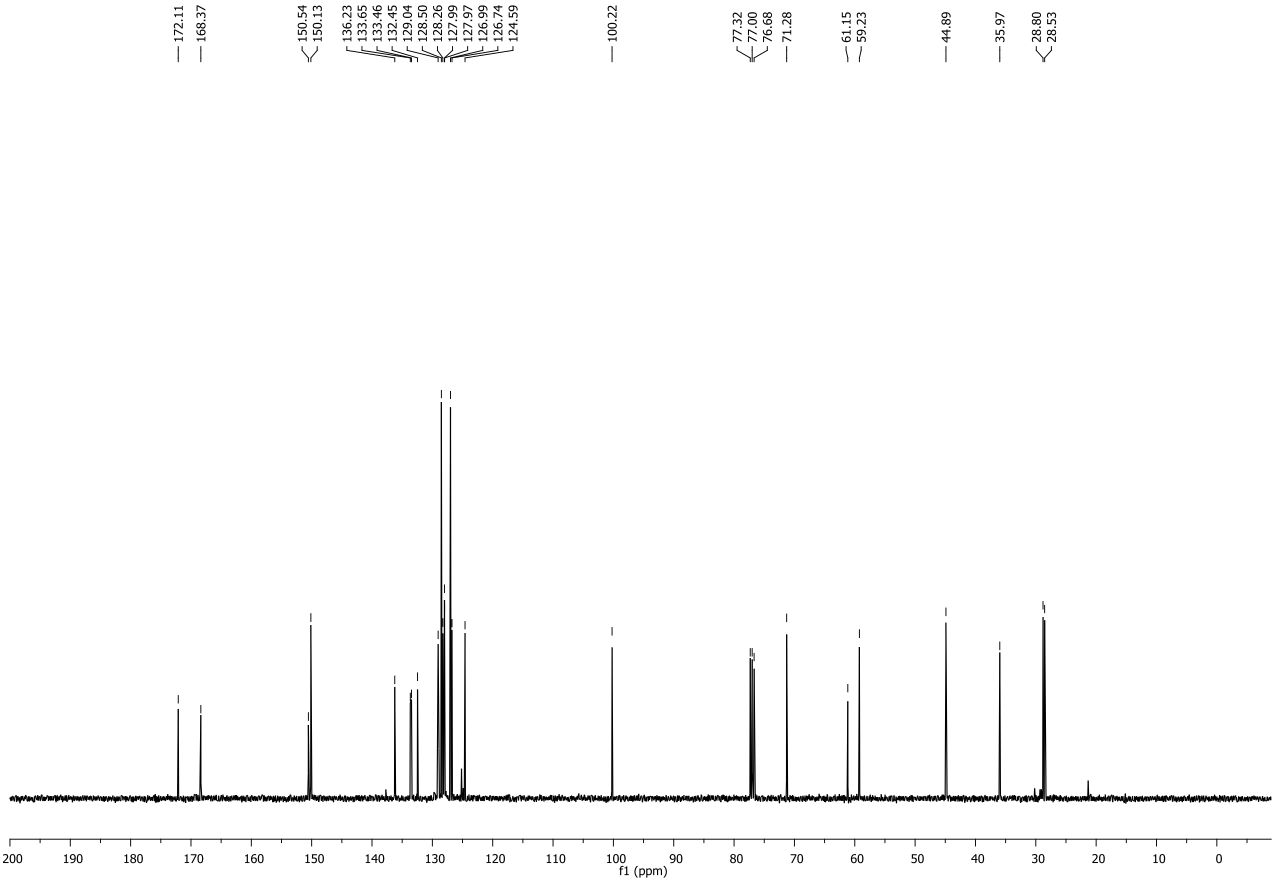
172.11
168.37
150.54
150.13
136.23
133.65
133.46
132.45
129.04
128.50
128.26
127.99
127.97
126.99
126.74
124.59
100.22
77.32
77.00
76.68
71.28
61.15
59.23
44.89
35.97
28.80
28.53
200 190 180 170 160 150 140 130 120 110 100 90 80 70 60 50 40 30 20 10 0
f1 (ppm)

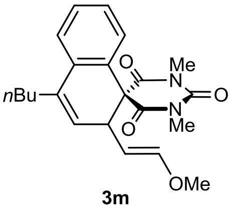
nBu
O
Me
N
O
N
O
Me
OMe

3m

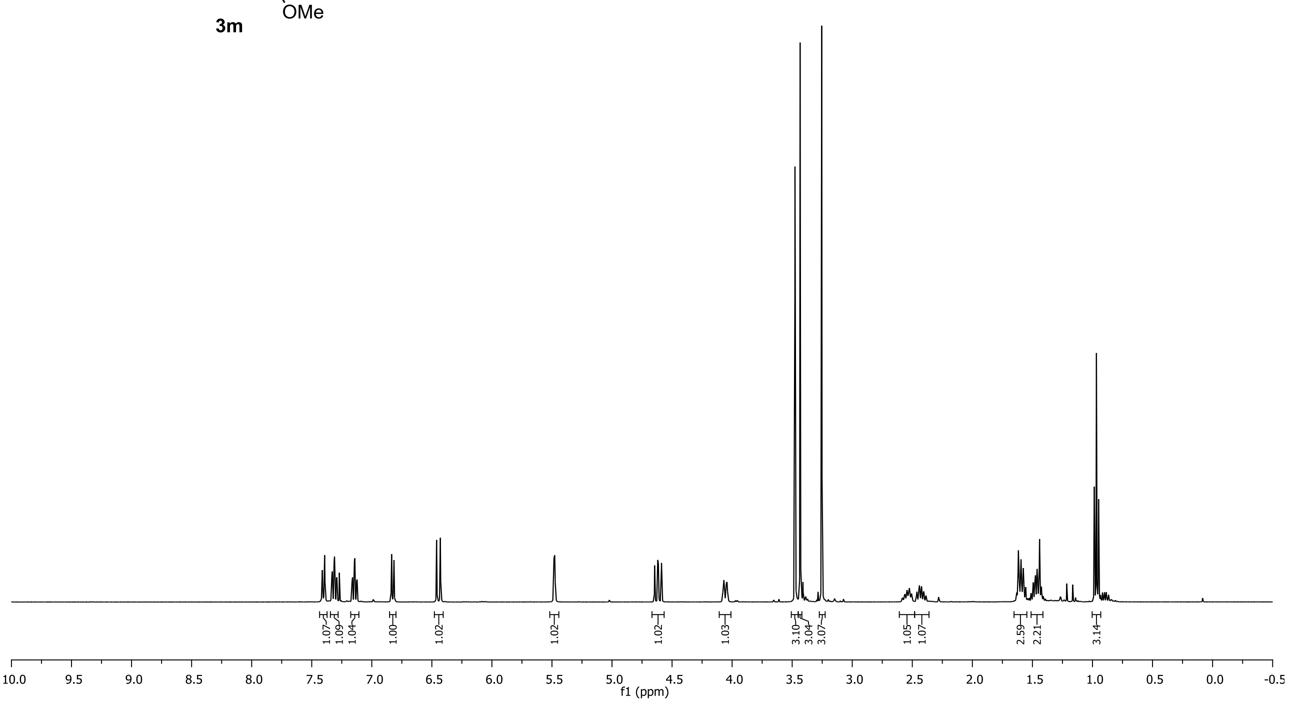
1.07
1.09
1.04
1.00
1.02
1.02
1.02
1.03
3.10
3.04
3.07
1.05
1.07
2.59
2.21
3.14
10.0 9.5 9.0 8.5 8.0 7.5 7.0 6.5 6.0 5.5 5.0 4.5 4.0 3.5 3.0 2.5 2.0 1.5 1.0 0.5 0.0 -0.5
f1 (ppm)

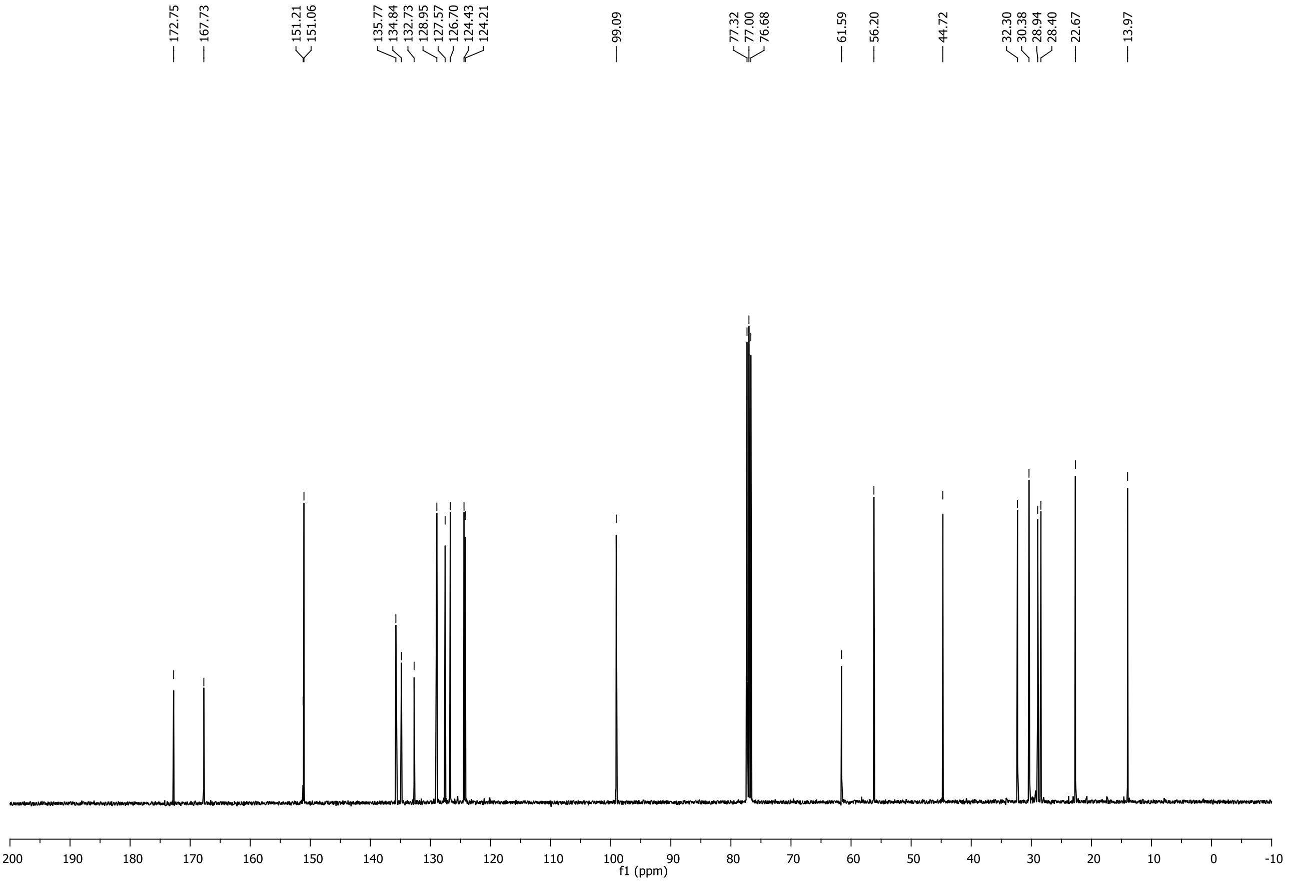
172.75
167.73
151.21
151.06
135.77
134.84
132.73
128.95
127.57
126.70
124.43
124.21
99.09
77.32
77.00
76.68
61.59
56.20
44.72
32.30
30.38
28.94
28.40
22.67
13.97
200 190 180 170 160 150 140 130 120 110 100 90 80 70 60 50 40 30 20 10 0 -10
f1 (ppm)

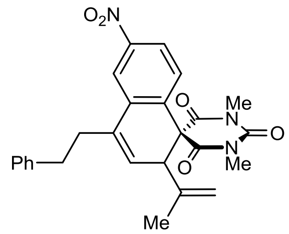
O2N
Me
N
O
O
N
O
Me
Ph
Me

3n

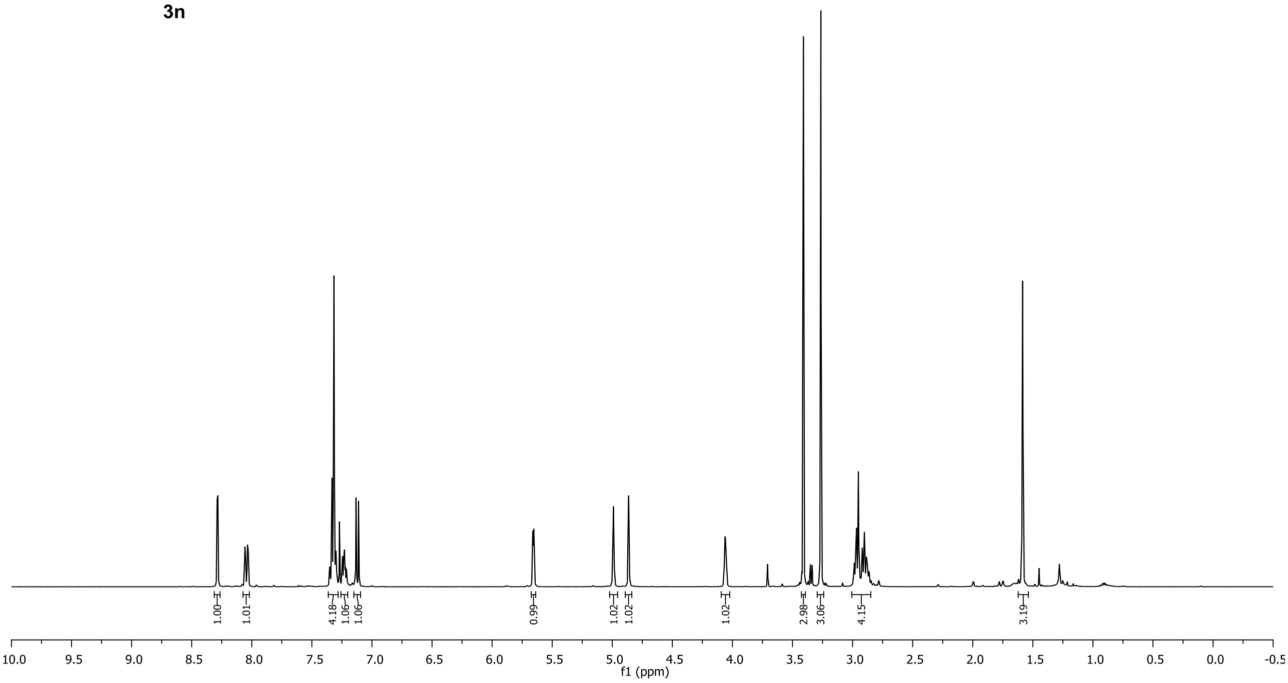
1.00
1.01
4.18
1.06
1.06
0.99
1.02
1.02
1.02
2.98
3.06
4.15
3.19
f1 (ppm)

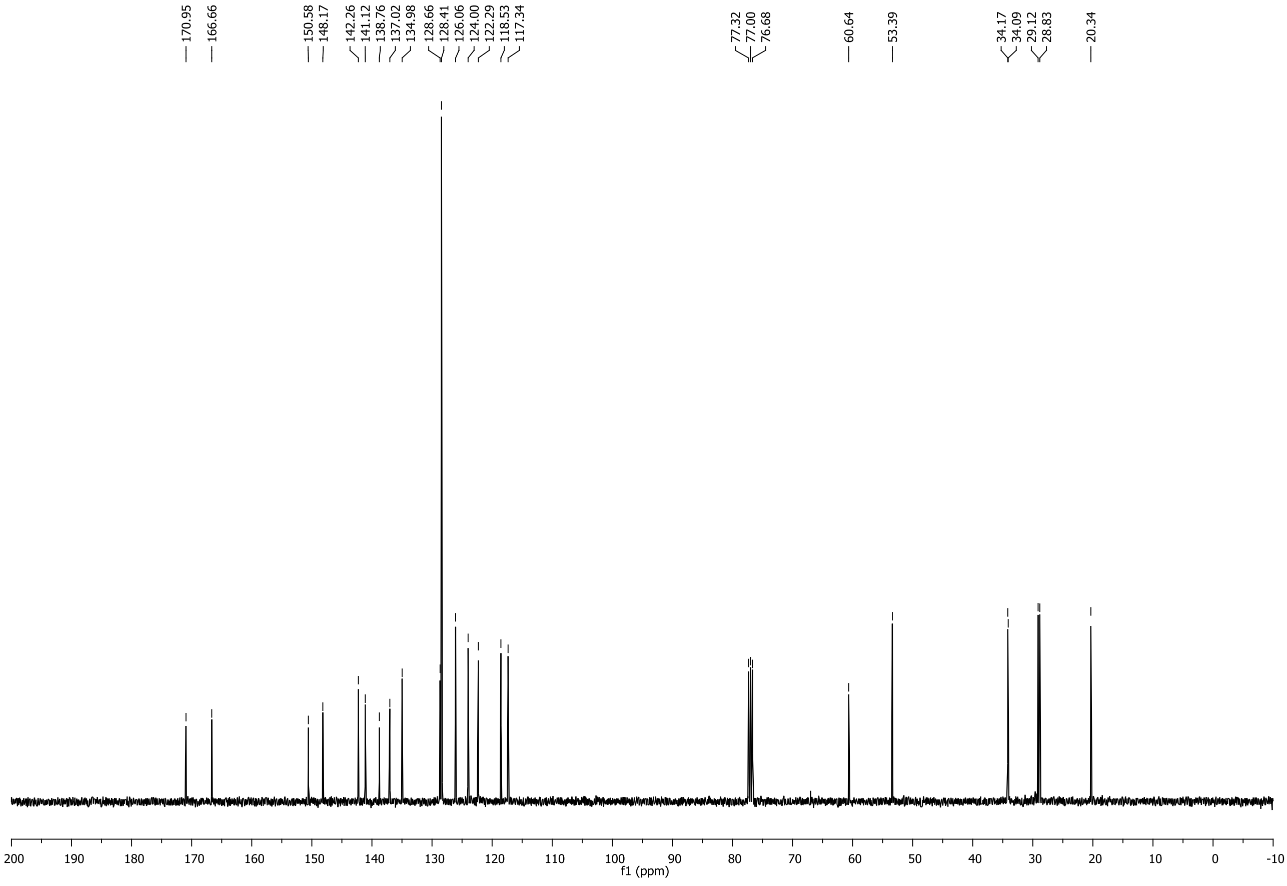
170.95
166.66
150.58
148.17
142.26
141.12
138.76
137.02
134.98
128.66
128.41
126.06
124.00
122.29
118.53
117.34
77.32
77.00
76.68
60.64
53.39
34.17
34.09
29.12
28.83
20.34
f1 (ppm)

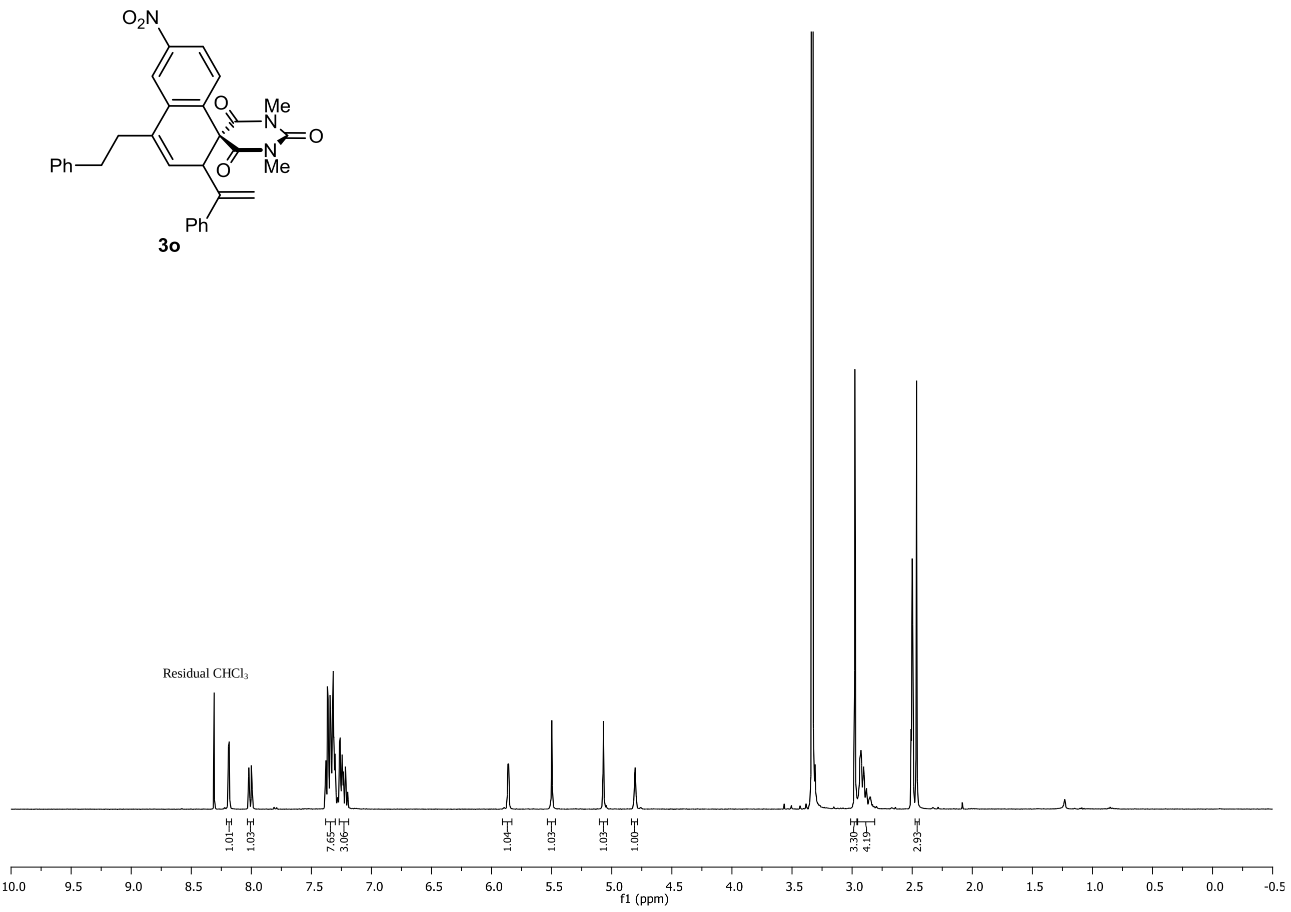
O2N
Me
N
O
Me
Ph
Ph

3o

Residual CHCl3
1.01
1.03
7.65
3.06
1.04
1.03
1.03
1.00
3.30
4.19
2.93
f1 (ppm)

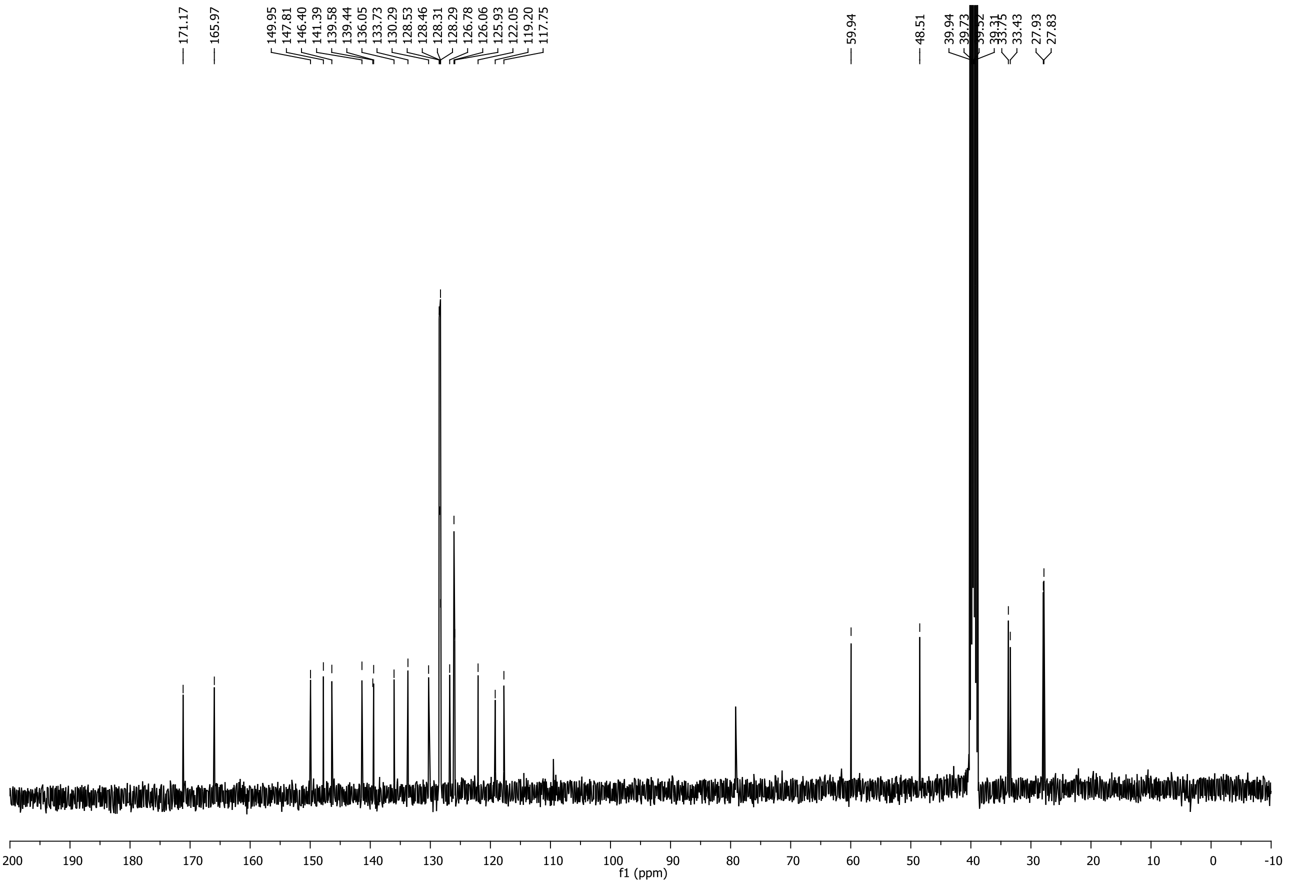
171.17
165.97
149.95
147.81
146.40
141.39
139.58
139.44
136.05
133.73
130.29
128.53
128.46
128.31
128.29
126.78
126.06
125.93
122.05
119.20
117.75
59.94
48.51
39.94
39.73
33.75
33.43
27.93
27.83
f1 (ppm)

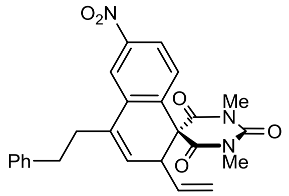
O2N
Me
N
O
Ph
Me

3p

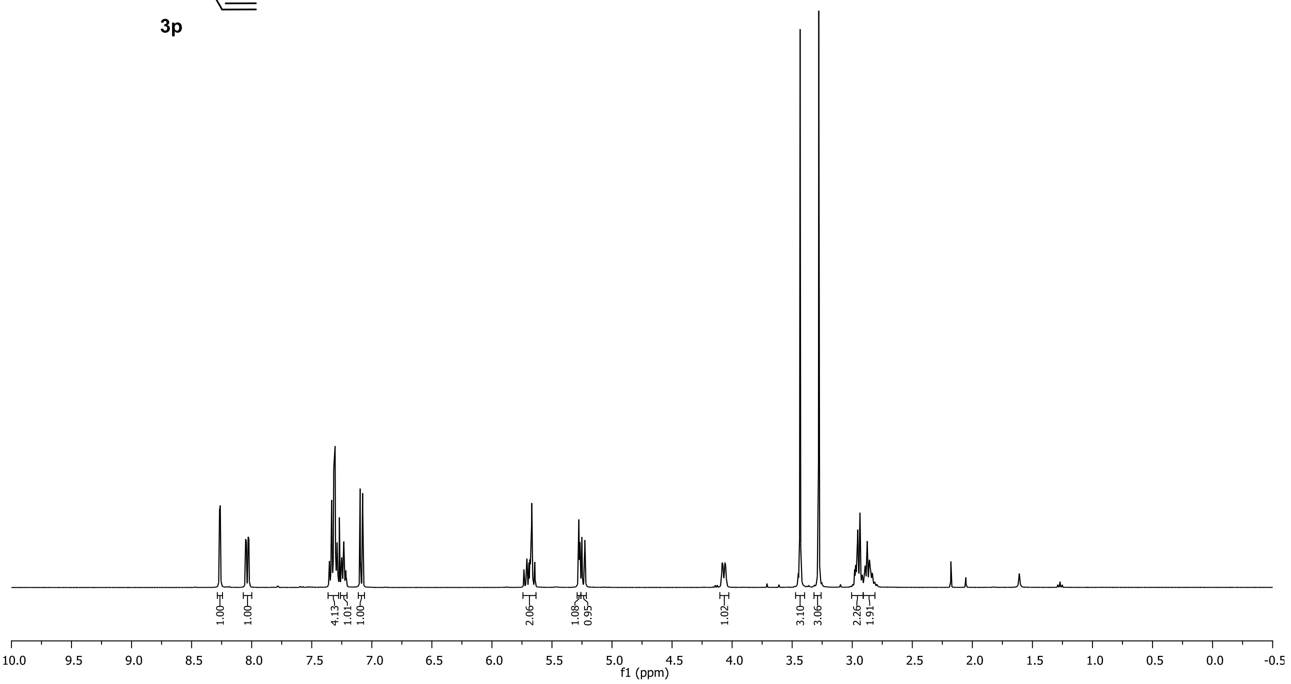
1.00
1.00
4.13
1.01
1.00
2.06
1.08
0.95
1.02
3.10
3.06
2.26
1.91
10.0 9.5 9.0 8.5 8.0 7.5 7.0 6.5 6.0 5.5 5.0 4.5 4.0 3.5 3.0 2.5 2.0 1.5 1.0 0.5 0.0 -0.5
f1 (ppm)

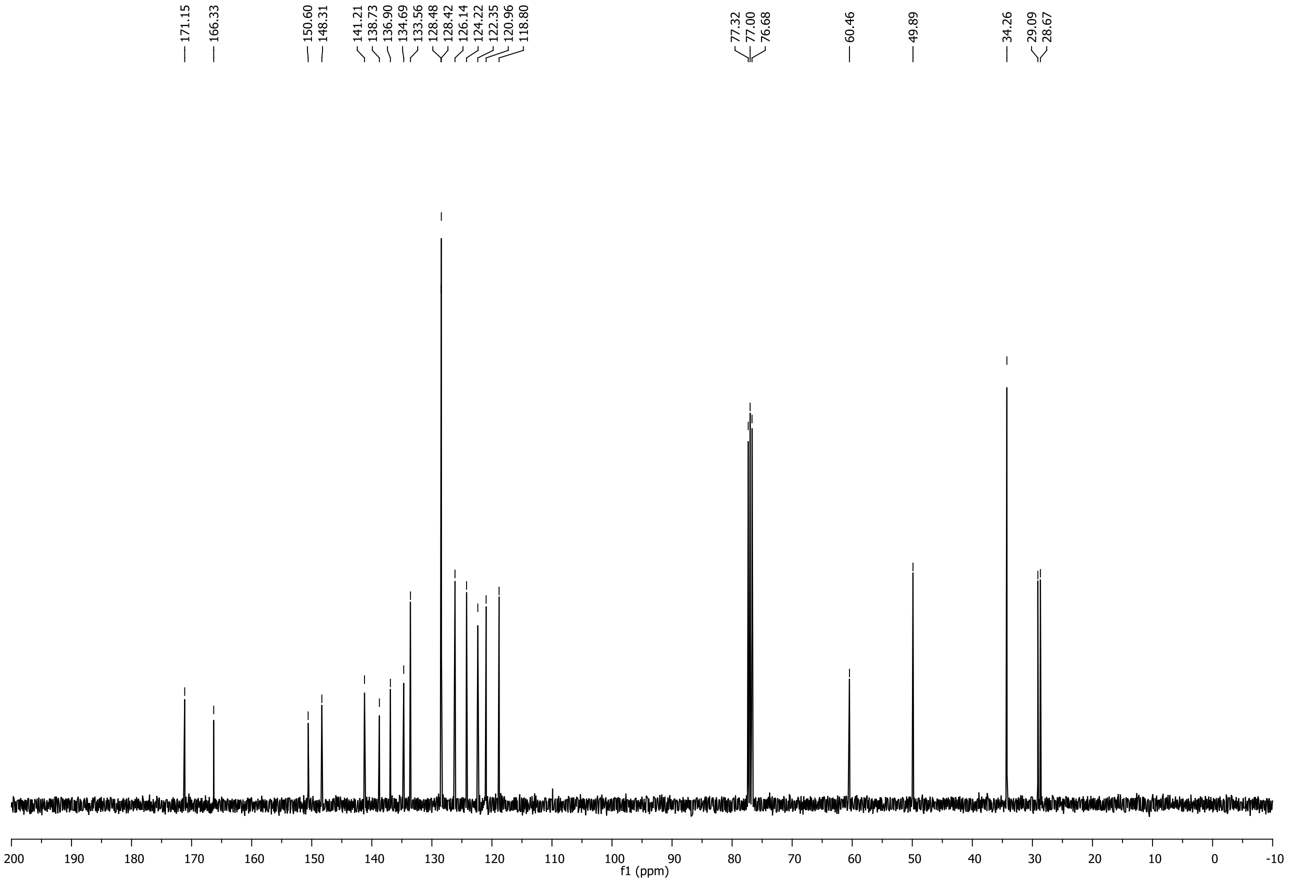
171.15
166.33
150.60
148.31
141.21
138.73
136.90
134.69
133.56
128.48
128.42
126.14
124.22
122.35
120.96
118.80
77.32
77.00
76.68
60.46
49.89
34.26
29.09
28.67
200 190 180 170 160 150 140 130 120 110 100 90 80 70 60 50 40 30 20 10 0 -10
f1 (ppm)

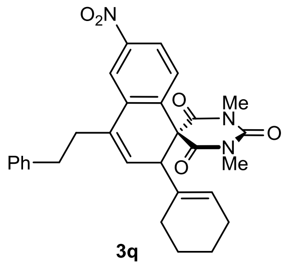
O2N
O
Me
N
O
N
O
Me
Ph

3q

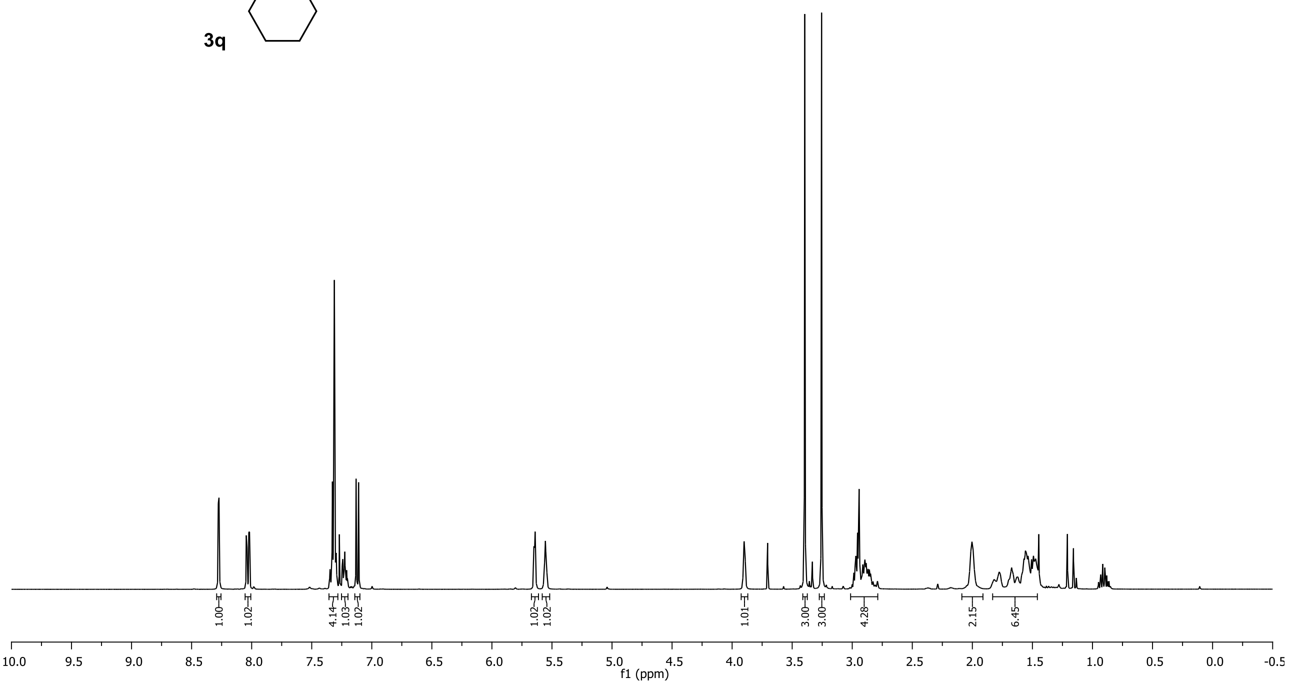
1.00
1.02
4.14
1.03
1.02
1.02
1.02
1.01
3.00
3.00
4.28
2.15
6.45
10.0 9.5 9.0 8.5 8.0 7.5 7.0 6.5 6.0 5.5 5.0 4.5 4.0 3.5 3.0 2.5 2.0 1.5 1.0 0.5 0.0 -0.5
f1 (ppm)

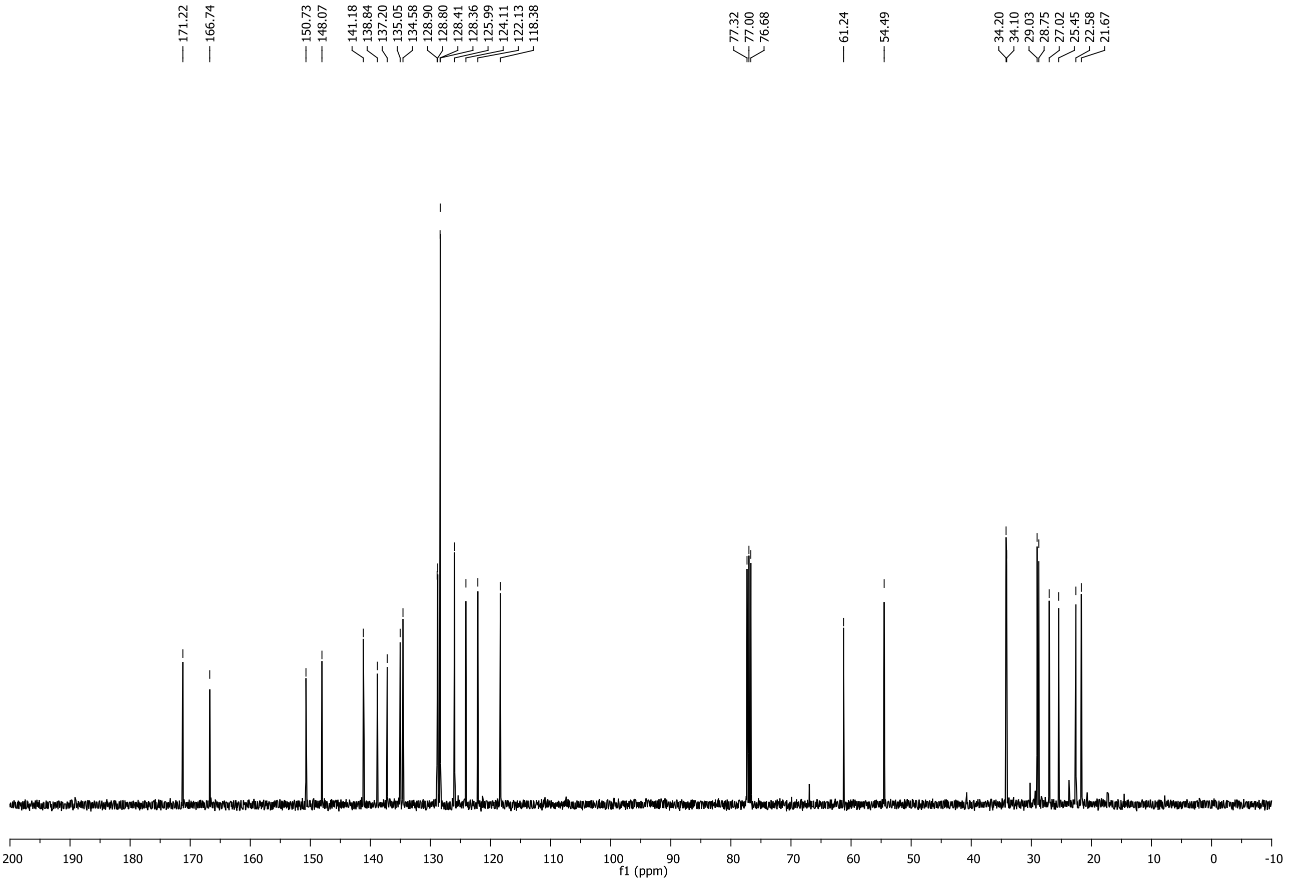
171.22
166.74
150.73
148.07
141.18
138.84
137.20
135.05
134.58
128.90
128.80
128.41
128.36
125.99
124.11
122.13
118.38
77.32
77.00
76.68
61.24
54.49
34.20
34.10
29.03
28.75
27.02
25.45
22.58
21.67
200 190 180 170 160 150 140 130 120 110 100 90 80 70 60 50 40 30 20 10 0 -10
f1 (ppm)

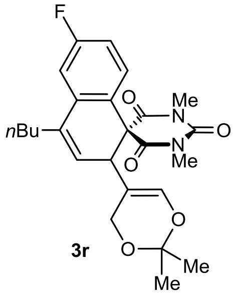
F
nBu
O
Me
N
O
N
Me
O
O
O
Me
Me

3r

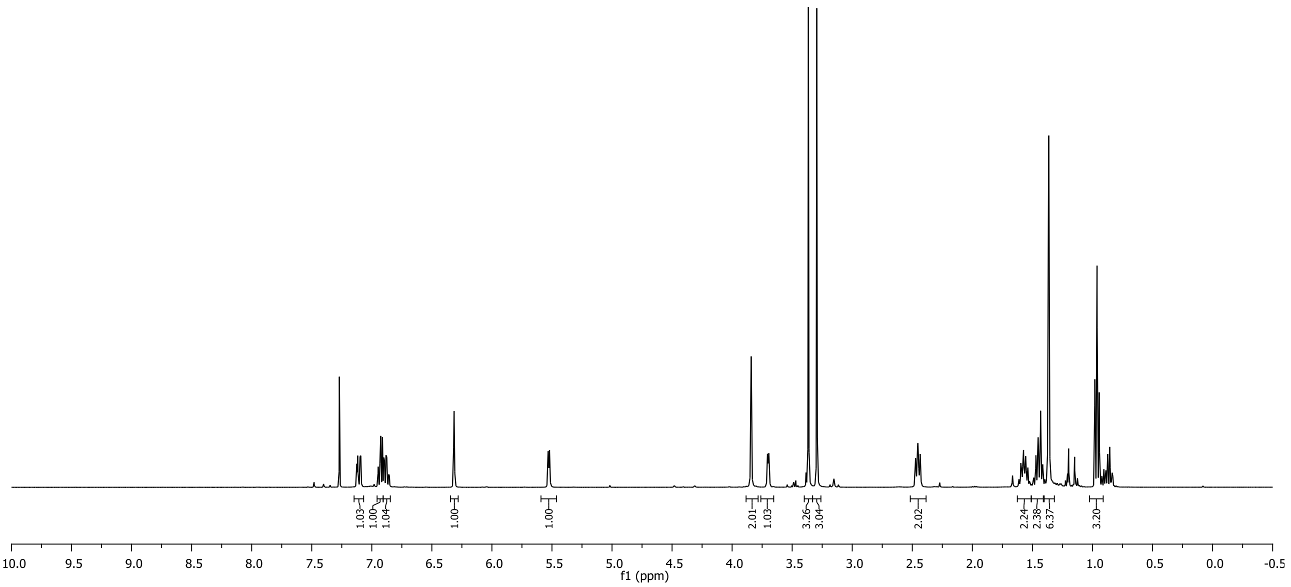
1.03
1.00
1.04
1.00
1.00
2.01
1.03
3.26
3.04
2.02
2.24
2.38
6.37
3.20
10.0 9.5 9.0 8.5 8.0 7.5 7.0 6.5 6.0 5.5 5.0 4.5 4.0 3.5 3.0 2.5 2.0 1.5 1.0 0.5 0.0 -0.5
f1 (ppm)

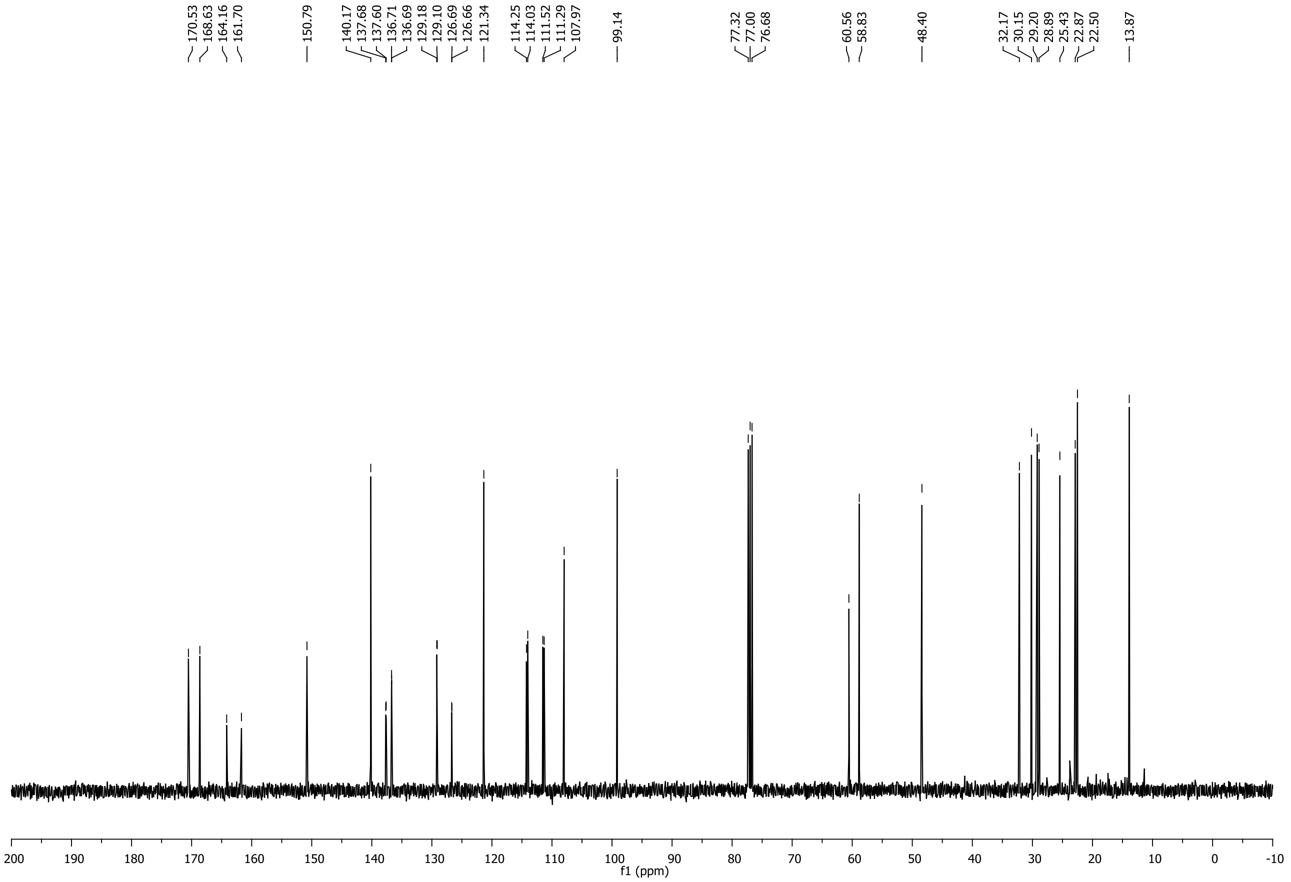
170.53
168.63
164.16
161.70
150.79
140.17
137.68
137.60
136.71
136.69
129.18
129.10
126.69
126.66
121.34
114.25
114.03
111.52
111.29
107.97
99.14
77.32
77.00
76.68
60.56
58.83
48.40
32.17
30.15
29.20
28.89
25.43
22.87
22.50
13.87
200 190 180 170 160 150 140 130 120 110 100 90 80 70 60 50 40 30 20 10 0 -10
f1 (ppm)

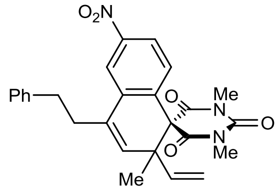

3s

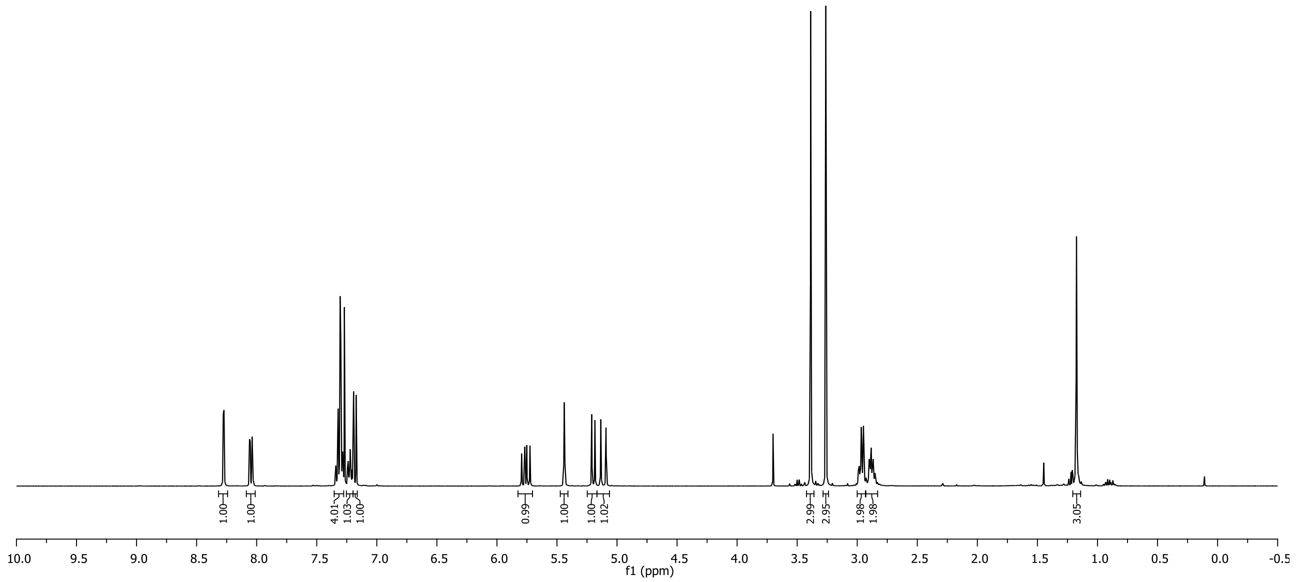

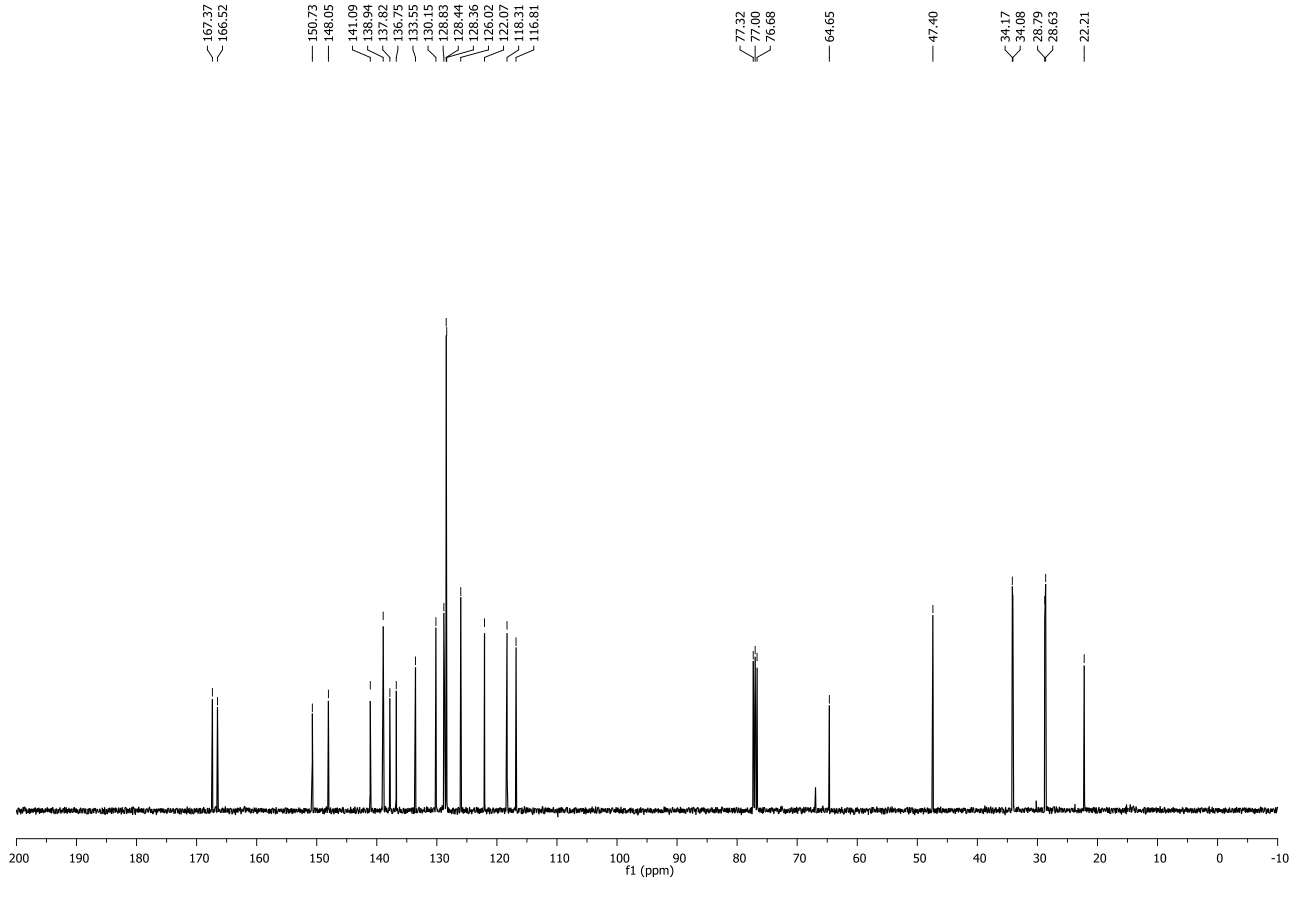

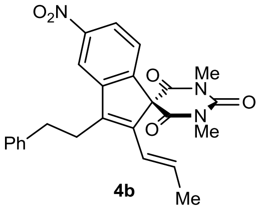
O2N
Me
N
O
N
Me
O
O
Ph
Me

4b

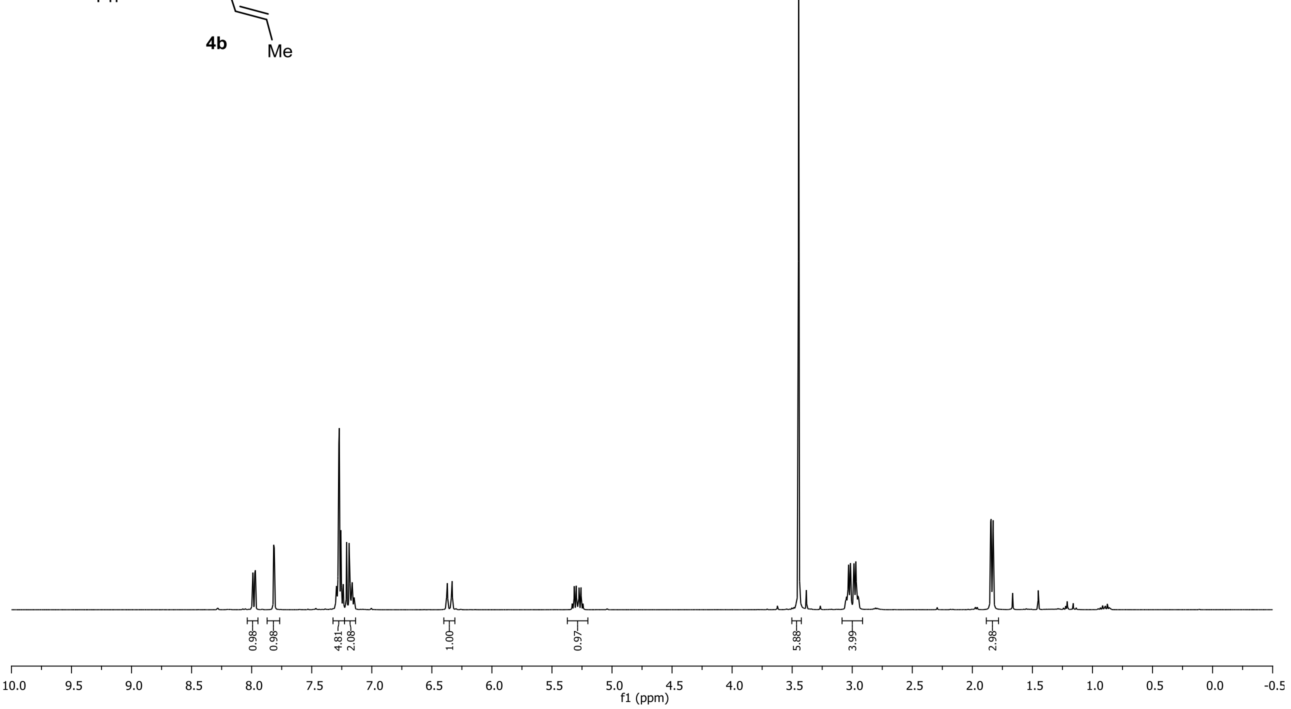
0.98
0.98
4.81
2.08
1.00
0.97
5.88
3.99
2.98
10.0 9.5 9.0 8.5 8.0 7.5 7.0 6.5 6.0 5.5 5.0 4.5 4.0 3.5 3.0 2.5 2.0 1.5 1.0 0.5 0.0 -0.5
f1 (ppm)

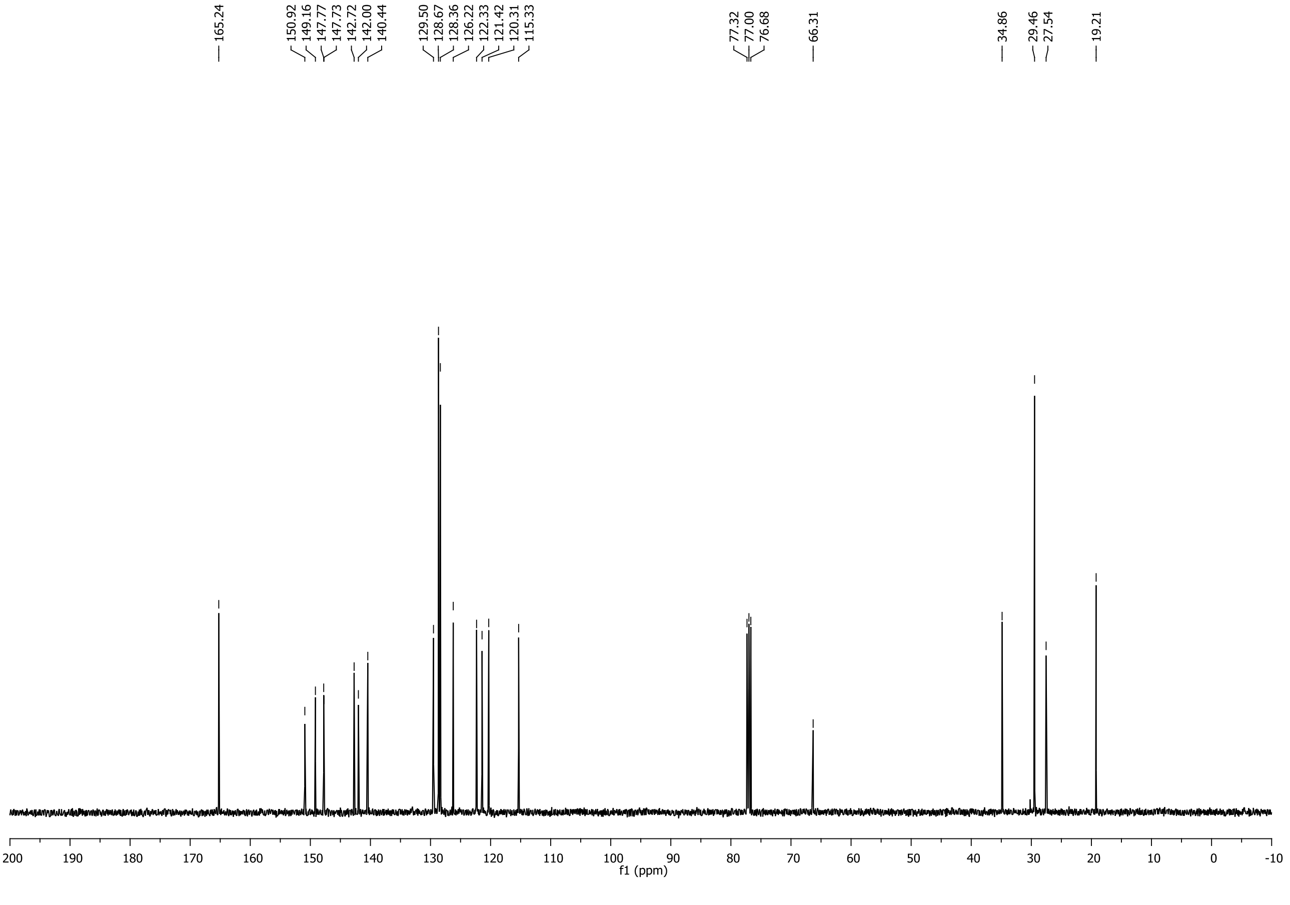
165.24
150.92
149.16
147.77
147.73
142.72
142.00
140.44
129.50
128.67
128.36
126.22
122.33
121.42
120.31
115.33
77.32
77.00
76.68
66.31
34.86
29.46
27.54
19.21
200 190 180 170 160 150 140 130 120 110 100 90 80 70 60 50 40 30 20 10 0 -10
f1 (ppm)

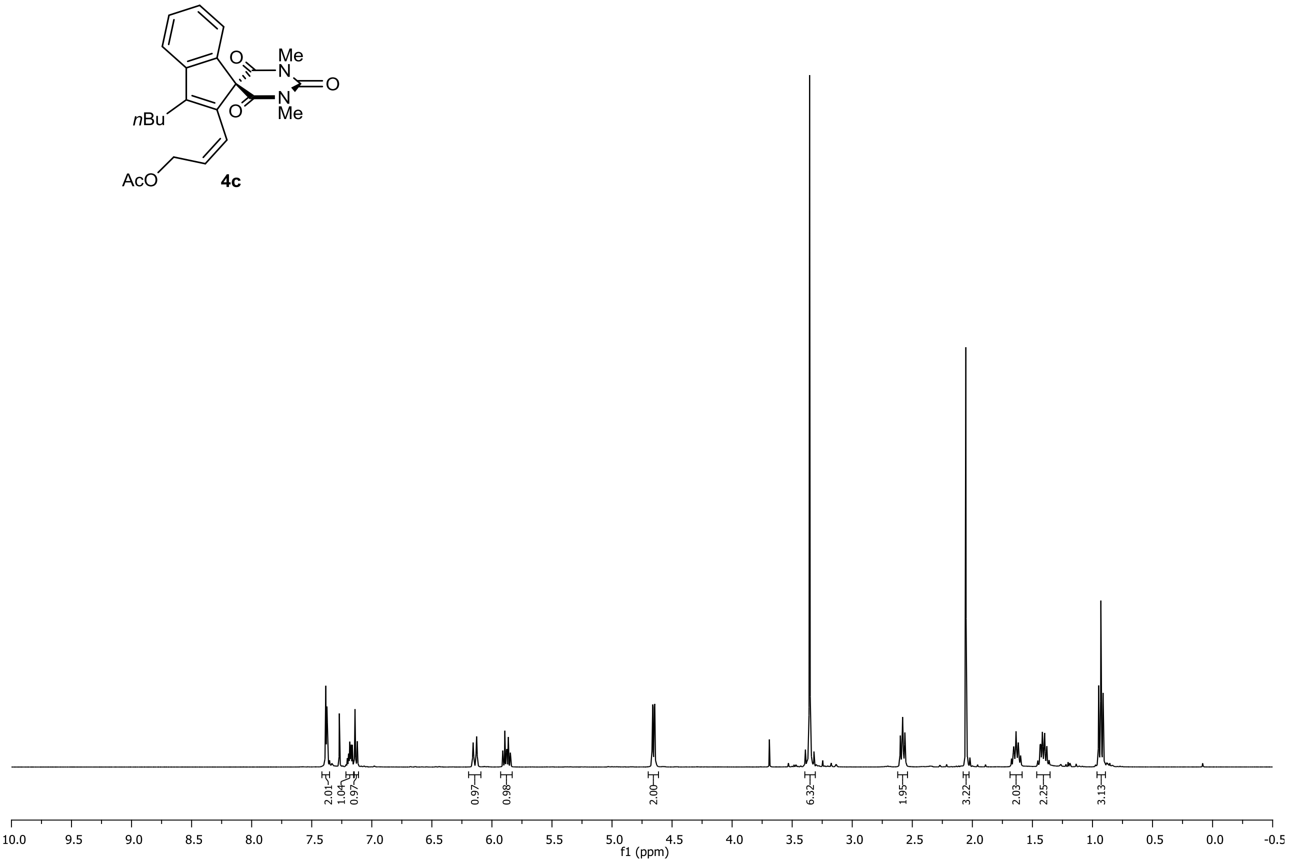
Me
N
O
O
O
N
Me
nBu
AcO
4c
2.01
1.04
0.97
0.97
0.98
2.00
6.32
1.95
3.22
2.03
2.25
3.13
f1 (ppm)

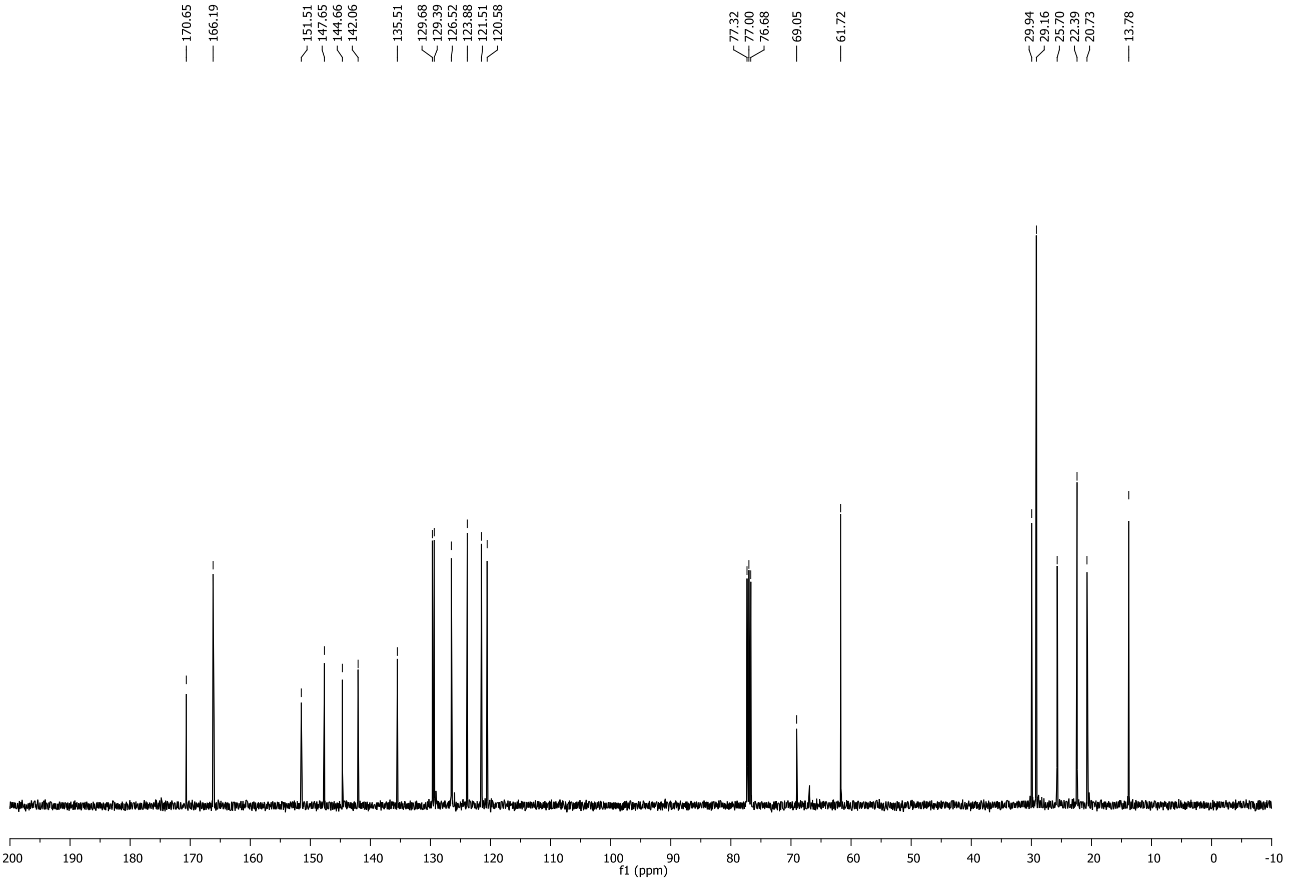
170.65
166.19
151.51
147.65
144.66
142.06
135.51
129.68
129.39
126.52
123.88
121.51
120.58
77.32
77.00
76.68
69.05
61.72
29.94
29.16
25.70
22.39
20.73
13.78
f1 (ppm)

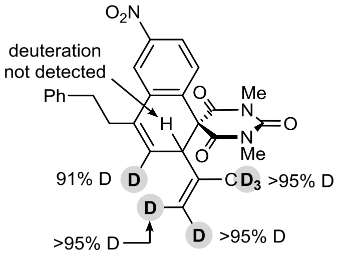
O2N
deuteration
not detected
Ph
H
O
Me
N
O
N
O
Me
91% D
D
C D3 >95% D
D
>95% D
D >95% D

$[D]_6$-**3n**

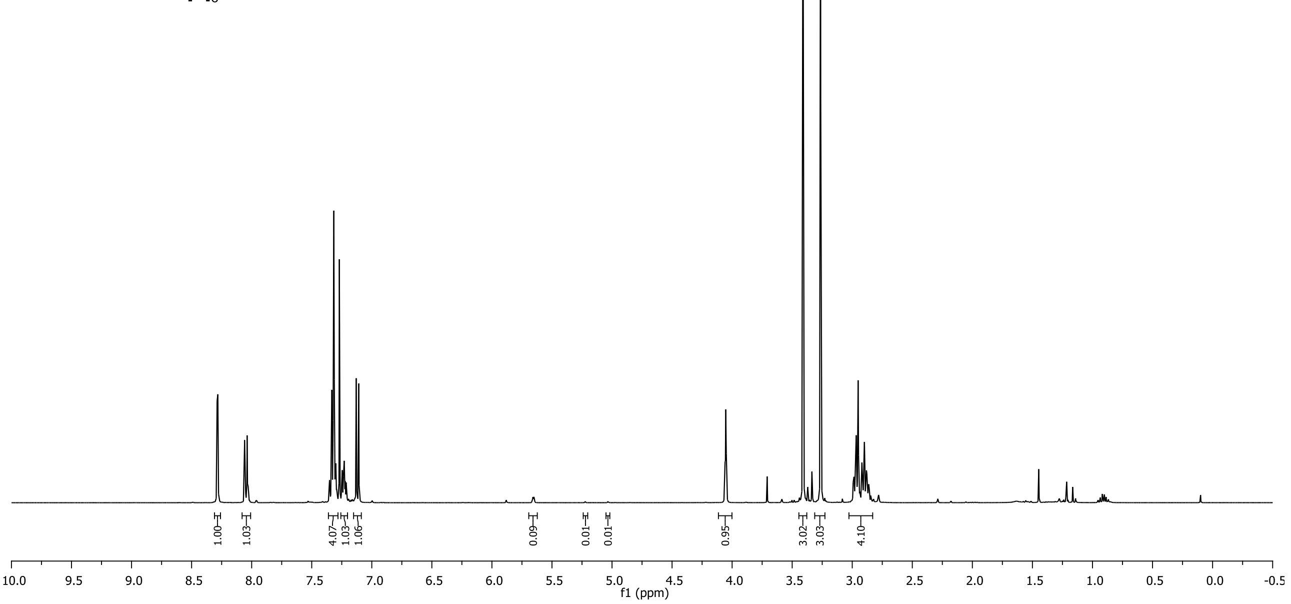
1.00
1.03
4.07
1.03
1.06
0.09
0.01
0.01
0.95
3.02
3.03
4.10
10.0 9.5 9.0 8.5 8.0 7.5 7.0 6.5 6.0 5.5 5.0 4.5 4.0 3.5 3.0 2.5 2.0 1.5 1.0 0.5 0.0 -0.5
f1 (ppm)

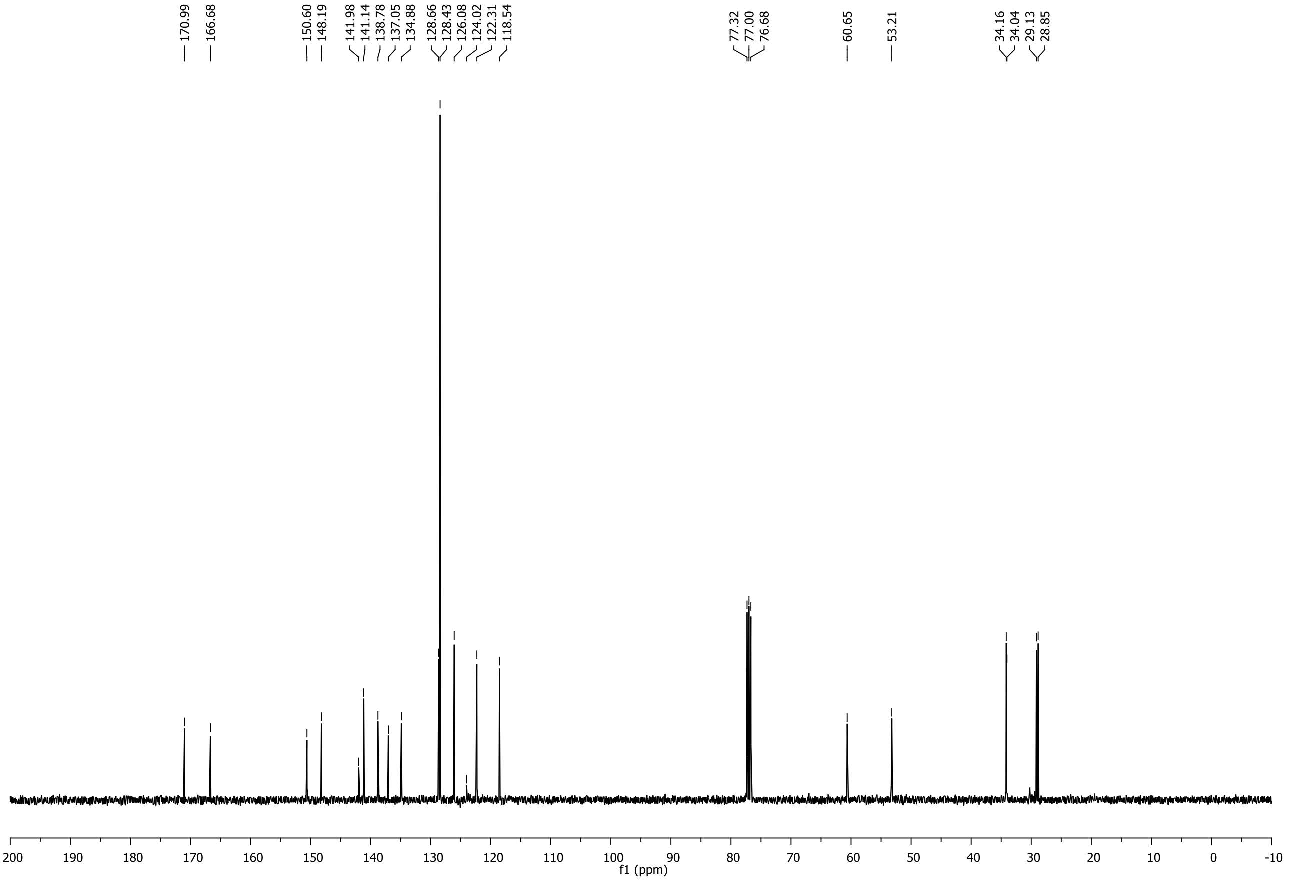
170.99
166.68
150.60
148.19
141.98
141.14
138.78
137.05
134.88
128.66
128.43
126.08
124.02
122.31
118.54
77.32
77.00
76.68
60.65
53.21
34.16
34.04
29.13
28.85
200 190 180 170 160 150 140 130 120 110 100 90 80 70 60 50 40 30 20 10 0 -10
f1 (ppm)

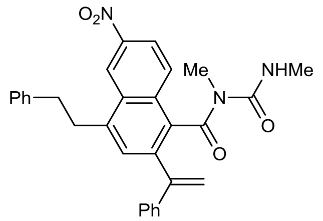
O2N
Me
NHMe
N
O
O
Ph
Ph

20

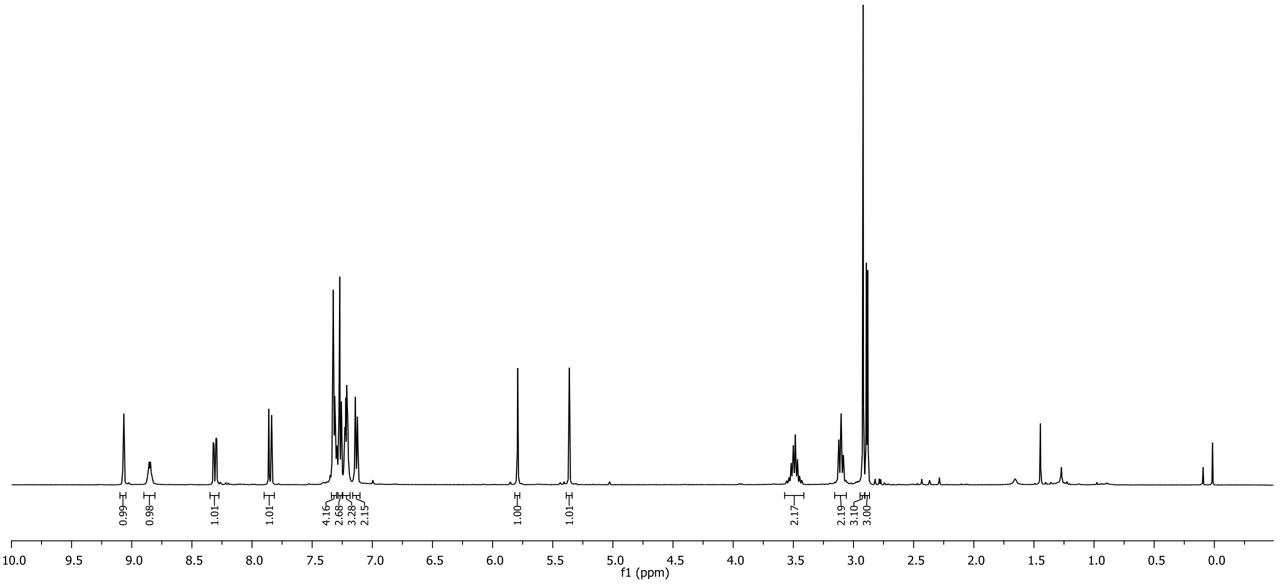
0.99
0.98
1.01
1.01
4.16
2.68
3.28
2.15
1.00
1.01
2.17
2.19
3.10
3.00
f1 (ppm)

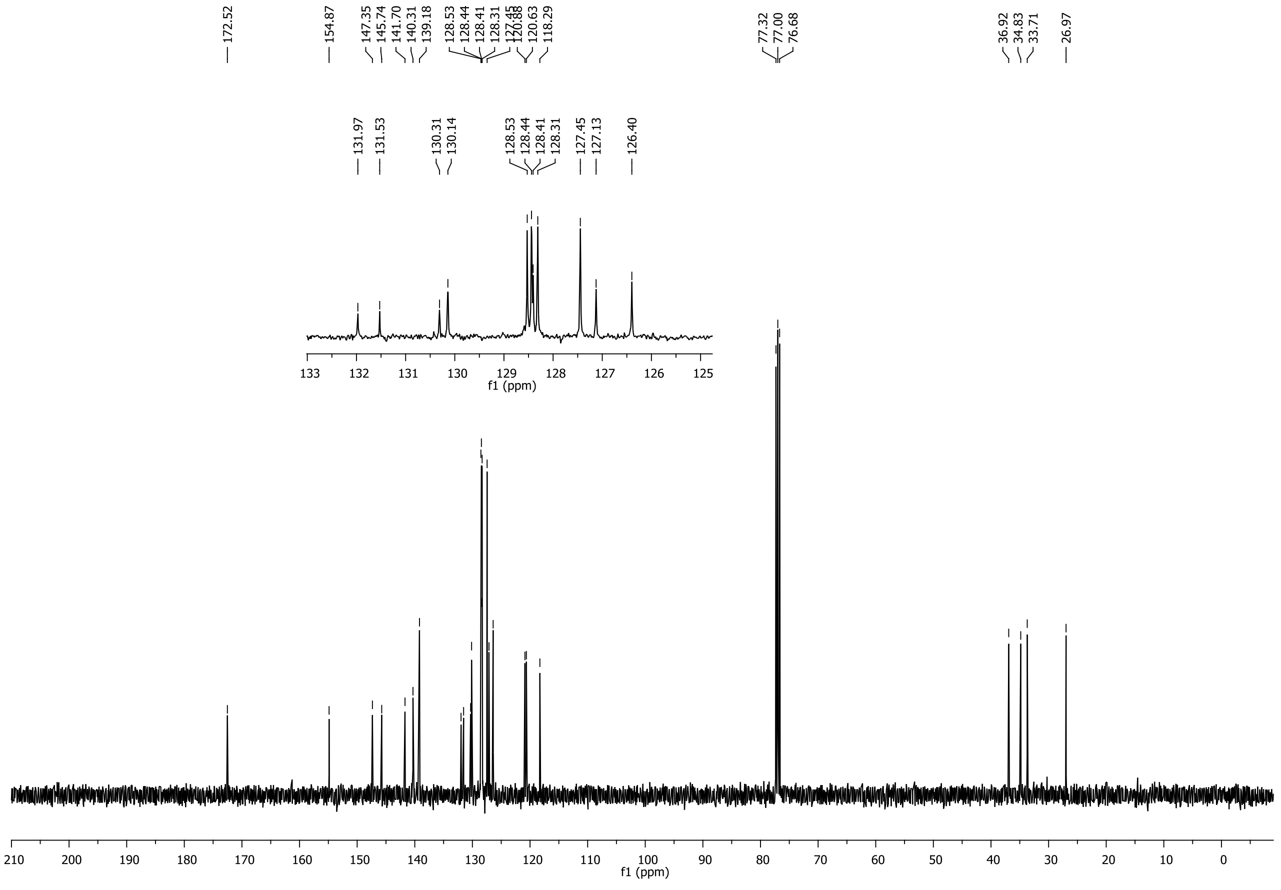
172.52
154.87
147.35
145.74
141.70
140.31
139.18
128.53
128.44
128.41
128.31
127.45
126.88
120.63
118.29
77.32
77.00
76.68
36.92
34.83
33.71
26.97
131.97
131.53
130.31
130.14
128.53
128.44
128.41
128.31
127.45
127.13
126.40
f1 (ppm)
